# A simple and highly accurate method for fuzzy edge-based 3D morphometry

**DOI:** 10.1371/journal.pone.0295988

**Published:** 2023-12-15

**Authors:** Li Jin

**Affiliations:** Department of Engineering Mechanics, State Key Laboratory for Strength and Vibration of Mechanical Structures, Xi’an Jiaotong University, Xi’an, China; Semnan University, ISLAMIC REPUBLIC OF IRAN

## Abstract

As the level of engineering technology increases, the mechanical problems posed in the area of mechanics become complex and diverse. The traditional measurement methods and content of past measurements no longer meet the needs, so more advanced experimental methods are needed to accurately measure the three-dimensional shape of objects and their deformations. Nitrate ester plasticized polyether (NEPE) propellants, are the highest energy solid propellant that have been applied in public reports in the world. Due to the material properties of the material itself, its mechanical properties cannot be accurately measured by conventional methods. This paper proposes a simple and highly accurate optical measurement method to study the volume change rate, Poisson’s ratio and true stress-strain curves of NEPE at different displacement loading rates of NEPE. In this paper, the strain of solid propellant NEPE in three directions during unidirectional stretching was measured by defocusing method and digital image correlation(DIC) method and the volume change rate, Poisson’s ratio and true stress-strain curves of NEPE under different stretching rates were obtained. The measured damage initiation engineering strain of NEPE at different tensile strain rates is 0.4687.

## 1 Introduction

The optical 3D morphology measurement method has made great progress, and some technologies have developed mature commercial products, including time-of-flight method [1–3], stereo vision method [4–6], grid line projection phase contour method [7, 8], etc. However, in recent years, with the development of industrial technology, the demand for dynamic measurement of three-dimensional morphology (i.e. deformation measurement) in mechanical experiments has become increasingly urgent. Some methods such as time flight method, monocular focusing method [9–13], and grid line projection method cannot be widely applied to measure three-dimensional deformation in mechanical experiments due to the need to scan objects, require multiple photos, and have high requirements for testing environments. At present, binocular stereo vision is the most commonly used method for measuring three-dimensional deformation of objects, with good measurement results. However, in some cases, such as measuring the deformation of objects under impact loads, maintaining the synchronization of the shooting requires a high cost. Moreover, the binocular stereo vision method requires two cameras to shoot at a certain angle, which not only poses occlusion issues but also imposes requirements on the experimental space. The 3D morphology measurement method based on a single photo can effectively overcome the above problems.

One of the major categories of 3D morphology measurement methods based on single images is monocular stereo vision, which is an improved hardware device for measuring 3D morphology from a single photo [14, 15]. The monocular stereo vision method obtains virtual stereo vision image pairs by attaching optical components. Due to its significant cost-effectiveness advantages, simple system, and avoiding complex camera synchronization issues, it has good application prospects in the field of experimental mechanics [16]. The monocular stereo vision described above requires two or even more photographs to fall separately on the camera sensor, so that the measured area on the object forms less than half the sensor size, which drastically reduces the resolution of the system and limits the application of the measurement system. The monocular defocus method has the advantages of simple algorithm and fast image processing speed, and has received widespread attention and in-depth research [17, 18] in recent years. The 3D morphology measurement method based on a single image has made significant progress, but with the continuous progress of technology, the requirements for product design are also increasing, and the performance requirements for measurement methods are also increasing. This method mainly develops towards high-speed, high-precision, high adaptability, automated processing, dynamic measurement, and convenient operation.

The highest energy solid propellant now used in public reporting around the globe is NEPE propellant, also known as nitrate ester plasticized polyether propellant (NEPE). In real-world applications, NEPE frequently supports difficult external loads, and the internal initial flaws will enlarge as a result of the loads, generating macro cracks that seriously compromise the mechanical characteristics of solid propellants and even compromise the structural integrity of motor grain, impairing the safety performance of weapon platforms [19]. Due to the significant difference between the stiffness of the matrix and the filled particle, stress concentration on the interface layer between the matrix and particles occurs when the material is subjected to external loads, which causes the matrix and particles to dehumidify and alters the material’s macromechanical properties [20]. NEPE exhibits high viscoelasticity for a polymer material, and the loading rate affects its mechanical properties. The foundation for determining the constitutive relations of NEPE propellant is accurate massive deformation measurement. The NEPE typically experiences necking at relatively low strain, which causes it to deform unevenly. Traditional extensometers are either ineffective or doubtful in this situation. Therefore, it is currently unclear how to assess the significant inhomogeneous deformation of NEPE, and new approaches are urgently needed.

By using the conventional strain measuring method, it is challenging to determine the specimen’s strain precisely because NEPE propellant exhibits non-uniform deformation, including necking. Currently, recording and analyzing photographs taken at various points throughout the tensile test is a common method for measuring the specimen’s deformation. Using optical methods, the real stress-strain curve of UHMWPE round rod specimens was measured [21]. By drawing a small square on the circular bar specimen and applying comparable testing methods, the strain at various locations was measured [22]. Through uniaxial tensile studies, the relationship between failure and Necking Deformation of thermoplastic crystalline polymer materials was examined [23]. In the experiment, the strain was determined by using the position change between seven sites. In order to better observe and analyse the effect of particle debonding within the NEPE solid propellant on the solid propellant damage, a fine-scale model of the realistically reduced NEPE solid propellant was established based on the scanning of it with X-rays [24–26]. Although it was anticipated in the aforementioned uniaxial tensile experiments that volume deformation would not occur during plastic deformation, volume deformation really occurs often during the tensile process. It is important to simultaneously measure the strain in the specimen’s three directions because the strain that was determined using the preceding premise had a significant experimental error. Fang et al [27, 28] used two cameras to simultaneously take pictures of the test piece’s front and side and concluded that the strain in the necking area did not change significantly along the width or thickness direction and could be replaced by their average values. Using the image correlation method (DIC) and pictures of the test piece’s front and side, the shrinkage strain in the test piece’s thickness and width directions, respectively, as well as the axial tensile strain, were determined. In contrast to the actual circumstance, Fang’s solution reduced the necking region’s cross-sectional shape to a rectangle. Additionally, the average strain was obtained from the entire field. Moreover, the full field strain obtained is the average strain of the necking region, and it is impossible to measure the full field strain anywhere on the surface of the specimen. In a previous work [29], a damage-containing phase-only isostructural model for NEPE solid propellants was proposed based on the results of mechanical property measurements of NEPE solid propellants by DIC and defocusing methods. And this paper focuses on the detailed description and validation of the optical measurement principle.

In this paper, the main content of this paper is to introduce the derivation process of the principle of the defocusing ranging method proposed, and then the accuracy of the method is experimentally verified. The mechanical properties of NEPE solid propellant were measured optically based on this method in this paper. In order to study on mechanical properties of solid propellant NEPE, the defocusing method and DIC method are used to measure the strain of solid propellant NEPE in three directions during unidirectional stretching. The volume change rate, Poisson’s ratio and true strain curves of NEPE under different stretching rates are obtained.

## 2 The research methods

### 2.1 The principle of overfocus and underfocus imaging

**Fig 1:**
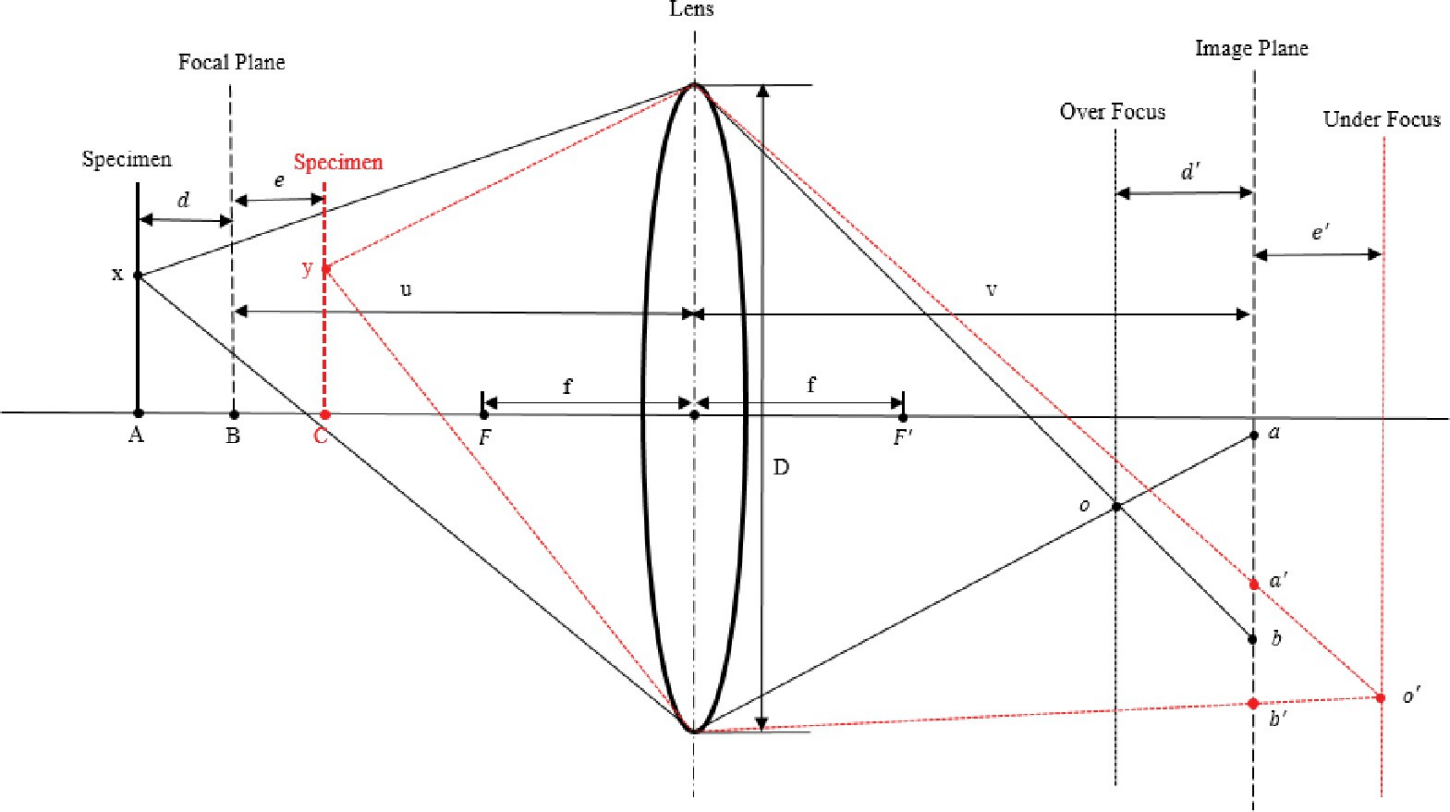
Camera imaging optical path diagram under overfocus and underfocus conditions.

A light source on an object falls onto the camera sensor through the lens of the lens, forming the image of that point. The points on the object plane have only one fully focused imaging plane for the lens system. When the imaging plane is offset, the image formed by the point light source on the imaging plane is a diffuse circle. The farther the deviation, the larger the diameter of the dispersion circle formed. There is a one-to-one correspondence between the diameter of the dispersion circle and the deviation distance [30]. So the defocus distance of an object can be obtained by evaluating the diameter of the dispersion circle. The relationship between the diameter of the dispersion circle and camera parameters can be obtained from the camera’s optical path diagram, as shown in **Fig 1**.

Once the camera parameters are given, a clearly focused object plane can be determined and defined as the focal object plane. When the object is in front of the focused object plane and the imaging plane is in front of the camera sensor, it is called an overfocus state. When the object is behind the focused object plane and the imaging plane is behind the camera sensor, it is called an underfocus state. As shown in **Fig** 1, the imaging optical path of the object under overfocus and underfocus conditions is shown. *f* is the focal length of the camera, *D* is the diameter of the camera lens, *u* is the object distance when the focus is clear, and *v* is the image distance when the focus is clear. As shown in **Fig** 1, when the object is located on the focusing plane, it forms a clear image on the camera sensor. When an object is located at point *x*, the distance it deviates from the focused object plane is *d*, and its image plane also deviates from the focused image plane forward, with a deviation distance of *d*′. A point *x* on the object forms a blurry circle with a diameter $D_{O}=\bar{ab}$ on the camera sensor. On the other hand, defocusing is the opposite of over focusing. A point *y* on the object deviates backwards from the focusing image plane by a distance of *e*′, forming a blurry circle with a diameter $D_{U}=\bar{a^{\prime }b^{\prime }}$ on the camera sensor. By using optical path **Fig** 1, a theoretical formula for the diameter of the dispersion circle with respect to camera parameters can be derived.

The lens equation of a convex lens is:

$$\frac{1}{u}+\frac{1}{v}=\frac{1}{f}$$ (1)

From **Eq** 1, it can be determined that point *x* satisfies the following equation in the overfocus state:

$$\frac{1}{u+d}+\frac{1}{v-d'}=\frac{1}{f}$$ (2)

According to the principle of triangle similarity, it can be concluded that

$$\frac{\bar{ab}}{D}=\frac{d'}{v-d'}$$ (3)

By combining **Eqs** 1, 2, and 3, the diameter of the dispersion circle can be obtained as:

$$D_{O}=\bar{ab}=D\cdot (\frac{v}{v-d'}-1)=D\cdot v\cdot (\frac{1}{f}-\frac{1}{u+d}-\frac{1}{v})=D\cdot v\cdot \frac{u+d-f}{f(u+d)}-D$$ (4)

Similarly, the formula for the diameter of the dispersion circle at point *y* in the case of underfocus is

$$D_{U}=-D\cdot v\cdot \frac{u-e-f}{f\cdot (u-e)}+D$$ (5)

Due to the fact that camera parameters are all fixed, i.e. *u*, *v*, *f*, and *D* are constants, the diameter of the dispersion circle *D*_*O*_ and *D*_*U*_ are only related to the defocus distance *d* and *e*. The larger the defocus distance, the larger the diameter of the dispersion circle. Therefore, as long as the diameter of the dispersion circle at a certain point in the image can be measured, the defocus distance of that point can be measured using **Eqs**xref 4 and 5. By measuring the diameter of the dispersion circle at all points on the entire image, the morphology information of the measured object in the depth direction can be obtained.

### 2.2 Light intensity distribution in areas with blurred stripe features

**Fig 2:**
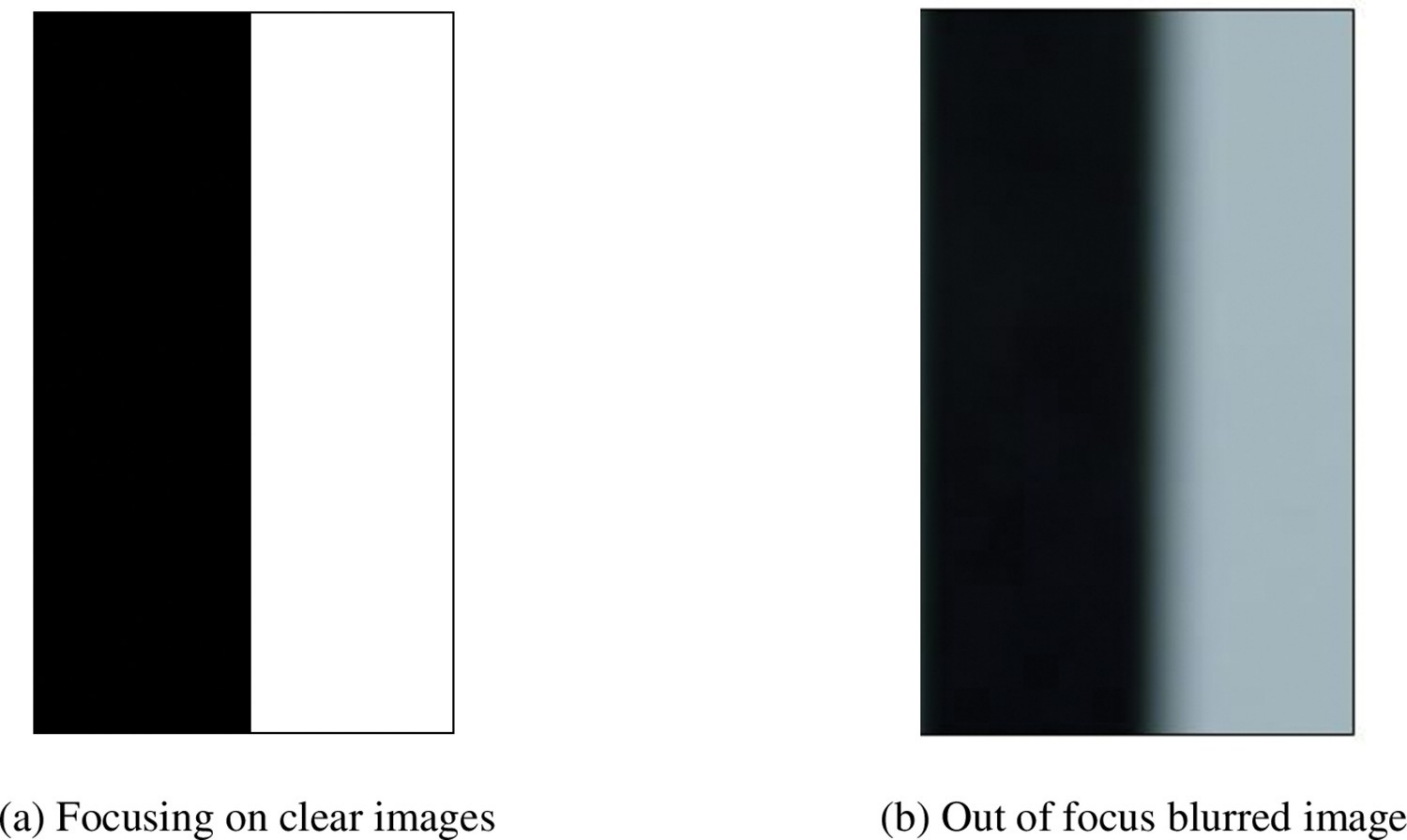
Stripe patterns with different focusing situations. (a) Focusing on clear images. (b) Out of focus blurred image.

For objects with striped surface features, blurry bands will appear on the fringe boundary of their defocused image. **Fig** 2(A) shows a clearly focused boundary map and **Fig** 2(B) shows a defocused blurry image of an object with striped features. According to the optical path **Fig** 1, it is easy to know that the width of the blur band is equal to the diameter of the dispersion circle. From the previous section, it is known that if the starting and ending points of the blurry band can be accurately measured, the defocus distance of the object can be directly obtained based on the difference between the starting and ending points of the blurry band. However, in reality, due to the fact that only discrete whole pixel information can be obtained from the image, coupled with the influence of noise, the measurement effect of directly measuring the start and end points of the blur band to determine the width is not ideal. This article calculates the width of the fuzzy band based on the distribution of light intensity inside the fuzzy band. This not only reduces the impact of edge noise on the measurement results, but also determines that the pixel width of the fuzzy area can be non-integer. This will greatly improve the accuracy of the measurement method proposed in this article.

The principle of converting grayscale images to light intensity maps is as follows. Along the direction perpendicular to the stripe boundary, an ideal stripe can be regarded as a one-dimensional edge function, whose mathematical expression is:

$$I_{0}(x;W,B,x_{e})=(W-B)u(x-x_{e})+B$$ (6)

The expression of the edge function is:

$$u(x,y)=\{\begin{array}{l} 1,\sqrt{{\left(x+R_{1}\right)}^{2}+y^{2}}\leq R_{1}\\ 0,others \end{array}$$ (7)

Where: *I*_0_ is the light intensity of a clear image; *W* and *B* are the light intensities of two different colored stripes on a clear image, namely the maximum and minimum light intensities on both sides of the stripe boundary; *x*_*e*_ is the position of the fringe boundary; *u*(⋅) is one dimensional ideal step equation. For simplicity, if *x*_*e*_ is set to zero, **Eq** 6 can be simplified as follows:

$$I_{0}(x)=(W-B)u(x)+B$$ (8)

According to geometrical optics, the internal light intensity of the dispersion circle formed by a point on the object on the sensor plane is evenly distributed [31]. The point spread function can be written as follows:

$$h(x,y)=\{\begin{array}{l} \frac{1}{\pi R^{2}},\sqrt{x^{2}+y^{2}}\leq R\\ 0,others \end{array}$$ (9)

Where, *R* is the radius of the dispersion circle.

Since the light intensity of the blurred picture is equal to the convolution of the light intensity of the clear picture and the point spread function, the expression is as follows:

$$I=I_{0}*h(x,y)$$ (10)

By combining **Eqs** 8, 9, and 10, the expression for the light intensity of blurred images can be obtained as follows:

$$\begin{array}{l} I=I_{0}*h(x,y)=B+(W-B)\cdot u(x)*h(x,y)\\ =B+(W-B)\int_{-\infty }^{+\infty }\int_{-\infty }^{+\infty }u(x-t)\cdot h(t,s)dtds\\ =B+(W-B)\int_{-\infty }^{+\infty }\int_{-\infty }^{x}h(t,s)dtds \end{array}$$ (11)

In summary, the theoretical formula for the intensity of light in the blurred area of a striped image is a piecewise function, with the following form:

$$\begin{array}{l} I=I_{0}*h(x,y)=B+(W-B)\int_{-\infty }^{+\infty }\int_{-\infty }^{+\infty }u(x-t)h(t,s)dtds\\ =\left\{ \begin{array}{l} W,x>R\\ B+\frac{\left(W-B\right)}{\pi R^{2}}\int_{-R}^{x}2\sqrt{R^{2}-t^{2}}dt,-R\leq x\leq R\\ B,x<-R \end{array}\right.\\ =\left\{ \begin{array}{l} W,x>R\\ B+\frac{\left(W-B\right)}{\pi }(\arccos(\frac{-x}{R})+\frac{x}{R}\sqrt{1-\frac{x^{2}}{R^{2}}}),-R\leq x\leq R\\ B,x<-R \end{array}\right. \end{array}$$ (12)

**Fig 3:**
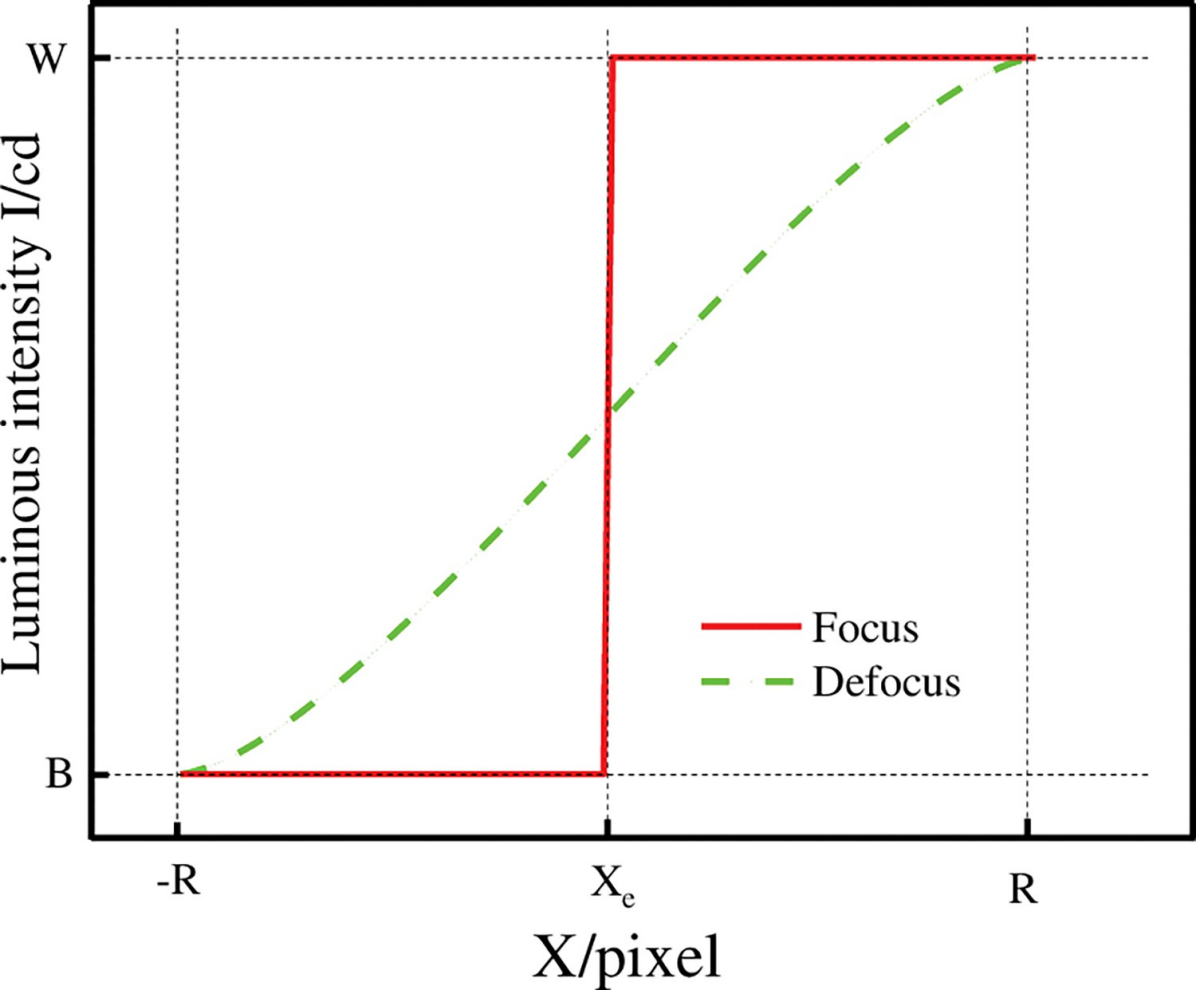
Theoretical light intensity distribution with clear and fuzzy boundaries.

According to **Eq** 12, the theoretical light intensity distribution of the boundary in the clear picture and the blurred picture can be obtained, as shown in **Fig** 3. It can be seen from the figure that the theoretical light intensity when focused clearly is an edged edge, while the light intensity distribution at the location of the boundary when out of focus blurred is a gradual process and centrosymmetric with point (*x*_*e*_,(*W*−*B*)/2) as the centre of symmetry. Its blurred area ranges from −*R* to *R*. So for the streak feature, the blurred picture forms a blurred band on the streak boundary with a width equal to the diameter of the diffuse circle.

### 2.3 Procedure for calculating the fuzzy width

**Fig 4:**
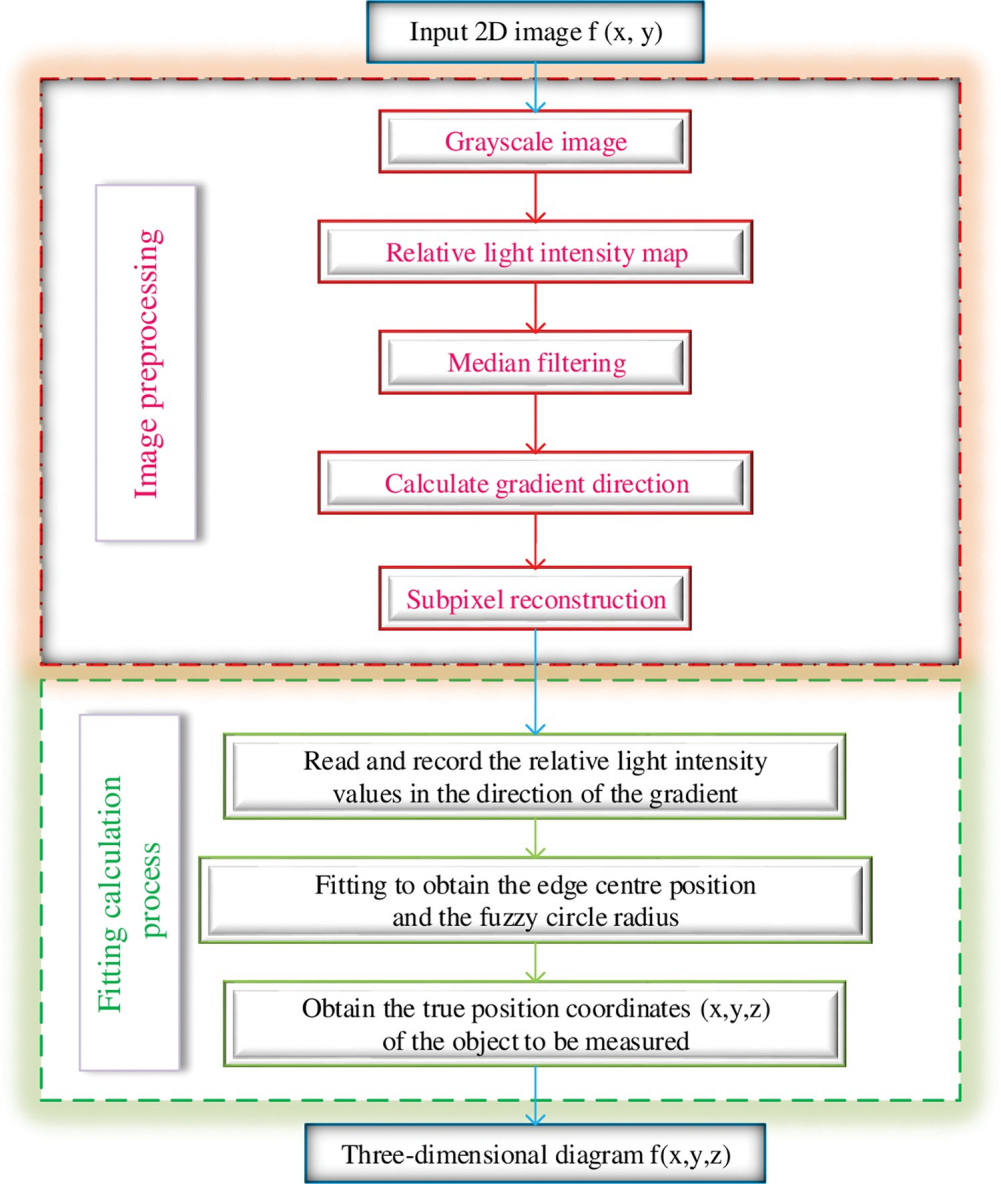
Calculation flow chart.

Through the theoretical derivation in the previous section, the law of light intensity distribution inside the blurred area is obtained. Based on this law, the blur width of the image can be calculated by combining the distribution of the light intensity of the actual image within the blurred area. In order to accurately calculate the blur width, this paper uses the process shown in the **Fig** 4, which can accurately calculate and restore the 3D shape of real objects.

#### 2.3.1 Grayscale of digital images

In this chapter, the camera used is a colour digital camera and the resulting image is a colour digital image, known as a three-color RGB image. The process of converting a colour digital image into a greyscale image is the greyscaling of the digital image.

Each pixel in a colour digital image has three components: red (*R*), green (*G*) and blue (*B*), each of which has 256 pixel values, so a single pixel has a range of more than 16 million (256*256*256) colour variations, which greatly affects the speed of image processing. Where possible, colour images are converted to greyscale by image conversion, so that each pixel has only one byte to store the greyscale value, which is an integer value in the range [0–255], reducing the amount of computation and speeding up the image processing. It is worth pointing out that although some colour levels are lost, the descriptions of the greyscale and colour maps are consistent in terms of the overall and local distribution of brightness levels in the image.

There are three commonly used methods of image greyscale processing, namely the maximum, average and weighted image greyscale methods:

1) Graying the image by the maximum value method

The maximum value of the three colour components *R*, *G* and *B* in a colour digital image is taken as the grey value Gray of the grey scale image. the expression is as follows:

Gray(i,j)=max(R(i,j),G(i,j),B(i,j)) (13)

2) Mean value method of image greyscaling

The values of the three colour components *R*, *G* and *B* in a colour digital image are averaged and this average is then used as the grey scale value of the grey scale image. The expression is as follows:

Gray(i,j)=(R(i,j)+G(i,j)+B(i,j))/3 (14)

3) Weighted image greyscaling

The values of *R*, *G* and *B* are weighted and averaged with different weights, taking into account the needs of the actual analysis, the importance of each colour component and other judgement indicators. There are two common weighting methods, the first being the OnpenCV open library grey-scale weighting method:

Gray(i,j)=0.212671R(i,j)+0.715160G(i,j)+0.072169B(i,j) (15)

The second weighting method proposes a weighting from the perspective of human physiology. Studies have shown that the human eye is most sensitive to green *G* and least sensitive to blue *B*. The specific weighting formula is shown below:

Gray(i,j)=0.299R(i,j)+0.587G(i,j)+0.114B(i,j) (16)

This is because the average of the different weighted values in **Eq** 16, based on the sensitivity of the human eye to different colours, gives the most reasonable grey-scale image. The features of the image are retained intact. Therefore, in later chapters of this thesis, **Eq** 16 is used to greyscale the acquired colour images.

#### 2.3.2 The conversion relationship between image grayscale value and light intensity

From the derivation in section 2.2, the theoretical distribution of the light intensity near the blurred area is obtained. However, from the picture, information can only be obtained about the grey scale values or *RGB* values. It is not possible to obtain information about the light intensity directly. So before the blur width is calculated, the grey scale values of the picture need to be converted into the light intensity received by the camera sensor.

A series of pictures are taken of an object with constant ambient lighting. The light intensity received by the camera sensor is influenced by the exposure time of the camera, the aperture value and the distance between the camera and the subject (in the case of small distances). When the other parameters of the camera are kept constant and only the exposure time is changed, the light intensity received by the camera sensor is proportional to the exposure time. Therefore, it is possible to establish a relationship between light intensity and greyscale values by reading the greyscale values of photographs of the same object taken at different exposure times.

**Fig 5:**
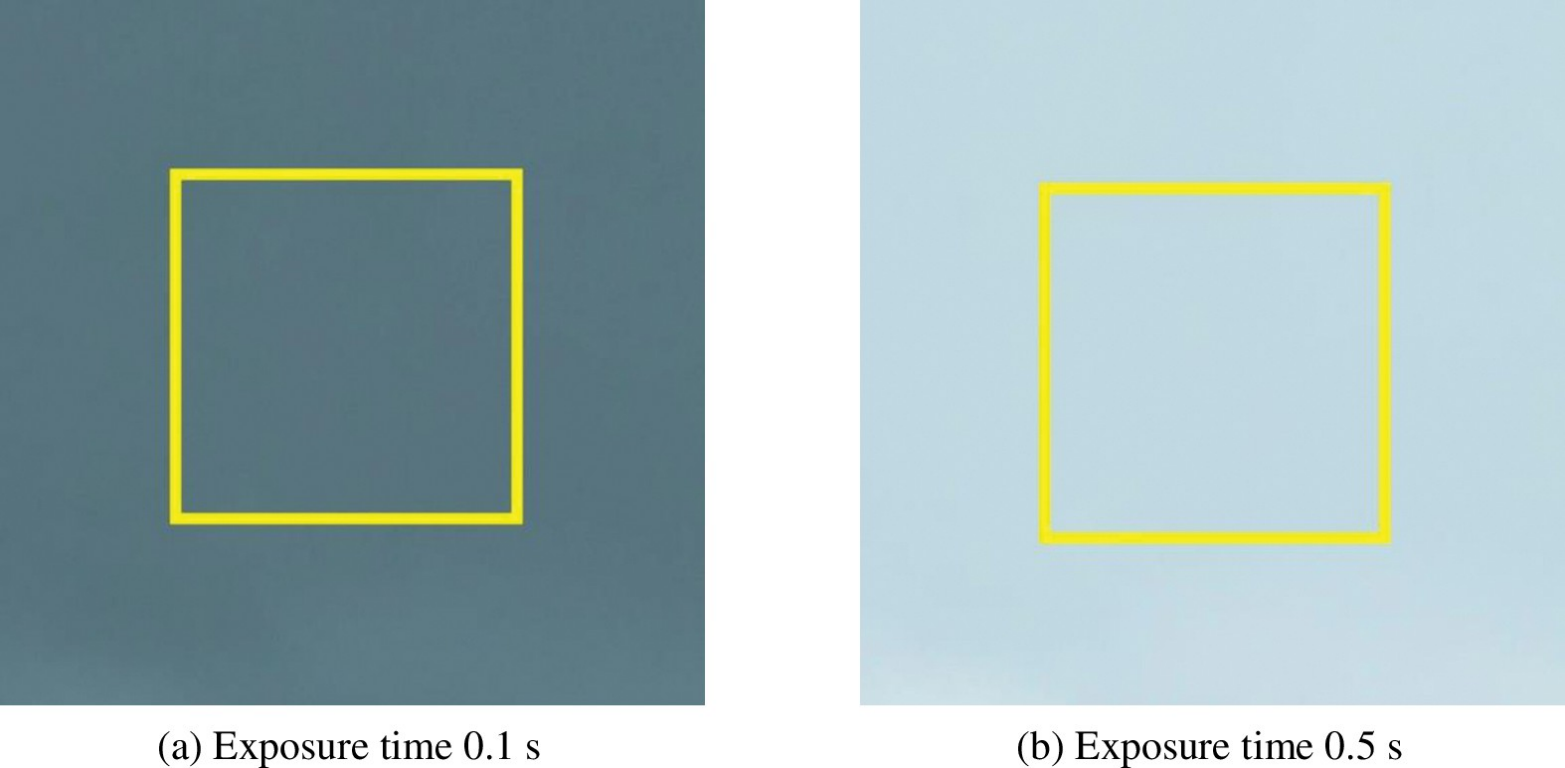
Photographs of the same object at different exposure times. (a) Exposure time 0.1 s. (b) Exposure time 0.5 s.

The calibration experiments were carried out using a Nikon D800 camera with a fixed focal length lens of 60 mm and an aperture value of *F = 4*.*5*. A series of photographs with different exposure times (increasing from 0.001 s to 3 s) were taken of a white flat plate. **Fig** 5 shows two of these photographs with exposure times of 0.1 s and 0.5 s. In this paper, a given exposure time is defined as the reference exposure time t*, at which point the light intensity received by the camera sensor is defined as the reference light intensity I*. Then the light intensity *I* at the other exposure times can be obtained according to the following equation:

$$I=\frac{t}{t^{*}}I^{*}$$ (17)

**Fig 6:**
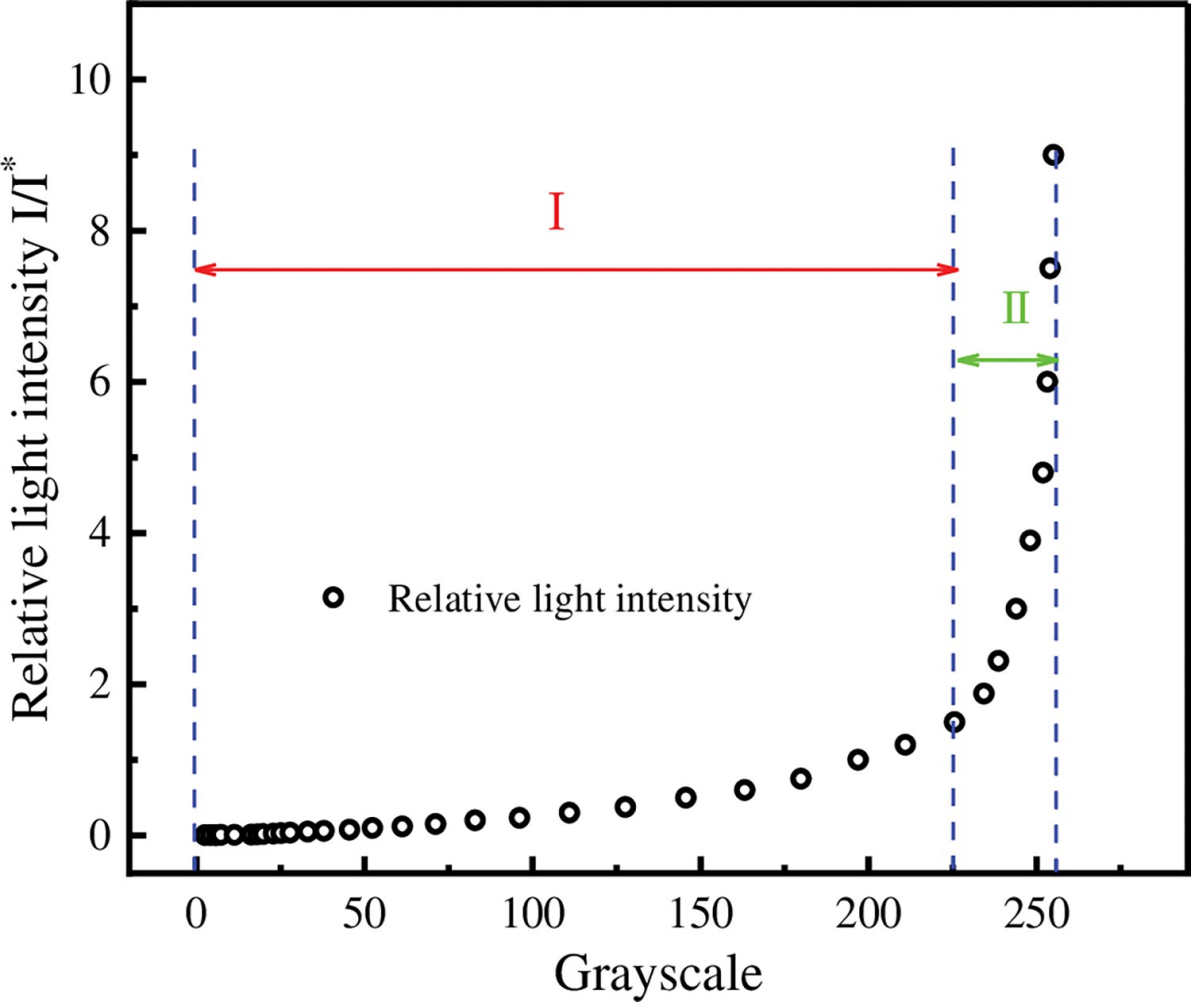
Grey scale values versus relative light intensity curve.

In this paper, I*/*I* is defined as the relative light intensity. The relative light intensities at other exposure times can be obtained from **Eq** 17. The procedure is as follows: read the grey scale values of this series of photographs in the same area (the area inside the yellow box in **Fig** 5) separately. The average of the greyscale values within that area is taken and defined as the average greyscale value for that image. The exposure time t = 0.5 s is then defined as the reference exposure time. The light intensity received by the camera sensor at this point is defined as the reference light intensity. The relative light intensities at other exposure times can be obtained according to **Eq** 17. Finally, the relative light intensities at the same exposure time and the average greyscale value of the picture are matched one to one. A relationship curve between the relative light intensity and the greyscale value is established and the results are shown in **Fig** 6. Because the relationship curve is determined by the characteristics of the camera sensor, it applies to all pictures taken by the same camera.

In **Fig** 6, the horizontal coordinates are the grey scale values and the vertical coordinates are the relative light intensities. As can be seen from the graph, the relationship between the grey scale value and the relative light intensity is non-linear. In general, the relative light intensity increases monotonically with the grey value. However, the rate of increase in relative light intensity varies from stage to stage. In stage I, when the grey level is small, the relative light intensity increases more slowly with increasing grey level. In stage II, when the grey level is large, the light intensity received by the sensor is close to saturation. The relative light intensity increases at an increasing rate as the grey value increases. In order to reduce errors, in this paper we only measure areas of the photograph where the grey value is less than 225. A quadratic polynomial fit to the data points in **Fig** 6 with grey values less than 225 yields the following equation for the relationship between grey value and relative light intensity:

$$I/I^{*}=0.0018+1.75965\times 10^{-4}G+4.49591\times 10^{-5}G^{2}-3.25837\times 10^{-7}G^{3}+1.12366\times 10^{-9}G^{4}$$ (18)

Where: G is the grey value of the photograph Gray and I*/*I* is the relative light intensity.

#### 2.3.3 Image denoising

The various factors in a digital image that prevent a person from accepting and understanding the information in a digital image. This can be called noise in digital images. Noise in digital images can theoretically be defined as unpredictable. Random errors that can only be recognised in a probabilistic statistical way.

This is because image noise can affect the accuracy of the measurement results. Therefore, the image needs to be filtered to remove the noise from the image before the measurement. The object under study in this paper is a blurred image of an object with striped features on the surface. The internal variation of the target region in the image is generally flat or even largely constant. Due to the effect of noise, the grey values of the picture fluctuate and change within a small area. It is important to note that the method proposed in this paper restores the three-dimensional shape of the object according to the degree of blurring of the image. Therefore, it is important to remove the noise while ensuring that the detailed information of the image at the edges of the stripes does not change. The filtering effect and the time spent are taken into account. In this paper, the median filter of the non-linear filter is used to remove the noise from the image.

The median filter sets the grey value of each point to the median of the grey values of all points in the neighbourhood window of that point, and is also known as a ranking statistics filter. It is a non-linear signal processing technique based on the theory of ranking statistics that effectively suppresses noise. It is because the median filter algorithmically replaces noisy points in the image that differ significantly from the surrounding pixel points with intermediate values within the neighbourhood window. And it can eliminate random noise and smooth out volatility. At the same time it prevents the digital image from being blurred during the noise removal process. Effectively retains the characteristic edge information of the digital image. This is the most important reason for applying median filter for noise removal in this paper.

The specific steps in median filtering are as follows:

1. Allow the filter’s sampling window to roam across the entire digital image and coincide the centre of the window with a particular pixel point of the digital image;
2. Read and record the grey-scale values of all the pixel points in the sampling window;
3. Arrange these grey values from smallest to largest, reading the grey value in the middle of the row;
4. Assign the middle grey value to the pixel in the centre of the sampling window.

**Fig 7:**
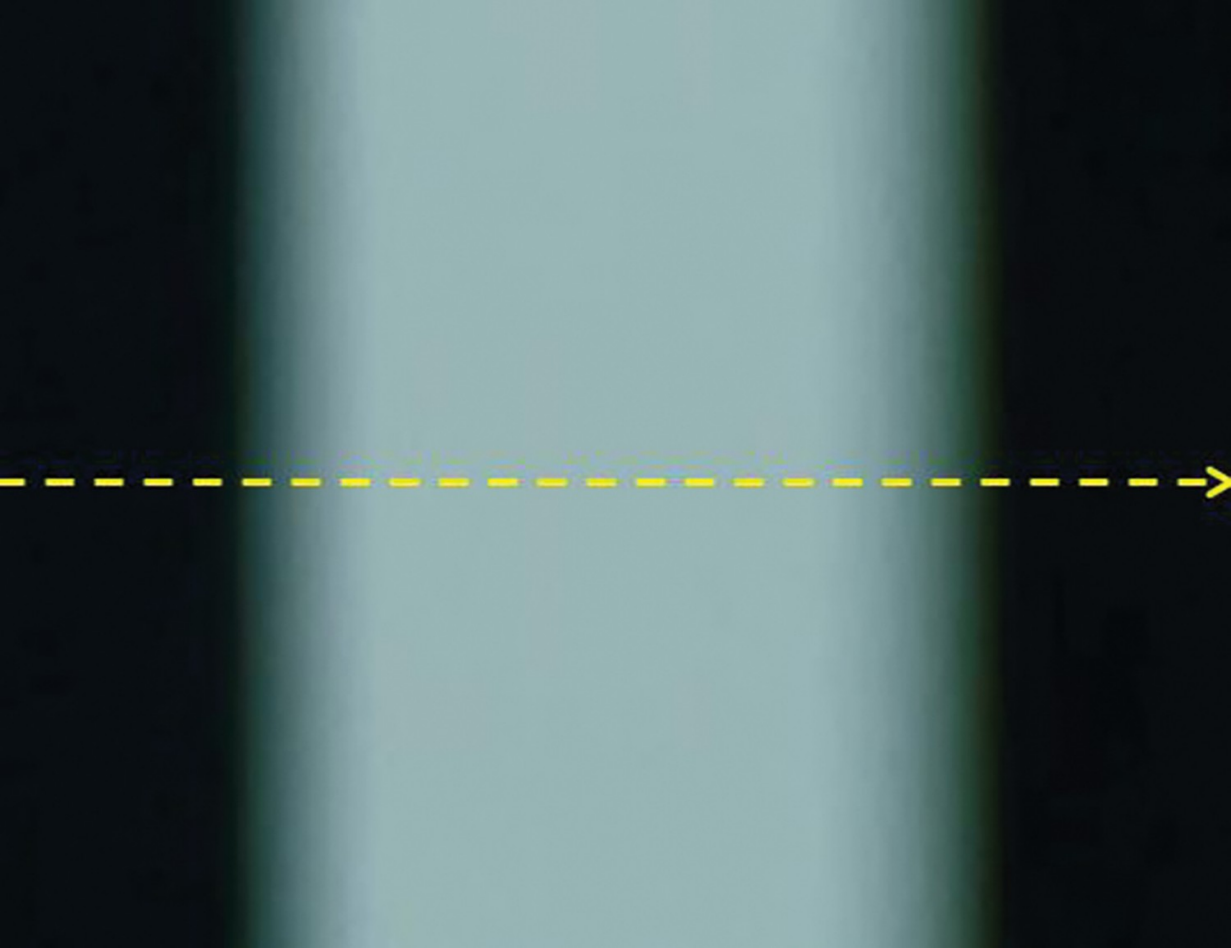
Out-of-focus blurred image.

The following is an analysis of the median filtered denoising process for the actual image taken. **Fig** 7 shows a partial intercept of the actual out-of-focus blurred image. First, the colour digital image is greyed out. The greyscale values in the image were then converted into relative light intensities according to the method described in section 2.3.2. Finally the relative light intensities of the images were denoised using median filtering.

**Fig 8:**
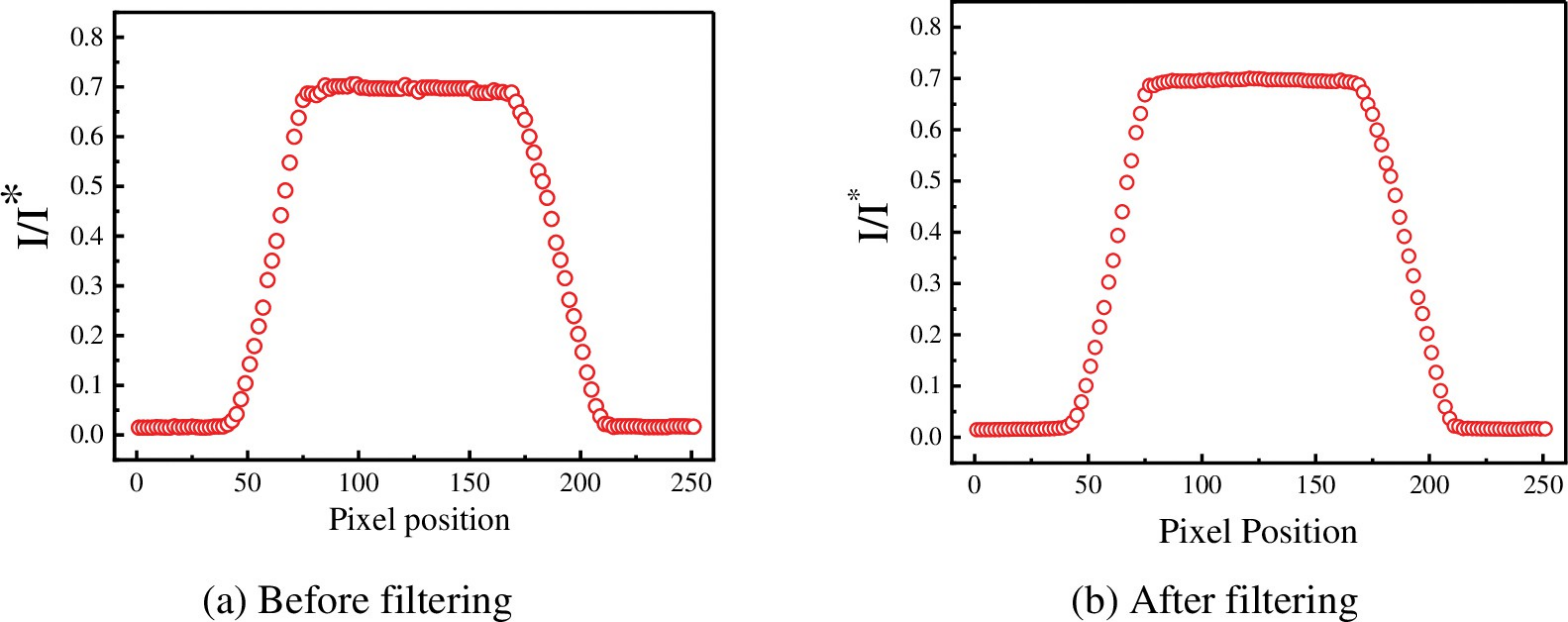
Relative light intensity distribution of the actual image before and after denoising. (a) Before filtering. (b) After filtering.

**Fig** 8(A) shows the relative light intensity distribution before median filtering for a single row of pixels at the yellow dotted line in **Fig** 7. **Fig** 8(B) shows the relative light intensity distribution after filtering. The comparison of these two figures shows that the noise in the image has been effectively removed. Especially at the edges of the streak blur width, the light intensity distribution of the filtered image becomes smooth. The volatility is nicely removed. Crucially, median filtering avoids the loss of effective information at the boundaries of the digital image.

#### 2.3.4 Blurred width of arbitrary directional stripes

In the previous chapters, the stripes in the pictures are all vertical. By reading the relative light intensity values of the pixel points along the horizontal direction, the light intensity distribution needed for the method proposed in this chapter can be obtained. For stripes in any direction, it is necessary to first find the direction perpendicular to the stripes. This is the direction of the gradient of light intensity, which is denoted as direction *L*. The light intensity distribution is then read at one pixel intervals along the direction *L*. The gradient of light intensity is the direction of the gradient of light intensity.

The gradient direction of the light intensity is the direction of the most dramatic change in light intensity. By means of sub-pixel reconstruction, the light intensity of sub-pixel points at the same distance as the pixel point under test is obtained. These sub-pixel points form a circle with the pixel under test as the centre of the circle. Among these sub-pixel intensities, the sub-pixel with the greatest absolute difference from the measured point is searched for. The direction of the line between it and the measured pixel is the direction of the light intensity gradient. When reconstructing a sub-pixel by interpolation, a sub-pixel is obtained by interpolation at certain angular intervals on a circle with the measured pixel point as the centre. In order to reduce the computation time, the Sobel operator is first applied to calculate an initial value of the gradient direction.

*a) Sobel operator calculates the initial value of the gradient direction*. The Sobel operator is an important operator for edge detection in digital images. It detects edges by finding the gradient of a pixel point through a convolution operation. Thus, it is able to find the direction of the gradient of a point on the image. the Sobel operator considers that pixels within a neighbourhood window have unequal influence on the current pixel. So pixels at different distances have different weights. The Sobel operator consists of two matrices, a horizontal and a vertical matrix. Convolution of these two matrices with the image respectively gives an approximation of the horizontal and vertical gradients as follows:

$$\left\{ \begin{array}{l} G_{x}=\left[\begin{array}{ccc} -1 & 0 & 1\\ -2 & 0 & 2\\ -1 & 0 & 1 \end{array}\right]*A\\ G_{y}=\left[\begin{array}{ccc} -1 & -2 & -1\\ 0 & 0 & 0\\ 1 & 2 & 1 \end{array}\right]*A \end{array}\right.$$ (19)

Where: *G*_*x*_ is the approximate gradient in the horizontal direction; *G*_*y*_ is the approximate gradient in the vertical direction; and *A* is the original image. The gradient of each pixel point on the image can be expressed by the following equation:

$$G_{pixel}=\sqrt{G_{x}{}^{2}+G_{y}{}^{2}}$$ (20)

The direction of the gradient can be calculated using the following equation:

$$\theta =\arctan(G_{y}/G_{x})$$ (21)

Once the direction of the light intensity gradient has been obtained. The relative light intensity values are read at intervals of one pixel width along that direction. Because the position coordinates of the read data may be non-integer pixels. It is therefore necessary to obtain the intensity values of the non-integer pixels by sub-pixel reconstruction.

*b) Sub-pixel reconstruction*. Commonly used interpolation methods for subpixel reconstruction are: bilinear interpolation and binary cubic spline interpolation. Bilinear interpolation is a relatively simple interpolation. The light intensity of the subpixel can be obtained by linear interpolation of the light intensities of the four neighbouring pixel points. Binary cubic spline interpolation, on the other hand, constructs a bicubic interpolation function to obtain the light intensity of the subpixel based on the surrounding light intensity matrix. Although this interpolation function ensures smooth first-order derivatives and continuous second-order derivatives. However, the calculation process is complex and time consuming. Based on the fact that the light intensity of the image used in this paper is approximately monotonically varying near the blurred region. So the bilinear interpolation has been able to meet the needs of this paper.

**Fig 9:**
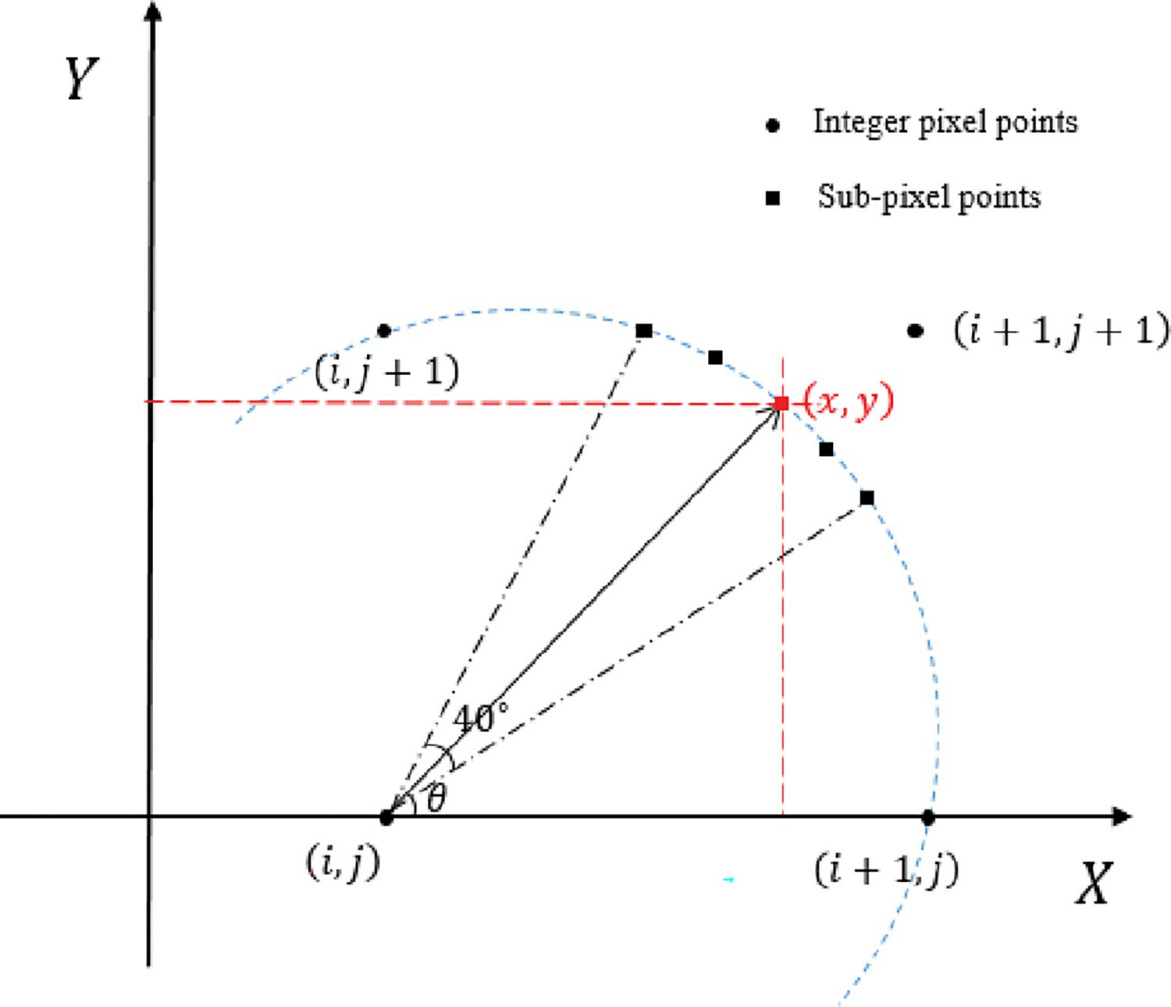
Sub-pixel coordinates and their final light intensity gradient finding range.

As shown in **Fig** 9, the coordinates of the point to be interpolated is (*x*,*y*), and the coordinates of the four adjacent whole-pixel points are: (*i*,*j*), (*i*+1,*j*), (*i*,*j*+1), (*i*+1,*j*+1). The corresponding light intensities are: *I*(*i*,*j*), *I*(*i*+1,*j*), *I*(*i*,*j*+1), *I*(*i*+1,*j*+1). Then the light intensity *I*(*x*,*y*) of any non-integer pixel (*x*,*y*) obtained by bilinear interpolation is:

$$\begin{array}{l} I(x,y)=I(i,j)\cdot (i+1-y)\cdot (j+1-x)+I(i+1,j)\cdot (y-i)\cdot (j+1-x)+\\ I(i,j+1)\cdot (i+1-y)\cdot (x-j)+I(i+1,j+1)\cdot (y-i)\cdot (x-j) \end{array}$$ (22)

*c) Determination of the final light intensity gradient direction*. The direction of the light intensity gradient will directly affect the accuracy of the blur width calculation. Therefore, it is crucial to measure the exact gradient direction. An initial value of the gradient direction *θ* has been obtained from the Sobel operator. However, in order to prevent the influence of factors such as image noise and inherent defects of the object under test on the results of the Sobel operator. In this paper, a limited range of angles will be used. The final more accurate light intensity gradient direction of the image is obtained by means of an angular search method.

The process is as follows: first set the coordinates of the pixel point to be measured as (*i*,*j*) and its light intensity as *I*(*i*,*j*), as shown in **Fig** 9; in the second step use the Sobel operator to find the gradient direction *θ*; then read the light intensity value of the sub-pixel every 1 pixel on the arc with (*i*,*j*) as the centre, the radius as 1 pixel and the angle as [*θ*−20°,*θ*+20°]; finally the direction with the largest absolute value of the difference between the light intensity value of these sub-pixels and *I*(*i*,*j*) is the final light intensity gradient direction L.

**Fig 10:**
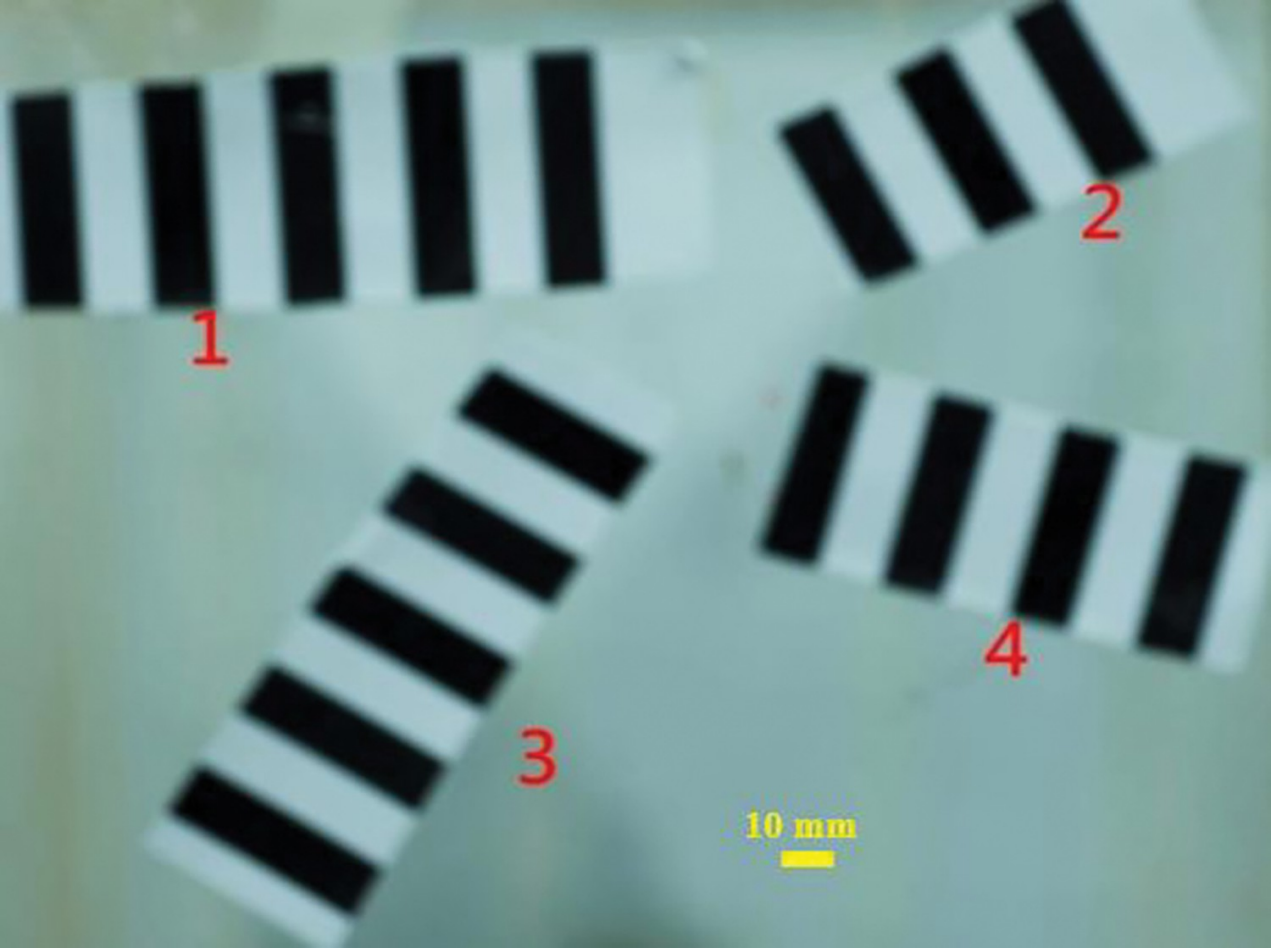
Out-of-focus picture of striped features at four angles.

*d) Validation experiments*. In **Fig** 10, an underfocused image of a plane with four sets of stripes with distinct angles is displayed. Their maximum gradient direction is calculated by the method described in this section. The results are shown in **Table** 1, where it can be seen that the calculated maximum gradient directions of the four different sets of angular stripes do not differ much from the original stripes. This indicates that the method proposed in this section for calculating the direction of the light intensity gradient works well in the actual picture.

**Table 1:** Gradient directions for different angular stripes and results of calculated maximum gradient directions.

Stripe number	Gradient direction (°)	Calculating gradient direction (°)
1	3.00	2.95
2	28.00	27.91
3	62.00	62.01
4	-14.00	-14.08

For the fuzzy circle radius *R*, this article directly uses the theoretical **Eq** 12 of light intensity distribution to fit the relative light intensity distribution curve in **Fig** 3. The light intensity *W* of the white stripe, the light intensity *B* of the black stripe, and the radius *R* of the dispersion circle can be obtained.

#### 2.3.5 Coordinate conversions

**Fig 11:**
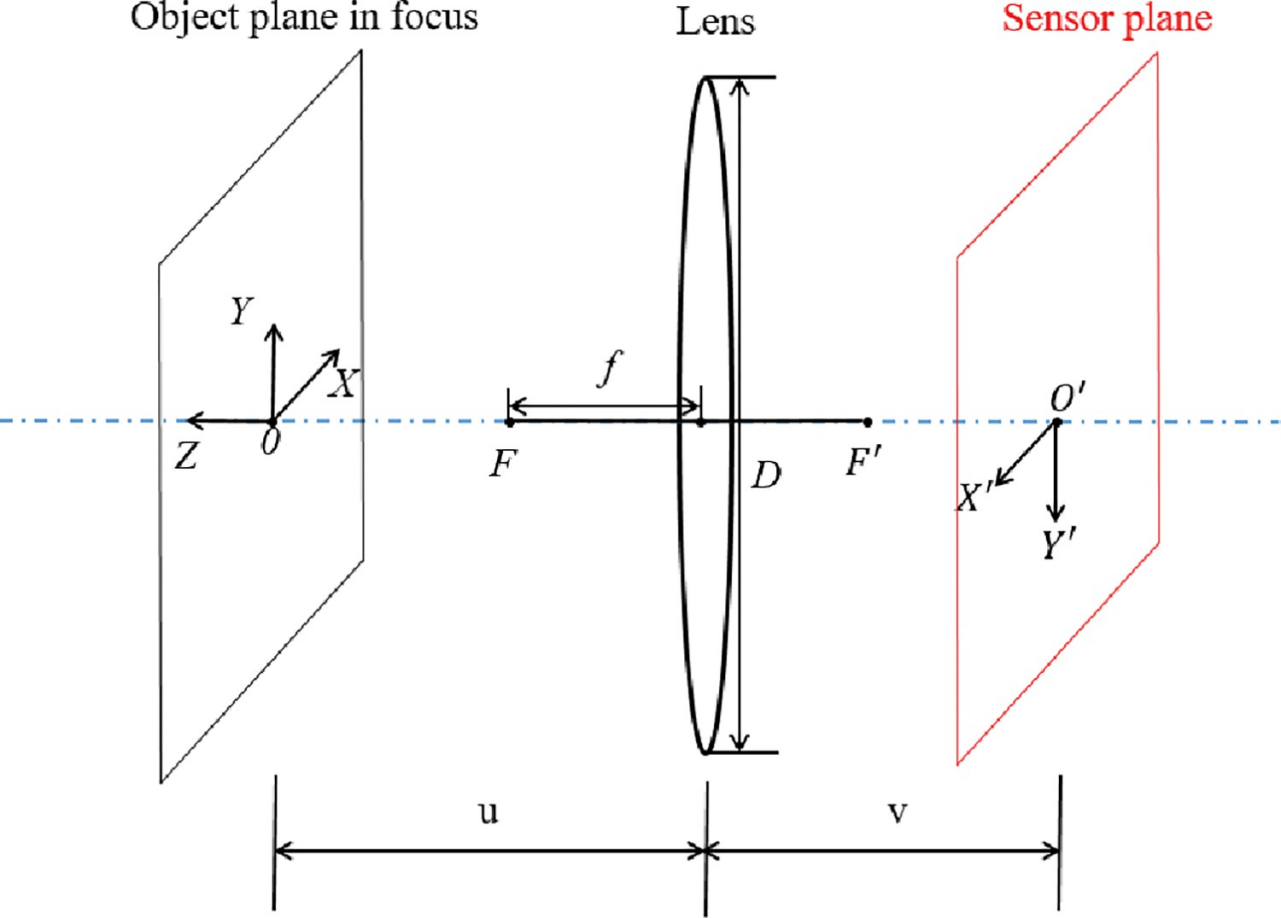
Coordinate systems on the object and image planes when focused.

In the previous work, information about the object in the depth direction was obtained by measuring the blurred width. The measurement of the in-plane coordinates of the object is given below. In **Fig** 11, the object plane when the object is focused is shown on the left. On the right is the plane where the sensor is located and also the image plane when focused. *u* and *v* are the object and image distances respectively at this point and *f* is the focal length. The world coordinate system *OXYZ* falls on the object plane. The coordinate origin *o* lies at the intersection of the optical axis and the object plane, *x* and *y* are the axes in the object plane, and the axis *z* is perpendicular to the object plane. Any point on an object can be expressed in coordinates (*x*,*y*,*z*) in mm. The *z* coordinate is the out-of-focus distance *d* when over focus and the *z* coordinate is the out-of-focus distance *e* when under focus. The image coordinate system *O*′*X*′*Y*′ lies in the image plane and can be used to represent the position of an object on a photograph in pixels. The origin *O*′(0,0) of the image coordinate system is the image of the origin *O*(0,0,0) of the world coordinate system.

The magnification of the camera is known from the optical path of the camera image as:

$$M^{\prime }=\frac{v}{u}$$ (23)

The in-plane coordinates read from the photo are image coordinates, in pixels. However, the coordinates located on the object are world coordinates, in millimeters. In order to connect image coordinates with world coordinates, it is necessary to know the conversion relationship between them. Since the resolution of the Nikon D800 camera is 7360*pixels*×4912*pixels*, the sensor size is 35.9*mm*×24*mm*. So, the pixel width corresponding to 1 mm width in the horizontal direction of the sensor is 205.01 pixels, and the pixel width corresponding to 1 mm width in the vertical direction is 204.67 pixels. The slight difference in pixel width per unit distance between the horizontal and vertical directions is caused by the manufacturing process of the sensor. For simplicity, the pixel widths corresponding to 1mm width on the sensor are taken as their average values *p* = 204.87*pixels*/*mm*. The magnification formula of the camera can be rewritten as:

$$M=p\frac{v}{u}$$ (24)

The camera imaging optical path shows that the coordinates of the image of a point (*x*,*y*,0) on the object plane in the image plane are:

$$\left\{ \begin{array}{l} x^{\prime }=p\frac{v}{u}x\\ y^{\prime }=p\frac{v}{u}y \end{array}\right.$$ (25)

A point (*x*,*y*,*z*) on the object deviates from the object plane by a distance *z*, so that the point forms a fuzzy circle of radius *R* in the image plane, and the image coordinates of the centre of the fuzzy circle are:

$$\left\{ \begin{array}{l} x^{\prime }=\frac{pv}{u+z}x\\ y^{\prime }=\frac{pv}{u+z}y \end{array}\right.$$ (26)

Thus, if the image coordinates (*x*′,*y*′) of a point on the photograph are known, and also the out-of-focus distance *z* of that point, the world coordinates of that point are:

$$\left\{ \begin{array}{l} x=\frac{u+z}{pv}x^{\prime }\\ y=\frac{u+z}{pv}y^{\prime }\\ z=z \end{array}\right.$$ (27)

The previous section describes methods for measuring the in-plane coordinates of an object. It is worth noting that currently object feature edge detection is often achieved by edge detection operators, such as the sobel operator, the prewitt operator and the canny operator. These methods require a clearer picture, otherwise the detection will not be satisfactory, and their measurements are only available as whole pixel results. These drawbacks make it difficult to apply them to mechanical experiments for strain measurement. The method proposed in this paper is effective for fuzzy images and its accuracy breaks through the whole-pixel level, which makes it more promising for strain measurement.

### 2.4 Measurements of real objects

In order to further verify the validity and feasibility of the method proposed in this paper for measuring the three-dimensional shape of an object from a single photograph, experiments were carried out to measure two objects in different pendulum positions.

**Fig 12:**
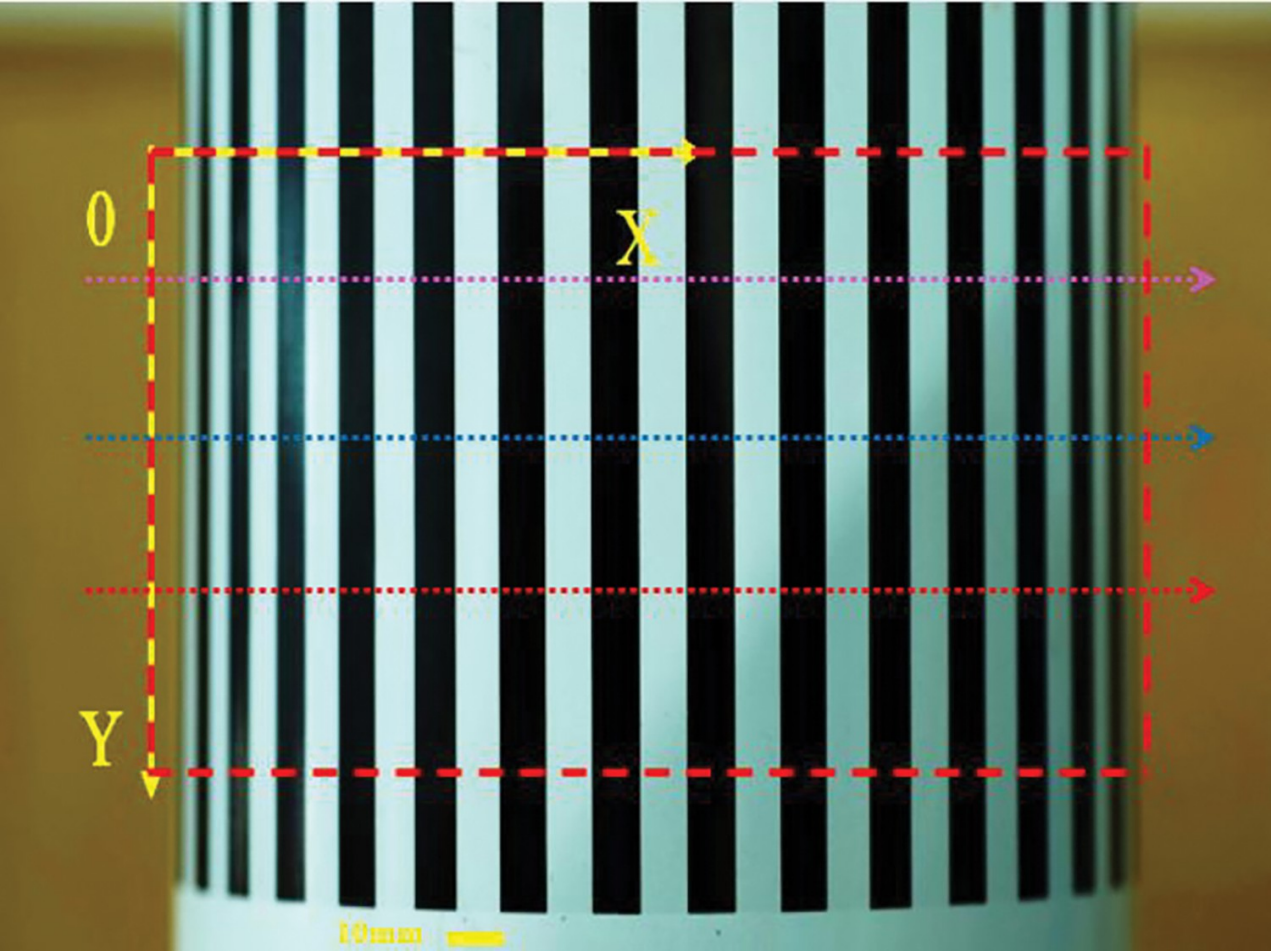
Cylindrical object placed perpendicular to the ground.

Firstly, the 3D shape of a cylinder with a radius of 63.50 mm placed perpendicular to the ground in an overfocused state was measured. The camera parameters at the time of the measurement were: *F* = 4.5, ISO = 100, exposure time of 0.25 s and object distance at focus *u* = 326.49 mm, *v* = 73.51 mm. The photograph was taken with the cylinder in front of the camera and both placed perpendicular to the ground. The photograph taken during the measurement is shown in **Fig** 12.

**Fig 13:**
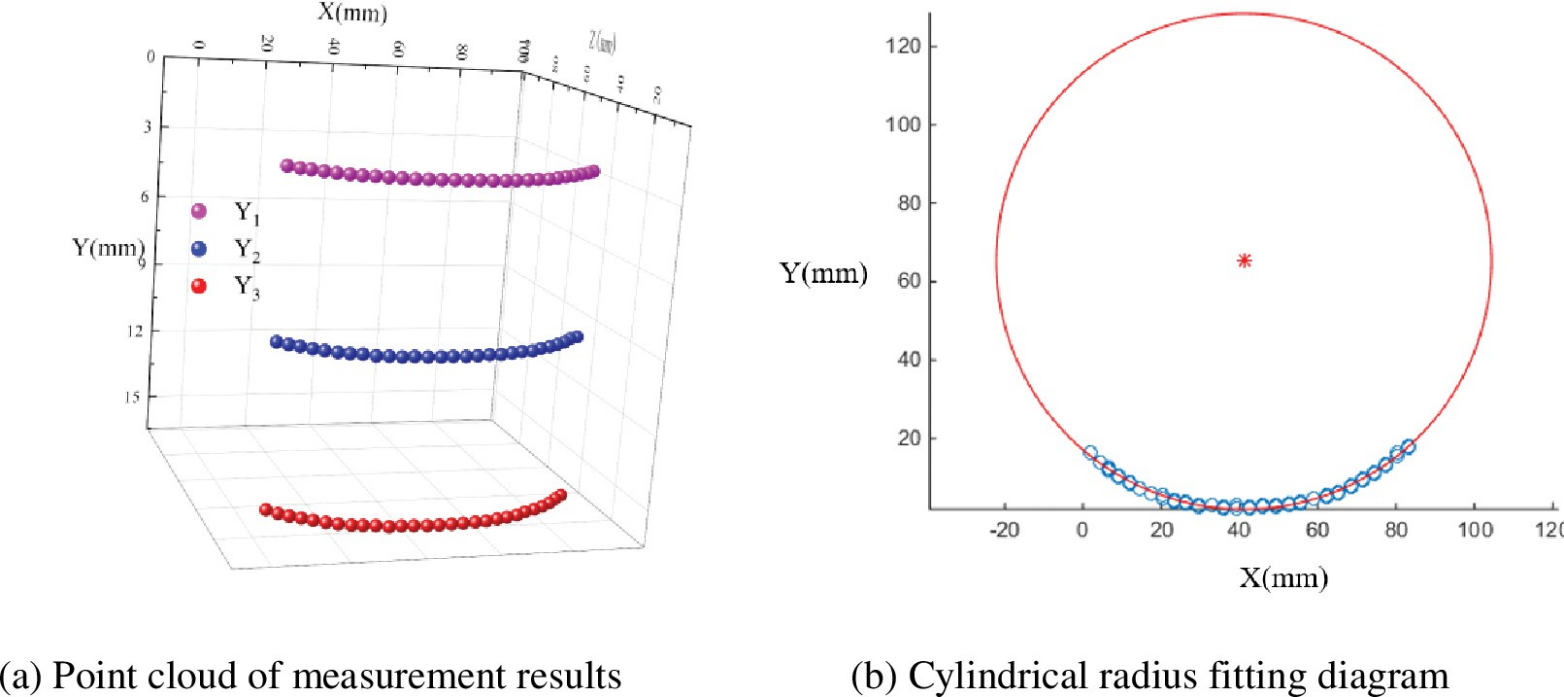
Vertical cylindrical measurement results. (a) Point cloud of measurement results. (b) Cylindrical radius fitting diagram.

In **Fig** 12, the surface of the cylinder has black and white stripes in the vertical direction. The red coloured box in the diagram shows the measurement area. As can be seen from the diagram, the light intensity within the measurement area is uneven due to reflections and shadows. For the measurement, the depth information was calculated for each of the three selected pixel points in the *Y* direction. The point cloud of the measurement results for the cylinder were shown in **Fig** 13(A). As both the cylinder and the camera are placed perpendicular to the ground, the out-of-focus distance along the vertical direction *Y* is the same on the cylinder. Based on the calculations in **Fig** 13(A), the depth measurements of the cylinder along the horizontal direction *X* are given in **Fig** 13(B). The solid line in the figure shows the true shape of the cylinder. It can be seen from the figure that the measurements on the same stripe boundary have good overlap and that they match the true shape of the cylinder well. The measured radius of the cylinder is 63.2473 mm. The error between the measured value and the true value is 0.2527 mm. The relative error is 0.0039. Both the error and the relative error are very small, so the method in this paper is very accurate.

**Fig 14:**
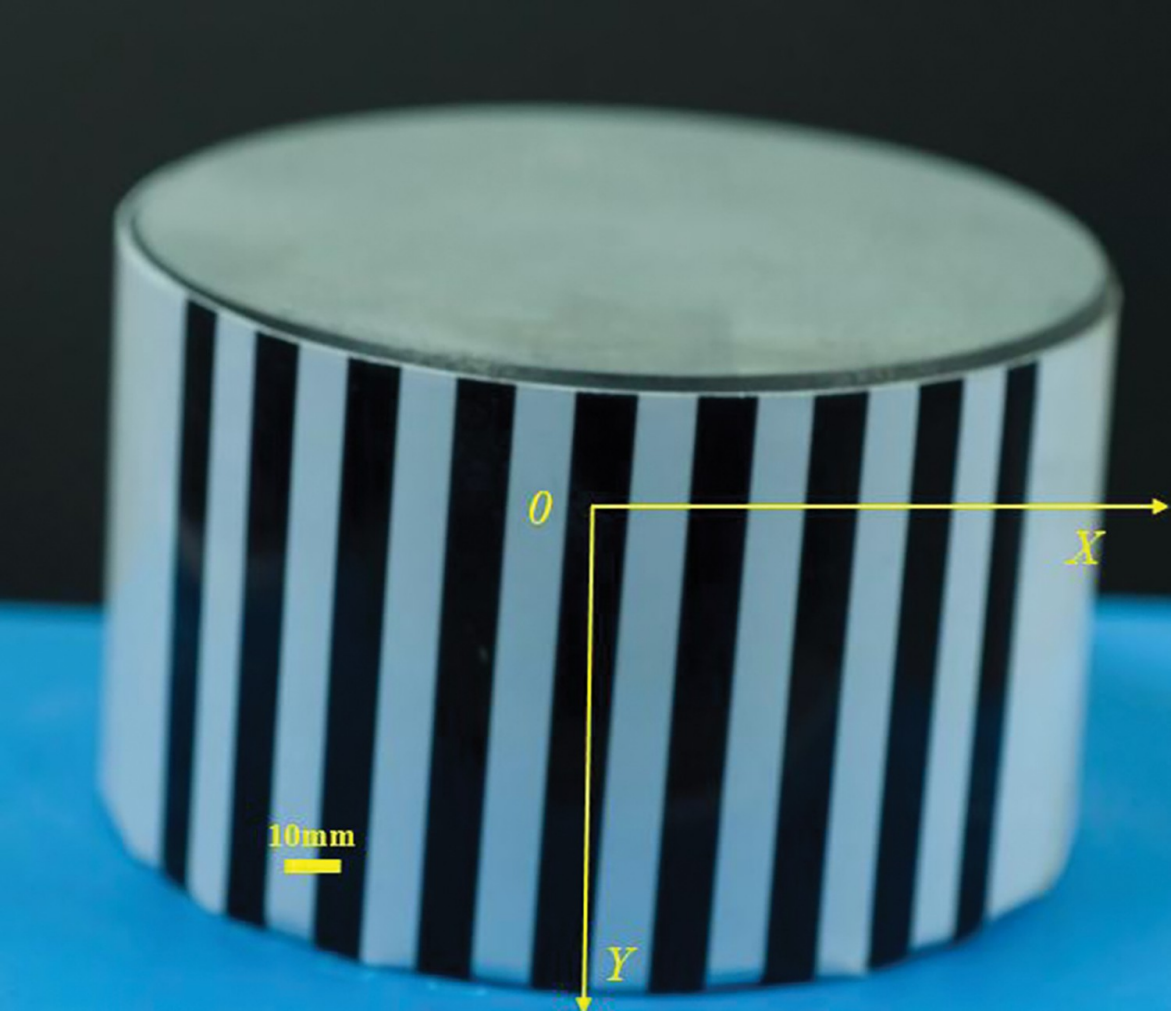
Cylinder placed at an angle (overfocused state).

In the experiment described earlier, the object under test and the camera were placed perpendicular to the ground. To test the practicality of this measurement method, a cylinder placed at an angle is measured below, as shown in **Fig** 14. The diameter of the cylinder is 88 mm (measured with a vernier caliper). The camera parameters used for the measurement are: *F* = 7.1, ISO = 250, exposure time of 1/3 s, object distance at sharp focus *u* = 430.28 mm, *v* = 69.72 mm.

**Fig 15:**
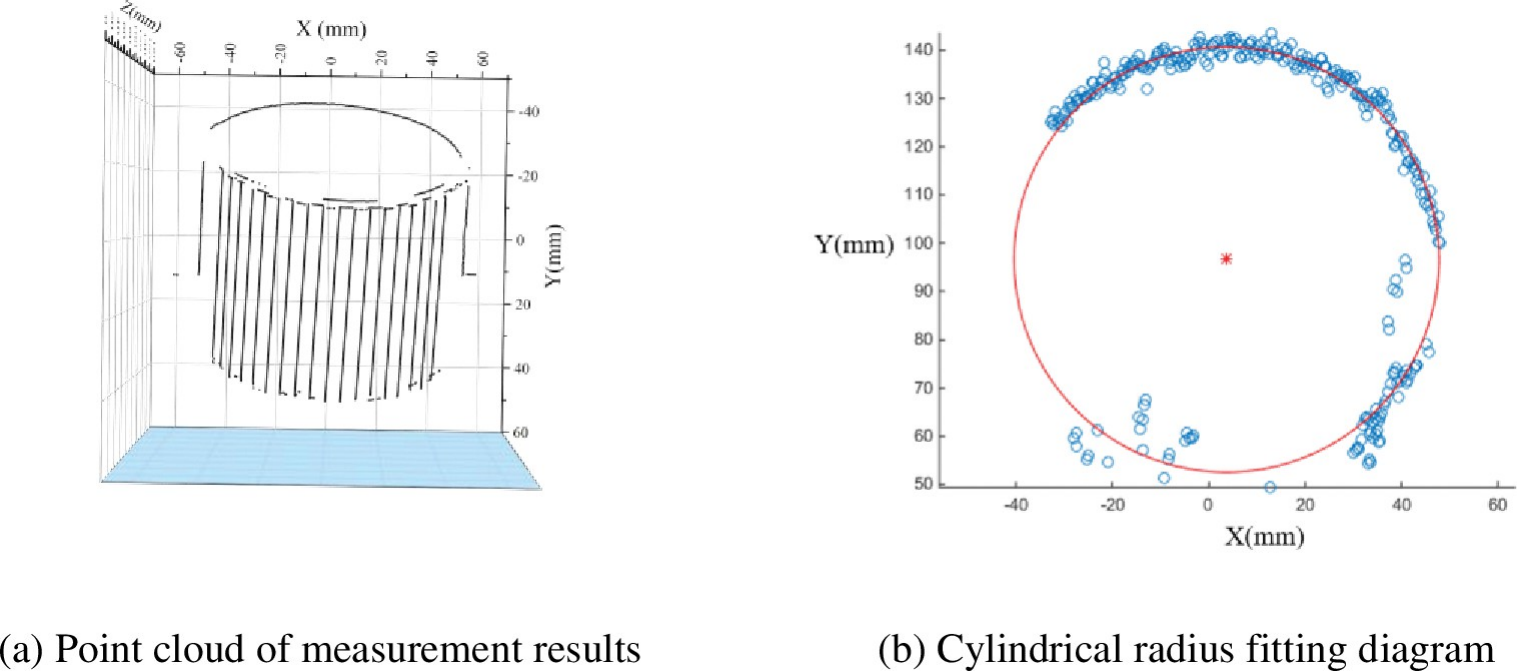
Measurement results of a column placed at an angle.

The results of the measurements are shown in **Fig** 15. Fitting the measurements to the cylindrical equation gives a fitted diameter of 88.226 mm. The error between the measured value and the true value is 0.226 mm. The relative error is 0.0026. Both the error and the relative error are very small, so the method in this paper is very accurate.

The method proposed in this paper restores the three-dimensional shape of an object based on the blurred areas near the stripe boundaries. So the surface of the object must have some feature boundaries, otherwise it will not be measured. The cylinder shown in **Fig** 14 has no features on the upper surface, so the 3D information inside the upper surface cannot be measured. However, because the colour of the object and the background are often different, these differences in colour allow the object to be approximated as a striped feature on the boundary. When blurred out of focus, the photograph forms blurred bands on the boundaries of the object. Using these blurred bands, the method proposed in this paper is able to measure the three-dimensional information on the boundaries of the object. The results of the measurements at the boundary of the upper surface of the cylinder have been presented in **Fig** 15. The measurements show that the proposed method is accurate and effective at the boundaries of the object.

## 3 NEPE solid propellant strain measurement

Due to their plentiful raw material sources, simple manufacture, extensive variety, and wide range of applications, polymer materials have drawn considerable attention from academics both locally and globally. Polymer materials are essential for human production and daily living in general. NEPE(Nitrate Ester Plasticized Polyether) solid propellant has been used in a variety of high-tech industries since it is a substance with excellent performance and high functionality. It frequently has strong ductility because of its structural makeup and material composition, so it is important to research its mechanical properties under large deformation. Traditional strain measurement methods have a tough time obtaining the strain at different regions of the specimen with accuracy because of the frequently non-uniform deformation and even necking occurrence during the stretching process. And the approach described in this article can successfully address this issue. The specific experimental procedure and a comparison of the measurement results with conventional approaches are provided below.

### 3.1 Composition of the material

The NEPE solid propellant was used in the test, it was prepared by reference [32] with the following composition and content: AP (ammonium perchlorate, 10 wt.%, 120 μm) as an oxidizer, Al (aluminum, 18 wt.%, 45 μm) powders as a metal fuel, HMX (cyclotetramethylene tetranitramine, 42 wt.%, 80 μm) as a high-energy explosive, NG (Nitroglycerin, 21.5 wt.%) as an energetic plasticizer, PEG (Polyethylene glycol, 7 wt.%) as a binder and N-100 (1,3,5- tris-(6-isocyanatohexyl) biuret, 1 wt.%) as a curing agent. Furthermore, C2 (dimethydiphenylurea, 0.5 wt.%) was added as a chemical stabilizer for the NG. The propellants were cured at 50°C for 7 days.

From the above material composition of NEPE solid propellant, it can be seen that NEPE solid rocket propellant is a particle-filled composite material, with a matrix of binder and plasticiser, and fillers such as oxidiser particles, metal particles, etc.The NEPE propellant matrix exhibits viscoelasticity, and the overall mechanical properties are also viscoelastic; the viscoelastic mechanical properties have the elasticity of a solid as well as the viscous nature of a liquid, and are dependent on time and temperature. At the same time, NEPE solid rocket propellant as a particle-filled composite material, when subjected to external loads, the adhesive surface between the filled particles and the matrix generates local stress; when the local stress reaches a certain level, there will be an interfacial debonding phenomenon between the filled particles and the matrix. If the external load continues to increase, the debonding phenomenon will be intensified, and micropores appear between the filler particles and the matrix. The micropores inside the propellant continue to expand, resulting in microcracks. Continued expansion of the microcracks induces macroscopic cracks and eventually leads to fracture. During the entire loading process, the internal structure of the propellant produces irreversible damage; the internal structure damage of the propellant extends from micro to macro, and its overall macroscopic mechanical properties deteriorate. Therefore, the mechanical properties of NEPE solid rocket propellant are viscoelastic on the whole, and will deteriorate due to damage after load bearing, showing obvious nonlinear viscoelasticity. It is very important to be able to accurately measure its viscoelastic damage, the viscoelastic damage properties of NEPE solid propellants would be tested under uniaxial tension.

### 3.2 Sample preparation

**Fig 16:**
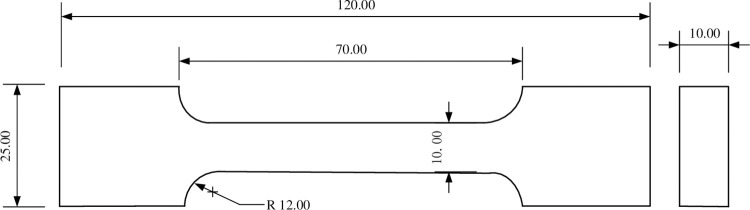
Schematic diagram of the NEPE propellant uniaxial tensile test sample (mm).

As shown in **Fig** 16, the specimen has a dog-bone shape. The size of the cross-section of the specimens is 10 mm ×10 mm. The specimens were stamped out of a bigger plate matrix (10 mm thick). In the experiment, the black ink was sprayed on the surface of the test piece several times with a spray to form a dense random artificial speckle.

### 3.3 Experimental device and strain measurement methods

**Fig 17:**
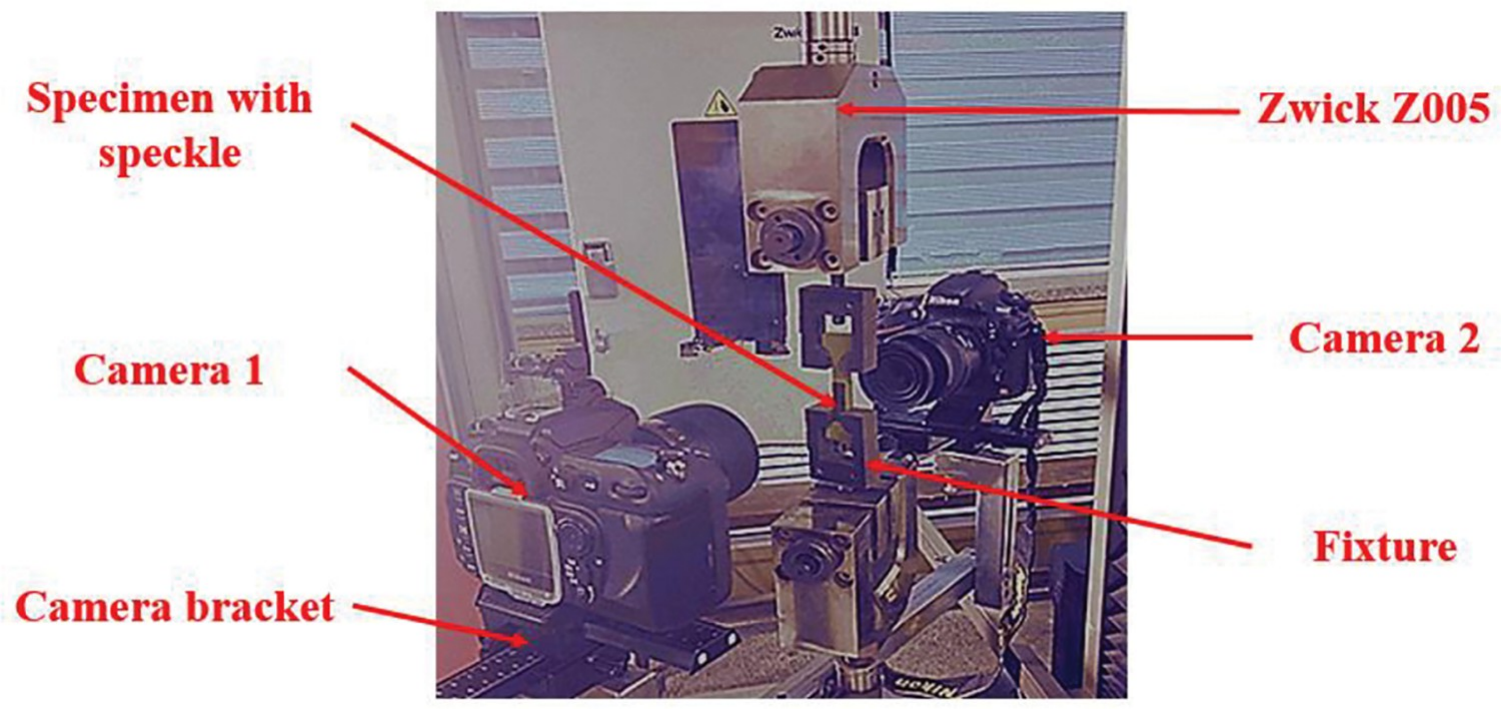
Experimental instruments and devices.

The Z005 universal testing machine is the experimental tool used in the uniaxial tensile test, its load cell capacity is 5 KN. The out-plane strain of the specimen can be measured using the picture out-of-focus method described in the previous section. And the in-plane in-plane strain of the specimen can be measured using the Digital Image Correlation (DIC) method. Combining the two optical measurement methods allows for the measurement of strain in three directions at each point on the specimen surface. Both methods are implemented by writing a programme. Ideally, if the deformation of the specimen in the thickness direction during stretching is symmetrical, only a camera is needed to record a picture of the stretching process. However, in practical experiments this is difficult to guarantee due to errors in the machining of the specimen and errors in the fixture hold-up. Therefore, in this experiment, two cameras were used to simultaneously take photographs of the front and back of the specimen during tensioning. Shrinkage deformations of the specimen in the thickness (out-of-face) direction were calculated for the front and back faces, respectively. The total deformation of the specimen in the thickness direction was obtained so that an accurate strain in the thickness direction could be obtained. The circular arc section of the bone specimen through the groove type fixture, as illustrated in **Fig** 17, holds the specimen on the testing apparatus. The test piece’s front has a Nikon D800 camera as camera 1. Nikon AF micro Nikkor 60 mm 1:2.8 D is the lens. The camera has an aperture of F = 2.8, a shutter speed of 1/40 s, an iso = 250, and a 1:1 magnification. The Nikon D200 camera is located on the back of the test item. The lens is an 85 mm 1:3.5 G Nikon AF-S micro Nikkor. The camera has an aperture of F = 3.5, a shutter speed of 1/30 s, an iso = 250, and a 1:1 magnification. Through the camera bracket, the two cameras are mounted on the testing device, and their positions are carefully adjusted using the micro movable pan tilt on the bracket to place their fields of view in an appropriate location. The experiment was conducted at 20°C ambient temperature. The uniaxial tensile tests of NEPE solid propellant were conducted in a closed laboratory where the laboratory temperature was controlled by two air conditioners. The two air conditioners were kept on before and during the test. Displacement loading served as the loading control strategy. Three different tensile rates 1 mm/min, 100 mm/min, and 500 mm/min were employed. The collet speed of the testing machine is referred to as the tensile rate in this context. The frame rate used to capture the image was 1fps for tests at 1 mm/min and 100 mm/min displacement rates, and 0.5 fps for tests at 500 mm/min displacement rate.

#### 3.3.1 DIC method (in plain strain)

**Fig 18:**
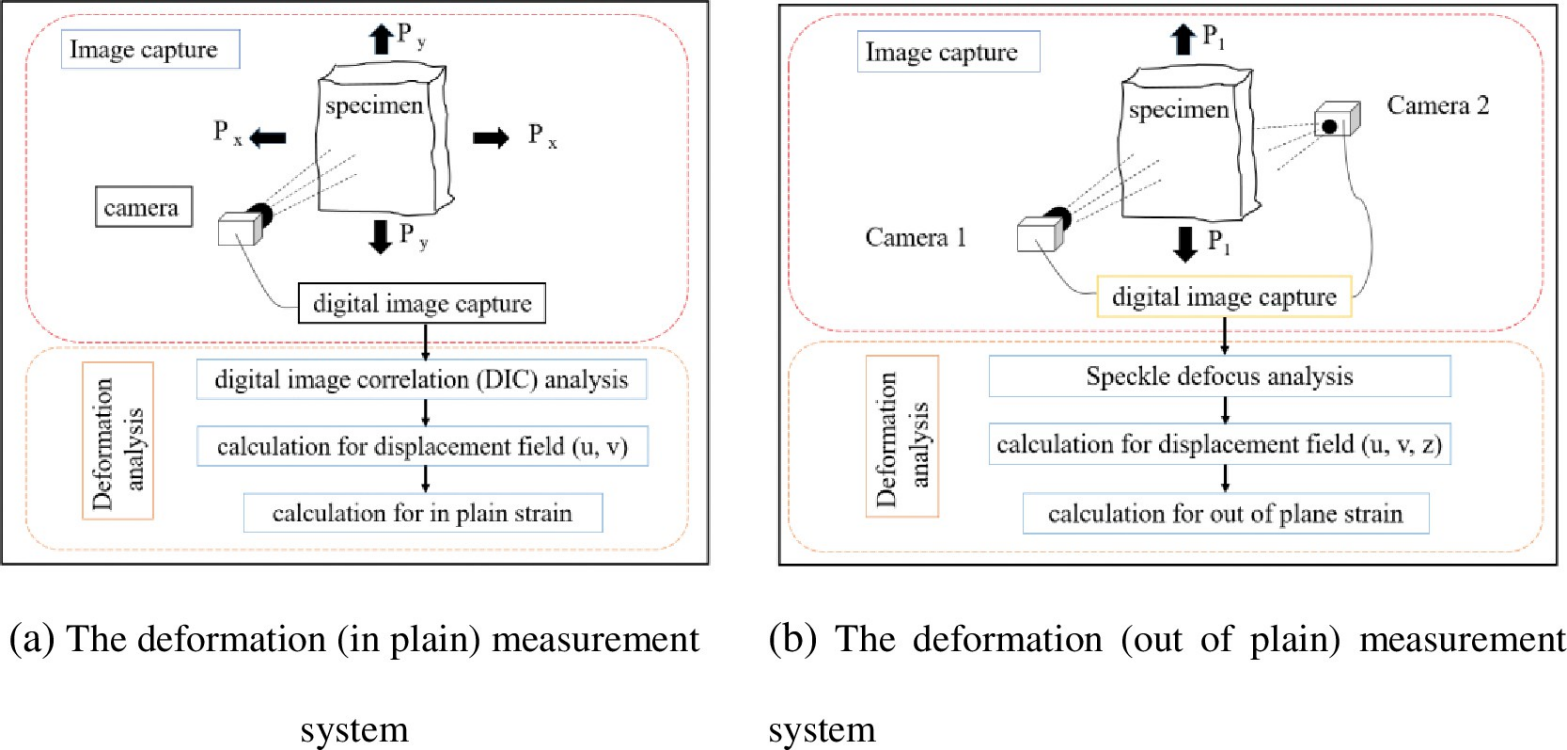
Experimental instruments and measurement system. **(a)** The deformation (in plain) measurement system. **(b)** The deformation (out of plain) measurement system.

The Digital Image Correlation (DIC) method was adopt to measure in plain displacement of this experiment. The principle of DIC method was given in reference [27, 28]. The principal features and procedures of displacement (in plain) measurement system were developed in this paper as shown in **Fig** 18(A).

#### 3.3.2 Defocus method (out of plain strain)

Out of plain deformation were measured by using defocused images of objects with artificial speckle features. The principle of speckle defocus method was given in section 2. The principal features and procedures of displacement measurement system were developed in this paper, as shown in **Fig** 18(B).

To sum up, during strain measurement, the in-plane displacement field in the measurement area is first measured by DIC method. According to the defocus method as shown in section 2, the defocusing distance in the measurement area can be obtained. The displacement field obtained by DIC method, the relationship between the position of a point on the object before and after deformation is obtained. According to this relationship, the defocusing distance after deformation of a point on the object is subtracted from the defocusing distance before deformation to obtain the out of plane displacement in the measurement area. Finally, according to the out of plane displacement and the thickness of the specimen before deformation, the out of plane strain can be obtained.

## 4 Results and discussion

### 4.1 In plain strain measurement

**Fig 19:**
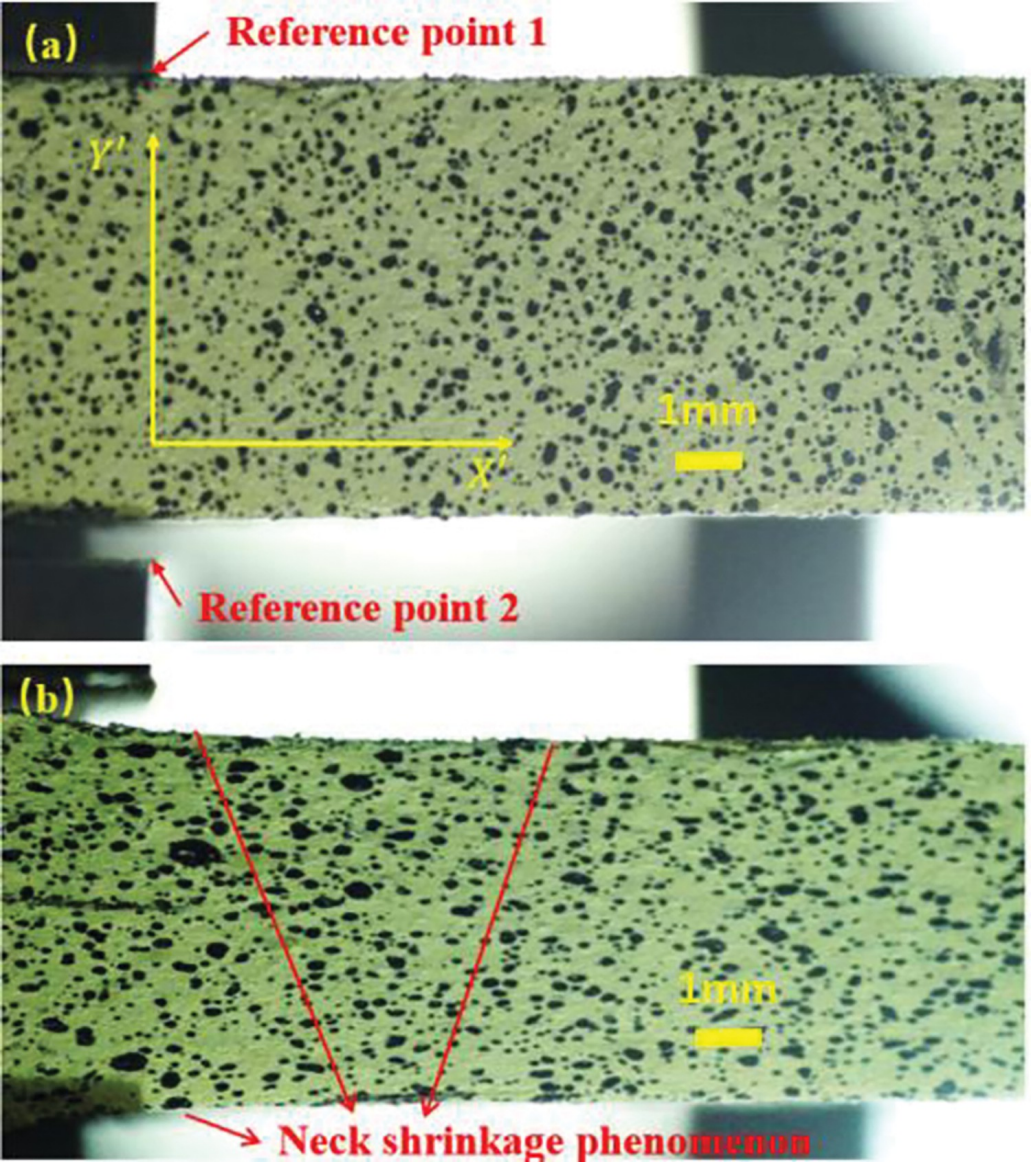
Front view images of NEPE tensile specimen at different time. (a) image at the time of the initialization t = 0 s (b) image at the time before destruction t = 25s, respectively.

As the specimen was continuously stretched, its deformation gradually increased, and the picture changed from clear to fuzzy. **Fig** 19 showed two images of NEPE specimen recorded at different stages of deformation. The deformation experienced by NEPE specimens during uniaxial tensile testing could be roughly divided into three stages. In the first stage, where the strain was small, the material showed an elastic deformation, which meant that when the applied tensile force was released, the material returns to its initial shape. The relationship between stress and strain follows Hooke’s law, which stated that stress was directly proportional to strain. In the second stage, as the strain increases, the material may exceed its elastic limit and enter the plastic deformation stage. During this stage, the material would continue to deform, but would not completely return to its initial shape when the tensile force was released. The material would deform permanently. During the plastic deformation stage, the material will experience necking. This was due to the uneven distribution of stresses within the material at this point, resulting in certain areas being subjected to greater stresses, further triggering local deformation. In the third stage, fracture eventually occured when the material exceeded its load-bearing capacity. The incremental displacement fields could be obtained by DIC analysis, and the total displacement field could be obtained at different deformation stage. In **Fig** 19(A), the two points are the corners of the fixture. There are also these two corners of the fixture in the photos on the back of the test piece. According to the positions of these two corners in their respective photos, the points with the same coordinates (*x*,*y*) on the front and back photos can be matched. The world coordinate system of the measured object is (*x*,*y*), which is the in-plane component of the world coordinate system.

**Fig 20:**
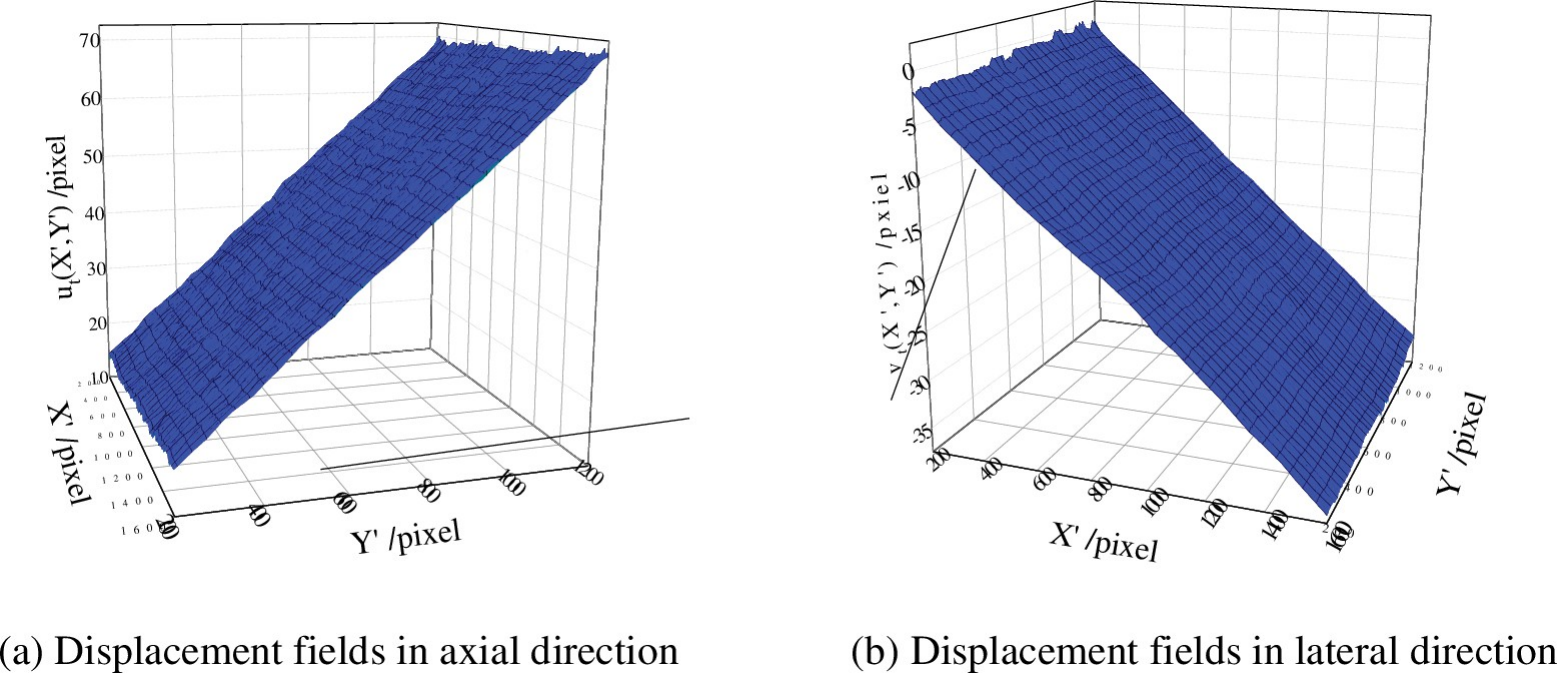
Displacement fields at the time during stretching. (a) Displacement fields in axial direction. (b) Displacement fields in lateral direction.

**Fig** 20(A) and 20(B) respectively showed the displacement of each point before the relative deformation u_*t*_(*X*′,*Y*′) of the front face of the specimen in the tensile direction and the displacement of each point before the transverse relative deformation *v*_*t*_(*X*′,*Y*′) at t = 12 s. It could be seen from the **Fig** 20 that the specimen had obvious necking, and the deformation along the tensile (axial) direction and width direction was relatively uniform.

According to the in-plane displacement field, the in plane strain of each point on the surface of the specimen could be written as follows:

$$\left\{ \begin{array}{l} \varepsilon_{ex}=\frac{\partial u_{t}(X',Y')}{\partial X'}\\ \varepsilon_{ey}=\frac{\partial v_{t}(X',Y')}{\partial Y'}\\ \gamma_{xy}=\frac{\partial u_{t}(X',Y')}{\partial Y'}+\frac{\partial v_{t}(X',Y')}{\partial X'} \end{array}\right.$$ (28)

Where *ε*_ex_ is the horizontal engineering strain, *ε*_*ey*_ is tensile engineering strain in the tensile direction and *γ*_*xy*_ is the shear strain.

**Fig 21:**
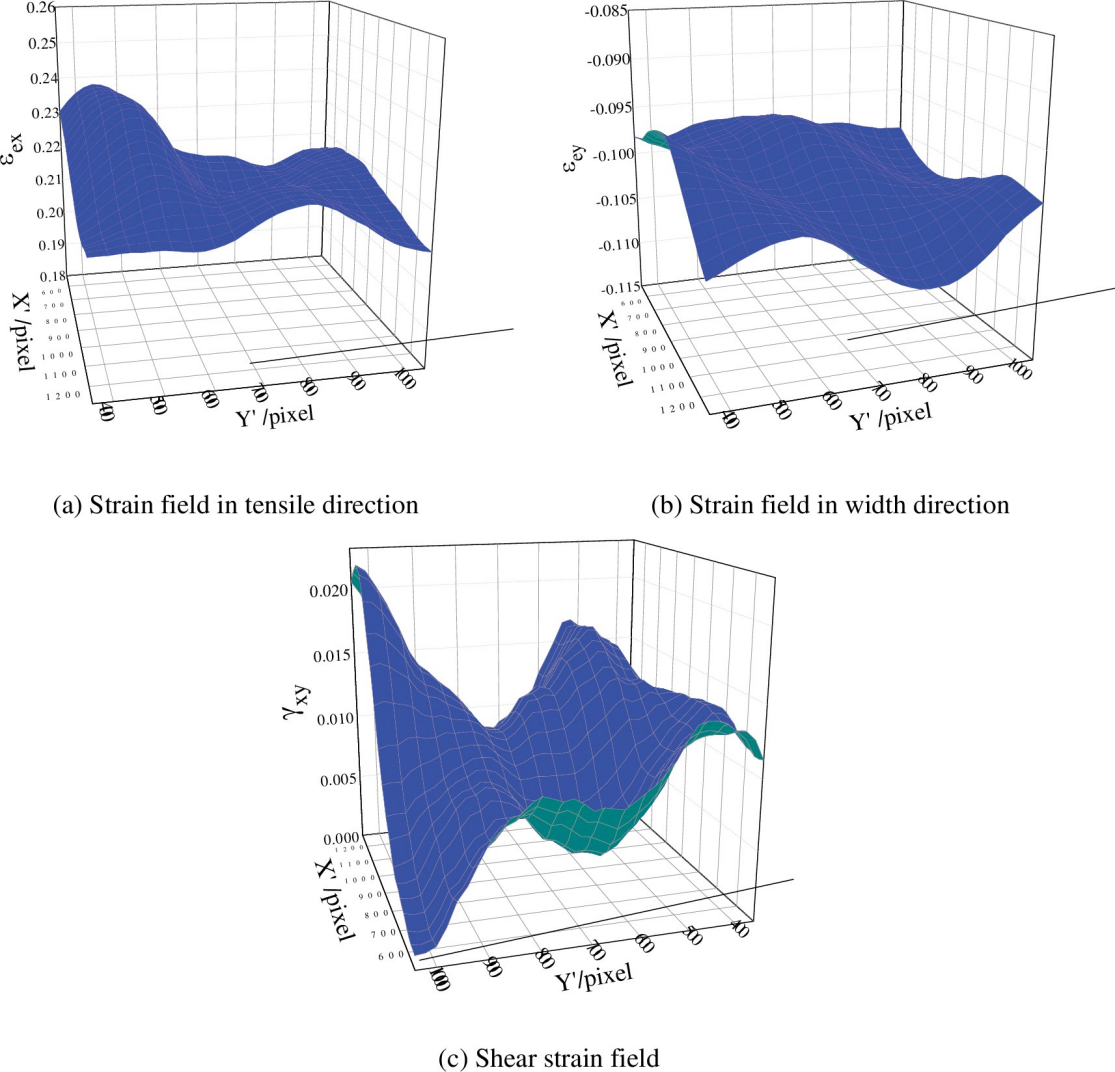
In plane strain field on the front of specimen (t = 12 s). (a) Strain field in tensile direction. (b) Strain field in width direction. (c) Shear strain field.

The engineering strain field could be obtained from **Eq** 28, as shown in **Fig** 21. A clear necking phenomenon could be seen in the strain field diagram.

The shear strain outside of the necking zone should be zero because there was only one axial tensile force applied to the specimen during the experiment. **Fig** 21(C) revealed, however, that the shear strain estimated using the image correlation method was not zero per cent. Because the test piece’s tensile direction somewhat deviates from the horizontal direction, as seen in the experiment photographs (**Fig** 19), the shear strain in the in-plane strain calculation result was not zero.

Normal strain and shear strain must be converted into main strain in order to remove the impact of picture tilt on the findings of strain. The following is the conversion formula:

$$\left\{ \begin{array}{l} \varepsilon_{e1}=(\varepsilon_{ey}+\varepsilon_{ex})/2+\sqrt{{\left(\varepsilon_{ey}-\varepsilon_{ex}\right)}^{2}+\gamma_{xy}^{2}}/2\\ \varepsilon_{e2}=(\varepsilon_{ey}+\varepsilon_{ex})/2-\sqrt{{\left(\varepsilon_{ey}-\varepsilon_{ex}\right)}^{2}+\gamma_{xy}^{2}}/2 \end{array}\right.$$ (29)

Where *ε*_*e*1_ is the axial engineering principal strain and *ε*_*e*2_ is the engineering principal strain in width direction.

**Fig** 21 showed that the value of *ε*_*ey*_−*ε*_*ex*_ was significantly higher than the value of *Y*_*xy*_. As a result, **Eq** 29 indicates that the value of *γ*_*xy*_ had little impact on the primary strain. The average value of all strains in the measurement area could be used to determine the in-plane strain of the specimen surface at this point because **Fig** 21 also shows that the strain in the tensile direction and the strain in the width direction vary within a small range throughout the entire measurement area.

### 4.2 Out of plane strain measurement

Based on a single shot, the object’s three-dimensional shape was measured using the defocus technique. The *X*′ and *Y*′ pixel coordinates on the image before distortion are shown in the **Fig** 19(A). The sample snapshot taken at time t can be used to determine the defocus distance *Z*_t_*(*X*’_*t*_,*Y*’*t*) of a specific place (*X*’_*t*_,*Y*’*t*) on the image at that time. The position (*X*’_*t*_−*u*_*t*_,*Y*’_*t*_−*v*_*t*_) of a point (*X*’_*t*_,*Y*’*t*) on the specimen at time t on the image before deformation (t = 0 s) could be determined according to the displacement field *u*_*t*_(*X*’,*Y*’) and *v*_*t*_(*X*’,*Y*’) at that time, according to the DIC method. That is, a point on the specimen after deformation could be traced back to its position before deformation. Therefore, when the defocus distance of a point on the test piece at time t was converted to the coordinate system before deformation, the expression of the converted defocus distance could be written as:

$$Z_{t}(X',Y')=Z_{t}(X{'}_{t}-u_{t},Y{'}_{t}-v_{t})=Z_{t}{}^{*}(X{'}_{t},Y{'}_{t})$$ (30)

Where: *Z*_*t*_ is the defocus distance distribution field after conversion, *Z*_t_* is the defocus distance field before conversion. In this paper, the defocusing distance of the front photo of the specimen is recorded as *Z*_*t*_^*f*^(*X*′_*t*_,*Y*′_*t*_), and the defocusing distance of the back photo is recorded as *Z*_*t*_^*b*^(*X*′_*t*_,*Y*′_*t*_).

The defocusing distance following deformation at any time was evenly traced back to the coordinate system prior to deformation, according to **Eq** 30. The displacement perpendicular to the optical direction may be calculated by measuring the difference between the defocusing distance before and after deformation at the same spot on the test specimen, which could be stated by **Eq** 31 as follows:

$$\left\{ \begin{array}{l} w_{t}^{f}(X',Y')=Z_{t}^{f}(X',Y')-Z_{0}^{f}(X',Y')\\ w_{t}^{b}(X',Y')=Z_{t}^{b}(X',Y')-Z_{0}^{b}(X',Y') \end{array}\right.$$ (31)

**Fig 22:**
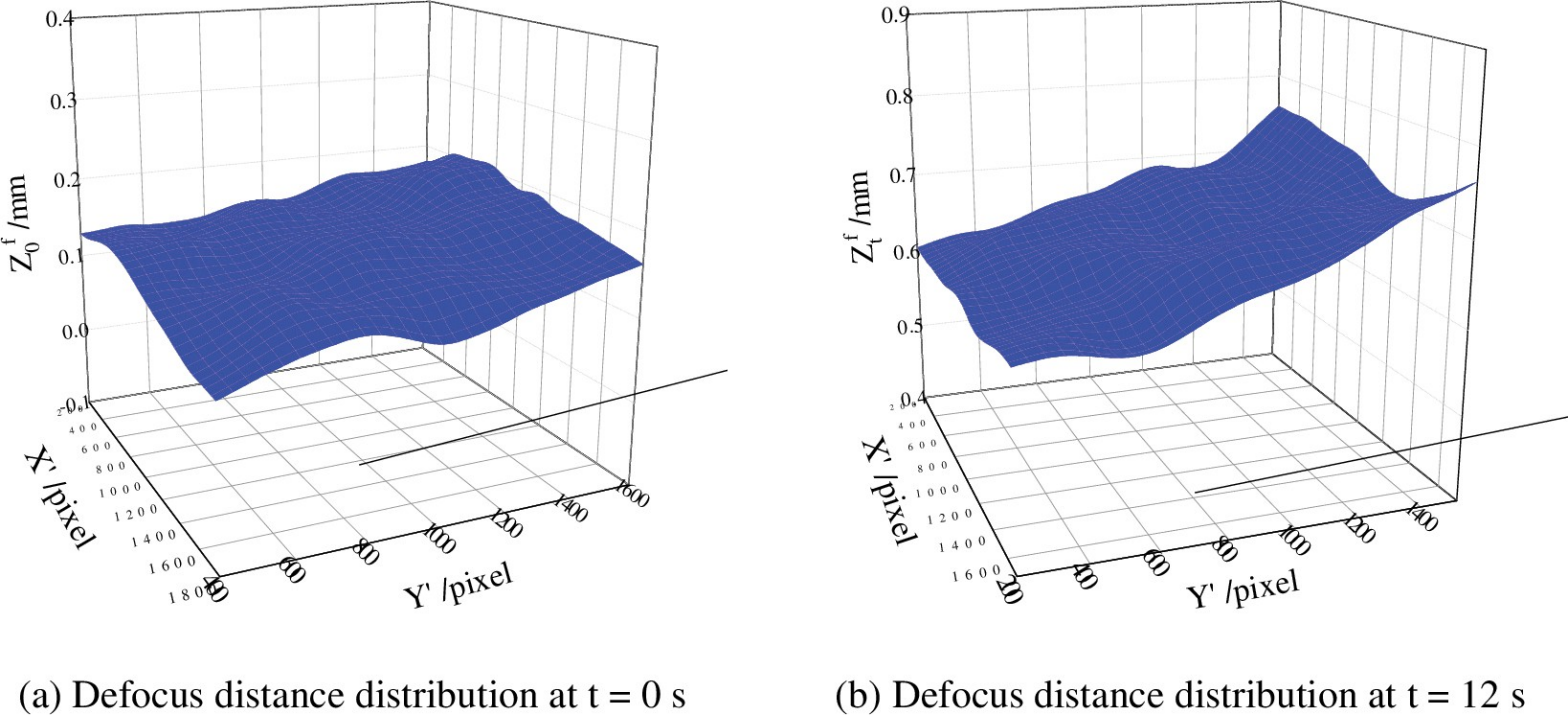
Defocus distance distribution in the measurement area at different time. (a) Defocus distance distribution at t = 0 s. (b) Defocus distance distribution at t = 12 s.

Where: $w_{t}^{f}$ is out of plane displacement of the front test specimens. $w_{t}^{b}$ is out of plane displacement of back of the test specimens. $Z_{0}^{f}$ is the defocusing distance of the front specimen before deformation and $Z_{0}^{b}$ is the defocusing distance of the back specimen before deformation. The defocusing distance of the measurement area at time t = 0 s and time t = 12 s were shown in **Fig** 22(A) and 22(B) respectively.

According to the fixture corners shown in the **Fig** 19(A), two pairs of pixel points with the same (*x*,*y*) on the front and back surfaces of the test piece could be matched. The total out of plane displacement of each point on the test specimen was as follows:

$$w_{t}(X',Y')=w_{t}^{f}(X',Y')+w_{t}^{b}(X',Y')$$ (32)

The engineering strain in the thickness direction is:

$$\varepsilon_{e3}=w_{t}/h_{t}$$ (33)

Where *h*_*t*_ is the initial thickness of test specimen.

**Fig 23:**
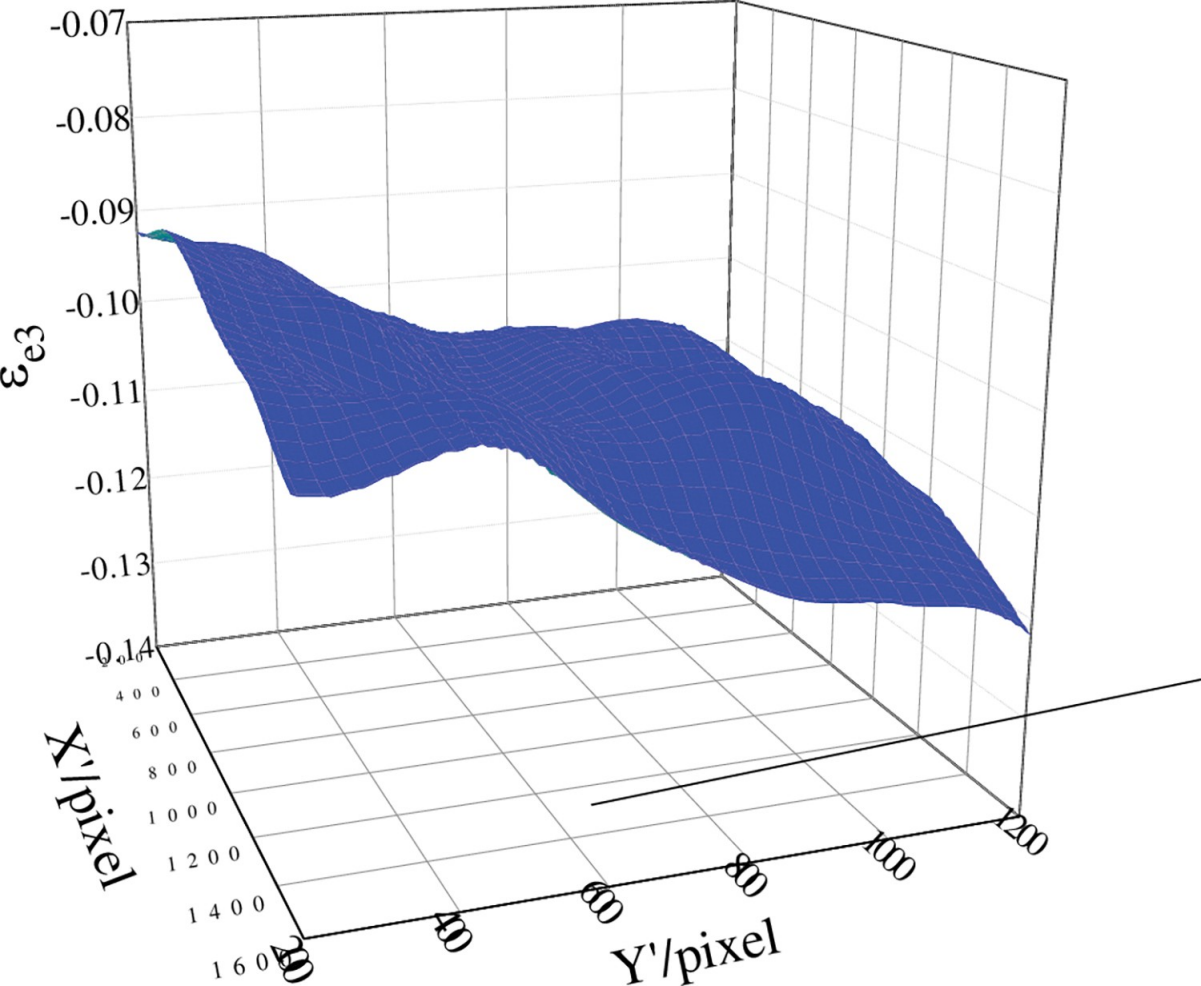
Engineering strain in the thickness direction in the measurement area when t = 12 s.

The engineering strain in the thickness direction of the measurement area when t = 12 s was shown in **Fig** 23. It could be seen from the **Fig** 23 that the engineering strain in the thickness direction changed in a small range. So the average value of all thickness direction strains in the measurement area could be taken as the thickness direction strain of the specimen surface at that time.

**Fig 24:**
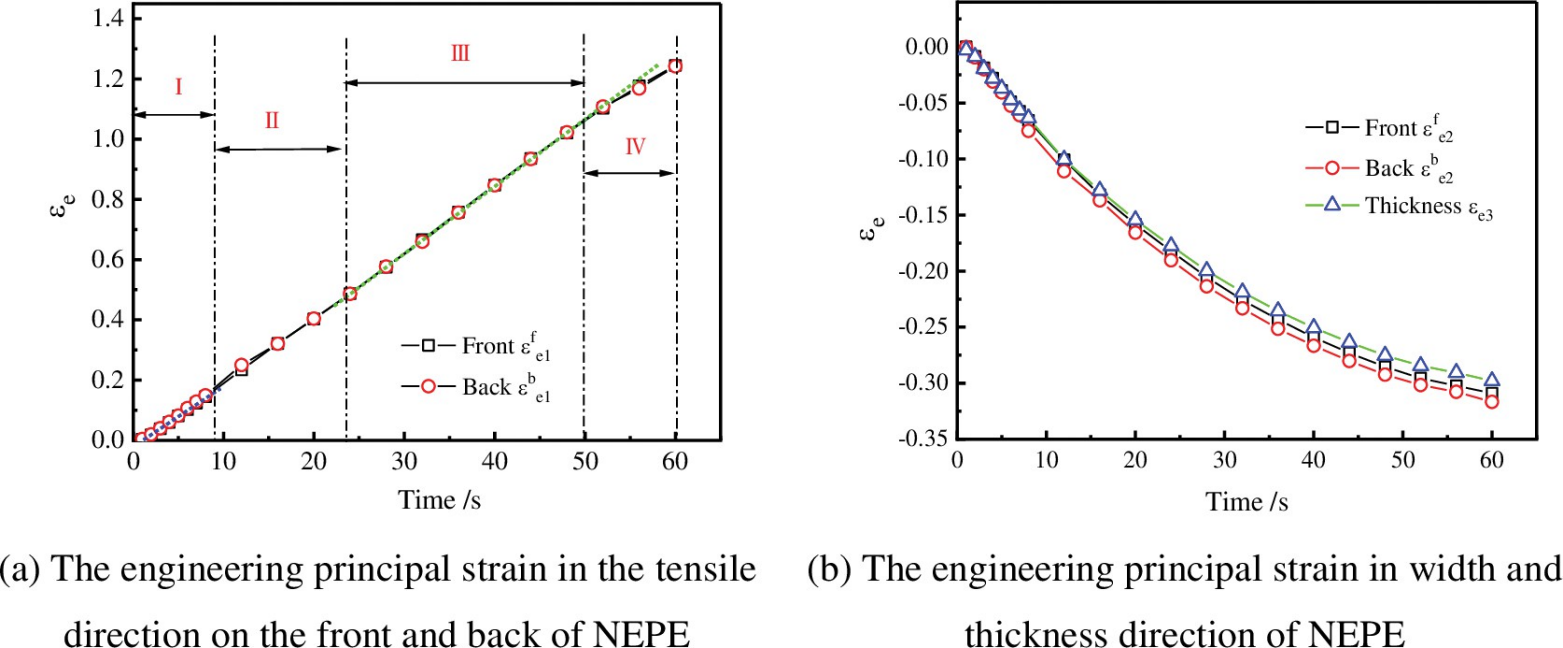
Engineering principal strain in tensile direction at different time (100 mm/min). (a) The engineering principal strain in the tensile direction on the front and back of NEPE. (b) The engineering principal strain in width and thickness direction of NEPE.

The engineering principal strain in the tensile direction of NEPE at different times under the tensile rate of 100 mm/min were shown in **Fig** 24(A). The square point in the **Fig** 24(A) is the engineering principal strain $\varepsilon_{e1}^{f}$ in the tensile direction of the front of the specimen, and the dot is the engineering principal strain $\varepsilon_{e1}^{b}$ in the tensile direction of the back. They are very close, and their average values are taken as the engineering principal strain in the tensile direction of the specimen. The formula is as follows:

$$\varepsilon_{e1}=(\varepsilon_{e1}^{f}+\varepsilon_{e1}^{b})/2$$ (34)

The engineering principal strain in width and thickness direction of NEPE at different times under the tensile rate of 100 mm/min were shown in **Fig** 24(B). The square point in the **Fig** 24(B) is the engineering principal strain $\varepsilon_{e2}^{f}$ in the width direction on the front of the specimen, and the circle point is the engineering principal strain $\varepsilon_{e2}^{b}$ in the width direction on the back. They are very close, and their average values are taken as the engineering principal strain in the width direction of the specimen. The formula is as follows:

$$\varepsilon_{e2}=(\varepsilon_{e2}^{f}+\varepsilon_{e2}^{b})/2$$ (35)

The primary strain *ε*_*e*3_ in the thickness direction is shown as a triangle in **Fig** 24(B). It is clear that the thickness direction experiences less primary strain than the width direction. There are two potential causes. One results from a geometric mistake made during manufacturing the material. Second, the material’s particle distribution is dispersive. There are disparities in shrinkage stresses in the thickness direction and width direction when dehumidification takes place because the distribution of voids at the particle-matrix interface is dispersive.

**Fig 25:**
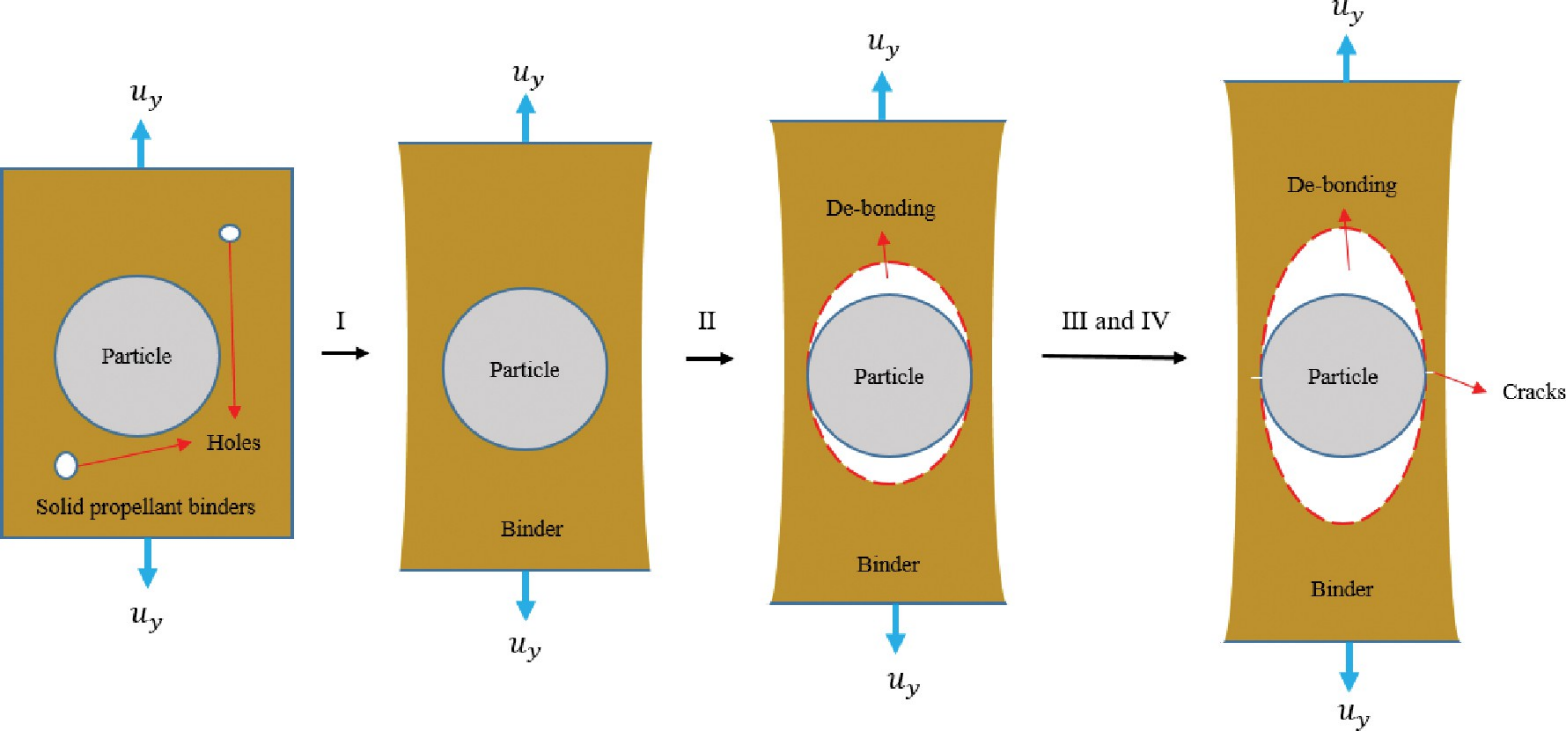
Microscopic changes in the interior of solid propellants under uniaxial tension.

From **Fig** 24, it can be seen that the time strain curve measured based on the photometry method proposed in this article is not strictly linear. Based on the microscopic analysis results of the scanning electron microscope experiment conducted on solid propellants in reference [33] during the loading process, as well as the slope changes corresponding to the time-strain curve measured in this paper, the stretching process is divided into four stages as follows:

The first stage is the overall bearing stage, during which the holes inside the solid propellant will be squeezed out due to stress; The second stage is the particle debonding stage, where sudden particle debonding leads to a sudden increase in the slope of the strain time curve in the tensile direction, while the slope of the strain time curve in other directions suddenly decreases; The third stage is the matrix bearing stage, where due to the detachment of particles from the matrix, the particles no longer bear the load, so the slope of the strain time curve does not change significantly; The fourth stage is the matrix damage and fracture stage. The microscopic changes inside the solid propellant corresponding to the above four stages are shown in **Fig** 25.

### 4.3 Volume deformation and Poisson’s ratio calculation

NEPE solid propellants undergo volume changes when subjected to tensile loading due to particle debonding and the formation of vacuum holes in the matrix itself during fabrication, which is accompanied by the nucleation and growth of vacuum holes. Accurate measurement of the volume change rate of the object in the deformation process is of great significance for obtaining the true stress-strain curve and establishing the constitutive model. According to the principal strains in three directions obtained above, the volume change rate of solid propellant NEPE during stretching could be given. The calculation formula of volume change rate is:

$$\frac{V}{V_{0}}=(1+\varepsilon_{e1}^{})(1+\varepsilon_{e2}^{})(1+\varepsilon_{e3})$$ (36)

Poisson’s ratio is another important mechanical parameter of materials. Because the tensile deformation of NEPE solid propellant belongs to large deformation, the strain when Poisson’s ratio is defined should be true strain. The relationship between true strain and engineering strain is:

$$\varepsilon_{\mathrm{true}}=\ln(1+\varepsilon_{e})$$ (37)

Where *ε*_*true*_ is the true strain, *ε*_*e*_ is the engineering strain. By using the **Eq** 37, the engineering principal strain in each direction can be transformed into the true strain in the corresponding direction.

Regarding the Poisson’s ratio of the material, it is an elastic constant reflecting the transverse deformation of the material. Because NEPE solid propellant is an isotropic material, and because the strains measured in this paper in the width direction and the thickness direction in the uniaxial stretching process as shown in **Fig** 24(B) in the text, the change rules and values are almost equal. So this paper chooses to calculate Poisson’s ratio directly by averaging the strains in both directions. The transverse shrinkage true strain can be defined as the average of the true strain in the width direction and the true strain in the thickness direction, so the Poisson’s ratio can be defined as:

$$\nu =-\frac{(\varepsilon_{true2}+\varepsilon_{true3})/2}{\varepsilon_{true1}}$$ (38)

Where *v* is the Poisson’s ratio, *ε*_*true*1_ is the true strain in tensile direction, *ε*_*true*2_ is the true strain in width direction and *ε*_*true*3_ is the true strain in thickness direction.

**Fig 26:**
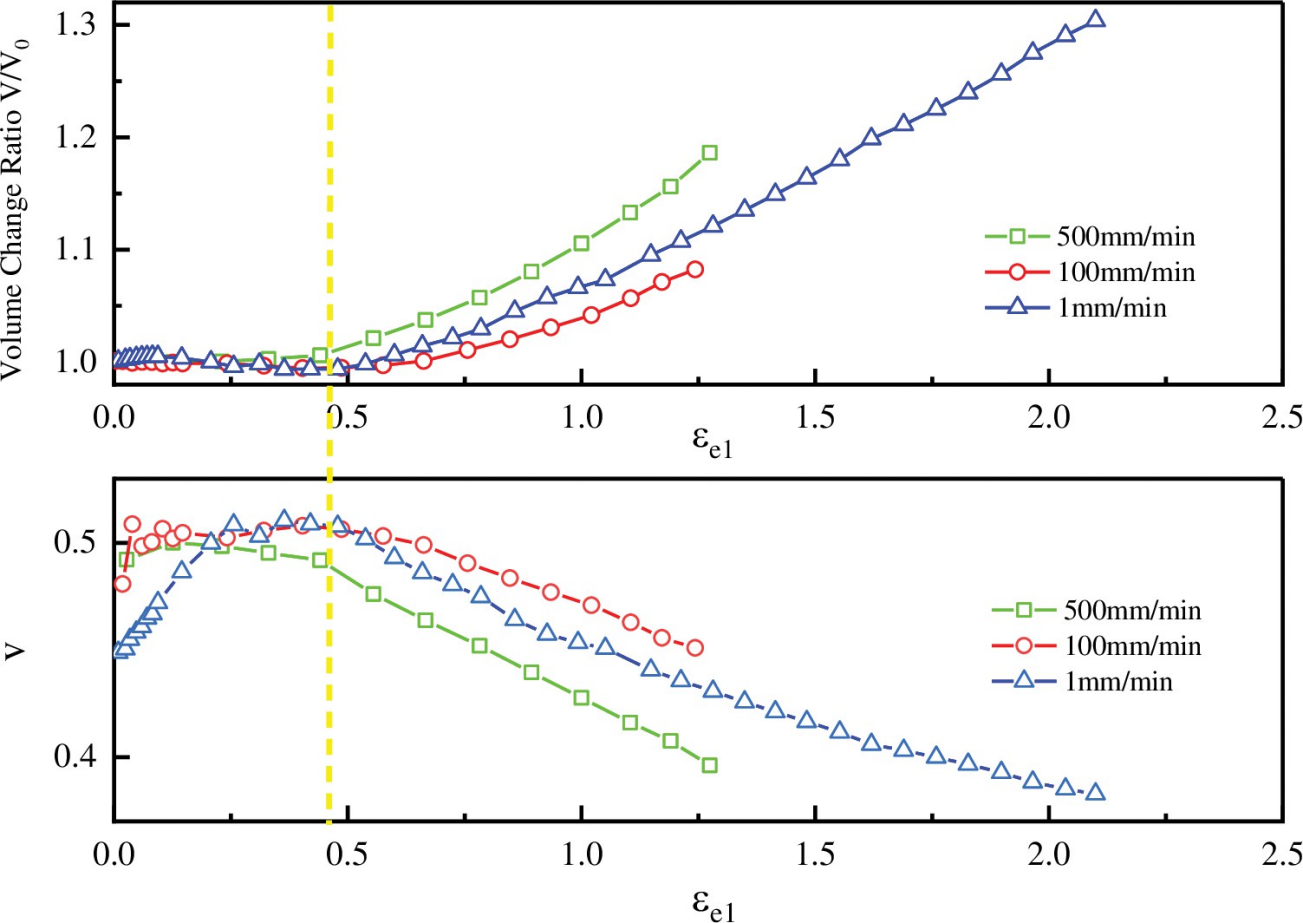
The volume change rate and the variation of Poisson’s ratio of NEPE with the engineering principal.

The volume change rate of NEPE under different loading rates and the variation of Poisson’s ratio of NEPE with the engineering principal strain *ε*_*e*1_ in the tensile direction at different loading rates were shown in were shown in **Fig** 26.

It can be seen from **Fig** 26 that in the initial stage of tension, the volume change rate is close to 1, and the Poisson’s ratio fluctuates in the range of close to 0.5, which means NEPE is close to Hyperelastic material material. When the engineering principal strain in the tensile direction reaches a certain value, the volume change rate starts to increase and the Poisson’s ratio starts to decline. This is defined as the starting point of destruction in this article. Then, as the volume change rate increases sharply, it shows an approximate linear growth until the material fractures. Based on the basis of the judgement of particle debonding of solid propellants in the literature [24, 34] when nonlinear mechanical properties of the material occur during stretching the particles start to de-bond at the same time the Poisson’s ratio of the material at this time decreases with the de-bonding of the particles. Therefore, in this paper, based on the measurement results of Poisson’s ratio and volume change rate of NEPE solid propellant during uniaxial stretching in **Fig** 26, the strain point corresponding to the yellow dotted line in **Fig** 26 is considered to be the onset of debonding of NEPE solid propellant particles from the substrate at this moment. It can also be seen from **Fig** 26 that the volume change rate curve at different loading rates and the change trend of Poisson’s ratio are consistent. When the NEPE solid propellant particles start to dehumidify, there will be some slight differences in the measured volumetric rate of change and Poisson’s ratio measurements at different tensile velocities. These differences may be caused by the differences in the different samples themselves, so it can be considered that the tensile rate has no effect on the volume change rate of NEPE. Similarly, by studying a solid propellant [35], it was found that the trend of volume expansion curves under different strain rates is consistent, and the dispersion is small. It is believed that the stretching rate has no effect on the rate of volume change of the solid propellant. The slopes of each point on the curve were calculated in **Fig** 26, and it was found that the slope in the elastic stage was zero, while the slope in the failure stage was greater than zero. Based on the experimental results measured in this paper, the point where the slope is greater than 0.01 for the first time is defined as the damage starting point, and the damage starting points of 500 mm/min, 100 mm/min and 1 mm/min tensile testing are 0.4411, 0.4866 and 0.4785 respectively. The reason why this paper chooses the strain point when the slope of the volume rate of change curve is greater than 0.01 for the first time to be defined as the damage initiation point is because at the beginning of the volume rate of change curve, the slope is approximately 0 but has some fluctuation in a very small range, so if the point when the slope is greater than 0 is chosen directly as the damage initiation point, the point is not accurate. Observation of the experimental results found that when the slope is greater than 0.01 after the volume rate of change curve is very stable, so this paper chooses the slope is equal to 0.01 when the point of particle debonding start point to avoid experimental errors. In this article, the average value of the damage starting point for the three sets of experiments is 0.4687, which will be considered as the critical strain of NEPE material damage during the tensile process.

When NEPE solid propellants were compared to those observed by scanning electron microscopy in the literature [36] during quasi-static uniaxial stretching, it was discovered that significant dewetting started to appear near the large particles at strain of 0.4 and that significant penetration-type cracks started to appear near the large particles at strain of 0.54. On the basis of the three different strain rates, it can be observed that the damage strain points produced are accurate. The three breaking strain points for this material’s material were all around 0.47 and did not differ significantly in value due to the different distribution of big particles within it. In this paper, the average value of the damage starting point of the three groups of experiments was 0.4687 which could be regarded as the damage critical strain of NEPE material in the tensile process.

### 4.4 Stress strain curves

Changes in the cross-sectional area of the specimen can be obtained by simultaneously detecting changes in width and thickness. Combined with the load data recorded by the testing machine, the true stress of the specimen in the tensile process could be obtained. The calculation formula of cross-sectional area is as follows:

$$S=(\frac{w}{w_{0}})(\frac{h}{h_{0}})S_{0}=(1+\varepsilon_{e2})(1+\varepsilon_{e3})S_{0}$$ (39)

Where *w*_0_ is the width before deformation, *w* is the width after deformation, *h*_0_ is the thickness before deformation, *h* is the thickness after deformation and *S*_0_ is the initial cross-sectional area.

**Fig 27:**
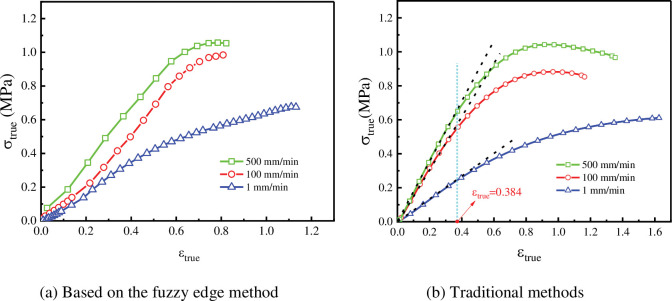
The true stress and true strain curves under different tensile rates. (a) Based on the fuzzy edge method. (b) Traditional methods.

The true stress of the specimen could be obtained by dividing the load recorded with the testing machine by the cross-sectional area of the specimen. The true stress and true strain curve of solid propellant NEPE under different tensile rates were shown in **Fig** 27(A). It could be seen from the **Fig 27** that with the increase of loading rate, the maximum stress that NEPE also increased, and the modulus of initial elastic stage also increased. This phenomenon was caused by the viscoelasticity of polymer materials. When the tensile speed was relatively low, the movement of the chain segment could completely keep up with the change of stress, and the tensile speed had little effect on the strength. When the stretching speed increased to a certain stage, the movement of the chain segment could not keep up with the external force, so that the same deformation of the material required greater external force, that is, the resistance offered by the material to deformation had increased.

The true stress and true strain curves of solid propellant NEPE obtained based on traditional methods at different tensile rates are shown in **Fig** 27(B). The true stress and true strain values in the results of the conventional method were obtained according to the Chinese aerospace industry standard of P.R.C, QJ 924–85 and the American JANNAF standard. The formulas for calculating the engineering strain by the conventional method are given below:

$$\varepsilon_{e}=\frac{L-L_{0}}{L_{0}}$$ (40)

Where: *L*_0_ is the initial marking length of the specimen in mm; *L* is the length of the specimen during stretching of the specimen in mm.

The *L* value was measured by a displacement transducer on the tester. The true strain in the traditional method is calculated as Eq 37. From the literature [37] for solid propellant damage measurements and observations, it is known that when the initial modulus of elasticity of the solid propellant stress-strain curve changes the solid propellant damage begins to occur. As can be seen in **Fig** 27(B), the NEPE solid propellant at three tensile rates starts to be damaged when the true strain *ε*_*true*_ = 0.384. From **Eq** 37 it is in perfect agreement with the damage initiation point *ε*_*e*_ = 0.4687 determined in this paper by the volume change rate and Poisson’s ratio. Since the NEPE solid propellant damage initiation points determined by the two measurement methods are identical, it could be demonstrated that the optical measurement method proposed in this paper is accurate in measuring strain.

The formulas for calculating the true stress by the conventional method are given below:

$$\sigma_{e}=\frac{F}{A_{0}}$$ (41)

$$\sigma_{ture}=(1+\varepsilon_{e})\sigma_{e}$$ (42)

Where: *σ*_*e*_ is the engineering stress in MPa; *σ*_*true*_ is the true stress in MPa; *A*_0_ is the cross-sectional area of the specimen at the initial moment in mm^2^; *F* is the experimentally loaded load in KN; *ε*_*e*_ is the engineering strain.

The traditional method of calculating the experimental value of the true stress of a specimen presupposes that the rate of change of the volume of the specimen during the tensile test is zero. However, the volume change rate of the specimen during uniaxial tensile testing measured based on the optical method proposed in this paper is shown in **Fig** 26. It is almost constant before the specimen is damaged. However, as the damage occurs, there is a significant change in its volume. That is to say, the traditional method is inaccurate in calculating the true stress during specimen stretching. However, based on the optical measurement method proposed in this paper, it is done by accurately recording the change in the cross-sectional area of the specimen during stretching and then directly calculating the true stress value during the test by using **Eq** 41.

The failure strain values *ε*_max_ corresponding to the maximum failure stresses *σ*_max_ of the NEPE solid propellant at the same temperature of 20° and at a tensile rate of 100 mm/min and 500 mm/min were compared with those of literature [38] respectively, as shown in Table 2. Compared with the same test conditions in Table 2, the maximum failure stress and failure strain with the loading speed of the change rule was consistent, and the size of their corresponding values were also very close. It could be proved that the uniaxial tensile test results of NEPE solid propellant based on the optical measurement method proposed in this paper were reliable.

**Table 2:** Comparison table of maximum failure stresses and failure strains.

Classifications	Tensile Rate(mm/min)	*ε*_max_	*σ*_max_ (MPa)
Traditional methods [38]	100	0.7839	1.08
	500	0.7608	1.29
Methodology of this article	100	0.8080	0.9839
	500	0.7844	1.0570

In conclusion, it can be seen that the optical measurement method proposed in this paper has good accuracy and practicality in measuring the mechanical properties of special materials.

## 5 Conclusions

In this paper, a three-dimensional measurement method based on out-of-focus fuzzy edges was proposed, its principle was described in detail and the accuracy of the method was verified experimentally, and the volume change rate, Poisson’s ratio, and real-time strain of NEPE solid propellant materials during uniaxial stretching were measured by using this method and the dic method. The following conclusions were drawn:

- Based on the optical measurement method in this paper by performing 3D measurements on differently placed cylinders, the errors of the measurement results were within 0.3 mm and the relative errors were all very small approximately equal to 0. The accuracy of the optical measurement method in this paper was proved.
- The in-plane strain in the direction of specimen stretching and width direction was measured using the DIC method, and the out-of-plane strain in the direction of specimen thickness was successfully measured using the optical measurement method in this paper. The full-field strain at each point on the specimen surface during uniaxial stretching was measured. The volumetric rate of change and Poisson’s ratio of NEPE solid propellant were obtained based on the real-time measured strains. The experimental results showed that the variation rules of the volume change rate and Poisson’s ratio of NEPE solid propellant during uniaxial stretching were independent of the rate of loading in the uniaxial stretching process. That is to say, the change trend of the volume change rate curve under different loading rates was consistent, and the stretching rate had no effect on the volume change rate of NEPE. And based on the change of volume change rate and Poisson’s ratio with strain, it was concluded that the damage initiation engineering strain of NEPE solid propellant was 0.4687.
- The true stress and true strain curves of the solid propellant NEPE at different tensile rates were determined by incorporating the loads measured by the experimental equipment. The experimental results showed that the load carrying capacity of NEPE increases dramatically with the increase of tensile rate. At the beginning of stretching, NEPE was almost a superelastic material. By comparing the measured calculated true stress-true strain curves with the traditional method. It was determined that the method proposed in this paper could accurately measure the mechanical properties of NEPE junction.

## Supporting information

S1 File (DOCX)
