# Supplementary material for: A simple and highly accurate method for fuzzy edge-based 3D morphometry
Source: PLoS One. 2023 Dec 15;18(12):e0295988. doi: 10.1371/journal.pone.0295988 (PMC10723708; doi:10.1371/journal.pone.0295988)
Supplement: S1 File — (DOCX) [file pone.0295988.s001.docx]

Data on light intensity values in Figure 3：

| pixel coordinate | Blurred picture light intensity values | pixel coordinate | Focus clear picture light intensity |
| --- | --- | --- | --- |
| -1 | 0 | -1 | 0 |
| -0.99 | 0.000599 | -0.99 | 0 |
| -0.98 | 0.001693 | -0.98 | 0 |
| -0.97 | 0.003105 | -0.97 | 0 |
| -0.96 | 0.004773 | -0.96 | 0 |
| -0.95 | 0.00666 | -0.95 | 0 |
| -0.94 | 0.008741 | -0.94 | 0 |
| -0.93 | 0.010999 | -0.93 | 0 |
| -0.92 | 0.013417 | -0.92 | 0 |
| -0.91 | 0.015985 | -0.91 | 0 |
| -0.9 | 0.018693 | -0.9 | 0 |
| -0.89 | 0.021533 | -0.89 | 0 |
| -0.88 | 0.024496 | -0.88 | 0 |
| -0.87 | 0.027578 | -0.87 | 0 |
| -0.86 | 0.030772 | -0.86 | 0 |
| -0.85 | 0.034074 | -0.85 | 0 |
| -0.84 | 0.037478 | -0.84 | 0 |
| -0.83 | 0.040981 | -0.83 | 0 |
| -0.82 | 0.044578 | -0.82 | 0 |
| -0.81 | 0.048267 | -0.81 | 0 |
| -0.8 | 0.052044 | -0.8 | 0 |
| -0.79 | 0.055906 | -0.79 | 0 |
| -0.78 | 0.059849 | -0.78 | 0 |
| -0.77 | 0.063873 | -0.77 | 0 |
| -0.76 | 0.067972 | -0.76 | 0 |
| -0.75 | 0.072147 | -0.75 | 0 |
| -0.74 | 0.076393 | -0.74 | 0 |
| -0.73 | 0.08071 | -0.73 | 0 |
| -0.72 | 0.085095 | -0.72 | 0 |
| -0.71 | 0.089545 | -0.71 | 0 |
| -0.7 | 0.09406 | -0.7 | 0 |
| -0.69 | 0.098638 | -0.69 | 0 |
| -0.68 | 0.103276 | -0.68 | 0 |
| -0.67 | 0.107973 | -0.67 | 0 |
| -0.66 | 0.112727 | -0.66 | 0 |
| -0.65 | 0.117538 | -0.65 | 0 |
| -0.64 | 0.122402 | -0.64 | 0 |
| -0.63 | 0.12732 | -0.63 | 0 |
| -0.62 | 0.13229 | -0.62 | 0 |
| -0.61 | 0.13731 | -0.61 | 0 |
| -0.6 | 0.142379 | -0.6 | 0 |
| -0.59 | 0.147495 | -0.59 | 0 |
| -0.58 | 0.152658 | -0.58 | 0 |
| -0.57 | 0.157867 | -0.57 | 0 |
| -0.56 | 0.16312 | -0.56 | 0 |
| -0.55 | 0.168415 | -0.55 | 0 |
| -0.54 | 0.173753 | -0.54 | 0 |
| -0.53 | 0.179131 | -0.53 | 0 |
| -0.52 | 0.18455 | -0.52 | 0 |
| -0.51 | 0.190007 | -0.51 | 0 |
| -0.5 | 0.195501 | -0.5 | 0 |
| -0.49 | 0.201033 | -0.49 | 0 |
| -0.48 | 0.2066 | -0.48 | 0 |
| -0.47 | 0.212202 | -0.47 | 0 |
| -0.46 | 0.217838 | -0.46 | 0 |
| -0.45 | 0.223507 | -0.45 | 0 |
| -0.44 | 0.229208 | -0.44 | 0 |
| -0.43 | 0.234941 | -0.43 | 0 |
| -0.42 | 0.240703 | -0.42 | 0 |
| -0.41 | 0.246495 | -0.41 | 0 |
| -0.4 | 0.252316 | -0.4 | 0 |
| -0.39 | 0.258164 | -0.39 | 0 |
| -0.38 | 0.26404 | -0.38 | 0 |
| -0.37 | 0.269942 | -0.37 | 0 |
| -0.36 | 0.275868 | -0.36 | 0 |
| -0.35 | 0.28182 | -0.35 | 0 |
| -0.34 | 0.287795 | -0.34 | 0 |
| -0.33 | 0.293794 | -0.33 | 0 |
| -0.32 | 0.299814 | -0.32 | 0 |
| -0.31 | 0.305856 | -0.31 | 0 |
| -0.3 | 0.311919 | -0.3 | 0 |
| -0.29 | 0.318002 | -0.29 | 0 |
| -0.28 | 0.324104 | -0.28 | 0 |
| -0.27 | 0.330225 | -0.27 | 0 |
| -0.26 | 0.336363 | -0.26 | 0 |
| -0.25 | 0.342519 | -0.25 | 0 |
| -0.24 | 0.348691 | -0.24 | 0 |
| -0.23 | 0.354879 | -0.23 | 0 |
| -0.22 | 0.361082 | -0.22 | 0 |
| -0.21 | 0.367299 | -0.21 | 0 |
| -0.2 | 0.37353 | -0.2 | 0 |
| -0.19 | 0.379774 | -0.19 | 0 |
| -0.18 | 0.386031 | -0.18 | 0 |
| -0.17 | 0.392299 | -0.17 | 0 |
| -0.16 | 0.398577 | -0.16 | 0 |
| -0.15 | 0.404867 | -0.15 | 0 |
| -0.14 | 0.411166 | -0.14 | 0 |
| -0.13 | 0.417473 | -0.13 | 0 |
| -0.12 | 0.42379 | -0.12 | 0 |
| -0.11 | 0.430114 | -0.11 | 0 |
| -0.1 | 0.436445 | -0.1 | 0 |
| -0.09 | 0.442782 | -0.09 | 0 |
| -0.08 | 0.449125 | -0.08 | 0 |
| -0.07 | 0.455473 | -0.07 | 0 |
| -0.06 | 0.461826 | -0.06 | 0 |
| -0.05 | 0.468183 | -0.05 | 0 |
| -0.04 | 0.474542 | -0.04 | 0 |
| -0.03 | 0.480905 | -0.03 | 0 |
| -0.02 | 0.487269 | -0.02 | 0 |
| -0.01 | 0.493634 | -0.01 | 0 |
| 0 | 0.5 | 0 | 0.5 |
| 0.01 | 0.506367 | 0.01 | 1 |
| 0.02 | 0.512732 | 0.02 | 1 |
| 0.03 | 0.519096 | 0.03 | 1 |
| 0.04 | 0.525458 | 0.04 | 1 |
| 0.05 | 0.531818 | 0.05 | 1 |
| 0.06 | 0.538175 | 0.06 | 1 |
| 0.07 | 0.544527 | 0.07 | 1 |
| 0.08 | 0.550876 | 0.08 | 1 |
| 0.09 | 0.557219 | 0.09 | 1 |
| 0.1 | 0.563556 | 0.1 | 1 |
| 0.11 | 0.569887 | 0.11 | 1 |
| 0.12 | 0.576211 | 0.12 | 1 |
| 0.13 | 0.582527 | 0.13 | 1 |
| 0.14 | 0.588835 | 0.14 | 1 |
| 0.15 | 0.595134 | 0.15 | 1 |
| 0.16 | 0.601423 | 0.16 | 1 |
| 0.17 | 0.607702 | 0.17 | 1 |
| 0.18 | 0.61397 | 0.18 | 1 |
| 0.19 | 0.620227 | 0.19 | 1 |
| 0.2 | 0.62647 | 0.2 | 1 |
| 0.21 | 0.632701 | 0.21 | 1 |
| 0.22 | 0.638919 | 0.22 | 1 |
| 0.23 | 0.645122 | 0.23 | 1 |
| 0.24 | 0.65131 | 0.24 | 1 |
| 0.25 | 0.657482 | 0.25 | 1 |
| 0.26 | 0.663637 | 0.26 | 1 |
| 0.27 | 0.669776 | 0.27 | 1 |
| 0.28 | 0.675897 | 0.28 | 1 |
| 0.29 | 0.681999 | 0.29 | 1 |
| 0.3 | 0.688082 | 0.3 | 1 |
| 0.31 | 0.694145 | 0.31 | 1 |
| 0.32 | 0.700187 | 0.32 | 1 |
| 0.33 | 0.706207 | 0.33 | 1 |
| 0.34 | 0.712206 | 0.34 | 1 |
| 0.35 | 0.718181 | 0.35 | 1 |
| 0.36 | 0.724132 | 0.36 | 1 |
| 0.37 | 0.730059 | 0.37 | 1 |
| 0.38 | 0.735961 | 0.38 | 1 |
| 0.39 | 0.741836 | 0.39 | 1 |
| 0.4 | 0.747685 | 0.4 | 1 |
| 0.41 | 0.753506 | 0.41 | 1 |
| 0.42 | 0.759298 | 0.42 | 1 |
| 0.43 | 0.76506 | 0.43 | 1 |
| 0.44 | 0.770793 | 0.44 | 1 |
| 0.45 | 0.776494 | 0.45 | 1 |
| 0.46 | 0.782163 | 0.46 | 1 |
| 0.47 | 0.787799 | 0.47 | 1 |
| 0.48 | 0.793401 | 0.48 | 1 |
| 0.49 | 0.798968 | 0.49 | 1 |
| 0.5 | 0.8045 | 0.5 | 1 |
| 0.51 | 0.809994 | 0.51 | 1 |
| 0.52 | 0.815451 | 0.52 | 1 |
| 0.53 | 0.82087 | 0.53 | 1 |
| 0.54 | 0.826248 | 0.54 | 1 |
| 0.55 | 0.831586 | 0.55 | 1 |
| 0.56 | 0.836881 | 0.56 | 1 |
| 0.57 | 0.842134 | 0.57 | 1 |
| 0.58 | 0.847342 | 0.58 | 1 |
| 0.59 | 0.852506 | 0.59 | 1 |
| 0.6 | 0.857622 | 0.6 | 1 |
| 0.61 | 0.862691 | 0.61 | 1 |
| 0.62 | 0.867711 | 0.62 | 1 |
| 0.63 | 0.872681 | 0.63 | 1 |
| 0.64 | 0.877598 | 0.64 | 1 |
| 0.65 | 0.882463 | 0.65 | 1 |
| 0.66 | 0.887274 | 0.66 | 1 |
| 0.67 | 0.892028 | 0.67 | 1 |
| 0.68 | 0.896725 | 0.68 | 1 |
| 0.69 | 0.901363 | 0.69 | 1 |
| 0.7 | 0.905941 | 0.7 | 1 |
| 0.71 | 0.910455 | 0.71 | 1 |
| 0.72 | 0.914906 | 0.72 | 1 |
| 0.73 | 0.919291 | 0.73 | 1 |
| 0.74 | 0.923607 | 0.74 | 1 |
| 0.75 | 0.927854 | 0.75 | 1 |
| 0.76 | 0.932028 | 0.76 | 1 |
| 0.77 | 0.936128 | 0.77 | 1 |
| 0.78 | 0.940151 | 0.78 | 1 |
| 0.79 | 0.944095 | 0.79 | 1 |
| 0.8 | 0.947957 | 0.8 | 1 |
| 0.81 | 0.951734 | 0.81 | 1 |
| 0.82 | 0.955422 | 0.82 | 1 |
| 0.83 | 0.95902 | 0.83 | 1 |
| 0.84 | 0.962523 | 0.84 | 1 |
| 0.85 | 0.965927 | 0.85 | 1 |
| 0.86 | 0.969229 | 0.86 | 1 |
| 0.87 | 0.972423 | 0.87 | 1 |
| 0.88 | 0.975505 | 0.88 | 1 |
| 0.89 | 0.978468 | 0.89 | 1 |
| 0.9 | 0.981308 | 0.9 | 1 |
| 0.91 | 0.984016 | 0.91 | 1 |
| 0.92 | 0.986584 | 0.92 | 1 |
| 0.93 | 0.989002 | 0.93 | 1 |
| 0.94 | 0.991259 | 0.94 | 1 |
| 0.95 | 0.993341 | 0.95 | 1 |
| 0.96 | 0.995228 | 0.96 | 1 |
| 0.97 | 0.996896 | 0.97 | 1 |
| 0.98 | 0.998308 | 0.98 | 1 |
| 0.99 | 0.999402 | 0.99 | 1 |
| 1 | 1.000001 | 1 | 1 |

Specific data in figure 6：

| Greyscale value | Relative light intensity value |
| --- | --- |
| 2.544207 | 0.003 |
| 5.544127 | 0.00375 |
| 4.204863 | 0.004688 |
| 5.989373 | 0.006 |
| 7.279312 | 0.0075 |
| 11.32688 | 0.009375 |
| 16.30836 | 0.012 |
| 18.09221 | 0.015 |
| 19.8601 | 0.01875 |
| 22.91662 | 0.024 |
| 25.15577 | 0.03 |
| 27.88977 | 0.0375 |
| 33.11391 | 0.05 |
| 37.8862 | 0.06 |
| 45.51687 | 0.075 |
| 52.3606 | 0.1 |
| 61.27339 | 0.12 |
| 71.13693 | 0.15 |
| 82.93557 | 0.2 |
| 96.09452 | 0.230769 |
| 110.9704 | 0.3 |
| 127.6666 | 0.375 |
| 145.6681 | 0.5 |
| 163.1471 | 0.6 |
| 179.862 | 0.75 |
| 196.8634 | 1 |
| 210.9804 | 1.2 |
| 225.6284 | 1.5 |
| 234.3543 | 1.875 |
| 238.6309 | 2.307692 |
| 243.961 | 3 |
| 248.0982 | 3.9 |
| 251.9227 | 4.8 |
| 253.2257 | 6 |
| 254 | 7.5 |
| 255 | 9 |

Light intensity-pixel curve data before filtering in Fig. 8 (a):

| Pixel | Light intensity |
| --- | --- |
| 1 | 0.014803 |
| 2 | 0.014803 |
| 3 | 0.014803 |
| 4 | 0.014803 |
| 5 | 0.014803 |
| 6 | 0.014803 |
| 7 | 0.014803 |
| 8 | 0.01572 |
| 9 | 0.01572 |
| 10 | 0.01572 |
| 11 | 0.015258 |
| 12 | 0.015258 |
| 13 | 0.014803 |
| 14 | 0.014803 |
| 15 | 0.014803 |
| 16 | 0.017638 |
| 17 | 0.017148 |
| 18 | 0.01572 |
| 19 | 0.015258 |
| 20 | 0.015258 |
| 21 | 0.01572 |
| 22 | 0.015258 |
| 23 | 0.01572 |
| 24 | 0.016665 |
| 25 | 0.016665 |
| 26 | 0.016665 |
| 27 | 0.01572 |
| 28 | 0.01572 |
| 29 | 0.014803 |
| 30 | 0.014803 |
| 31 | 0.014803 |
| 32 | 0.015453 |
| 33 | 0.015453 |
| 34 | 0.015918 |
| 35 | 0.016869 |
| 36 | 0.016869 |
| 37 | 0.016869 |
| 38 | 0.017355 |
| 39 | 0.017355 |
| 40 | 0.017676 |
| 41 | 0.02129 |
| 42 | 0.024634 |
| 43 | 0.028514 |
| 44 | 0.031856 |
| 45 | 0.042063 |
| 46 | 0.058405 |
| 47 | 0.071747 |
| 48 | 0.092362 |
| 49 | 0.103844 |
| 50 | 0.12372 |
| 51 | 0.142472 |
| 52 | 0.15901 |
| 53 | 0.178696 |
| 54 | 0.200609 |
| 55 | 0.218507 |
| 56 | 0.234453 |
| 57 | 0.256077 |
| 58 | 0.285948 |
| 59 | 0.311431 |
| 60 | 0.330276 |
| 61 | 0.350455 |
| 62 | 0.371484 |
| 63 | 0.39023 |
| 64 | 0.426561 |
| 65 | 0.441994 |
| 66 | 0.466212 |
| 67 | 0.491819 |
| 68 | 0.521274 |
| 69 | 0.547712 |
| 70 | 0.578272 |
| 71 | 0.599711 |
| 72 | 0.629104 |
| 73 | 0.637838 |
| 74 | 0.655429 |
| 75 | 0.673879 |
| 76 | 0.684884 |
| 77 | 0.686617 |
| 78 | 0.686254 |
| 79 | 0.686254 |
| 80 | 0.693034 |
| 81 | 0.683434 |
| 82 | 0.683434 |
| 83 | 0.689817 |
| 84 | 0.69952 |
| 85 | 0.702789 |
| 86 | 0.702789 |
| 87 | 0.696268 |
| 88 | 0.701008 |
| 89 | 0.701008 |
| 90 | 0.701008 |
| 91 | 0.701008 |
| 92 | 0.701008 |
| 93 | 0.701008 |
| 94 | 0.701008 |
| 95 | 0.701008 |
| 96 | 0.708333 |
| 97 | 0.705034 |
| 98 | 0.705034 |
| 99 | 0.705034 |
| 100 | 0.698491 |
| 101 | 0.698491 |
| 102 | 0.701754 |
| 103 | 0.698491 |
| 104 | 0.697008 |
| 105 | 0.697008 |
| 106 | 0.697008 |
| 107 | 0.697008 |
| 108 | 0.697008 |
| 109 | 0.697008 |
| 110 | 0.697008 |
| 111 | 0.697008 |
| 112 | 0.696268 |
| 113 | 0.696268 |
| 114 | 0.696268 |
| 115 | 0.696268 |
| 116 | 0.696268 |
| 117 | 0.696268 |
| 118 | 0.696268 |
| 119 | 0.696268 |
| 120 | 0.703536 |
| 121 | 0.703536 |
| 122 | 0.697008 |
| 123 | 0.697008 |
| 124 | 0.697008 |
| 125 | 0.697008 |
| 126 | 0.690549 |
| 127 | 0.690549 |
| 128 | 0.698491 |
| 129 | 0.698491 |
| 130 | 0.698491 |
| 131 | 0.698491 |
| 132 | 0.698491 |
| 133 | 0.698491 |
| 134 | 0.698491 |
| 135 | 0.698491 |
| 136 | 0.697008 |
| 137 | 0.697008 |
| 138 | 0.697008 |
| 139 | 0.697008 |
| 140 | 0.697008 |
| 141 | 0.697008 |
| 142 | 0.697008 |
| 143 | 0.697008 |
| 144 | 0.697008 |
| 145 | 0.697008 |
| 146 | 0.697008 |
| 147 | 0.697008 |
| 148 | 0.697008 |
| 149 | 0.697008 |
| 150 | 0.697008 |
| 151 | 0.697008 |
| 152 | 0.688074 |
| 153 | 0.688074 |
| 154 | 0.688074 |
| 155 | 0.688074 |
| 156 | 0.688074 |
| 157 | 0.688074 |
| 158 | 0.688074 |
| 159 | 0.688074 |
| 160 | 0.693034 |
| 161 | 0.693034 |
| 162 | 0.689817 |
| 163 | 0.689817 |
| 164 | 0.689817 |
| 165 | 0.689817 |
| 166 | 0.686617 |
| 167 | 0.686617 |
| 168 | 0.692016 |
| 169 | 0.688804 |
| 170 | 0.679271 |
| 171 | 0.669888 |
| 172 | 0.660653 |
| 173 | 0.648564 |
| 174 | 0.639663 |
| 175 | 0.633806 |
| 176 | 0.608196 |
| 177 | 0.599922 |
| 178 | 0.583754 |
| 179 | 0.568076 |
| 180 | 0.545438 |
| 181 | 0.530905 |
| 182 | 0.516799 |
| 183 | 0.5099 |
| 184 | 0.489797 |
| 185 | 0.476869 |
| 186 | 0.458148 |
| 187 | 0.434357 |
| 188 | 0.409972 |
| 189 | 0.386893 |
| 190 | 0.364992 |
| 191 | 0.352046 |
| 192 | 0.337761 |
| 193 | 0.315196 |
| 194 | 0.293696 |
| 195 | 0.271898 |
| 196 | 0.258029 |
| 197 | 0.238836 |
| 198 | 0.22092 |
| 199 | 0.202949 |
| 200 | 0.182503 |
| 201 | 0.166787 |
| 202 | 0.144418 |
| 203 | 0.125794 |
| 204 | 0.109226 |
| 205 | 0.091372 |
| 206 | 0.071798 |
| 207 | 0.057884 |
| 208 | 0.046063 |
| 209 | 0.037467 |
| 210 | 0.027959 |
| 211 | 0.022125 |
| 212 | 0.022881 |
| 213 | 0.020662 |
| 214 | 0.019272 |
| 215 | 0.016306 |
| 216 | 0.01759 |
| 217 | 0.01759 |
| 218 | 0.01759 |
| 219 | 0.01759 |
| 220 | 0.01759 |
| 221 | 0.01759 |
| 222 | 0.01759 |
| 223 | 0.01759 |
| 224 | 0.01759 |
| 225 | 0.01759 |
| 226 | 0.01759 |
| 227 | 0.016619 |
| 228 | 0.016619 |
| 229 | 0.015676 |
| 230 | 0.015676 |
| 231 | 0.015676 |
| 232 | 0.015676 |
| 233 | 0.015676 |
| 234 | 0.015676 |
| 235 | 0.015676 |
| 236 | 0.015676 |
| 237 | 0.015676 |
| 238 | 0.015676 |
| 239 | 0.015676 |
| 240 | 0.01759 |
| 241 | 0.01759 |
| 242 | 0.01759 |
| 243 | 0.01759 |
| 244 | 0.01759 |
| 245 | 0.01759 |
| 246 | 0.01759 |
| 247 | 0.01759 |
| 248 | 0.017101 |
| 249 | 0.017101 |
| 250 | 0.016619 |
| 251 | 0.016619 |

Light intensity-pixel curve data after filtering in Fig. 8 (b):

| Pixel | Light intensity |
| --- | --- |
| 1 | 0.01482 |
| 2 | 0.014793 |
| 3 | 0.014753 |
| 4 | 0.014807 |
| 5 | 0.01474 |
| 6 | 0.014793 |
| 7 | 0.014834 |
| 8 | 0.015018 |
| 9 | 0.015032 |
| 10 | 0.015045 |
| 11 | 0.015086 |
| 12 | 0.015113 |
| 13 | 0.015167 |
| 14 | 0.015154 |
| 15 | 0.015167 |
| 16 | 0.015702 |
| 17 | 0.015743 |
| 18 | 0.015647 |
| 19 | 0.015674 |
| 20 | 0.015702 |
| 21 | 0.015743 |
| 22 | 0.015647 |
| 23 | 0.015647 |
| 24 | 0.015385 |
| 25 | 0.015358 |
| 26 | 0.015605 |
| 27 | 0.015702 |
| 28 | 0.015702 |
| 29 | 0.015868 |
| 30 | 0.016344 |
| 31 | 0.016485 |
| 32 | 0.016527 |
| 33 | 0.016498 |
| 34 | 0.016456 |
| 35 | 0.016912 |
| 36 | 0.017417 |
| 37 | 0.017724 |
| 38 | 0.018152 |
| 39 | 0.018435 |
| 40 | 0.019023 |
| 41 | 0.022124 |
| 42 | 0.025247 |
| 43 | 0.029021 |
| 44 | 0.033577 |
| 45 | 0.043106 |
| 46 | 0.05706 |
| 47 | 0.068993 |
| 48 | 0.087506 |
| 49 | 0.100901 |
| 50 | 0.120553 |
| 51 | 0.138678 |
| 52 | 0.155524 |
| 53 | 0.175145 |
| 54 | 0.198464 |
| 55 | 0.21522 |
| 56 | 0.234822 |
| 57 | 0.253178 |
| 58 | 0.279197 |
| 59 | 0.302729 |
| 60 | 0.321741 |
| 61 | 0.344835 |
| 62 | 0.371913 |
| 63 | 0.39357 |
| 64 | 0.418349 |
| 65 | 0.439865 |
| 66 | 0.471942 |
| 67 | 0.496977 |
| 68 | 0.517652 |
| 69 | 0.539442 |
| 70 | 0.569087 |
| 71 | 0.594521 |
| 72 | 0.621475 |
| 73 | 0.631551 |
| 74 | 0.649161 |
| 75 | 0.668144 |
| 76 | 0.680679 |
| 77 | 0.686394 |
| 78 | 0.686016 |
| 79 | 0.686111 |
| 80 | 0.690667 |
| 81 | 0.690476 |
| 82 | 0.692292 |
| 83 | 0.692483 |
| 84 | 0.692866 |
| 85 | 0.693729 |
| 86 | 0.695554 |
| 87 | 0.695939 |
| 88 | 0.695248 |
| 89 | 0.695248 |
| 90 | 0.695152 |
| 91 | 0.694768 |
| 92 | 0.694768 |
| 93 | 0.695152 |
| 94 | 0.695056 |
| 95 | 0.695056 |
| 96 | 0.694591 |
| 97 | 0.694591 |
| 98 | 0.696129 |
| 99 | 0.696129 |
| 100 | 0.696129 |
| 101 | 0.696129 |
| 102 | 0.697672 |
| 103 | 0.697672 |
| 104 | 0.696022 |
| 105 | 0.696022 |
| 106 | 0.696792 |
| 107 | 0.696792 |
| 108 | 0.696889 |
| 109 | 0.697757 |
| 110 | 0.69853 |
| 111 | 0.698627 |
| 112 | 0.696968 |
| 113 | 0.696968 |
| 114 | 0.69774 |
| 115 | 0.69774 |
| 116 | 0.69774 |
| 117 | 0.69774 |
| 118 | 0.698513 |
| 119 | 0.698513 |
| 120 | 0.70025 |
| 121 | 0.70025 |
| 122 | 0.699475 |
| 123 | 0.699475 |
| 124 | 0.699475 |
| 125 | 0.699475 |
| 126 | 0.698701 |
| 127 | 0.698701 |
| 128 | 0.697525 |
| 129 | 0.697428 |
| 130 | 0.697332 |
| 131 | 0.697525 |
| 132 | 0.697428 |
| 133 | 0.697718 |
| 134 | 0.697621 |
| 135 | 0.697621 |
| 136 | 0.697254 |
| 137 | 0.697254 |
| 138 | 0.697254 |
| 139 | 0.697254 |
| 140 | 0.697254 |
| 141 | 0.697254 |
| 142 | 0.697254 |
| 143 | 0.697254 |
| 144 | 0.695937 |
| 145 | 0.696033 |
| 146 | 0.696129 |
| 147 | 0.695648 |
| 148 | 0.695456 |
| 149 | 0.695263 |
| 150 | 0.695071 |
| 151 | 0.694975 |
| 152 | 0.695009 |
| 153 | 0.695009 |
| 154 | 0.695009 |
| 155 | 0.69424 |
| 156 | 0.69424 |
| 157 | 0.69424 |
| 158 | 0.69424 |
| 159 | 0.69424 |
| 160 | 0.697076 |
| 161 | 0.696883 |
| 162 | 0.695246 |
| 163 | 0.693614 |
| 164 | 0.693422 |
| 165 | 0.693039 |
| 166 | 0.691412 |
| 167 | 0.691317 |
| 168 | 0.690328 |
| 169 | 0.687664 |
| 170 | 0.682087 |
| 171 | 0.673215 |
| 172 | 0.662288 |
| 173 | 0.649245 |
| 174 | 0.638062 |
| 175 | 0.630184 |
| 176 | 0.607041 |
| 177 | 0.599516 |
| 178 | 0.586599 |
| 179 | 0.570989 |
| 180 | 0.55102 |
| 181 | 0.534319 |
| 182 | 0.51866 |
| 183 | 0.50929 |
| 184 | 0.490006 |
| 185 | 0.472113 |
| 186 | 0.447874 |
| 187 | 0.429412 |
| 188 | 0.411975 |
| 189 | 0.391671 |
| 190 | 0.36912 |
| 191 | 0.353543 |
| 192 | 0.33403 |
| 193 | 0.315032 |
| 194 | 0.292335 |
| 195 | 0.272947 |
| 196 | 0.260542 |
| 197 | 0.241296 |
| 198 | 0.218657 |
| 199 | 0.201943 |
| 200 | 0.182838 |
| 201 | 0.165166 |
| 202 | 0.144315 |
| 203 | 0.126817 |
| 204 | 0.108253 |
| 205 | 0.090852 |
| 206 | 0.073051 |
| 207 | 0.059133 |
| 208 | 0.044645 |
| 209 | 0.03663 |
| 210 | 0.027874 |
| 211 | 0.022173 |
| 212 | 0.02185 |
| 213 | 0.020421 |
| 214 | 0.019214 |
| 215 | 0.017083 |
| 216 | 0.017474 |
| 217 | 0.017474 |
| 218 | 0.017082 |
| 219 | 0.017082 |
| 220 | 0.017082 |
| 221 | 0.016824 |
| 222 | 0.01644 |
| 223 | 0.01644 |
| 224 | 0.016373 |
| 225 | 0.016373 |
| 226 | 0.016344 |
| 227 | 0.016344 |
| 228 | 0.016344 |
| 229 | 0.016344 |
| 230 | 0.016316 |
| 231 | 0.016316 |
| 232 | 0.015767 |
| 233 | 0.015754 |
| 234 | 0.015865 |
| 235 | 0.015837 |
| 236 | 0.015781 |
| 237 | 0.015754 |
| 238 | 0.015865 |
| 239 | 0.015851 |
| 240 | 0.01627 |
| 241 | 0.016284 |
| 242 | 0.016553 |
| 243 | 0.016595 |
| 244 | 0.016595 |
| 245 | 0.016638 |
| 246 | 0.016909 |
| 247 | 0.016924 |
| 248 | 0.016341 |
| 249 | 0.016355 |
| 250 | 0.016327 |
| 251 | 0.016369 |

Cylinder 3D reduction data in Figure 13:

| x_1_ | y_1_ | z_1_ | x_2_ | y_2_ | z_2_ | x_3_ | y_3_ | z_3_ |
| --- | --- | --- | --- | --- | --- | --- | --- | --- |
| 2.021198 | 15.19396 | 16.56562 | 2.021198 | 8.682261 | 16.29455 | 2.021198 | 2.170565 | 16.39785 |
| 4.36612 | 15.19396 | 13.88099 | 4.36612 | 8.682261 | 14.03019 | 4.36612 | 2.170565 | 13.81239 |
| 6.687118 | 15.19396 | 11.97654 | 6.687118 | 8.682261 | 12.33164 | 6.687118 | 2.170565 | 12.46488 |
| 9.225551 | 15.19396 | 10.40687 | 9.225551 | 8.682261 | 10.28263 | 9.225551 | 2.170565 | 10.40283 |
| 11.78506 | 15.19396 | 8.777603 | 11.78506 | 8.682261 | 8.673699 | 11.78506 | 2.170565 | 8.634802 |
| 14.57324 | 15.19396 | 7.131543 | 14.57324 | 8.682261 | 7.06109 | 14.57324 | 2.170565 | 7.2254 |
| 17.28811 | 15.19396 | 5.771286 | 17.28811 | 8.682261 | 5.916907 | 17.28811 | 2.170565 | 6.049261 |
| 20.34224 | 15.19396 | 4.695048 | 20.34224 | 8.682261 | 5.380321 | 20.34224 | 2.170565 | 5.46266 |
| 23.19656 | 15.19396 | 4.234053 | 23.19656 | 8.682261 | 3.804799 | 23.19656 | 2.170565 | 4.285765 |
| 26.37197 | 15.19396 | 3.785971 | 26.37197 | 8.682261 | 3.410248 | 26.37197 | 2.170565 | 3.782908 |
| 29.42739 | 15.19396 | 2.633715 | 29.42739 | 8.682261 | 2.659025 | 29.42739 | 2.170565 | 2.938811 |
| 32.71622 | 15.19396 | 2.881488 | 32.71622 | 8.682261 | 2.910189 | 32.71622 | 2.170565 | 2.871872 |
| 35.93247 | 15.19396 | 2.672722 | 35.93247 | 8.682261 | 2.338292 | 35.93247 | 2.170565 | 2.618139 |
| 39.31752 | 15.19396 | 2.285715 | 39.31752 | 8.682261 | 2.275993 | 39.31752 | 2.170565 | 2.791049 |
| 42.50355 | 15.19396 | 2.313577 | 42.50355 | 8.682261 | 2.518816 | 42.50355 | 2.170565 | 2.505111 |
| 45.88223 | 15.19396 | 2.523603 | 45.88223 | 8.682261 | 2.409015 | 45.88223 | 2.170565 | 3.043987 |
| 49.08528 | 15.19396 | 2.783371 | 49.08528 | 8.682261 | 2.759433 | 49.08528 | 2.170565 | 2.725437 |
| 52.48979 | 15.19396 | 3.138754 | 52.48979 | 8.682261 | 3.412724 | 52.48979 | 2.170565 | 2.940478 |
| 55.58926 | 15.19396 | 3.48284 | 55.58926 | 8.682261 | 3.481528 | 55.58926 | 2.170565 | 3.666424 |
| 58.98903 | 15.19396 | 4.468024 | 58.98903 | 8.682261 | 4.627795 | 58.98903 | 2.170565 | 4.467702 |
| 62.05161 | 15.19396 | 5.283801 | 62.05161 | 8.682261 | 5.550757 | 62.05161 | 2.170565 | 5.482438 |
| 65.24721 | 15.19396 | 6.290654 | 65.24721 | 8.682261 | 6.205214 | 65.24721 | 2.170565 | 6.131814 |
| 68.35128 | 15.19396 | 8.182284 | 68.35128 | 8.682261 | 8.086084 | 68.35128 | 2.170565 | 7.779371 |
| 71.47713 | 15.19396 | 9.458441 | 71.47713 | 8.682261 | 9.555623 | 71.47713 | 2.170565 | 9.638424 |
| 74.45617 | 15.19396 | 11.09317 | 74.45617 | 8.682261 | 11.34926 | 74.45617 | 2.170565 | 11.24675 |
| 77.50131 | 15.19396 | 13.17722 | 77.50131 | 8.682261 | 13.48587 | 77.50131 | 2.170565 | 13.51583 |
| 80.41051 | 15.19396 | 15.76529 | 80.41051 | 8.682261 | 16.27027 | 80.41051 | 2.170565 | 15.53988 |
| 83.12565 | 15.19396 | 18.01432 | 83.12565 | 8.682261 | 17.7872 | 83.12565 | 2.170565 | 17.87931 |

Cylinder 3D reduction data in Fig. 15 (a):

| x | y | z |
| --- | --- | --- |
| -42.915 | -21.2258 | 70.07179 |
| -43.0772 | -20.9468 | 71.79397 |
| -43.1851 | -20.6415 | 72.934 |
| -35.4325 | -19.2441 | 38.8682 |
| -42.9721 | -20.1834 | 70.32273 |
| 50.03751 | -20.7738 | 84.96731 |
| -43.0205 | -19.8488 | 70.73888 |
| -35.1977 | -18.8904 | 46.54687 |
| -43.1239 | -19.5406 | 71.8451 |
| -34.9239 | -18.4796 | 44.58051 |
| -43.0521 | -19.1523 | 70.89693 |
| -34.8518 | -18.1256 | 44.03109 |
| -43.1988 | -18.8621 | 72.5302 |
| -34.795 | -17.7434 | 42.7097 |
| -43.3274 | -18.5604 | 73.91136 |
| -34.939 | -17.4788 | 44.53027 |
| -43.2808 | -18.1835 | 73.26566 |
| -34.9833 | -17.1439 | 44.47524 |
| -43.2261 | -17.8072 | 72.60818 |
| -35.0097 | -16.8199 | 44.72703 |
| -31.2415 | -16.3642 | 31.85595 |
| 46.61192 | -16.6975 | 41.26939 |
| -43.2433 | -17.4582 | 72.71406 |
| -35.0206 | -16.488 | 44.76132 |
| -31.8142 | -16.3694 | 41.34201 |
| 47.32002 | -16.6548 | 49.56588 |
| -43.3618 | -17.1445 | 73.85846 |
| -35.0201 | -16.1305 | 44.04107 |
| -31.6629 | -15.9633 | 39.12656 |
| -43.3895 | -16.7987 | 74.09107 |
| -35.0297 | -15.8085 | 44.35912 |
| -31.6944 | -15.6297 | 38.99189 |
| -43.3499 | -16.4266 | 73.52493 |
| -35.1125 | -15.5033 | 45.20804 |
| -31.6636 | -15.2728 | 38.13858 |
| 46.08291 | -15.9968 | 60.34139 |
| -43.4583 | -16.1049 | 74.51248 |
| -35.1575 | -15.181 | 45.55453 |
| -31.711 | -14.9566 | 38.52024 |
| -28.4789 | -14.9846 | 39.39896 |
| 45.86542 | -15.7197 | 62.44148 |
| -43.5292 | -15.774 | 75.25276 |
| -35.1459 | -14.8348 | 45.15335 |
| -31.7233 | -14.6257 | 38.45255 |
| -28.2208 | -14.5161 | 34.93974 |
| 48.99084 | -15.6155 | 70.17153 |
| -43.4902 | -15.403 | 74.71201 |
| -35.2033 | -14.5192 | 45.7338 |
| -31.8464 | -14.3459 | 40.05325 |
| -28.3174 | -14.2276 | 36.17539 |
| 44.26757 | -14.9028 | 58.31101 |
| 49.1935 | -15.3444 | 72.78806 |
| -43.4058 | -15.0185 | 73.6916 |
| -35.1837 | -14.1705 | 45.23366 |
| -31.8871 | -14.026 | 40.38573 |
| -28.4299 | -13.9347 | 37.32194 |
| 43.40014 | -14.5559 | 58.16774 |
| 49.27061 | -15.0349 | 74.23992 |
| -43.4688 | -14.6882 | 74.4851 |
| -35.2022 | -13.8394 | 45.31275 |
| -31.892 | -13.6916 | 40.23369 |
| -28.3277 | -13.5672 | 35.96041 |
| -24.2816 | -13.4865 | 33.18541 |
| 42.34678 | -14.1034 | 54.38611 |
| 49.32757 | -14.6982 | 74.82682 |
| -43.4578 | -14.327 | 74.22858 |
| -35.2277 | -13.5078 | 45.37875 |
| -31.8822 | -13.3495 | 39.80535 |
| -28.3746 | -13.2382 | 35.88597 |
| -24.2009 | -13.1284 | 32.01997 |
| 49.17642 | -14.3215 | 74.03491 |
| -43.4932 | -13.9837 | 74.60334 |
| -35.2522 | -13.1752 | 45.41337 |
| -31.8927 | -13.0173 | 39.71434 |
| -28.343 | -12.9035 | 35.60512 |
| -24.2324 | -12.8078 | 32.14983 |
| 49.24067 | -13.9756 | 74.31328 |
| -43.5723 | -13.6521 | 75.43396 |
| -35.2245 | -12.8359 | 45.19976 |
| -31.9436 | -12.7008 | 40.19456 |
| -28.4174 | -12.601 | 36.49764 |
| -24.2599 | -12.4867 | 32.26376 |
| -20.1024 | -12.4486 | 30.85472 |
| 39.45355 | -12.5878 | 36.01182 |
| 49.28734 | -13.6536 | 75.48987 |
| -43.7173 | -13.339 | 77.01322 |
| -35.3006 | -12.522 | 45.94286 |
| -32.0121 | -12.3919 | 40.99489 |
| -28.4669 | -12.2873 | 37.01571 |
| -24.2678 | -12.1557 | 32.01099 |
| -19.9707 | -12.052 | 28.06848 |
| 39.53953 | -12.3037 | 37.64018 |
| 49.38665 | -13.3363 | 76.91036 |
| -43.7834 | -12.9995 | 77.64643 |
| -35.3586 | -12.2021 | 46.48936 |
| -31.9879 | -12.0468 | 40.4239 |
| -28.4687 | -11.9419 | 36.32262 |
| -24.2732 | -11.824 | 31.71829 |
| -20.0157 | -11.7456 | 28.65233 |
| 40.01603 | -12.1262 | 43.52414 |
| 49.23588 | -12.9494 | 75.68882 |
| -43.7934 | -12.6428 | 77.62632 |
| -35.3787 | -11.869 | 46.5377 |
| -31.9754 | -11.7068 | 40.02069 |
| -28.5125 | -11.6388 | 37.28905 |
| -24.3064 | -11.5074 | 32.01373 |
| -20.0422 | -11.4327 | 29.01234 |
| -15.3319 | -11.3636 | 26.23543 |
| 40.12392 | -11.8351 | 45.17856 |
| 49.26723 | -12.6116 | 76.3732 |
| -43.7669 | -12.2721 | 77.02154 |
| -35.4449 | -11.5495 | 47.15173 |
| -32.0266 | -11.3894 | 40.53316 |
| -28.5469 | -11.3048 | 37.0374 |
| -24.3669 | -11.2025 | 32.80928 |
| -20.0579 | -11.1138 | 29.14286 |
| -15.3108 | -11.0321 | 25.76425 |
| 36.64197 | -11.3627 | 39.43174 |
| 40.10497 | -11.5033 | 45.24178 |
| 49.30552 | -12.2765 | 77.20284 |
| -43.6575 | -11.8821 | 75.56186 |
| -35.4974 | -11.2258 | 47.62459 |
| -32.078 | -11.0708 | 41.02561 |
| -28.5599 | -10.9896 | 37.56866 |
| -24.4197 | -10.8931 | 33.45889 |
| -20.0832 | -10.7826 | 28.7556 |
| -15.3677 | -10.7326 | 26.62777 |
| -10.7771 | -10.814 | 30.09031 |
| 33.0014 | -10.966 | 36.56435 |
| 36.59308 | -11.0272 | 39.17 |
| 40.00935 | -11.1482 | 44.32097 |
| 49.18824 | -11.894 | 76.07086 |
| -43.7568 | -11.5511 | 76.60428 |
| -35.3875 | -10.8515 | 45.9018 |
| -32.0675 | -10.7315 | 40.6366 |
| -28.6021 | -10.6585 | 37.43364 |
| -24.4312 | -10.5654 | 33.34955 |
| -20.1398 | -10.469 | 29.11958 |
| -15.3778 | -10.403 | 26.22069 |
| -10.6454 | -10.3711 | 24.8237 |
| 32.90397 | -10.6119 | 35.38675 |
| 36.61557 | -10.7191 | 40.09255 |
| 40.03569 | -10.8282 | 44.88182 |
| 49.18885 | -11.5575 | 76.88466 |
| -43.7996 | -11.2044 | 76.9994 |
| -35.4319 | -10.524 | 46.1937 |
| -32.1347 | -10.4168 | 41.34122 |
| -28.577 | -10.3286 | 37.34745 |
| -24.4792 | -10.2524 | 33.89581 |
| -20.1137 | -10.1438 | 28.97759 |
| -15.405 | -10.0864 | 26.37842 |
| -10.6794 | -10.0379 | 24.18524 |
| -5.63507 | -10.0671 | 25.50552 |
| 32.95231 | -10.3068 | 36.35757 |
| 36.6289 | -10.387 | 39.99107 |
| 40.03313 | -10.4984 | 45.03129 |
| 49.23959 | -11.2091 | 77.21048 |
| -43.6865 | -10.8189 | 75.602 |
| -35.4218 | -10.1912 | 46.25188 |
| -32.1072 | -10.0728 | 40.71525 |
| -28.6097 | -9.99537 | 37.09605 |
| -24.4813 | -9.91919 | 33.53404 |
| -20.1735 | -9.82959 | 29.34457 |
| -15.4301 | -9.76883 | 26.50348 |
| -10.7036 | -9.72987 | 24.68162 |
| -5.59483 | -9.6919 | 22.90625 |
| 28.6475 | -9.89508 | 32.40668 |
| 32.87161 | -9.96241 | 35.55502 |
| 36.61725 | -10.066 | 40.40076 |
| 40.12089 | -10.1921 | 46.29558 |
| 49.29344 | -10.8681 | 77.90252 |
| -43.7988 | -10.4887 | 76.78946 |
| -35.4529 | -9.86146 | 46.4657 |
| -32.1447 | -9.74964 | 41.05966 |
| -28.5567 | -9.657 | 36.58116 |
| -24.4668 | -9.57998 | 32.85785 |
| -20.2017 | -9.51577 | 29.75351 |
| -15.4672 | -9.45733 | 26.92825 |
| -10.7196 | -9.41568 | 24.91452 |
| -5.60875 | -9.38558 | 23.45939 |
| -0.64789 | -9.45138 | 26.64074 |
| 24.43392 | -9.5387 | 30.86214 |
| 28.66779 | -9.56959 | 32.35546 |
| 32.83333 | -9.63157 | 35.35148 |
| 36.54418 | -9.72679 | 39.95489 |
| 40.08973 | -9.85654 | 46.22762 |
| 49.34411 | -10.5319 | 78.87699 |
| -43.9605 | -10.1676 | 78.51543 |
| -35.5142 | -9.5384 | 47.02758 |
| -32.1731 | -9.42365 | 41.28535 |
| -28.628 | -9.3369 | 36.9444 |
| -24.4785 | -9.25164 | 32.67754 |
| -20.2342 | -9.20145 | 30.16619 |
| -15.4882 | -9.13695 | 26.93873 |
| -10.7528 | -9.09267 | 24.72301 |
| -5.63311 | -9.05925 | 23.05047 |
| -0.62465 | -9.05526 | 22.85087 |
| 4.662766 | -9.11698 | 25.93932 |
| 19.59721 | -9.19962 | 30.07443 |
| 24.35485 | -9.19205 | 29.69587 |
| 28.65883 | -9.25711 | 32.95161 |
| 32.82505 | -9.31022 | 35.60909 |
| 36.50459 | -9.39656 | 39.92942 |
| 39.97156 | -9.50086 | 45.14903 |
| 49.25039 | -10.159 | 78.08174 |
| -44.3332 | -9.89199 | 82.72126 |
| -35.6097 | -9.21491 | 47.60756 |
| -32.2116 | -9.09999 | 41.6478 |
| -28.6558 | -9.01285 | 37.12908 |
| -24.5002 | -8.92769 | 32.7124 |
| -20.3215 | -8.89723 | 31.13263 |
| -15.5151 | -8.81963 | 27.10821 |
| -10.7807 | -8.79134 | 25.64123 |
| -5.66644 | -8.75466 | 23.7391 |
| -0.66017 | -8.7344 | 22.68827 |
| 4.62239 | -8.72989 | 22.4543 |
| 9.579721 | -8.74976 | 23.48468 |
| 14.7265 | -8.7618 | 24.10931 |
| 19.46721 | -8.82338 | 27.30302 |
| 24.27334 | -8.85066 | 28.71746 |
| 28.6103 | -8.93059 | 32.86302 |
| 32.80591 | -8.98514 | 35.69187 |
| 36.5297 | -9.07454 | 40.32788 |
| 39.98594 | -9.17672 | 45.62745 |
| 49.09356 | -9.78772 | 77.31358 |
| -43.9826 | -9.45377 | 78.49584 |
| -35.5828 | -8.87721 | 47.46725 |
| -32.2571 | -8.77694 | 42.0707 |
| -28.7107 | -8.69708 | 37.77309 |
| -24.5265 | -8.60506 | 32.82094 |
| -20.3122 | -8.56567 | 30.70098 |
| -15.5413 | -8.50079 | 27.20906 |
| -10.8249 | -8.47772 | 25.96744 |
| -5.69098 | -8.43911 | 23.88961 |
| -0.70313 | -8.42914 | 23.35348 |
| 4.599853 | -8.40959 | 22.30091 |
| 9.553568 | -8.43979 | 23.92641 |
| 14.72816 | -8.45508 | 24.74943 |
| 19.4369 | -8.49345 | 26.81416 |
| 24.26967 | -8.53913 | 29.2728 |
| 28.58754 | -8.59109 | 32.06902 |
| 32.8237 | -8.67015 | 36.32357 |
| 36.52986 | -8.74578 | 40.39391 |
| 39.9356 | -8.83873 | 45.39618 |
| 49.073 | -9.42301 | 76.84059 |
| -44.1758 | -9.13494 | 80.6167 |
| -35.5798 | -8.5296 | 46.76112 |
| -32.2404 | -8.43757 | 41.61444 |
| -28.7125 | -8.36526 | 37.56993 |
| -24.5775 | -8.29144 | 33.44153 |
| -20.2912 | -8.22994 | 30.0019 |
| -15.5788 | -8.18828 | 27.67205 |
| -10.8507 | -8.14842 | 25.44269 |
| -5.71106 | -8.1178 | 23.73015 |
| -0.74401 | -8.11477 | 23.56081 |
| 4.566738 | -8.09179 | 22.2757 |
| 9.513481 | -8.11972 | 23.83758 |
| 14.68499 | -8.12789 | 24.29451 |
| 19.39366 | -8.17321 | 26.82933 |
| 24.26142 | -8.22462 | 29.70434 |
| 28.6245 | -8.28865 | 33.28559 |
| 32.80863 | -8.34586 | 36.48511 |
| 36.45875 | -8.41396 | 40.29382 |
| 39.91704 | -8.5085 | 45.58129 |
| 48.9164 | -9.04625 | 75.65614 |
| -43.8089 | -8.70228 | 76.28416 |
| -35.6341 | -8.2101 | 47.63423 |
| -32.2541 | -8.10645 | 41.60067 |
| -28.7384 | -8.03998 | 37.73139 |
| -24.6409 | -7.98102 | 34.299 |
| -20.3203 | -7.90098 | 29.64035 |
| -15.6107 | -7.87126 | 27.91 |
| -10.8679 | -7.83436 | 25.76193 |
| -5.74224 | -7.80698 | 24.16847 |
| -0.74883 | -7.7876 | 23.04038 |
| 4.548771 | -7.78773 | 23.04772 |
| 9.505687 | -7.80015 | 23.77071 |
| 14.68272 | -7.82318 | 25.11116 |
| 19.40832 | -7.84953 | 26.64524 |
| 24.21735 | -7.89696 | 29.40602 |
| 28.6056 | -7.96772 | 33.52496 |
| 32.83225 | -8.03107 | 37.21259 |
| 36.50567 | -8.09525 | 40.94851 |
| 39.84389 | -8.1663 | 45.08462 |
| 48.93538 | -8.7026 | 76.30298 |
| -44.008 | -8.38261 | 78.44013 |
| -35.6179 | -7.8748 | 47.62232 |
| -32.2457 | -7.77086 | 41.31446 |
| -28.7619 | -7.71373 | 37.84727 |
| -24.6491 | -7.65171 | 34.08341 |
| -20.3203 | -7.58564 | 30.0742 |
| -15.605 | -7.53606 | 27.06483 |
| -10.8831 | -7.51613 | 25.85558 |
| -5.75414 | -7.47418 | 23.30958 |
| -0.75474 | -7.46952 | 23.02684 |
| 4.523326 | -7.47589 | 23.41325 |
| 9.49992 | -7.48307 | 23.84896 |
| 14.64522 | -7.49798 | 24.7542 |
| 19.39097 | -7.53848 | 27.21219 |
| 24.205 | -7.57996 | 29.7294 |
| 28.55075 | -7.63712 | 33.19836 |
| 32.80595 | -7.70448 | 37.28576 |
| 36.4962 | -7.77689 | 41.68033 |
| 39.8284 | -7.83671 | 45.31084 |
| 49.03451 | -8.36551 | 77.40208 |
| -43.9151 | -8.00695 | 77.23899 |
| -35.7051 | -7.54691 | 48.07899 |
| -32.3261 | -7.45546 | 42.28262 |
| -28.7542 | -7.37974 | 37.48323 |
| -24.6708 | -7.3266 | 34.11513 |
| -20.3778 | -7.26769 | 30.38095 |
| -15.6443 | -7.22268 | 27.52765 |
| -10.9333 | -7.20002 | 26.09163 |
| -5.78353 | -7.16142 | 23.64515 |
| -0.79331 | -7.15267 | 23.09031 |
| 4.497179 | -7.15801 | 23.42874 |
| 9.460719 | -7.16452 | 23.84155 |
| 14.63967 | -7.18906 | 25.39712 |
| 19.35972 | -7.22014 | 27.3671 |
| 24.16826 | -7.25557 | 29.61264 |
| 28.50372 | -7.3097 | 33.0438 |
| 32.69836 | -7.35924 | 36.18395 |
| 36.50856 | -7.45563 | 42.29332 |
| 39.8127 | -7.50583 | 45.47564 |
| 48.94802 | -8.00195 | 76.92186 |
| -44.0577 | -7.67525 | 78.84194 |
| -35.7189 | -7.21066 | 48.02397 |
| -32.3225 | -7.11976 | 41.99442 |
| -28.8092 | -7.05295 | 37.56285 |
| -24.6656 | -6.99391 | 33.64652 |
| -20.4513 | -6.95538 | 31.09059 |
| -15.6815 | -6.90777 | 27.93263 |
| -10.9076 | -6.8764 | 25.85184 |
| -5.81473 | -6.85071 | 24.14717 |
| -0.83306 | -6.84425 | 23.71909 |
| 4.46946 | -6.83626 | 23.18892 |
| 9.421377 | -6.84508 | 23.7741 |
| 14.62654 | -6.87583 | 25.81362 |
| 19.29815 | -6.89091 | 26.81381 |
| 24.12376 | -6.92776 | 29.25816 |
| 28.49057 | -6.99085 | 33.44367 |
| 32.68365 | -7.03516 | 36.38276 |
| 36.47125 | -7.12418 | 42.28736 |
| 39.79892 | -7.17559 | 45.69761 |
| 48.95274 | -7.65861 | 77.73778 |
| -43.9606 | -7.30024 | 77.58778 |
| -35.7268 | -6.87396 | 47.93226 |
| -32.3147 | -6.78358 | 41.64446 |
| -28.8128 | -6.72978 | 37.90209 |
| -24.7227 | -6.67894 | 34.36463 |
| -20.4667 | -6.63292 | 31.16321 |
| -15.7045 | -6.58684 | 27.95749 |
| -10.9474 | -6.55761 | 25.92413 |
| -5.83014 | -6.52468 | 23.6329 |
| -0.83939 | -6.52477 | 23.63921 |
| 4.441241 | -6.51648 | 23.06264 |
| 9.387358 | -6.53057 | 24.04292 |
| 14.60548 | -6.55877 | 26.00486 |
| 19.27007 | -6.5627 | 26.27839 |
| 24.10993 | -6.60961 | 29.54164 |
| 28.47297 | -6.67034 | 33.76653 |
| 32.69327 | -6.71573 | 36.92401 |
| 36.42917 | -6.7854 | 41.77119 |
| 39.80072 | -6.84759 | 46.09739 |
| 48.82377 | -7.27992 | 76.17442 |
| -44.0927 | -6.96426 | 79.06027 |
| -35.6847 | -6.53365 | 47.56705 |
| -32.3811 | -6.46267 | 42.37563 |
| -28.8435 | -6.4046 | 38.12832 |
| -24.7782 | -6.36151 | 34.97693 |
| -20.4944 | -6.31492 | 31.57 |
| -15.7155 | -6.26215 | 27.71034 |
| -10.9887 | -6.23762 | 25.91667 |
| -5.86064 | -6.2136 | 24.15936 |
| -0.84484 | -6.20679 | 23.66162 |
| 4.408514 | -6.1919 | 22.57228 |
| 9.387285 | -6.21579 | 24.31986 |
| 14.55733 | -6.22977 | 25.3421 |
| 19.29569 | -6.25178 | 26.95159 |
| 24.10677 | -6.29364 | 30.01359 |
| 28.46152 | -6.34349 | 33.659 |
| 32.68742 | -6.39291 | 37.2735 |
| 36.34448 | -6.44609 | 41.16307 |
| 39.74878 | -6.51109 | 45.91672 |
| 48.79832 | -6.92572 | 76.24133 |
| -44.2348 | -6.62707 | 80.59796 |
| -35.6722 | -6.18767 | 46.72478 |
| -32.4206 | -6.13629 | 42.76428 |
| -28.8924 | -6.08354 | 38.69778 |
| -24.7664 | -6.02786 | 34.40535 |
| -20.5077 | -5.99129 | 31.5863 |
| -15.7577 | -5.94785 | 28.23721 |
| -11.0259 | -5.91548 | 25.7417 |
| -5.89167 | -5.90039 | 24.57898 |
| -0.88116 | -5.88724 | 23.56509 |
| 4.394003 | -5.88625 | 23.48885 |
| 9.369015 | -5.90756 | 25.13165 |
| 14.52422 | -5.90956 | 25.28577 |
| 19.25604 | -5.9392 | 27.57072 |
| 24.09939 | -5.97671 | 30.46242 |
| 28.4147 | -6.0095 | 32.99012 |
| 32.68883 | -6.07095 | 37.72678 |
| 36.28989 | -6.11284 | 40.95608 |
| 39.69281 | -6.17308 | 45.60025 |
| 48.81356 | -6.57835 | 76.84214 |
| -44.1811 | -6.25882 | 79.78079 |
| -35.8143 | -5.87891 | 48.81988 |
| -32.4633 | -5.80907 | 43.12885 |
| -28.9518 | -5.76368 | 39.42973 |
| -24.7914 | -5.70363 | 34.53529 |
| -20.514 | -5.66596 | 31.46567 |
| -15.8051 | -5.63596 | 29.02106 |
| -11.0536 | -5.59077 | 25.33817 |
| -5.91776 | -5.58351 | 24.74668 |
| -0.92077 | -5.57976 | 24.44059 |
| 4.368915 | -5.56904 | 23.56728 |
| 9.351724 | -5.58143 | 24.57696 |
| 14.52483 | -5.60035 | 26.11877 |
| 19.21577 | -5.61651 | 27.4357 |
| 24.12657 | -5.66666 | 31.52246 |
| 28.37763 | -5.68452 | 32.97827 |
| 32.6178 | -5.73581 | 37.1585 |
| 36.28872 | -5.78779 | 41.39412 |
| 39.69274 | -5.84411 | 45.98394 |
| 48.64161 | -6.20636 | 75.50585 |
| -43.9768 | -5.87295 | 77.34166 |
| -35.8878 | -5.55171 | 49.57502 |
| -32.5151 | -5.48328 | 43.66094 |
| -28.9856 | -5.43153 | 39.18727 |
| -24.7916 | -5.37332 | 34.15674 |
| -20.554 | -5.34114 | 31.37533 |
| -15.8231 | -5.31284 | 28.9289 |
| -11.0484 | -5.27988 | 26.07962 |
| -5.94559 | -5.26732 | 24.99462 |
| -0.95934 | -5.26481 | 24.77709 |
| 4.3488 | -5.25785 | 24.17586 |
| 9.308158 | -5.25892 | 24.26857 |
| 14.50195 | -5.28135 | 26.20735 |
| 19.21121 | -5.29512 | 27.39707 |
| 24.05754 | -5.33432 | 30.78507 |
| 28.35551 | -5.36267 | 33.23565 |
| 32.57422 | -5.40565 | 36.95118 |
| 36.30287 | -5.46391 | 41.98662 |
| 39.65883 | -5.51011 | 45.97976 |
| 48.75115 | -5.86835 | 76.94404 |
| -44.3225 | -5.5594 | 81.24143 |
| -35.8577 | -5.2082 | 48.92751 |
| -32.5438 | -5.15238 | 43.79114 |
| -29.0042 | -5.10314 | 39.26044 |
| -24.8412 | -5.05447 | 34.78292 |
| -20.5794 | -5.02061 | 31.66678 |
| -15.8378 | -4.9882 | 28.68543 |
| -11.0959 | -4.96406 | 26.46378 |
| -5.97319 | -4.95019 | 25.1875 |
| -0.99824 | -4.94099 | 24.34127 |
| 4.321644 | -4.93607 | 23.88832 |
| 9.283782 | -4.94628 | 24.82798 |
| 14.46343 | -4.95792 | 25.8994 |
| 19.17581 | -4.97442 | 27.41715 |
| 23.9969 | -5.00511 | 30.24082 |
| 28.32947 | -5.03885 | 33.34528 |
| 32.55709 | -5.08047 | 37.1746 |
| 36.23635 | -5.12389 | 41.17006 |
| 39.61773 | -5.1752 | 45.89102 |
| 48.96039 | -5.54028 | 79.48216 |
| -44.2563 | -5.19218 | 80.4006 |
| -35.8386 | -4.86676 | 48.39344 |
| -32.5225 | -4.81425 | 43.22902 |
| -29.0434 | -4.77775 | 39.63903 |
| -24.8691 | -4.73053 | 34.99501 |
| -20.6126 | -4.69418 | 31.4191 |
| -15.8566 | -4.66562 | 28.61033 |
| -11.1385 | -4.64529 | 26.61081 |
| -5.99003 | -4.62209 | 24.32926 |
| -1.00558 | -4.61694 | 23.82246 |
| 4.29728 | -4.62102 | 24.22354 |
| 9.271119 | -4.63871 | 25.96398 |
| 14.44138 | -4.63991 | 26.08143 |
| 19.14183 | -4.65356 | 27.42427 |
| 24.01072 | -4.69105 | 31.1115 |
| 28.31378 | -4.71698 | 33.66142 |
| 32.58665 | -4.76157 | 38.04721 |
| 36.21179 | -4.79455 | 41.2911 |
| 39.57903 | -4.84051 | 45.81132 |
| 48.88349 | -5.1761 | 78.81881 |
| -44.3247 | -4.84067 | 81.09462 |
| -35.8446 | -4.5296 | 48.23296 |
| -32.5402 | -4.48245 | 43.25138 |
| -29.0648 | -4.44901 | 39.71962 |
| -24.9036 | -4.40695 | 35.27563 |
| -20.6269 | -4.37135 | 31.51536 |
| -15.8718 | -4.34221 | 28.43716 |
| -11.1885 | -4.32846 | 26.98359 |
| -6.01106 | -4.30056 | 24.0365 |
| -1.01277 | -4.2965 | 23.60792 |
| 4.270944 | -4.30549 | 24.55801 |
| 9.261418 | -4.31583 | 25.64962 |
| 14.40045 | -4.31664 | 25.73594 |
| 19.12288 | -4.33639 | 27.82172 |
| 23.95363 | -4.36223 | 30.55182 |
| 28.28958 | -4.3933 | 33.83426 |
| 32.56326 | -4.43432 | 38.16727 |
| 36.23057 | -4.4701 | 41.947 |
| 39.5281 | -4.50505 | 45.63911 |
| 48.78803 | -4.81128 | 77.98948 |
| -44.1418 | -4.46256 | 78.86546 |
| -35.8707 | -4.19871 | 48.76183 |
| -32.5557 | -4.15057 | 43.26875 |
| -29.0374 | -4.11296 | 38.97874 |
| -24.9139 | -4.07907 | 35.11118 |
| -20.6662 | -4.05325 | 32.16565 |
| -15.9247 | -4.02823 | 29.31168 |
| -11.1506 | -4.00246 | 26.37155 |
| -6.03464 | -3.9829 | 24.13956 |
| -1.05429 | -3.98847 | 24.7752 |
| 4.241125 | -3.98311 | 24.1634 |
| 9.210723 | -3.99024 | 24.97721 |
| 14.38173 | -3.9999 | 26.07876 |
| 19.08858 | -4.01541 | 27.84832 |
| 23.95023 | -4.04351 | 31.0549 |
| 28.275 | -4.07077 | 34.16433 |
| 32.50095 | -4.10189 | 37.71502 |
| 36.17526 | -4.1368 | 41.69787 |
| 39.53136 | -4.17551 | 46.11527 |
| 48.58823 | -4.44136 | 76.4461 |
| -44.3904 | -4.12709 | 81.53596 |
| -35.8503 | -3.85537 | 47.8381 |
| -32.6031 | -3.82236 | 43.7449 |
| -29.0881 | -3.78812 | 39.49923 |
| -24.9401 | -3.75347 | 35.20209 |
| -20.7144 | -3.72966 | 32.24893 |
| -15.9709 | -3.71113 | 29.95155 |
| -11.1952 | -3.6832 | 26.48673 |
| -6.06109 | -3.6669 | 24.46606 |
| -1.09182 | -3.66774 | 24.5703 |
| 4.215004 | -3.66632 | 24.39428 |
| 9.16223 | -3.66671 | 24.44283 |
| 14.3532 | -3.67964 | 26.04554 |
| 19.0909 | -3.69488 | 27.9353 |
| 23.92747 | -3.721 | 31.17541 |
| 28.2573 | -3.74729 | 34.43571 |
| 32.45155 | -3.77157 | 37.44638 |
| 36.23727 | -3.81612 | 42.97111 |
| 39.55236 | -3.84688 | 46.78612 |
| 48.46872 | -4.07717 | 75.34445 |
| -44.1464 | -3.74669 | 78.61297 |
| -35.899 | -3.52325 | 48.26344 |
| -32.6255 | -3.49055 | 43.82231 |
| -29.1124 | -3.45945 | 39.59759 |
| -24.9513 | -3.42591 | 35.04241 |
| -20.6702 | -3.40148 | 31.72467 |
| -16.009 | -3.39152 | 30.37104 |
| -11.2147 | -3.36589 | 26.88989 |
| -6.08595 | -3.3488 | 24.56856 |
| -1.09547 | -3.34955 | 24.67116 |
| 4.190113 | -3.34921 | 24.62424 |
| 9.129779 | -3.35032 | 24.77568 |
| 14.32527 | -3.35934 | 26.00057 |
| 19.03825 | -3.37579 | 28.2346 |
| 23.91615 | -3.4 | 31.52336 |
| 28.25756 | -3.42548 | 34.98449 |
| 32.45685 | -3.44748 | 37.97223 |
| 36.14698 | -3.47861 | 42.20084 |
| 39.57496 | -3.51726 | 47.45041 |
| 48.21223 | -3.70576 | 73.05312 |
| -44.2242 | -3.39433 | 79.28331 |
| -35.9687 | -3.19267 | 49.00975 |
| -32.6206 | -3.15613 | 43.52412 |
| -29.1062 | -3.13103 | 39.75592 |
| -24.9804 | -3.10112 | 35.2665 |
| -20.7709 | -3.08623 | 33.03061 |
| -16.0292 | -3.06823 | 30.32834 |
| -11.261 | -3.04664 | 27.08733 |
| -6.11883 | -3.03411 | 25.20689 |
| -1.13641 | -3.03319 | 25.06816 |
| 4.157183 | -3.02669 | 24.09279 |
| 9.098045 | -3.03226 | 24.92872 |
| 14.31876 | -3.04336 | 26.59406 |
| 19.04757 | -3.05617 | 28.51717 |
| 23.8753 | -3.07475 | 31.30768 |
| 28.17167 | -3.09346 | 34.11531 |
| 32.45349 | -3.12182 | 38.37397 |
| 36.13339 | -3.14914 | 42.47471 |
| 39.52122 | -3.18058 | 47.19516 |
| 48.25223 | -3.35743 | 73.74407 |
| -44.3326 | -3.0431 | 80.30078 |
| -36.024 | -2.8597 | 49.5296 |
| -32.575 | -2.81864 | 42.64063 |
| -29.1618 | -2.80214 | 39.87267 |
| -24.9828 | -2.77314 | 35.00568 |
| -20.8319 | -2.7637 | 33.42322 |
| -16.0219 | -2.74049 | 29.52904 |
| -11.2718 | -2.72628 | 27.14477 |
| -6.14435 | -2.71424 | 25.12421 |
| -1.17344 | -2.71657 | 25.51452 |
| 4.1319 | -2.70829 | 24.12487 |
| 9.093804 | -2.71327 | 24.96095 |
| 14.32053 | -2.72917 | 27.62965 |
| 19.02682 | -2.73622 | 28.81262 |
| 23.89944 | -2.75745 | 32.37405 |
| 28.22458 | -2.77297 | 34.97824 |
| 32.42255 | -2.79315 | 38.36446 |
| 36.13707 | -2.82032 | 42.92211 |
| 39.48113 | -2.8449 | 47.04701 |
| 48.22645 | -3.00603 | 74.0817 |
| -44.4629 | -2.69174 | 81.56682 |
| -36.0168 | -2.52162 | 49.21633 |
| -32.6422 | -2.49106 | 43.40491 |
| -29.1497 | -2.47298 | 39.96709 |
| -25.0621 | -2.45301 | 36.17095 |
| -20.7683 | -2.43379 | 32.51533 |
| -16.0738 | -2.42262 | 30.3919 |
| -11.2976 | -2.40875 | 27.7534 |
| -6.18092 | -2.39925 | 25.94851 |
| -1.21131 | -2.39664 | 25.45068 |
| 4.105208 | -2.38964 | 24.121 |
| 9.065512 | -2.39592 | 25.3141 |
| 14.28882 | -2.40724 | 27.46632 |
| 18.99266 | -2.41436 | 28.82127 |
| 23.80889 | -2.42716 | 31.25498 |
| 28.23504 | -2.45056 | 35.70478 |
| 32.48323 | -2.47148 | 39.68248 |
| 36.0742 | -2.48658 | 42.55303 |
| 39.55003 | -2.51653 | 48.24905 |
| 48.16954 | -2.64935 | 73.50619 |
| -44.1015 | -2.31335 | 77.29049 |
| -36.0903 | -2.1888 | 49.96285 |
| -32.6893 | -2.16091 | 43.84307 |
| -29.2481 | -2.14682 | 40.75153 |
| -25.093 | -2.12681 | 36.36111 |
| -20.8092 | -2.10912 | 32.48034 |
| -16.0634 | -2.09562 | 29.51647 |
| -11.353 | -2.08974 | 28.22843 |
| -6.20615 | -2.08027 | 26.14953 |
| -1.21695 | -2.07546 | 25.0933 |
| 4.082992 | -2.07298 | 24.55033 |
| 9.07748 | -2.08017 | 26.12737 |
| 14.26752 | -2.08734 | 27.69988 |
| 18.95287 | -2.09185 | 28.68947 |
| 23.80578 | -2.10587 | 31.76691 |
| 28.1766 | -2.12188 | 35.27938 |
| 32.42362 | -2.14029 | 39.31806 |
| 36.02339 | -2.15408 | 42.34472 |
| 39.5467 | -2.18239 | 48.55673 |
| 48.11223 | -2.29368 | 72.97446 |
| -44.1724 | -1.96033 | 78.03673 |
| -36.1539 | -1.8544 | 50.56808 |
| -32.7209 | -1.82936 | 44.07659 |
| -29.2215 | -1.81609 | 40.63357 |
| -25.068 | -1.79661 | 35.58347 |
| -20.893 | -1.78786 | 33.31528 |
| -16.0937 | -1.77411 | 29.75014 |
| -11.3489 | -1.76644 | 27.75986 |
| -6.23527 | -1.76156 | 26.49397 |
| -1.25497 | -1.75487 | 24.76014 |
| 4.066243 | -1.75773 | 25.50232 |
| 9.046944 | -1.76115 | 26.38801 |
| 14.23014 | -1.76554 | 27.52637 |
| 18.94006 | -1.7721 | 29.22696 |
| 23.79675 | -1.78324 | 32.11533 |
| 28.12964 | -1.79467 | 35.07937 |
| 32.34042 | -1.80809 | 38.56078 |
| 35.995 | -1.82306 | 42.44242 |
| 39.48496 | -1.84532 | 48.21475 |
| 48.10775 | -1.94243 | 73.39545 |
| -44.1749 | -1.60376 | 77.98836 |
| -36.1914 | -1.51813 | 50.85148 |
| -32.7721 | -1.49834 | 44.57928 |
| -29.2361 | -1.48413 | 40.07607 |
| -25.1301 | -1.47251 | 36.39332 |
| -20.9058 | -1.46508 | 34.03846 |
| -16.1251 | -1.45235 | 30.00465 |
| -11.3743 | -1.44341 | 27.17007 |
| -6.25684 | -1.44055 | 26.26433 |
| -1.26432 | -1.43814 | 25.50077 |
| 4.041661 | -1.43832 | 25.55691 |
| 9.006757 | -1.44002 | 26.096 |
| 14.18951 | -1.44353 | 27.21013 |
| 18.90586 | -1.44991 | 29.23138 |
| 23.78897 | -1.46032 | 32.53017 |
| 28.15135 | -1.47141 | 36.04514 |
| 32.37104 | -1.48217 | 39.45542 |
| 35.99464 | -1.49299 | 42.88297 |
| 39.42245 | -1.50859 | 47.8278 |
| 48.10222 | -1.59037 | 73.74564 |
| -44.1493 | -1.2466 | 77.67814 |
| -36.2532 | -1.18219 | 51.43028 |
| -32.793 | -1.16559 | 44.6654 |
| -29.2753 | -1.15648 | 40.95665 |
| -25.1957 | -1.14737 | 37.24186 |
| -20.9466 | -1.13919 | 33.91109 |
| -16.1655 | -1.13101 | 30.57744 |
| -11.3984 | -1.12425 | 27.82125 |
| -6.28874 | -1.12201 | 26.90944 |
| -1.30362 | -1.11988 | 26.04114 |
| 4.010575 | -1.11791 | 25.23961 |
| 8.97983 | -1.12131 | 26.62245 |
| 14.18172 | -1.12434 | 27.85719 |
| 18.88499 | -1.12852 | 29.56246 |
| 23.71997 | -1.134 | 31.79632 |
| 28.05335 | -1.1419 | 35.01464 |
| 32.32402 | -1.15239 | 39.29014 |
| 35.97654 | -1.16171 | 43.08442 |
| 39.48142 | -1.17594 | 48.88591 |
| 48.07055 | -1.23719 | 73.84127 |
| -44.3232 | -0.8939 | 79.65926 |
| -36.3142 | -0.84543 | 52.00548 |
| -32.7506 | -0.83107 | 43.81346 |
| -29.3077 | -0.82559 | 40.68733 |
| -25.2081 | -0.81931 | 37.10782 |
| -20.9366 | -0.8129 | 33.45051 |
| -16.1897 | -0.80805 | 30.68536 |
| -11.4609 | -0.80448 | 28.64403 |
| -6.30129 | -0.80002 | 26.10066 |
| -1.34286 | -0.79997 | 26.07572 |
| 3.98398 | -0.79857 | 25.27603 |
| 8.945608 | -0.80104 | 26.6814 |
| 14.14573 | -0.80265 | 27.60273 |
| 18.87078 | -0.80687 | 30.00812 |
| 23.71061 | -0.81068 | 32.18078 |
| 28.03726 | -0.8153 | 34.81597 |
| 32.31758 | -0.82383 | 39.68661 |
| 36.02286 | -0.83166 | 44.15012 |
| 39.43831 | -0.83978 | 48.78505 |
| 48.19618 | -0.88666 | 75.52856 |
| -44.2006 | -0.53479 | 78.18242 |
| -36.2281 | -0.5063 | 51.09201 |
| -32.7847 | -0.49893 | 44.09022 |
| -29.3522 | -0.49581 | 41.12389 |
| -25.2023 | -0.49108 | 36.62321 |
| -20.9444 | -0.48762 | 33.33697 |
| -16.2304 | -0.48534 | 31.17004 |
| -11.4634 | -0.48242 | 28.38841 |
| -6.32681 | -0.47989 | 25.98289 |
| -1.34597 | -0.47942 | 25.53803 |
| 3.962244 | -0.4796 | 25.70714 |
| 8.93469 | -0.48035 | 26.42126 |
| 14.11269 | -0.48137 | 27.39361 |
| 18.84094 | -0.48426 | 30.1413 |
| 23.68803 | -0.48661 | 32.3718 |
| 27.99738 | -0.48966 | 35.27668 |
| 32.28238 | -0.49425 | 39.63421 |
| 35.94703 | -0.4984 | 43.58042 |
| 39.37734 | -0.50347 | 48.4036 |
| 48.31673 | -0.53337 | 76.83243 |
| -44.223 | -0.17834 | 78.40995 |
| -36.2965 | -0.16894 | 51.60061 |
| -32.8371 | -0.16651 | 44.6456 |
| -29.3543 | -0.1652 | 40.92287 |
| -25.1932 | -0.16351 | 36.11557 |
| -20.9922 | -0.16259 | 33.46407 |
| -16.242 | -0.16167 | 30.84884 |
| -11.5079 | -0.16088 | 28.5878 |
| -6.37581 | -0.16055 | 27.67286 |
| -1.34789 | -0.15966 | 25.11342 |
| 3.942219 | -0.16009 | 26.34407 |
| 8.913258 | -0.15983 | 25.6193 |
| 14.09309 | -0.16059 | 27.77713 |
| 18.87669 | -0.16149 | 30.32986 |
| 23.66655 | -0.16227 | 32.55555 |
| 27.997 | -0.16342 | 35.83432 |
| 32.25462 | -0.16477 | 39.70116 |
| 35.94465 | -0.16628 | 44.00336 |
| 39.39131 | -0.168 | 48.921 |
| 48.35312 | -0.17808 | 77.65294 |
| -44.1493 | 0.178014 | 77.47324 |
| -36.3336 | 0.169004 | 51.77282 |
| -32.8745 | 0.166617 | 44.96419 |
| -29.3611 | 0.165342 | 41.32702 |
| -25.1999 | 0.163453 | 35.94048 |
| -20.9921 | 0.162737 | 33.89636 |
| -16.2811 | 0.161828 | 31.30536 |
| -11.5175 | 0.160894 | 28.63911 |
| -6.40381 | 0.16067 | 28.00141 |
| -1.38528 | 0.159904 | 25.81595 |
| 3.913152 | 0.16005 | 26.23242 |
| 8.882287 | 0.159967 | 25.99586 |
| 14.06523 | 0.160611 | 27.83364 |
| 18.77236 | 0.161437 | 30.19008 |
| 23.63455 | 0.162239 | 32.47689 |
| 27.96542 | 0.163424 | 35.85619 |
| 32.2212 | 0.164755 | 39.65297 |
| 35.91771 | 0.166318 | 44.11022 |
| 39.43875 | 0.168345 | 49.89263 |
| 48.29983 | 0.178041 | 77.54877 |
| -44.2178 | 0.534784 | 78.17781 |
| -36.2734 | 0.506313 | 51.10828 |
| -32.9254 | 0.500352 | 45.44057 |
| -29.4708 | 0.497028 | 42.2801 |
| -25.2854 | 0.491654 | 37.17041 |
| -21.0257 | 0.487949 | 33.64785 |
| -16.3017 | 0.485419 | 31.24282 |
| -11.5261 | 0.482702 | 28.65953 |
| -6.41902 | 0.481304 | 27.33047 |
| -1.42327 | 0.480157 | 26.24005 |
| 3.890108 | 0.480257 | 26.33486 |
| 8.858882 | 0.480586 | 26.64766 |
| 14.03546 | 0.481707 | 27.71371 |
| 18.77189 | 0.483588 | 29.50156 |
| 23.6055 | 0.486756 | 32.51384 |
| 27.9315 | 0.490259 | 35.84417 |
| 32.21932 | 0.494716 | 40.08224 |
| 35.85094 | 0.498516 | 43.69483 |
| 39.33844 | 0.504179 | 49.07875 |
| 48.38073 | 0.534805 | 78.19724 |
| -44.2959 | 0.892582 | 78.90476 |
| -36.3027 | 0.843934 | 51.15294 |
| -32.8908 | 0.832667 | 44.72565 |
| -29.4756 | 0.828007 | 42.06756 |
| -25.3124 | 0.819643 | 37.296 |
| -21.0953 | 0.814244 | 34.2162 |
| -16.3082 | 0.808254 | 30.79878 |
| -11.5599 | 0.803838 | 28.27995 |
| -6.43648 | 0.801545 | 26.97186 |
| -1.45648 | 0.79915 | 25.60541 |
| 3.862385 | 0.800172 | 26.18854 |
| 8.829462 | 0.801477 | 26.93321 |
| 14.01576 | 0.803223 | 27.9291 |
| 18.73538 | 0.805963 | 29.49201 |
| 23.59476 | 0.811866 | 32.85939 |
| 27.9018 | 0.817214 | 35.91051 |
| 32.15975 | 0.823839 | 39.68979 |
| 35.86586 | 0.832003 | 44.34702 |
| 39.30069 | 0.840265 | 49.06018 |
| 48.63069 | 0.896602 | 81.1981 |
| -44.4634 | 1.254011 | 80.6965 |
| -36.2422 | 1.180829 | 50.87657 |
| -32.9136 | 1.166075 | 44.86471 |
| -29.4961 | 1.159306 | 42.10654 |
| -25.3343 | 1.147601 | 37.33716 |
| -21.1175 | 1.140466 | 34.42987 |
| -16.3411 | 1.132347 | 31.12142 |
| -11.5719 | 1.125366 | 28.27676 |
| -6.46205 | 1.122761 | 27.21527 |
| -1.49556 | 1.119711 | 25.97256 |
| 3.831927 | 1.11991 | 26.05363 |
| 8.789489 | 1.121807 | 26.82656 |
| 13.98205 | 1.123958 | 27.70312 |
| 18.70281 | 1.128691 | 29.63169 |
| 23.59071 | 1.137883 | 33.37735 |
| 27.90138 | 1.144245 | 35.96977 |
| 32.17996 | 1.155232 | 40.44657 |
| 35.8543 | 1.165492 | 44.62718 |
| 39.23885 | 1.175398 | 48.66374 |
| 48.55472 | 1.254316 | 80.82053 |
| -44.515 | 1.613575 | 81.10031 |
| -36.2196 | 1.515099 | 49.89109 |
| -33.002 | 1.502573 | 45.92133 |
| -29.5552 | 1.49259 | 42.7574 |
| -25.3525 | 1.475401 | 37.30973 |
| -21.1243 | 1.466072 | 34.35309 |
| -16.3669 | 1.456216 | 31.22954 |
| -11.6349 | 1.449774 | 29.18789 |
| -6.49366 | 1.444487 | 27.51236 |
| -1.50444 | 1.439564 | 25.95226 |
| 3.808355 | 1.440608 | 26.28317 |
| 8.7905 | 1.443012 | 27.04498 |
| 13.9715 | 1.447035 | 28.3201 |
| 18.66394 | 1.45091 | 29.54791 |
| 23.55296 | 1.462744 | 33.2984 |
| 27.8698 | 1.472812 | 36.48945 |
| 32.16378 | 1.485869 | 40.6274 |
| 35.74632 | 1.49537 | 43.63841 |
| 39.16443 | 1.509525 | 48.12465 |
| 48.43198 | 1.61026 | 80.04988 |
| -44.2498 | 1.959912 | 77.92797 |
| -36.1727 | 1.850238 | 49.48929 |
| -32.9985 | 1.835397 | 45.64082 |
| -29.5013 | 1.822058 | 42.18203 |
| -25.333 | 1.800671 | 36.63654 |
| -21.1287 | 1.791241 | 34.19129 |
| -16.3906 | 1.779732 | 31.20689 |
| -11.6918 | 1.774074 | 29.73975 |
| -6.51143 | 1.764066 | 27.14482 |
| -1.51729 | 1.764251 | 27.19255 |
| 3.783388 | 1.761146 | 26.38766 |
| 8.727391 | 1.764472 | 27.25006 |
| 13.9454 | 1.769191 | 28.47374 |
| 18.66708 | 1.777126 | 30.53109 |
| 23.5219 | 1.787498 | 33.22069 |
| 27.92485 | 1.801582 | 36.87265 |
| 32.12625 | 1.81577 | 40.55159 |
| 35.70467 | 1.825693 | 43.12471 |
| 39.39182 | 1.856982 | 51.23791 |
| 48.36254 | 1.96429 | 79.06316 |
| -44.3081 | 2.318793 | 78.48361 |
| -36.1995 | 2.185817 | 49.30751 |
| -32.9401 | 2.164278 | 44.5817 |
| -29.5739 | 2.155031 | 42.55294 |
| -25.3461 | 2.127754 | 36.56793 |
| -21.1327 | 2.116198 | 34.03259 |
| -16.4133 | 2.103311 | 31.20513 |
| -11.6648 | 2.096717 | 29.7583 |
| -6.53987 | 2.086635 | 27.54606 |
| -1.5561 | 2.08621 | 27.45302 |
| 3.759137 | 2.083706 | 26.90359 |
| 8.723283 | 2.084848 | 27.15398 |
| 13.90669 | 2.089567 | 28.18937 |
| 18.64143 | 2.101231 | 30.74871 |
| 23.50474 | 2.113607 | 33.46411 |
| 27.79794 | 2.126934 | 36.38815 |
| 32.08715 | 2.14527 | 40.41126 |
| 35.74431 | 2.162442 | 44.17882 |
| 39.35789 | 2.194602 | 51.23509 |
| 48.53289 | 2.331967 | 81.37427 |
| -44.4486 | 2.683538 | 80.0063 |
| -36.2379 | 2.521856 | 49.26179 |
| -32.9594 | 2.497613 | 44.65184 |
| -29.5876 | 2.489232 | 43.05815 |
| -25.3608 | 2.454975 | 36.54414 |
| -21.1864 | 2.442512 | 34.17432 |
| -16.4239 | 2.424798 | 30.80588 |
| -11.6913 | 2.416314 | 29.19249 |
| -6.56915 | 2.409831 | 27.9598 |
| -1.59798 | 2.407068 | 27.43448 |
| 3.735651 | 2.40477 | 26.99748 |
| 8.720847 | 2.406692 | 27.36298 |
| 13.88461 | 2.412887 | 28.54089 |
| 18.58774 | 2.422174 | 30.30684 |
| 23.45509 | 2.436931 | 33.1129 |
| 27.79853 | 2.457207 | 36.96851 |
| 32.08004 | 2.477325 | 40.79409 |
| 35.7457 | 2.497549 | 44.63973 |
| 39.24805 | 2.527256 | 50.28862 |
| 48.68121 | 2.701196 | 83.36419 |
| -44.6324 | 3.053058 | 81.97188 |
| -36.2172 | 2.855485 | 48.82252 |
| -33.0342 | 2.835586 | 45.48374 |
| -29.6049 | 2.818136 | 42.55589 |
| -25.405 | 2.785211 | 37.03178 |
| -21.2053 | 2.768852 | 34.28692 |
| -16.4473 | 2.748307 | 30.83986 |
| -11.7479 | 2.741762 | 29.74173 |
| -6.60413 | 2.734592 | 28.53874 |
| -1.60431 | 2.729694 | 27.7169 |
| 3.710491 | 2.725936 | 27.08638 |
| 8.664966 | 2.731128 | 27.95748 |
| 13.88098 | 2.739474 | 29.35787 |
| 18.56911 | 2.742901 | 29.93271 |
| 23.49172 | 2.769531 | 34.40093 |
| 27.79703 | 2.784784 | 36.9601 |
| 31.99969 | 2.803248 | 40.05805 |
| 35.75235 | 2.831264 | 44.75857 |
| 39.17094 | 2.861001 | 49.748 |
| 48.80031 | 3.071387 | 85.04718 |
| -44.563 | 3.406309 | 81.08143 |
| -36.244 | 3.192479 | 48.98088 |
| -33.0727 | 3.171423 | 45.81986 |
| -29.7023 | 3.161311 | 44.30181 |
| -25.49 | 3.121132 | 38.27003 |
| -21.2186 | 3.09479 | 34.31549 |
| -16.4793 | 3.073287 | 31.08743 |
| -11.7907 | 3.065334 | 29.89361 |
| -6.62017 | 3.051419 | 27.80462 |
| -1.61246 | 3.048517 | 27.36891 |
| 3.669946 | 3.039542 | 26.02159 |
| 8.662271 | 3.053074 | 28.05315 |
| 13.82425 | 3.055165 | 28.36693 |
| 18.556 | 3.064155 | 29.71652 |
| 23.45286 | 3.094033 | 34.20193 |
| 27.80796 | 3.11425 | 37.23691 |
| 31.96888 | 3.132711 | 40.00838 |
| 35.69495 | 3.162485 | 44.47801 |
| 39.0843 | 3.193138 | 49.07981 |
| 48.73995 | 3.43184 | 84.91418 |
| -44.5555 | 3.762867 | 80.80956 |
| -36.4038 | 3.539075 | 50.41319 |
| -33.0244 | 3.498684 | 44.92711 |
| -29.7989 | 3.499722 | 45.06813 |
| -25.521 | 3.45126 | 38.4857 |
| -21.265 | 3.425601 | 35.00067 |
| -16.5273 | 3.402631 | 31.88077 |
| -11.7841 | 3.392088 | 30.44869 |
| -6.64642 | 3.372855 | 27.83637 |
| -1.6537 | 3.36956 | 27.38883 |
| 3.643889 | 3.358209 | 25.84709 |
| 8.637437 | 3.379742 | 28.77184 |
| 13.80456 | 3.37828 | 28.57331 |
| 18.55104 | 3.392311 | 30.47901 |
| 23.45555 | 3.424926 | 34.90897 |
| 27.70957 | 3.433743 | 36.10656 |
| 31.97088 | 3.466248 | 40.52153 |
| 35.62054 | 3.491694 | 43.97763 |
| 39.13689 | 3.536819 | 50.1068 |
| 48.62837 | 3.787167 | 84.11015 |
| -44.6916 | 4.132973 | 82.26529 |
| -36.4941 | 3.887764 | 51.85594 |
| -33.0347 | 3.831392 | 44.86505 |
| -29.6617 | 3.817435 | 43.13422 |
| -25.5188 | 3.776809 | 38.09602 |
| -21.2959 | 3.755042 | 35.39657 |
| -16.5749 | 3.732855 | 32.64509 |
| -11.8168 | 3.711675 | 30.01855 |
| -6.66789 | 3.694327 | 27.86708 |
| -1.69596 | 3.697901 | 28.3104 |
| 3.620613 | 3.682187 | 26.36159 |
| 8.595496 | 3.698452 | 28.37872 |
| 13.8048 | 3.707521 | 29.50333 |
| 18.52336 | 3.71712 | 30.69381 |
| 23.39872 | 3.746661 | 34.35722 |
| 27.66512 | 3.76369 | 36.46915 |
| 31.95792 | 3.799017 | 40.85014 |
| 35.60804 | 3.826895 | 44.30738 |
| 39.10265 | 3.873958 | 50.14387 |
| 48.46065 | 4.137934 | 82.88042 |
| -44.7203 | 4.49361 | 82.40762 |
| -36.6194 | 4.234135 | 52.8034 |
| -33.0341 | 4.16266 | 44.64866 |
| -29.6398 | 4.139115 | 41.96233 |
| -25.5237 | 4.10281 | 37.82022 |
| -21.3276 | 4.079418 | 35.15139 |
| -16.5916 | 4.055878 | 32.46567 |
| -11.8392 | 4.038815 | 30.51894 |
| -6.68321 | 4.011512 | 27.40379 |
| -1.70033 | 4.020032 | 28.37595 |
| 3.602366 | 4.011246 | 27.37344 |
| 8.586479 | 4.018511 | 28.20234 |
| 13.77369 | 4.029059 | 29.4058 |
| 18.51465 | 4.046021 | 31.34102 |
| 23.34579 | 4.068441 | 33.89896 |
| 27.66894 | 4.096359 | 37.08418 |
| 31.98577 | 4.136637 | 41.67966 |
| 35.58725 | 4.161265 | 44.48952 |
| 39.06277 | 4.20992 | 50.04067 |
| 48.39004 | 4.488545 | 81.82971 |
| -44.6738 | 4.847647 | 81.83175 |
| -36.555 | 4.567148 | 52.19941 |
| -33.0645 | 4.497241 | 44.81435 |
| -29.6563 | 4.47549 | 42.51656 |
| -25.5442 | 4.431322 | 37.8506 |
| -21.3433 | 4.407338 | 35.31684 |
| -16.6026 | 4.376979 | 32.10974 |
| -11.8762 | 4.360695 | 30.3895 |
| -6.70753 | 4.33169 | 27.32535 |
| -1.737 | 4.338381 | 28.03217 |
| 3.573987 | 4.330411 | 27.19022 |
| 8.555933 | 4.342585 | 28.47628 |
| 13.74584 | 4.351769 | 29.44652 |
| 18.44322 | 4.360986 | 30.42021 |
| 23.32084 | 4.395743 | 34.092 |
| 27.63631 | 4.419157 | 36.56541 |
| 31.86694 | 4.45516 | 40.36884 |
| 35.61801 | 4.502464 | 45.36617 |
| 39.02914 | 4.54641 | 50.00861 |
| -44.8063 | 5.220822 | 83.21751 |
| -36.5844 | 4.907394 | 52.39012 |
| -33.1353 | 4.83818 | 45.58249 |
| -29.7579 | 4.815885 | 43.38963 |
| -25.5463 | 4.756719 | 37.57034 |
| -21.424 | 4.742235 | 36.14581 |
| -16.6372 | 4.704976 | 32.4811 |
| -11.8835 | 4.683146 | 30.33404 |
| -6.74867 | 4.663679 | 28.41934 |
| -1.77255 | 4.651698 | 27.24099 |
| 3.545414 | 4.653043 | 27.37328 |
| 8.518714 | 4.663441 | 28.39592 |
| 13.73135 | 4.679054 | 29.93156 |
| 18.43304 | 4.690026 | 31.0107 |
| 23.33461 | 4.730164 | 34.95854 |
| 27.64557 | 4.754031 | 37.30602 |
| 31.89877 | 4.794714 | 41.30743 |
| 35.54244 | 4.830397 | 44.81698 |
| 39.03767 | 4.888732 | 50.55457 |
| 48.41499 | 5.221427 | 83.27705 |
| -44.7926 | 5.57681 | 82.84317 |
| -36.5978 | 5.245217 | 52.33328 |
| -33.2576 | 5.188068 | 47.07498 |
| -29.7684 | 5.152547 | 43.80662 |
| -25.5749 | 5.086241 | 37.7058 |
| -21.4503 | 5.072905 | 36.47884 |
| -16.6707 | 5.033624 | 32.86451 |
| -11.9346 | 5.009986 | 30.68959 |
| -6.77361 | 4.986015 | 28.48404 |
| -1.78061 | 4.974404 | 27.41567 |
| 3.518224 | 4.973041 | 27.29027 |
| 8.478895 | 4.983418 | 28.24504 |
| 13.69461 | 4.99852 | 29.6346 |
| 18.41946 | 5.019426 | 31.55817 |
| 23.2418 | 5.042975 | 33.72489 |
| 27.54473 | 5.069385 | 36.15494 |
| 31.87313 | 5.126264 | 41.38837 |
| 35.46154 | 5.156578 | 44.17752 |
| 38.98895 | 5.223872 | 50.36934 |
| 48.44679 | 5.585595 | 83.6515 |
| -44.7755 | 5.933617 | 82.58502 |
| -36.6483 | 5.583285 | 52.30448 |
| -33.2832 | 5.52433 | 47.20875 |
| -29.761 | 5.474334 | 42.88741 |
| -25.6021 | 5.416409 | 37.88071 |
| -21.4473 | 5.396225 | 36.13617 |
| -16.7114 | 5.364036 | 33.3539 |
| -11.9399 | 5.332184 | 30.60081 |
| -6.8009 | 5.309593 | 28.64821 |
| -1.82278 | 5.297251 | 27.58142 |
| 3.496517 | 5.299639 | 27.78789 |
| 8.490041 | 5.316638 | 29.25718 |
| 13.67089 | 5.322512 | 29.76482 |
| 18.38872 | 5.34491 | 31.70082 |
| 23.20768 | 5.367611 | 33.66292 |
| 27.54484 | 5.408843 | 37.22678 |
| 31.84954 | 5.458359 | 41.50663 |
| 35.43674 | 5.490621 | 44.29515 |
| 38.95642 | 5.560594 | 50.34318 |
| 48.42358 | 5.948586 | 83.87884 |
| -44.9098 | 6.309799 | 83.93527 |
| -36.6086 | 5.917503 | 51.96522 |
| -33.3082 | 5.860897 | 47.35215 |
| -29.7626 | 5.809648 | 43.1756 |
| -25.6546 | 5.752527 | 38.5205 |
| -21.4705 | 5.717831 | 35.69298 |
| -16.7419 | 5.692571 | 33.63445 |
| -11.9509 | 5.65677 | 30.71685 |
| -6.82637 | 5.63268 | 28.75364 |
| -1.83394 | 5.633316 | 28.8055 |
| 3.474712 | 5.627785 | 28.35468 |
| 8.444883 | 5.632696 | 28.75493 |
| 13.63611 | 5.641668 | 29.4861 |
| 18.38349 | 5.667985 | 31.63079 |
| 23.239 | 5.707826 | 34.87763 |
| 27.54627 | 5.744084 | 37.83244 |
| 31.822 | 5.790312 | 41.59979 |
| 35.50335 | 5.839629 | 45.61886 |
| 38.96061 | 5.902851 | 50.77115 |
| 48.077 | 6.270192 | 80.7075 |
| -44.9706 | 6.677715 | 84.50235 |
| -36.6982 | 6.267253 | 52.85998 |
| -33.3336 | 6.197062 | 47.44898 |
| -29.8422 | 6.148396 | 43.69731 |
| -25.7188 | 6.092064 | 39.35475 |
| -21.4733 | 6.050639 | 36.16131 |
| -16.7517 | 6.012376 | 33.21165 |
| -11.9945 | 5.980157 | 30.72785 |
| -6.85577 | 5.959985 | 29.17283 |
| -1.87448 | 5.959565 | 29.14043 |
| 3.446614 | 5.948476 | 28.28562 |
| 8.398932 | 5.948048 | 28.25258 |
| 13.62324 | 5.971137 | 30.03254 |
| 18.34984 | 5.992442 | 31.67494 |
| 23.20337 | 6.032456 | 34.75959 |
| 27.57129 | 6.078161 | 38.28299 |
| 31.80972 | 6.124558 | 41.85971 |
| 35.44072 | 6.168828 | 45.27241 |
| 38.87445 | 6.231676 | 50.11737 |
| 48.37661 | 6.671788 | 84.04542 |
| -45.1055 | 7.057405 | 85.87232 |
| -36.7792 | 6.617373 | 53.69004 |
| -33.3998 | 6.540973 | 48.10247 |
| -29.9162 | 6.486765 | 44.13783 |
| -25.7481 | 6.424729 | 39.60077 |
| -21.4791 | 6.37527 | 35.98353 |
| -16.7633 | 6.332777 | 32.87573 |
| -12.0086 | 6.305243 | 30.86205 |
| -6.88182 | 6.283423 | 29.26616 |
| -1.91125 | 6.27293 | 28.49873 |
| 3.415918 | 6.263216 | 27.7883 |
| 8.401879 | 6.276589 | 28.76634 |
| 13.58995 | 6.293404 | 29.99618 |
| 18.33403 | 6.323559 | 32.2016 |
| 23.18274 | 6.36166 | 34.98815 |
| 27.50377 | 6.399647 | 37.76635 |
| 31.75359 | 6.451865 | 41.58544 |
| 35.44242 | 6.508719 | 45.74349 |
| 38.87173 | 6.57317 | 50.4572 |
| 48.35261 | 7.030649 | 83.91552 |
| -44.914 | 7.384959 | 83.48164 |
| -36.8388 | 6.963666 | 54.17286 |
| -33.4218 | 6.877365 | 48.16908 |
| -29.8901 | 6.816834 | 43.95798 |
| -25.7789 | 6.757749 | 39.84755 |
| -21.4999 | 6.694061 | 35.41684 |
| -16.7704 | 6.651023 | 32.42276 |
| -12.0748 | 6.643164 | 31.87603 |
| -6.90418 | 6.60359 | 29.12286 |
| -1.89091 | 6.607831 | 29.41793 |
| 3.394595 | 6.593331 | 28.40921 |
| 8.374461 | 6.604547 | 29.18946 |
| 13.57237 | 6.621025 | 30.33578 |
| 18.31223 | 6.652581 | 32.5311 |
| 23.14619 | 6.687 | 34.92563 |
| 27.47445 | 6.729013 | 37.84839 |
| 31.75752 | 6.789679 | 42.06886 |
| 35.55169 | 6.863986 | 47.23829 |
| 38.92231 | 6.924587 | 51.45422 |
| 48.40351 | 7.405913 | 84.93943 |
| -44.8559 | 7.732603 | 82.64599 |
| -36.8412 | 7.306499 | 54.38129 |
| -33.4656 | 7.217908 | 48.50478 |
| -29.8825 | 7.150896 | 44.05968 |
| -25.8162 | 7.092745 | 40.20238 |
| -21.5694 | 7.039445 | 36.66683 |
| -16.8199 | 6.986731 | 33.17014 |
| -12.0842 | 6.967278 | 31.87981 |
| -6.93006 | 6.926851 | 29.19815 |
| -1.93048 | 6.928017 | 29.27549 |
| 3.371136 | 6.91979 | 28.72981 |
| 8.343492 | 6.93269 | 29.58547 |
| 13.54419 | 6.94441 | 30.36286 |
| 18.29521 | 6.984141 | 32.99835 |
| 23.12517 | 7.016017 | 35.11281 |
| 27.43578 | 7.056781 | 37.81681 |
| 31.76358 | 7.128817 | 42.59516 |
| 35.47519 | 7.189974 | 46.65185 |
| 38.95598 | 7.274136 | 52.2346 |
| 48.27871 | 7.754682 | 84.11059 |
| -44.7895 | 8.076709 | 81.66038 |
| -36.91 | 7.649393 | 54.57503 |
| -33.4881 | 7.554995 | 48.59164 |
| -29.9591 | 7.490864 | 44.52674 |
| -25.7882 | 7.409031 | 39.33979 |
| -21.637 | 7.37546 | 37.21188 |
| -16.8666 | 7.321902 | 33.81713 |
| -12.1152 | 7.285662 | 31.52008 |
| -6.95075 | 7.246344 | 29.0279 |
| -1.96671 | 7.255086 | 29.58202 |
| 3.340599 | 7.232719 | 28.16429 |
| 8.307808 | 7.256737 | 29.68666 |
| 13.5161 | 7.2674 | 30.36252 |
| 18.24757 | 7.303389 | 32.6437 |
| 23.10445 | 7.345161 | 35.2914 |
| 27.42234 | 7.391347 | 38.2189 |
| 31.72706 | 7.459676 | 42.5499 |
| 35.41274 | 7.518768 | 46.29543 |
| 38.88934 | 7.605481 | 51.79168 |
| 48.42771 | 8.13703 | 85.48385 |
| -44.8857 | 8.450758 | 82.5758 |
| -36.8614 | 7.981449 | 54.09461 |
| -33.5571 | 7.902518 | 49.30446 |
| -29.9728 | 7.822946 | 44.47546 |
| -25.8336 | 7.746205 | 39.81823 |
| -21.6575 | 7.706813 | 37.4276 |
| -16.8746 | 7.641205 | 33.44604 |
| -12.1363 | 7.616445 | 31.94339 |
| -6.97258 | 7.565684 | 28.86285 |
| -2.00788 | 7.579211 | 29.68376 |
| 3.309999 | 7.549975 | 27.9095 |
| 8.306407 | 7.581813 | 29.84167 |
| 13.47933 | 7.586256 | 30.11133 |
| 18.19468 | 7.621408 | 32.24461 |
| 23.08435 | 7.675233 | 35.51112 |
| 27.36694 | 7.714697 | 37.90607 |
| 31.68286 | 7.787831 | 42.34441 |
| 35.36308 | 7.849565 | 46.09086 |
| 38.90445 | 7.952988 | 52.36737 |
| 48.66543 | 8.541525 | 88.08421 |
| -44.9798 | 8.82365 | 83.34916 |
| -36.9447 | 8.334897 | 54.89861 |
| -33.6269 | 8.25107 | 50.01896 |
| -29.9632 | 8.149047 | 44.08016 |
| -25.8715 | 8.081915 | 40.17236 |
| -21.6574 | 8.029527 | 37.12285 |
| -16.9155 | 7.975726 | 33.99104 |
| -12.1789 | 7.940659 | 31.94982 |
| -7.00708 | 7.89986 | 29.57485 |
| -1.98347 | 7.902617 | 29.73537 |
| 3.287045 | 7.880602 | 28.45384 |
| 8.252648 | 7.887471 | 28.85368 |
| 13.47221 | 7.922286 | 30.88032 |
| 18.20106 | 7.950021 | 32.49475 |
| 23.06958 | 8.007271 | 35.82734 |
| 27.37707 | 8.046878 | 38.13283 |
| 31.64908 | 8.119261 | 42.34632 |
| 35.34592 | 8.188289 | 46.36448 |
| 38.83785 | 8.284502 | 51.96507 |
| 48.48197 | 8.887089 | 87.04198 |
| -45.1355 | 9.212736 | 84.96754 |
| -36.9515 | 8.67127 | 54.68456 |
| -33.6528 | 8.58977 | 50.12647 |
| -30.0257 | 8.496293 | 44.89851 |
| -25.9268 | 8.424408 | 40.8781 |
| -21.6753 | 8.346387 | 36.5146 |
| -16.943 | 8.303853 | 34.13577 |
| -12.2184 | 8.266909 | 32.06958 |
| -7.0394 | 8.233461 | 30.19888 |
| -2.05 | 8.213306 | 29.07166 |
| 3.25955 | 8.195145 | 28.05597 |
| 8.204817 | 8.196406 | 28.12648 |
| 13.4391 | 8.243649 | 30.76871 |
| 18.17374 | 8.277841 | 32.68097 |
| 23.0364 | 8.333964 | 35.81978 |
| 27.36095 | 8.380092 | 38.39966 |
| 31.62593 | 8.454211 | 42.54494 |
| 35.33818 | 8.529605 | 46.76155 |
| 38.73736 | 8.606789 | 51.07826 |
| 48.18832 | 9.194265 | 83.93448 |
| -45.6299 | 9.677818 | 90.55365 |
| -36.9479 | 9.013488 | 54.80127 |
| -33.6819 | 8.929313 | 50.2712 |
| -30.1038 | 8.848088 | 45.89987 |
| -25.9758 | 8.764886 | 41.4222 |
| -21.6664 | 8.677966 | 36.74436 |
| -16.9536 | 8.623679 | 33.82282 |
| -12.1985 | 8.592528 | 32.14634 |
| -7.06431 | 8.556697 | 30.21799 |
| -2.0596 | 8.550137 | 29.86498 |
| 3.239272 | 8.528793 | 28.71629 |
| 8.207621 | 8.525398 | 28.53357 |
| 13.39428 | 8.555864 | 30.17315 |
| 18.16782 | 8.600875 | 32.59554 |
| 23.01839 | 8.665107 | 36.05234 |
| 27.39033 | 8.728379 | 39.45747 |
| 31.65148 | 8.801033 | 43.36752 |
| 35.2772 | 8.858165 | 46.44217 |
| 38.72783 | 8.948702 | 51.31464 |
| -45.5414 | 10.02353 | 89.5431 |
| -36.8506 | 9.333367 | 53.75096 |
| -33.6828 | 9.262309 | 50.06584 |
| -30.1383 | 9.178824 | 45.73628 |
| -26.009 | 9.099413 | 41.61805 |
| -21.7106 | 9.003828 | 36.66098 |
| -16.9948 | 8.95839 | 34.30453 |
| -12.2463 | 8.921014 | 32.36623 |
| -7.08142 | 8.87005 | 29.72319 |
| -2.09282 | 8.853622 | 28.87123 |
| 3.220868 | 8.867414 | 29.58652 |
| 8.182183 | 8.856232 | 29.00658 |
| 13.39665 | 8.89955 | 31.25309 |
| 18.1339 | 8.926384 | 32.64471 |
| 23.02741 | 9.007804 | 36.86714 |
| 27.32597 | 9.047619 | 38.93198 |
| 31.59036 | 9.125837 | 42.98837 |
| 35.31236 | 9.210842 | 47.39679 |
| 38.79787 | 9.310451 | 52.56251 |
| -37.0157 | 9.700861 | 55.15703 |
| -33.6779 | 9.59295 | 49.75711 |
| -30.073 | 9.498611 | 45.03633 |
| -25.977 | 9.411708 | 40.68766 |
| -21.7628 | 9.348792 | 37.53926 |
| -17.0237 | 9.287535 | 34.47392 |
| -12.2586 | 9.248015 | 32.49633 |
| -7.10737 | 9.194949 | 29.84088 |
| -2.13307 | 9.185135 | 29.34978 |
| 3.19497 | 9.197343 | 29.9607 |
| 8.15719 | 9.193942 | 29.79048 |
| 13.38224 | 9.232707 | 31.7303 |
| 18.08579 | 9.24469 | 32.32995 |
| 22.97755 | 9.327509 | 36.47427 |
| 27.30265 | 9.380602 | 39.13106 |
| 31.59468 | 9.467209 | 43.46493 |
| 35.22765 | 9.533346 | 46.77445 |
| 38.76432 | 9.649403 | 52.58202 |
| -37.0036 | 10.0444 | 55.30959 |
| -33.7384 | 9.942048 | 50.36155 |
| -30.0648 | 9.812717 | 44.10914 |
| -26.0221 | 9.751348 | 41.14232 |
| -21.7886 | 9.683568 | 37.86551 |
| -17.0409 | 9.609721 | 34.29543 |
| -12.3311 | 9.595402 | 33.60318 |
| -7.1456 | 9.53444 | 30.65605 |
| -2.14382 | 9.511387 | 29.54156 |
| 3.168381 | 9.519969 | 29.95643 |
| 8.134075 | 9.533944 | 30.63206 |
| 13.35126 | 9.553856 | 31.59466 |
| 18.09672 | 9.594805 | 33.57431 |
| 22.92821 | 9.646007 | 36.04966 |
| 27.2993 | 9.720958 | 39.67312 |
| 31.57524 | 9.802872 | 43.63318 |
| 35.18087 | 9.864159 | 46.59606 |
| 38.7501 | 9.992039 | 52.77834 |
| -37.0017 | 10.37003 | 54.61506 |
| -33.723 | 10.26879 | 49.88091 |
| -30.1113 | 10.16674 | 45.10942 |
| -26.0542 | 10.08589 | 41.32897 |
| -21.8027 | 9.998933 | 37.26271 |
| -17.0496 | 9.9284 | 33.96465 |
| -12.3448 | 9.922732 | 33.69963 |
| -7.17151 | 9.85887 | 30.71347 |
| -2.1532 | 9.836318 | 29.65894 |
| 3.142671 | 9.844131 | 30.02431 |
| 8.087835 | 9.846683 | 30.1436 |
| 13.32919 | 9.882271 | 31.8077 |
| 18.07236 | 9.925106 | 33.81064 |
| 22.91074 | 9.978848 | 36.32356 |
| 27.27507 | 10.05363 | 39.82025 |
| 31.55246 | 10.13724 | 43.72993 |
| 35.1606 | 10.20236 | 46.77462 |
| 38.76925 | 10.34439 | 53.41614 |
| -36.9498 | 10.69945 | 54.13576 |
| -33.7469 | 10.60724 | 49.96097 |
| -30.2244 | 10.52436 | 46.20878 |
| -26.087 | 10.42153 | 41.55333 |
| -21.7767 | 10.3246 | 37.16453 |
| -17.0753 | 10.25751 | 34.12693 |
| -12.3598 | 10.22863 | 32.81974 |
| -7.1945 | 10.17749 | 30.50444 |
| -2.19302 | 10.1667 | 30.01583 |
| 3.117254 | 10.16813 | 30.08042 |
| 8.052857 | 10.17124 | 30.22127 |
| 13.3047 | 10.20958 | 31.95706 |
| 18.00523 | 10.23254 | 32.99646 |
| 22.86551 | 10.2998 | 36.04194 |
| 27.22835 | 10.3784 | 39.60062 |
| 31.58379 | 10.49027 | 44.66518 |
| 35.16401 | 10.54883 | 47.31639 |
| 38.76121 | 10.68992 | 53.70463 |
| -37.0665 | 11.05914 | 55.01478 |
| -33.7719 | 10.94579 | 50.04061 |
| -30.302 | 10.88132 | 47.21152 |
| -26.0824 | 10.74203 | 41.09942 |
| -21.8383 | 10.6606 | 37.5259 |
| -17.1123 | 10.59315 | 34.56602 |
| -12.4153 | 10.56348 | 33.26412 |
| -7.21978 | 10.49978 | 30.46873 |
| -2.23452 | 10.50189 | 30.56148 |
| 3.093469 | 10.50389 | 30.64919 |
| 8.06039 | 10.50737 | 30.80191 |
| 13.26364 | 10.52385 | 31.52506 |
| 17.97359 | 10.56146 | 33.17544 |
| 22.8312 | 10.62402 | 35.92067 |
| 27.19458 | 10.70934 | 39.66464 |
| 31.52258 | 10.81356 | 44.23814 |
| 35.17472 | 10.88695 | 47.45865 |
| 38.68354 | 11.01676 | 53.15482 |
| -37.0063 | 11.38558 | 54.42556 |
| -33.8555 | 11.30487 | 50.98955 |
| -30.246 | 11.17825 | 45.59911 |
| -26.0871 | 11.06707 | 40.8658 |
| -21.8746 | 10.98459 | 37.35469 |
| -17.1509 | 10.93053 | 35.05292 |
| -12.3888 | 10.88706 | 33.20226 |
| -7.24729 | 10.82882 | 30.72326 |
| -2.27118 | 10.81765 | 30.24764 |
| 3.063238 | 10.81835 | 30.27727 |
| 8.060036 | 10.83555 | 31.00953 |
| 13.22314 | 10.84014 | 31.20483 |
| 17.99213 | 10.90053 | 33.7757 |
| 22.84778 | 10.97258 | 36.84331 |
| 27.17213 | 11.04349 | 39.86184 |
| 31.53643 | 11.16232 | 44.92079 |
| 35.18048 | 11.23504 | 48.01662 |
| 38.56266 | 11.32976 | 52.04906 |
| -37.0833 | 11.73228 | 54.70761 |
| -33.8518 | 11.63497 | 50.68539 |
| -30.3305 | 11.53854 | 46.69879 |
| -26.1656 | 11.42357 | 41.94638 |
| -21.8939 | 11.31633 | 37.51309 |
| -17.184 | 11.26367 | 35.33646 |
| -12.4382 | 11.21965 | 33.51672 |
| -7.28484 | 11.16922 | 31.4321 |
| -2.24352 | 11.11529 | 29.20288 |
| 3.03711 | 11.15365 | 30.78842 |
| 8.022082 | 11.15645 | 30.90406 |
| 13.215 | 11.18132 | 31.93245 |
| 17.96538 | 11.22854 | 33.88407 |
| 22.81766 | 11.30036 | 36.85328 |
| 27.14911 | 11.37732 | 40.03457 |
| 31.44256 | 11.47323 | 43.99936 |
| 35.17594 | 11.57055 | 48.02222 |
| 38.65432 | 11.70473 | 53.56875 |
| -37.1405 | 12.08479 | 55.20753 |
| -33.8125 | 11.95266 | 49.89964 |
| -30.3368 | 11.86892 | 46.53547 |
| -26.1976 | 11.7607 | 42.18773 |
| -21.9596 | 11.67222 | 38.63322 |
| -17.2099 | 11.59084 | 35.36406 |
| -12.5109 | 11.57688 | 34.80311 |
| -7.30487 | 11.48663 | 31.17755 |
| -2.28352 | 11.44772 | 29.61426 |
| 3.005228 | 11.45781 | 30.01967 |
| 7.989562 | 11.48866 | 31.25922 |
| 13.17179 | 11.49496 | 31.51214 |
| 17.93081 | 11.55539 | 33.93986 |
| 22.78138 | 11.62458 | 36.71934 |
| 27.1056 | 11.68926 | 39.31773 |
| 31.41805 | 11.80876 | 44.11866 |
| 35.14033 | 11.90497 | 47.98365 |
| 38.65895 | 12.05421 | 53.97924 |
| -37.1707 | 12.43993 | 55.78311 |
| -33.8431 | 12.29346 | 50.06009 |
| -30.3167 | 12.18967 | 46.00469 |
| -26.2174 | 12.09163 | 42.17408 |
| -21.9758 | 12.00354 | 38.7319 |
| -17.2504 | 11.92921 | 35.82791 |
| -12.5189 | 11.90335 | 34.81733 |
| -7.32881 | 11.80957 | 31.15318 |
| -2.32012 | 11.76294 | 29.33112 |
| 2.976578 | 11.77883 | 29.95203 |
| 7.938576 | 11.79314 | 30.51104 |
| 13.1722 | 11.84175 | 32.41055 |
| 17.92491 | 11.87948 | 33.88467 |
| 22.72671 | 11.93761 | 36.15578 |
| 27.09453 | 12.04545 | 40.36946 |
| 31.42934 | 12.15797 | 44.76594 |
| 35.0626 | 12.22692 | 47.46007 |
| 38.56159 | 12.37288 | 53.16327 |
| -37.1742 | 12.77678 | 55.63191 |
| -33.9033 | 12.64617 | 50.66469 |
| -30.3485 | 12.5307 | 46.27333 |
| -26.224 | 12.41609 | 41.91468 |
| -22.0036 | 12.32407 | 38.41513 |
| -17.247 | 12.23633 | 35.07837 |
| -12.5532 | 12.22232 | 34.54533 |
| -7.36058 | 12.14808 | 31.72191 |
| -2.36023 | 12.09088 | 29.54675 |
| 2.954941 | 12.12085 | 30.68645 |
| 7.892083 | 12.10674 | 30.1498 |
| 13.15229 | 12.17156 | 32.61497 |
| 17.88793 | 12.20528 | 33.89719 |
| 22.72917 | 12.28258 | 36.83705 |
| 27.05672 | 12.37332 | 40.28822 |
| 31.41121 | 12.4973 | 45.00324 |
| 35.02615 | 12.56186 | 47.45838 |
| 38.48223 | 12.69611 | 52.56401 |
| -37.1245 | 13.09381 | 54.75462 |
| -33.9481 | 12.99415 | 51.06307 |
| -30.3447 | 12.85888 | 46.052 |
| -26.2297 | 12.73957 | 41.63271 |
| -22.0224 | 12.65723 | 38.58255 |
| -17.3084 | 12.59309 | 36.2065 |
| -12.5528 | 12.53595 | 34.08997 |
| -7.38651 | 12.47802 | 31.9438 |
| -2.40617 | 12.45944 | 31.25581 |
| 2.930861 | 12.45105 | 30.94478 |
| 7.886278 | 12.43039 | 30.1795 |
| 13.12022 | 12.49079 | 32.41697 |
| 17.87714 | 12.54778 | 34.52798 |
| 22.75185 | 12.63937 | 37.92086 |
| 27.03603 | 12.71052 | 40.5563 |
| 31.38802 | 12.83424 | 45.13927 |
| 35.03536 | 12.91371 | 48.08325 |
| 38.48754 | 13.04693 | 53.01802 |
| -37.2928 | 13.47648 | 56.2917 |
| -33.9218 | 13.31468 | 50.44982 |
| -30.4043 | 13.21257 | 46.76322 |
| -26.267 | 13.0775 | 41.88656 |
| -22.0579 | 12.99909 | 39.05546 |
| -17.3341 | 12.92235 | 36.28465 |
| -12.5645 | 12.86064 | 34.05662 |
| -7.40719 | 12.79714 | 31.76401 |
| -2.41207 | 12.77471 | 30.95423 |
| 2.906485 | 12.7814 | 31.19566 |
| 7.899037 | 12.78653 | 31.3809 |
| 13.09244 | 12.8177 | 32.50642 |
| 17.85814 | 12.88516 | 34.94203 |
| 22.73923 | 12.9781 | 38.29761 |
| 27.03793 | 13.04373 | 40.66719 |
| 31.26445 | 13.13103 | 43.81918 |
| 34.98765 | 13.25529 | 48.30561 |
| 38.54196 | 13.41557 | 54.0925 |
| -37.3638 | 13.84833 | 57.3718 |
| -33.9148 | 13.64296 | 50.14009 |
| -30.5138 | 13.57565 | 47.76972 |
| -26.3233 | 13.42835 | 42.58281 |
| -22.1307 | 13.34354 | 39.59633 |
| -17.346 | 13.24309 | 36.0591 |
| -12.5865 | 13.19798 | 34.47042 |
| -7.42517 | 13.10635 | 31.24391 |
| -2.4553 | 13.12298 | 31.82962 |
| 2.87962 | 13.103 | 31.12592 |
| 7.854115 | 13.09532 | 30.85553 |
| 13.0739 | 13.15213 | 32.85591 |
| 17.82453 | 13.21165 | 34.95211 |
| 22.6921 | 13.29853 | 38.01123 |
| 26.98123 | 13.3471 | 39.72166 |
| 31.22258 | 13.45914 | 43.66708 |
| 34.96895 | 13.58566 | 48.12219 |
| 38.57988 | 13.77977 | 54.95751 |
| -37.3545 | 14.1793 | 56.99521 |
| -33.9546 | 13.98822 | 50.4287 |
| -30.5001 | 13.88407 | 46.84951 |
| -26.3375 | 13.75802 | 42.51774 |
| -22.1262 | 13.68231 | 39.91602 |
| -17.3608 | 13.56316 | 35.82111 |
| -12.6321 | 13.5276 | 34.5991 |
| -7.46144 | 13.44944 | 31.91321 |
| -2.49496 | 13.45802 | 32.20806 |
| 2.857765 | 13.4469 | 31.82605 |
| 7.816652 | 13.41641 | 30.77818 |
| 13.06017 | 13.49256 | 33.39504 |
| 17.80869 | 13.55138 | 35.41647 |
| 22.62137 | 13.60232 | 37.16698 |
| 27.01701 | 13.71203 | 40.9373 |
| 31.24475 | 13.81444 | 44.45648 |
| 34.95033 | 13.92855 | 48.37793 |
| 38.54964 | 14.12202 | 55.02654 |
| -37.3371 | 14.50586 | 56.48813 |
| -34.0048 | 14.33938 | 50.90154 |
| -30.4854 | 14.22068 | 46.91822 |
| -26.3412 | 14.08022 | 42.205 |
| -22.1209 | 13.98237 | 38.92143 |
| -17.375 | 13.88272 | 35.57742 |
| -12.6398 | 13.84993 | 34.47717 |
| -7.48035 | 13.76311 | 31.56394 |
| -2.4957 | 13.76359 | 31.57981 |
| 2.822239 | 13.74656 | 31.00854 |
| 7.785383 | 13.74111 | 30.82546 |
| 13.01469 | 13.79855 | 32.75292 |
| 17.78915 | 13.86575 | 35.00819 |
| 22.61407 | 13.94351 | 37.61745 |
| 27.00092 | 14.05003 | 41.19197 |
| 31.23921 | 14.16015 | 44.887 |
| 34.90317 | 14.25987 | 48.23349 |
| 38.56541 | 14.48069 | 55.64348 |
| -37.3083 | 14.82779 | 55.85237 |
| -34.0141 | 14.67309 | 50.78058 |
| -30.6199 | 14.5947 | 48.21065 |
| -26.3594 | 14.41032 | 42.16579 |
| -22.1543 | 14.32439 | 39.3485 |
| -17.4183 | 14.22572 | 36.11342 |
| -12.6441 | 14.17208 | 34.35492 |
| -7.51938 | 14.11433 | 32.46156 |
| -2.50407 | 14.08912 | 31.63513 |
| 2.79047 | 14.05246 | 30.43299 |
| 7.785358 | 14.07045 | 31.02298 |
| 12.98664 | 14.12819 | 32.916 |
| 17.71519 | 14.18556 | 34.79681 |
| 22.62804 | 14.30035 | 38.5602 |
| 26.94145 | 14.36653 | 40.72984 |
| 31.25435 | 14.51438 | 45.57729 |
| 34.96055 | 14.63383 | 49.49347 |
| 38.59658 | 14.84579 | 56.44275 |
| -37.3371 | 15.18827 | 56.48114 |
| -34.0524 | 15.02035 | 51.09954 |
| -30.5983 | 14.92861 | 48.15945 |
| -26.4195 | 14.76287 | 42.84745 |
| -22.2008 | 14.67533 | 40.04201 |
| -17.4489 | 14.55901 | 36.31412 |
| -12.6933 | 14.50288 | 34.51517 |
| -7.54066 | 14.43283 | 32.27023 |
| -2.54086 | 14.39838 | 31.16618 |
| 2.769037 | 14.40266 | 31.30328 |
| 7.757952 | 14.41332 | 31.64514 |
| 12.96095 | 14.45685 | 33.04003 |
| 17.69603 | 14.49831 | 34.36874 |
| 22.59861 | 14.62848 | 38.54065 |
| 26.90344 | 14.71476 | 41.30584 |
| 31.16935 | 14.82399 | 44.80641 |
| 34.99025 | 14.98319 | 49.90853 |
| 38.4251 | 15.13451 | 54.75814 |
| -37.3789 | 15.53939 | 56.78852 |
| -34.0729 | 15.36058 | 51.18401 |
| -30.5418 | 15.22649 | 46.98108 |
| -26.44 | 15.095 | 42.85936 |
| -22.2416 | 15.00007 | 39.88406 |
| -17.4771 | 14.89296 | 36.52664 |
| -12.7254 | 14.81707 | 34.148 |
| -7.55857 | 14.74688 | 31.94795 |
| -2.55301 | 14.73833 | 31.68011 |
| 2.74189 | 14.71662 | 30.99953 |
| 7.721618 | 14.73604 | 31.60827 |
| 12.97 | 14.82147 | 34.28578 |
| 17.65374 | 14.82118 | 34.27674 |
| 22.5719 | 14.96156 | 38.67679 |
| 26.90485 | 15.04729 | 41.36413 |
| 31.13482 | 15.15603 | 44.77234 |
| 34.82418 | 15.27665 | 48.553 |
| 38.33829 | 15.45166 | 54.03862 |
| -37.4395 | 15.89797 | 57.31162 |
| -34.1045 | 15.70652 | 51.43985 |
| -30.6 | 15.58118 | 47.5957 |
| -26.4608 | 15.42675 | 42.85928 |
| -22.2545 | 15.35112 | 40.53957 |
| -17.5034 | 15.22438 | 36.65242 |
| -12.7246 | 15.12863 | 33.7159 |
| -7.59452 | 15.09032 | 32.54087 |
| -2.59611 | 15.08143 | 32.2684 |
| 2.718939 | 15.06214 | 31.67677 |
| 7.719532 | 15.0641 | 31.7367 |
| 12.95732 | 15.16471 | 34.82252 |
| 17.66142 | 15.18557 | 35.46226 |
| 22.52757 | 15.28286 | 38.44622 |
| 26.89872 | 15.39554 | 41.90213 |
| 31.15256 | 15.5137 | 45.52587 |
| 34.7838 | 15.60911 | 48.45208 |
| 38.26134 | 15.77291 | 53.47603 |
| -37.5022 | 16.25817 | 57.86134 |
| -34.1218 | 16.04445 | 51.44439 |
| -30.6373 | 15.91143 | 47.45066 |
| -26.5223 | 15.78404 | 43.62576 |
| -22.2966 | 15.67883 | 40.46687 |
| -17.5487 | 15.57466 | 37.33939 |
| -12.7427 | 15.46682 | 34.1016 |
| -7.62485 | 15.41936 | 32.67661 |
| -2.59764 | 15.38657 | 31.69191 |
| 2.693277 | 15.3884 | 31.747 |
| 7.684624 | 15.39885 | 32.06061 |
| 12.91302 | 15.4691 | 34.16984 |
| 17.64063 | 15.52619 | 35.88413 |
| 22.5107 | 15.6219 | 38.75778 |
| 26.84963 | 15.71729 | 41.62156 |
| 31.10546 | 15.84044 | 45.31929 |
| 34.82064 | 15.96301 | 48.99936 |
| 38.20397 | 16.10125 | 53.14991 |
| -37.5305 | 16.60426 | 57.97346 |
| -34.2238 | 16.42124 | 52.59153 |
| -30.7086 | 16.29572 | 48.90076 |
| -26.5495 | 16.12055 | 43.74976 |
| -22.3205 | 16.01683 | 40.69987 |
| -17.5746 | 15.90699 | 37.47 |
| -12.8062 | 15.81946 | 34.89601 |
| -7.64653 | 15.73763 | 32.48996 |
| -2.63286 | 15.68292 | 30.88114 |
| 2.664 | 15.7139 | 31.79212 |
| 7.60733 | 15.69971 | 31.37495 |
| 12.8965 | 15.80997 | 34.61694 |
| 17.62112 | 15.86784 | 36.31886 |
| 22.48536 | 15.95473 | 38.87367 |
| 26.79377 | 16.03444 | 41.21764 |
| 31.03285 | 16.15128 | 44.65337 |
| 34.70768 | 16.27639 | 48.33241 |
| 38.33076 | 16.50781 | 55.13724 |
| -37.4876 | 16.93256 | 57.56842 |
| -34.2098 | 16.74502 | 52.16516 |
| -30.7122 | 16.60751 | 48.20329 |
| -26.5711 | 16.45344 | 43.7643 |
| -22.3274 | 16.34172 | 40.54563 |
| -17.5818 | 16.22047 | 37.05214 |
| -12.8221 | 16.1547 | 35.15729 |
| -7.66577 | 16.04986 | 32.13679 |
| -2.67954 | 16.04226 | 31.91773 |
| 2.639826 | 16.05169 | 32.18933 |
| 7.60235 | 16.02572 | 31.44129 |
| 12.84559 | 16.10924 | 33.84754 |
| 17.60757 | 16.21284 | 36.83232 |
| 22.4531 | 16.2819 | 38.82212 |
| 26.80703 | 16.3933 | 42.03159 |
| 31.1535 | 16.56563 | 46.99658 |
| 34.77259 | 16.64493 | 49.28144 |
| 38.21066 | 16.8115 | 54.08055 |
| -37.594 | 17.31462 | 58.69769 |
| -34.269 | 17.10277 | 52.71501 |
| -30.6697 | 16.91214 | 47.33133 |
| -26.5807 | 16.7801 | 43.60256 |
| -22.3526 | 16.65685 | 40.12174 |
| -17.6043 | 16.55033 | 37.11373 |
| -12.8247 | 16.47178 | 34.89537 |
| -7.70292 | 16.39909 | 32.84251 |
| -2.72271 | 16.40084 | 32.89195 |
| 2.613182 | 16.3703 | 32.02946 |
| 7.596536 | 16.35013 | 31.45989 |
| 12.79717 | 16.41022 | 33.15671 |
| 17.62281 | 16.58787 | 38.17378 |
| 22.43534 | 16.62093 | 39.10737 |
| 26.76872 | 16.7239 | 42.01531 |
| 31.12725 | 16.9014 | 47.02816 |
| 34.71441 | 16.96775 | 48.90189 |
| 38.20827 | 17.16396 | 54.44307 |
| -37.6643 | 17.66596 | 58.93245 |
| -34.3556 | 17.47655 | 53.68722 |
| -30.7245 | 17.26904 | 47.94078 |
| -26.6392 | 17.13529 | 44.23705 |
| -22.3516 | 17.00134 | 40.52762 |
| -17.618 | 16.87118 | 36.92309 |
| -12.8903 | 16.82491 | 35.64177 |
| -7.73928 | 16.75186 | 33.61894 |
| -2.73367 | 16.7537 | 33.66972 |
| 2.585461 | 16.68773 | 31.84283 |
| 7.566719 | 16.68709 | 31.8253 |
| 12.76709 | 16.73569 | 33.17114 |
| 17.58926 | 16.88633 | 37.34275 |
| 22.43799 | 16.97498 | 39.79763 |
| 26.72382 | 17.04977 | 41.86855 |
| 31.10093 | 17.24013 | 47.14018 |
| 34.64251 | 17.30164 | 48.84359 |
| 38.35739 | 17.58614 | 56.72197 |
| -37.6894 | 18.01215 | 59.01846 |
| -34.342 | 17.80191 | 53.30728 |
| -30.7817 | 17.61001 | 48.09422 |
| -26.6501 | 17.46246 | 44.08592 |
| -22.4466 | 17.37031 | 41.58284 |
| -17.6327 | 17.19159 | 36.72786 |
| -12.8922 | 17.146 | 35.48946 |
| -7.76432 | 17.07492 | 33.55851 |
| -2.7353 | 17.04947 | 32.8672 |
| 2.556034 | 16.99945 | 31.5085 |
| 7.545419 | 17.04376 | 32.71218 |
| 12.77668 | 17.1158 | 34.66901 |
| 17.58143 | 17.20902 | 37.20142 |
| 22.43028 | 17.32095 | 40.24186 |
| 26.73395 | 17.39106 | 42.14652 |
| 31.07549 | 17.57872 | 47.24413 |
| 34.64807 | 17.64251 | 48.97702 |
| 38.27262 | 17.90314 | 56.05698 |
| -37.7529 | 18.39263 | 60.0151 |
| -34.3508 | 18.13617 | 53.1786 |
| -30.7822 | 17.9563 | 48.38385 |
| -26.6469 | 17.77949 | 43.67042 |
| -22.4714 | 17.7088 | 41.78616 |
| -17.6613 | 17.52826 | 36.97343 |
| -12.9185 | 17.44914 | 34.86424 |
| -7.7812 | 17.38862 | 33.25114 |
| -2.76676 | 17.35081 | 32.24324 |
| 2.530966 | 17.34208 | 32.01052 |
| 7.511392 | 17.36976 | 32.74831 |
| 12.75573 | 17.45275 | 34.96066 |
| 17.53496 | 17.52881 | 36.98803 |
| 22.40138 | 17.6504 | 40.22927 |
| 26.72897 | 17.73858 | 42.58007 |
| 30.98767 | 17.88061 | 46.36599 |
| 34.68635 | 18.01733 | 50.01064 |
| 38.2508 | 18.24977 | 56.20682 |
| -37.7308 | 18.71645 | 59.49248 |
| -34.428 | 18.50736 | 54.02112 |
| -30.7969 | 18.27222 | 47.86792 |
| -26.7172 | 18.14494 | 44.53719 |
| -22.4513 | 18.01524 | 41.14334 |
| -17.71 | 17.88204 | 37.65761 |
| -12.9833 | 17.84916 | 36.79724 |
| -7.802 | 17.7049 | 33.0223 |
| -2.80783 | 17.68065 | 32.38767 |
| 2.510186 | 17.69329 | 32.71837 |
| 7.484858 | 17.71936 | 33.4008 |
| 12.71621 | 17.76274 | 34.53583 |
| 17.47027 | 17.83182 | 36.34351 |
| 22.32527 | 17.94524 | 39.31146 |
| 26.68592 | 18.06579 | 42.46614 |
| 30.93947 | 18.2071 | 46.16384 |
| 34.59965 | 18.32592 | 49.27327 |
| 38.18265 | 18.57402 | 55.76552 |
| -37.6258 | 18.99641 | 57.86181 |
| -34.4054 | 18.82455 | 53.44555 |
| -30.8337 | 18.63872 | 48.67036 |
| -26.7367 | 18.47917 | 44.57052 |
| -22.483 | 18.35898 | 41.4821 |
| -17.761 | 18.24078 | 38.44481 |
| -13.0054 | 18.19441 | 37.25326 |
| -7.83445 | 18.04874 | 33.51005 |
| -2.84994 | 18.01897 | 32.74502 |
| 2.484728 | 18.01112 | 32.54338 |
| 7.428027 | 17.9938 | 32.09829 |
| 12.69456 | 18.10023 | 34.833 |
| 17.43297 | 18.1575 | 36.3048 |
| 22.31157 | 18.28849 | 39.67087 |
| 26.72006 | 18.44592 | 43.71619 |
| 30.94755 | 18.5663 | 46.80943 |
| 34.66262 | 18.7136 | 50.59457 |
| 38.19195 | 18.93466 | 56.27517 |
| -37.6187 | 19.32482 | 57.51187 |
| -34.4564 | 19.18226 | 53.91338 |
| -30.9132 | 18.99299 | 49.13576 |
| -26.728 | 18.79181 | 44.05778 |
| -22.5072 | 18.69821 | 41.69504 |
| -17.7809 | 18.56381 | 38.30252 |
| -13.0371 | 18.50992 | 36.94219 |
| -7.87508 | 18.41305 | 34.49725 |
| -2.89611 | 18.39278 | 33.9854 |
| 2.45851 | 18.34772 | 32.8482 |
| 7.388631 | 18.31446 | 32.00849 |
| 12.6533 | 18.40859 | 34.38468 |
| 17.42517 | 18.51445 | 37.05677 |
| 22.34174 | 18.66945 | 40.96923 |
| 26.65547 | 18.75692 | 43.17689 |
| 30.88534 | 18.88015 | 46.28748 |
| 34.73724 | 19.10904 | 52.06512 |
| 38.18988 | 19.29041 | 56.64323 |
| -37.6748 | 19.70371 | 58.42585 |
| -34.5781 | 19.57894 | 55.33132 |
| -30.8992 | 19.33009 | 49.15917 |
| -26.7364 | 19.11518 | 43.82885 |
| -22.5631 | 19.03754 | 41.9031 |
| -17.7689 | 18.85615 | 37.4042 |
| -13.0679 | 18.82282 | 36.57756 |
| -7.91569 | 18.76917 | 35.24682 |
| -2.86794 | 18.68033 | 33.04337 |
| 2.430469 | 18.64942 | 32.2766 |
| 7.407532 | 18.69534 | 33.41567 |
| 12.6403 | 18.75454 | 34.88402 |
| 17.38676 | 18.84104 | 37.02947 |
| 22.28522 | 18.97475 | 40.34585 |
| 26.60377 | 19.07741 | 42.892 |
| 30.90683 | 19.24703 | 47.09902 |
| 34.57083 | 19.37403 | 50.24899 |
| 38.14064 | 19.62074 | 56.36815 |
| -37.775 | 20.07249 | 59.06246 |
| -34.511 | 19.87267 | 54.19114 |
| -30.9403 | 19.66121 | 49.03597 |
| -26.7963 | 19.47852 | 44.58214 |
| -22.5922 | 19.38141 | 42.21475 |
| -17.7937 | 19.18713 | 37.47834 |
| -13.0666 | 19.13596 | 36.23099 |
| -7.93681 | 19.0841 | 34.96679 |
| -2.91008 | 19.03525 | 33.77586 |
| 2.40252 | 18.97565 | 32.32289 |
| 7.38098 | 19.04568 | 34.03012 |
| 12.60771 | 19.07555 | 34.75824 |
| 17.35288 | 19.16698 | 36.98714 |
| 22.27555 | 19.3212 | 40.74689 |
| 26.56261 | 19.40083 | 42.68807 |
| 30.86879 | 19.58121 | 47.08568 |
| 34.59034 | 19.74276 | 51.02394 |
| 38.10064 | 19.95773 | 56.26479 |
| -37.8787 | 20.46122 | 60.15558 |
| -34.5407 | 20.22062 | 54.3888 |
| -30.9425 | 20.00865 | 49.30803 |
| -26.8053 | 19.80244 | 44.36529 |
| -22.6043 | 19.71088 | 42.17065 |
| -17.8163 | 19.51928 | 37.57827 |
| -13.073 | 19.45648 | 36.07302 |
| -7.96404 | 19.41118 | 34.98716 |
| -2.95433 | 19.37539 | 34.12948 |
| 2.37975 | 19.34707 | 33.45066 |
| 7.382664 | 19.38721 | 34.41276 |
| 12.59076 | 19.42176 | 35.24079 |
| 17.34488 | 19.52745 | 37.77408 |
| 22.25642 | 19.66066 | 40.96701 |
| 26.50356 | 19.71719 | 42.32192 |
| 30.86695 | 19.93432 | 47.52647 |
| 34.62714 | 20.12203 | 52.02561 |
| 38.09669 | 20.3181 | 56.72521 |
| -37.8592 | 20.78358 | 59.64831 |
| -34.5968 | 20.58372 | 54.93695 |
| -31.0071 | 20.37632 | 50.04784 |
| -26.8365 | 20.14183 | 44.52028 |
| -22.629 | 20.05124 | 42.38489 |
| -17.8776 | 19.89189 | 38.62858 |
| -13.1057 | 19.77184 | 35.79853 |
| -7.97712 | 19.71383 | 34.43124 |
| -2.99526 | 19.71745 | 34.51658 |
| 2.36168 | 19.73041 | 34.82209 |
| 7.331268 | 19.67771 | 33.57981 |
| 12.58276 | 19.77576 | 35.89105 |
| 17.3649 | 19.87955 | 38.33773 |
| 22.24389 | 20.0061 | 41.32076 |
| 26.5136 | 20.08311 | 43.13615 |
| 30.8542 | 20.28278 | 47.84305 |
| 34.57377 | 20.44919 | 51.76565 |
| 38.09929 | 20.67807 | 57.16112 |
| -37.8881 | 21.15061 | 60.19312 |
| -34.5495 | 20.88611 | 54.05957 |
| -31.0957 | 20.74001 | 50.67154 |
| -26.8695 | 20.48721 | 44.80918 |
| -22.6611 | 20.40189 | 42.83065 |
| -17.9399 | 20.26442 | 39.64291 |
| -13.1653 | 20.1273 | 36.46312 |
| -8.00763 | 20.05476 | 34.78082 |
| -2.99937 | 20.04237 | 34.4936 |
| 2.326122 | 20.00991 | 33.74078 |
| 7.28906 | 19.99968 | 33.50362 |
| 12.57403 | 20.13561 | 36.65585 |
| 17.30855 | 20.18148 | 37.71942 |
| 22.18842 | 20.31622 | 40.8441 |
| 26.50962 | 20.43847 | 43.67893 |
| 30.77526 | 20.58585 | 47.09654 |
| 34.58228 | 20.81587 | 52.43065 |
| 38.06508 | 21.02017 | 57.16835 |
| -37.8645 | 21.49026 | 60.09592 |
| -34.5432 | 21.21017 | 53.70469 |
| -31.0764 | 21.05857 | 50.2454 |
| -26.8012 | 20.75416 | 43.29917 |
| -22.7488 | 20.77568 | 43.79016 |
| -17.9656 | 20.60014 | 39.78475 |
| -13.1835 | 20.51557 | 37.85488 |
| -8.03745 | 20.40042 | 35.22737 |
| -3.00605 | 20.36695 | 34.46354 |
| 2.295106 | 20.30888 | 33.13855 |
| 7.257854 | 20.34178 | 33.8892 |
| 12.54406 | 20.45888 | 36.56135 |
| 17.26951 | 20.46728 | 36.75311 |
| 22.14667 | 20.63729 | 40.63239 |
| 26.47091 | 20.76774 | 43.60907 |
| 30.80164 | 20.95934 | 47.98116 |
| 34.54705 | 21.15377 | 52.41782 |
| 38.02985 | 21.36025 | 57.12932 |
| -37.9499 | 21.85254 | 60.50999 |
| -34.5741 | 21.55811 | 53.89722 |
| -31.0932 | 21.39746 | 50.2892 |
| -26.9411 | 21.1814 | 45.43671 |
| -22.7694 | 21.11383 | 43.91921 |
| -17.9591 | 20.89592 | 39.02499 |
| -13.1939 | 20.79644 | 36.79078 |
| -8.06062 | 20.72225 | 35.1246 |
| -3.0385 | 20.64618 | 33.41622 |
| 2.272502 | 20.68781 | 34.35119 |
| 7.260974 | 20.68566 | 34.30285 |
| 12.49333 | 20.75076 | 35.76498 |
| 17.2603 | 20.86811 | 38.40038 |
| 22.11983 | 20.97404 | 40.77959 |
| 26.45569 | 21.11512 | 43.94805 |
| 30.76965 | 21.29475 | 47.98258 |
| 34.49122 | 21.47958 | 52.13357 |
| 37.96204 | 21.688 | 56.81464 |
| -37.8685 | 22.15735 | 59.64038 |
| -34.6612 | 21.94162 | 54.87051 |
| -31.1229 | 21.72157 | 50.00494 |
| -26.9487 | 21.50604 | 45.23943 |
| -22.7644 | 21.40438 | 42.99162 |
| -17.9704 | 21.21101 | 38.7159 |
| -13.2331 | 21.12448 | 36.80265 |
| -8.08795 | 21.0528 | 35.21771 |
| -3.079 | 20.97845 | 33.57378 |
| 2.247283 | 21.01534 | 34.38942 |
| 7.239733 | 21.04884 | 35.13025 |
| 12.4652 | 21.08342 | 35.89485 |
| 17.21506 | 21.1854 | 38.1498 |
| 22.13073 | 21.34521 | 41.68316 |
| 26.42009 | 21.4482 | 43.96058 |
| 30.72398 | 21.6202 | 47.76348 |
| 34.39166 | 21.777 | 51.23055 |
| 37.9442 | 22.0386 | 57.01486 |
| -37.8979 | 22.50515 | 59.73363 |
| -34.6952 | 22.28929 | 55.03348 |
| -31.1249 | 22.07222 | 50.30713 |
| -26.9619 | 21.83594 | 45.16255 |
| -22.7726 | 21.7331 | 42.9235 |
| -18.0522 | 21.61231 | 40.29341 |
| -13.3104 | 21.50554 | 37.96873 |
| -8.10618 | 21.35854 | 34.76803 |
| -3.0913 | 21.34237 | 34.41581 |
| 2.216738 | 21.3279 | 34.10085 |
| 7.230388 | 21.36245 | 34.85312 |
| 12.43698 | 21.41307 | 35.95527 |
| 17.18897 | 21.48765 | 37.57911 |
| 22.06697 | 21.64394 | 40.98201 |
| 26.42302 | 21.76077 | 43.52579 |
| 30.68758 | 21.95174 | 47.68386 |
| 34.37256 | 22.12592 | 51.4764 |
| 37.89493 | 22.37187 | 56.83159 |
| -37.9975 | 22.87846 | 60.37092 |
| -34.6654 | 22.59806 | 54.35739 |
| -31.1655 | 22.40251 | 50.16379 |
| -27.0042 | 22.1873 | 45.54837 |
| -22.7968 | 22.07547 | 43.14999 |
| -18.0814 | 21.95043 | 40.46848 |
| -13.2775 | 21.81653 | 37.59671 |
| -8.12823 | 21.67321 | 34.52321 |
| -3.1339 | 21.69611 | 35.01425 |
| 2.189568 | 21.64797 | 33.98182 |
| 7.178766 | 21.64555 | 33.92994 |
| 12.40558 | 21.73608 | 35.87143 |
| 17.14918 | 21.80854 | 37.42544 |
| 22.07453 | 22.01208 | 41.7906 |
| 26.36404 | 22.12462 | 44.20402 |
| 30.70192 | 22.3189 | 48.37054 |
| 34.40445 | 22.48434 | 51.91858 |
| 37.90702 | 22.74081 | 57.41892 |
| -37.9899 | 23.20761 | 60.05636 |
| -34.6757 | 22.9337 | 54.26909 |
| -31.1603 | 22.74789 | 50.34336 |
| -27.0304 | 22.52517 | 45.63761 |
| -22.802 | 22.40083 | 43.01054 |
| -18.1069 | 22.28786 | 40.62359 |
| -13.349 | 22.18918 | 38.53883 |
| -8.16015 | 22.01819 | 34.92607 |
| -3.17219 | 22.02046 | 34.97388 |
| 2.160378 | 21.95803 | 33.655 |
| 7.149523 | 21.99134 | 34.35864 |
| 12.37711 | 22.06149 | 35.84077 |
| 17.13185 | 22.15968 | 37.91552 |
| 22.01736 | 22.31344 | 41.1642 |
| 26.42515 | 22.51057 | 45.3291 |
| 30.68949 | 22.67095 | 48.71778 |
| 34.36894 | 22.80236 | 51.49419 |
| 37.83103 | 23.05615 | 56.85625 |
| -37.997 | 23.56544 | 60.34812 |
| -34.7177 | 23.28842 | 54.58057 |
| -31.2224 | 23.09222 | 50.49582 |
| -27.0995 | 22.8997 | 46.48764 |
| -22.8122 | 22.73022 | 42.95898 |
| -18.0753 | 22.55154 | 39.23899 |
| -13.3392 | 22.48768 | 37.9094 |
| -8.18697 | 22.3544 | 35.13451 |
| -3.17565 | 22.32094 | 34.43781 |
| 2.138304 | 22.33079 | 34.64297 |
| 7.124396 | 22.35561 | 35.15964 |
| 12.34464 | 22.38169 | 35.7026 |
| 17.08568 | 22.47856 | 37.71954 |
| 21.9899 | 22.6478 | 41.24301 |
| 26.44013 | 22.85714 | 45.60157 |
| 30.59672 | 22.95896 | 47.72129 |
| 34.21302 | 23.1017 | 50.69323 |
| 37.85242 | 23.43242 | 57.57867 |
| -38.1568 | 23.99737 | 62.15212 |
| -34.7985 | 23.67042 | 55.44288 |
| -31.2147 | 23.43974 | 50.70943 |
| -27.1163 | 23.23218 | 46.4501 |
| -22.8584 | 23.06096 | 42.93673 |
| -18.1046 | 22.89086 | 39.44626 |
| -13.3415 | 22.80206 | 37.62407 |
| -8.21987 | 22.70635 | 35.65994 |
| -3.22123 | 22.66982 | 34.91038 |
| 2.115265 | 22.66645 | 34.84118 |
| 7.113842 | 22.66759 | 34.86456 |
| 12.32754 | 22.73345 | 36.21611 |
| 17.08071 | 22.80613 | 37.70751 |
| 21.98312 | 23.00132 | 41.71285 |
| 26.28432 | 23.11757 | 44.09842 |
| 30.66659 | 23.3728 | 49.33568 |
| 34.32658 | 23.51392 | 52.2315 |
| 37.87906 | 23.81236 | 58.3556 |
| -38.1119 | 24.3027 | 61.34384 |
| -34.7806 | 23.98735 | 54.96446 |
| -31.3211 | 23.82073 | 51.59403 |
| -27.1503 | 23.58141 | 46.75267 |
| -22.9209 | 23.40862 | 43.25732 |
| -18.1413 | 23.24225 | 39.89176 |
| -13.439 | 23.17024 | 38.43502 |
| -8.24376 | 23.02968 | 35.5916 |
| -3.22819 | 22.98808 | 34.75004 |
| 2.08273 | 22.96568 | 34.29688 |
| 7.083736 | 23.0145 | 35.28464 |
| 12.32246 | 23.10469 | 37.10909 |
| 17.0819 | 23.18806 | 38.79557 |
| 21.93058 | 23.30761 | 41.21386 |
| 26.30188 | 23.46848 | 44.46821 |
| 30.66408 | 23.73222 | 49.80354 |
| 34.26995 | 23.83462 | 51.87487 |
| 37.73839 | 24.08768 | 56.99409 |
| -38.1856 | 24.68399 | 62.07324 |
| -34.8065 | 24.33285 | 55.06936 |
| -31.3281 | 24.17809 | 51.98235 |
| -27.151 | 23.89742 | 46.38417 |
| -22.8957 | 23.73366 | 43.11767 |
| -18.1658 | 23.57552 | 39.9635 |
| -13.4634 | 23.52918 | 39.03906 |
| -8.26495 | 23.34921 | 35.44931 |
| -3.26393 | 23.27598 | 33.98874 |
| 2.054014 | 23.291 | 34.28821 |
| 7.045975 | 23.34271 | 35.31972 |
| 12.2772 | 23.40959 | 36.65373 |
| 17.04306 | 23.46749 | 37.80863 |
| 21.90363 | 23.64406 | 41.33061 |
| 26.26528 | 23.79952 | 44.43135 |
| 30.60292 | 24.04768 | 49.38121 |
| 34.19423 | 24.16588 | 51.73892 |
| 37.81653 | 24.50271 | 58.45745 |
| -38.2018 | 25.00674 | 61.63106 |
| -34.8495 | 24.68776 | 55.35624 |
| -31.3638 | 24.5046 | 51.75334 |
| -27.1782 | 24.23957 | 46.53993 |
| -22.9349 | 24.05893 | 42.98643 |
| -18.2004 | 23.92153 | 40.2837 |
| -13.4699 | 23.84953 | 38.86738 |
| -8.30107 | 23.70055 | 35.93676 |
| -3.31015 | 23.67947 | 35.52211 |
| 2.029227 | 23.6339 | 34.62567 |
| 7.02259 | 23.69548 | 35.83712 |
| 12.23853 | 23.72089 | 36.33687 |
| 17.03355 | 23.83158 | 38.51429 |
| 21.91156 | 24.01811 | 42.18359 |
| 26.24662 | 24.15129 | 44.80343 |
| 30.55541 | 24.37437 | 49.19157 |
| 34.20509 | 24.53482 | 52.3478 |
| 37.71345 | 24.80038 | 57.57176 |
| -38.1325 | 25.31555 | 60.93028 |
| -34.8105 | 24.98938 | 54.60151 |
| -31.3336 | 24.78329 | 50.6026 |
| -27.184 | 24.56165 | 46.30201 |
| -22.9578 | 24.43788 | 43.90038 |
| -18.2518 | 24.28943 | 41.02006 |
| -13.4912 | 24.20168 | 39.3173 |
| -8.33201 | 24.04451 | 36.26778 |
| -3.31086 | 23.99405 | 35.28866 |
| 2.002105 | 23.95261 | 34.48451 |
| 6.989342 | 24.03987 | 36.17763 |
| 12.22678 | 24.08679 | 37.08817 |
| 16.99386 | 24.19708 | 39.2282 |
| 21.8783 | 24.34869 | 42.1699 |
| 26.20503 | 24.47931 | 44.7044 |
| 30.49839 | 24.68595 | 48.71394 |
| 34.18851 | 24.88551 | 52.58613 |
| 37.72204 | 25.17161 | 58.13738 |
| -38.1123 | 25.63471 | 60.44663 |
| -34.9147 | 25.39106 | 55.78239 |
| -31.3843 | 25.17636 | 51.67248 |
| -27.1965 | 24.88565 | 46.10734 |
| -23.0267 | 24.79436 | 44.35975 |
| -18.2709 | 24.61698 | 40.96418 |
| -13.5375 | 24.53032 | 39.30516 |
| -8.36384 | 24.39154 | 36.64858 |
| -3.35123 | 24.33003 | 35.47108 |
| 1.978933 | 24.30107 | 34.91663 |
| 6.977293 | 24.35031 | 35.85936 |
| 12.24503 | 24.51009 | 38.91791 |
| 16.99498 | 24.58172 | 40.28912 |
| 21.88416 | 24.71854 | 42.90824 |
| 26.20735 | 24.84636 | 45.35528 |
| 30.55976 | 25.09785 | 50.16939 |
| 34.17804 | 25.2206 | 52.51935 |
| 37.71648 | 25.53297 | 58.49904 |
| -38.2203 | 26.03956 | 61.5944 |
| -34.9218 | 25.7246 | 55.64495 |
| -31.3939 | 25.51057 | 51.60195 |
| -27.2617 | 25.26414 | 46.947 |
| -23.0753 | 25.13089 | 44.42999 |
| -18.3002 | 24.95242 | 41.05886 |
| -13.5526 | 24.86635 | 39.433 |
| -8.38508 | 24.71482 | 36.57075 |
| -3.36325 | 24.68029 | 35.91835 |
| 1.955451 | 24.63856 | 35.13008 |
| 6.932933 | 24.64419 | 35.23641 |
| 12.20091 | 24.80953 | 38.35968 |
| 16.94363 | 24.88941 | 39.86849 |
| 21.82518 | 25.01827 | 42.30277 |
| 26.15008 | 25.15701 | 44.92349 |
| 30.46297 | 25.38451 | 49.22071 |
| 34.13066 | 25.54994 | 52.34565 |
| 37.70164 | 25.89123 | 58.79244 |
| -38.2336 | 26.37904 | 61.49336 |
| -34.8884 | 26.02833 | 54.9552 |
| -31.4162 | 25.85763 | 51.77293 |
| -27.3023 | 25.6159 | 47.26652 |
| -23.0542 | 25.46484 | 44.45036 |
| -18.3273 | 25.28963 | 41.18399 |
| -13.5473 | 25.16441 | 38.84955 |
| -8.42185 | 25.0786 | 37.24985 |
| -3.40208 | 25.01124 | 35.99415 |
| 1.922619 | 24.90021 | 33.92423 |
| 6.940464 | 25.02901 | 36.32537 |
| 12.17083 | 25.13799 | 38.35699 |
| 16.92491 | 25.19812 | 39.47793 |
| 21.80804 | 25.36414 | 42.57312 |
| 26.12885 | 25.5063 | 45.22318 |
| 30.39265 | 25.68424 | 48.5405 |
| 34.08393 | 25.90075 | 52.57691 |
| 37.69137 | 26.24966 | 59.08133 |
| -38.2901 | 26.75226 | 62.01602 |
| -34.984 | 26.42573 | 56.00713 |
| -31.463 | 26.19289 | 51.7224 |
| -27.3348 | 25.96687 | 47.56323 |
| -23.0535 | 25.74762 | 43.52851 |
| -18.3634 | 25.64071 | 41.56118 |
| -13.5942 | 25.50597 | 39.08175 |
| -8.44634 | 25.4062 | 37.24576 |
| -3.44746 | 25.36959 | 36.57205 |
| 1.898035 | 25.26824 | 34.70697 |
| 6.898281 | 25.34146 | 36.05439 |
| 12.1451 | 25.47143 | 38.44607 |
| 16.89988 | 25.54637 | 39.82515 |
| 21.79638 | 25.7171 | 42.96682 |
| 26.14761 | 25.86372 | 45.66498 |
| 30.46424 | 26.10699 | 50.14166 |
| 34.13575 | 26.27836 | 53.29519 |
| 37.66534 | 26.59637 | 59.14728 |
| -38.4836 | 27.22388 | 64.31294 |
| -35.0132 | 26.77705 | 56.19506 |
| -31.4694 | 26.55144 | 52.09619 |
| -27.3634 | 26.30999 | 47.70965 |
| -23.1352 | 26.15603 | 44.91265 |
| -18.3711 | 25.95557 | 41.27075 |
| -13.6129 | 25.85125 | 39.37549 |
| -8.47263 | 25.72996 | 37.17194 |
| -3.48832 | 25.70257 | 36.67434 |
| 1.863372 | 25.58292 | 34.50047 |
| 6.857006 | 25.651 | 35.73733 |
| 12.11666 | 25.80542 | 38.54287 |
| 16.90124 | 25.88156 | 39.92604 |
| 21.80403 | 26.0946 | 43.79645 |
| 26.11502 | 26.19903 | 45.69385 |
| 30.50847 | 26.50844 | 51.31507 |
| 34.1582 | 26.66271 | 54.11775 |
| 37.69974 | 26.9894 | 60.05291 |
| -38.4264 | 27.52075 | 63.41718 |
| -35.1186 | 27.18523 | 57.39829 |
| -31.5178 | 26.88872 | 52.07917 |
| -27.4035 | 26.66438 | 48.05457 |
| -23.1709 | 26.51744 | 45.41861 |
| -18.426 | 26.33181 | 42.08869 |
| -13.6446 | 26.16724 | 39.13646 |
| -8.49936 | 26.05698 | 37.15842 |
| -3.47042 | 26.07141 | 37.41732 |
| 1.842256 | 25.95141 | 35.26461 |
| 6.827118 | 26.01205 | 36.35249 |
| 12.07985 | 26.11915 | 38.27376 |
| 16.85177 | 26.188 | 39.50884 |
| 21.75946 | 26.40706 | 43.43863 |
| 26.07201 | 26.52494 | 45.55316 |
| 30.46605 | 26.83832 | 51.17502 |
| 34.04305 | 26.96023 | 53.36188 |
| 37.63382 | 27.31029 | 59.64173 |
| -38.4236 | 27.82969 | 62.75749 |
| -35.0954 | 27.49546 | 56.83615 |
| -31.5391 | 27.23264 | 52.18006 |
| -27.43 | 27.00717 | 48.18558 |
| -23.1455 | 26.76777 | 43.94436 |
| -18.4714 | 26.69608 | 42.67417 |
| -13.7049 | 26.53002 | 39.73221 |
| -8.53771 | 26.4243 | 37.8592 |
| -3.50483 | 26.37832 | 37.04472 |
| 1.822247 | 26.36545 | 36.8167 |
| 6.808098 | 26.40706 | 37.5539 |
| 12.04742 | 26.44274 | 38.18594 |
| 16.82058 | 26.52597 | 39.66055 |
| 21.73964 | 26.75377 | 43.69623 |
| 26.071 | 26.89321 | 46.16661 |
| 30.36985 | 27.12483 | 50.26997 |
| 34.01487 | 27.27696 | 52.96519 |
| 37.63507 | 27.68039 | 60.11254 |
| -38.4221 | 28.18602 | 62.94336 |
| -35.1053 | 27.82998 | 56.71297 |
| -31.6154 | 27.62295 | 53.09023 |
| -27.4948 | 27.38581 | 48.94052 |
| -23.1817 | 27.16611 | 45.0961 |
| -18.483 | 27.01267 | 42.41109 |
| -13.708 | 26.91223 | 40.6535 |
| -8.55362 | 26.72232 | 37.3303 |
| -3.54041 | 26.67058 | 36.42483 |
| 1.797314 | 26.67068 | 36.42665 |
| 6.780557 | 26.76615 | 38.09718 |
| 12.00507 | 26.73983 | 37.63659 |
| 16.77545 | 26.83879 | 39.36834 |
| 21.71229 | 27.09246 | 43.80724 |
| 26.0666 | 27.259 | 46.72157 |
| 30.29008 | 27.42015 | 49.54152 |
| 33.91871 | 27.59272 | 52.56126 |
| 37.625 | 28.04599 | 60.49291 |
| -38.4412 | 28.5053 | 62.4842 |
| -35.1466 | 28.1834 | 56.91955 |
| -31.6507 | 27.95435 | 52.96004 |
| -27.5727 | 27.77921 | 49.93246 |
| -23.2668 | 27.54758 | 45.92831 |
| -18.5414 | 27.40023 | 43.38117 |
| -13.7294 | 27.20513 | 40.00855 |
| -8.57647 | 27.04541 | 37.24741 |
| -3.58509 | 27.01945 | 36.79859 |
| 1.768726 | 26.98846 | 36.26288 |
| 6.774483 | 27.07871 | 37.823 |
| 11.9778 | 27.07711 | 37.79544 |
| 16.75212 | 27.18836 | 39.71862 |
| 21.62026 | 27.34705 | 42.46177 |
| 26.02963 | 27.59005 | 46.6625 |
| 30.35425 | 27.84841 | 51.12876 |
| 33.94775 | 27.95511 | 52.97315 |
| 37.52614 | 28.34735 | 59.7538 |
| -38.468 | 28.88386 | 63.04851 |
| -35.1532 | 28.51354 | 56.72351 |
| -31.6451 | 28.30399 | 53.14444 |
| -27.5349 | 28.05989 | 48.97541 |
| -23.2768 | 27.84047 | 45.22767 |
| -18.5564 | 27.72489 | 43.25358 |
| -13.7676 | 27.5287 | 39.90282 |
| -8.59405 | 27.35206 | 36.8858 |
| -3.58578 | 27.32413 | 36.40879 |
| 1.741711 | 27.34803 | 36.817 |
| 6.744756 | 27.44079 | 38.40132 |
| 11.97183 | 27.45657 | 38.67088 |
| 16.72826 | 27.53806 | 40.06269 |
| 21.6026 | 27.69412 | 42.72815 |
| 26.01586 | 27.95011 | 47.10028 |
| 30.33944 | 28.20554 | 51.46307 |
| 33.99533 | 28.38991 | 54.61197 |
| 37.37612 | 28.60814 | 58.33925 |
| -38.5516 | 29.25373 | 63.45277 |
| -35.1675 | 28.85276 | 56.68544 |
| -31.6864 | 28.6399 | 53.09292 |
| -27.547 | 28.38859 | 48.85143 |
| -23.3224 | 28.21155 | 45.86335 |
| -18.5633 | 28.03067 | 42.81059 |
| -13.8334 | 27.90857 | 40.74977 |
| -8.61648 | 27.66797 | 36.68896 |
| -3.59174 | 27.64349 | 36.27586 |
| 1.713017 | 27.71116 | 37.41791 |
| 6.698959 | 27.7164 | 37.50648 |
| 11.95834 | 27.82758 | 39.38282 |
| 16.70431 | 27.89169 | 40.46493 |
| 21.62134 | 28.09407 | 43.88049 |
| 25.9907 | 28.29697 | 47.3051 |
| 30.22489 | 28.46538 | 50.14742 |
| 34.00212 | 28.78985 | 55.6237 |
| 37.37109 | 28.97312 | 58.71677 |
| -38.583 | 29.61113 | 63.63968 |
| -35.2929 | 29.28546 | 58.20745 |
| -31.6894 | 29.00063 | 53.4565 |
| -27.6462 | 28.80677 | 50.22278 |
| -23.3467 | 28.56239 | 46.14658 |
| -18.5945 | 28.3768 | 43.0509 |
| -13.8339 | 28.21953 | 40.42745 |
| -8.65423 | 28.04199 | 37.46608 |
| -3.64093 | 28.04314 | 37.48536 |
| 1.686874 | 28.06825 | 37.90421 |
| 6.689476 | 28.03984 | 37.43025 |
| 11.94552 | 28.1951 | 40.02002 |
| 16.67036 | 28.22106 | 40.45312 |
| 21.637 | 28.48246 | 44.8133 |
| 25.95961 | 28.64007 | 47.44218 |
| 30.31631 | 28.92106 | 52.12923 |
| 33.97032 | 29.10535 | 55.20312 |
| 37.38192 | 29.35044 | 59.29134 |
| -38.659 | 30.00439 | 64.41343 |
| -35.2799 | 29.59861 | 57.72319 |
| -31.7611 | 29.32932 | 53.28331 |
| -27.6389 | 29.11302 | 49.71718 |
| -23.3409 | 28.87215 | 45.74575 |
| -18.6054 | 28.69208 | 42.77698 |
| -13.8318 | 28.52901 | 40.08841 |
| -8.69316 | 28.40117 | 37.98059 |
| -3.68148 | 28.40534 | 38.04932 |
| 1.662402 | 28.42335 | 38.34621 |
| 6.646499 | 28.3559 | 37.23427 |
| 11.88946 | 28.46179 | 38.98007 |
| 16.64824 | 28.57888 | 40.91063 |
| 21.58639 | 28.78626 | 44.32974 |
| 25.96289 | 28.94325 | 46.91812 |
| 30.22869 | 29.20499 | 51.23339 |
| 33.94744 | 29.45609 | 55.37352 |
| 37.41737 | 29.7507 | 60.23082 |
| -38.7142 | 30.37944 | 64.87271 |
| -35.2591 | 29.90502 | 57.14024 |
| -31.7751 | 29.70009 | 53.80003 |
| -27.6357 | 29.42325 | 49.28786 |
| -23.44 | 29.27712 | 46.90605 |
| -18.6328 | 29.02883 | 42.85929 |
| -13.9 | 28.89956 | 40.75227 |
| -8.71158 | 28.70844 | 37.63721 |
| -3.71962 | 28.72338 | 37.8808 |
| 1.630007 | 28.65901 | 36.83149 |
| 6.604459 | 28.66278 | 36.89298 |
| 11.86501 | 28.80697 | 39.24318 |
| 16.62067 | 28.86242 | 40.14692 |
| 21.56212 | 29.12708 | 44.46054 |
| 25.95085 | 29.30964 | 47.43608 |
| 30.15749 | 29.50457 | 50.61328 |
| 33.94278 | 29.79316 | 55.31705 |
| 37.40539 | 30.11371 | 60.54167 |
| -38.6588 | 30.69082 | 64.29566 |
| -35.3345 | 30.29381 | 57.89787 |
| -31.7882 | 30.03669 | 53.75449 |
| -27.6442 | 29.74755 | 49.09508 |
| -23.4732 | 29.63734 | 47.31909 |
| -18.6187 | 29.29957 | 41.87588 |
| -13.8681 | 29.20305 | 40.3206 |
| -8.74808 | 29.07892 | 38.32028 |
| -3.72669 | 29.04451 | 37.76564 |
| 1.598202 | 28.95349 | 36.29887 |
| 6.573741 | 29.01389 | 37.27233 |
| 11.85562 | 29.18451 | 40.02178 |
| 16.61527 | 29.18833 | 40.08329 |
| 21.57651 | 29.52337 | 45.48234 |
| 25.88509 | 29.6117 | 46.90577 |
| 30.1938 | 29.90941 | 51.70341 |
| 33.89764 | 30.13066 | 55.26876 |
| 37.38239 | 30.46687 | 60.68667 |
| -38.669 | 31.05125 | 64.51304 |
| -35.4133 | 30.68396 | 58.6603 |
| -31.8608 | 30.40735 | 54.25259 |
| -27.7176 | 30.13989 | 49.99072 |
| -23.4688 | 29.95183 | 46.99397 |
| -18.6645 | 29.67053 | 42.51152 |
| -13.9089 | 29.52873 | 40.25199 |
| -8.77658 | 29.41795 | 38.48677 |
| -3.7358 | 29.38613 | 37.97984 |
| 1.575855 | 29.35094 | 37.41899 |
| 6.572286 | 29.3527 | 37.44702 |
| 11.80495 | 29.46374 | 39.2165 |
| 16.57521 | 29.56799 | 40.8777 |
| 21.54981 | 29.86235 | 45.56827 |
| 25.83354 | 29.92564 | 46.57671 |
| 30.12915 | 30.21814 | 51.23758 |
| 33.85417 | 30.49474 | 55.64522 |
| 37.3447 | 30.80507 | 60.59012 |
| -38.6732 | 31.4021 | 64.57462 |
| -35.3803 | 30.97635 | 57.86531 |
| -31.8643 | 30.73958 | 54.13409 |
| -27.7082 | 30.44161 | 49.43849 |
| -23.4967 | 30.26177 | 46.60458 |
| -18.7245 | 30.06326 | 43.47625 |
| -13.9251 | 29.86887 | 40.41287 |
| -8.81006 | 29.78665 | 39.11729 |
| -3.77241 | 29.70098 | 37.76725 |
| 1.552305 | 29.69563 | 37.68294 |
| 6.56314 | 29.66992 | 37.27769 |
| 11.75028 | 29.72556 | 38.15464 |
| 16.54923 | 29.85991 | 40.27177 |
| 21.49838 | 30.16728 | 45.11545 |
| 25.81819 | 30.28411 | 46.9565 |
| 30.09628 | 30.55701 | 51.25709 |
| 33.79384 | 30.77884 | 54.75282 |
| 37.25938 | 31.1058 | 59.90521 |
| -38.6739 | 31.73549 | 64.36265 |
| -35.3708 | 31.29424 | 57.48518 |
| -31.9374 | 31.10462 | 54.52966 |
| -27.7242 | 30.77346 | 49.36811 |
| -23.501 | 30.63064 | 47.14209 |
| -18.7728 | 30.43738 | 44.12979 |
| -14.0002 | 30.28459 | 41.74837 |
| -8.82253 | 30.06442 | 38.3167 |
| -3.808 | 29.98495 | 37.07797 |
| 1.525527 | 30.04357 | 37.99177 |
| 6.525368 | 29.98878 | 37.1378 |
| 11.73334 | 30.09013 | 38.71736 |
| 16.51423 | 30.19347 | 40.32807 |
| 21.45958 | 30.48522 | 44.87547 |
| 25.77086 | 30.60617 | 46.76064 |
| 30.11123 | 30.94277 | 52.00698 |
| 33.78863 | 31.15268 | 55.27883 |
| 37.30088 | 31.51441 | 60.91685 |
| -35.4863 | 31.72332 | 58.8275 |
| -31.9205 | 31.44973 | 54.60933 |
| -27.7641 | 31.12732 | 49.63855 |
| -23.5617 | 30.98467 | 47.4391 |
| -18.7681 | 30.72023 | 43.36193 |
| -14.0143 | 30.62379 | 41.87514 |
| -8.83686 | 30.35965 | 37.80258 |
| -3.81494 | 30.30609 | 36.97676 |
| 1.490784 | 30.3542 | 37.71865 |
| 6.494616 | 30.34389 | 37.55962 |
| 11.7261 | 30.47477 | 39.57751 |
| 16.49751 | 30.55928 | 40.88045 |
| 21.45121 | 30.84844 | 45.33873 |
| 25.7254 | 30.927 | 46.55001 |
| 30.08134 | 31.28332 | 52.0437 |
| 33.85244 | 31.58108 | 56.63457 |
| 37.33018 | 31.91218 | 61.73941 |
| -37.4562 | 31.46033 | 49.58511 |
| -35.4699 | 32.03282 | 58.31733 |
| -31.954 | 31.7781 | 54.43204 |
| -27.802 | 31.48228 | 49.91993 |
| -23.5789 | 31.28219 | 46.86793 |
| -18.752 | 30.98632 | 42.35494 |
| -14.0268 | 30.88881 | 40.86769 |
| -8.87519 | 30.73847 | 38.57447 |
| -3.82583 | 30.63397 | 36.98062 |
| 1.464903 | 30.66674 | 37.48039 |
| 6.464778 | 30.70033 | 37.99276 |
| 11.71591 | 30.85159 | 40.30002 |
| 16.49277 | 30.94705 | 41.75605 |
| 21.45628 | 31.234 | 46.13284 |
| 25.69469 | 31.27148 | 46.70452 |
| 30.06556 | 31.63954 | 52.3186 |
| 33.75404 | 31.86155 | 55.70494 |
| 37.24926 | 32.21703 | 61.127 |
| -38.62 | 32.83822 | 65.30173 |
| -35.5363 | 32.42086 | 59.00312 |
| -32.0214 | 32.14129 | 54.78385 |
| -27.8172 | 31.81421 | 49.84782 |
| -23.5822 | 31.64795 | 47.33863 |
| -18.7946 | 31.34912 | 42.82887 |
| -14.0473 | 31.24317 | 41.22985 |
| -8.91223 | 31.10434 | 39.13463 |
| -3.87012 | 31.00669 | 37.66094 |
| 1.436248 | 30.98948 | 37.40126 |
| 6.432416 | 31.04215 | 38.19619 |
| 11.68708 | 31.18079 | 40.2884 |
| 16.46617 | 31.29761 | 42.05149 |
| 21.38537 | 31.50995 | 45.25595 |
| 25.72121 | 31.68261 | 47.86177 |
| 30.01265 | 31.95537 | 51.97804 |
| 33.67491 | 32.16103 | 55.08179 |
| 37.24719 | 32.58721 | 61.5135 |
| -38.6166 | 33.20673 | 65.61552 |
| -35.556 | 32.77127 | 59.11251 |
| -31.9722 | 32.38615 | 53.36137 |
| -27.8421 | 32.15323 | 49.883 |
| -23.6374 | 31.99802 | 47.56517 |
| -18.8699 | 31.7723 | 44.19435 |
| -14.1192 | 31.64091 | 42.23224 |
| -8.93275 | 31.41685 | 38.88617 |
| -3.91251 | 31.37108 | 38.20268 |
| 1.412755 | 31.33668 | 37.68901 |
| 6.432779 | 31.39006 | 38.48622 |
| 11.66408 | 31.52966 | 40.5709 |
| 16.40518 | 31.58008 | 41.32382 |
| 21.36707 | 31.86365 | 45.55859 |
| 25.78114 | 32.09364 | 48.99312 |
| 29.9975 | 32.30813 | 52.19627 |
| 33.6292 | 32.49318 | 54.95974 |
| 37.24798 | 32.95914 | 61.91814 |
| -37.9754 | 33.03347 | 57.91616 |
| -35.58 | 33.12466 | 59.26391 |
| -31.9874 | 32.76427 | 53.93767 |
| -27.8752 | 32.50524 | 50.10958 |
| -23.6376 | 32.31647 | 47.31967 |
| -18.9136 | 32.13727 | 44.67132 |
| -14.1746 | 32.01133 | 42.81011 |
| -8.95915 | 31.74807 | 38.91942 |
| -3.95293 | 31.70485 | 38.28067 |
| 1.387527 | 31.72716 | 38.61037 |
| 6.402539 | 31.75579 | 39.03357 |
| 11.61535 | 31.80811 | 39.8068 |
| 16.39182 | 31.89568 | 41.10092 |
| 21.35531 | 32.22305 | 45.93908 |
| 25.70698 | 32.3841 | 48.31928 |
| 30.03584 | 32.72211 | 53.31456 |
| 33.60637 | 32.84934 | 55.195 |
| 37.17261 | 33.26152 | 61.28652 |
| -35.607 | 33.48988 | 59.58507 |
| -32.0779 | 33.15384 | 54.66969 |
| -27.8977 | 32.83978 | 50.07585 |
| -23.6297 | 32.62583 | 46.9464 |
| -18.9269 | 32.44739 | 44.33622 |
| -14.1846 | 32.34444 | 42.83043 |
| -9.00059 | 32.12184 | 39.57433 |
| -3.98677 | 32.01303 | 37.98283 |
| 1.362915 | 32.08648 | 39.05709 |
| 6.35867 | 32.064 | 38.72836 |
| 11.58737 | 32.1391 | 39.82683 |
| 16.36033 | 32.297 | 42.13652 |
| 21.33018 | 32.56341 | 46.03335 |
| 25.68954 | 32.7422 | 48.64857 |
| 29.99877 | 33.05653 | 53.24629 |
| 33.66704 | 33.28452 | 56.58114 |
| 37.1447 | 33.60947 | 61.33429 |
| -35.5985 | 33.83462 | 59.60314 |
| -32.1533 | 33.5291 | 55.17969 |
| -27.9784 | 33.24653 | 51.08839 |
| -23.6918 | 32.98591 | 47.31491 |
| -18.9058 | 32.69887 | 43.15892 |
| -14.194 | 32.68447 | 42.95049 |
| -9.01444 | 32.41125 | 38.99458 |
| -3.98734 | 32.29006 | 37.23996 |
| 1.337816 | 32.42852 | 39.24466 |
| 6.315497 | 32.37822 | 38.5164 |
| 11.5733 | 32.5083 | 40.39969 |
| 16.3647 | 32.63832 | 42.28224 |
| 21.33443 | 32.94707 | 46.75253 |
| 25.60972 | 33.02269 | 47.84746 |
| 29.91609 | 33.33486 | 52.36724 |
| 33.72142 | 33.68344 | 57.41434 |
| 37.11297 | 33.95476 | 61.34271 |
| -35.3632 | 33.98578 | 56.84633 |
| -32.2064 | 33.91555 | 55.83971 |
| -28.0089 | 33.59834 | 51.2931 |
| -23.7599 | 33.35828 | 47.85232 |
| -18.9784 | 33.11356 | 44.34466 |
| -14.2286 | 32.99698 | 42.6736 |
| -9.06841 | 32.84866 | 40.5477 |
| -4.03991 | 32.71634 | 38.65112 |
| 1.306274 | 32.69794 | 38.38749 |
| 6.293558 | 32.79677 | 39.80405 |
| 11.55809 | 32.87054 | 40.86136 |
| 16.32844 | 32.97359 | 42.33834 |
| 21.27669 | 33.23969 | 46.15244 |
| 25.58968 | 33.33587 | 47.53108 |
| 29.94907 | 33.74054 | 53.33127 |
| 33.66782 | 34.00588 | 57.13446 |
| 37.0708 | 34.28903 | 61.1929 |
| -32.1916 | 34.23272 | 55.50358 |
| -27.995 | 33.89312 | 50.68449 |
| -23.7716 | 33.74698 | 48.61067 |
| -19.0061 | 33.45668 | 44.49103 |
| -14.2248 | 33.29939 | 42.25906 |
| -9.08313 | 33.13986 | 39.99515 |
| -4.08164 | 33.0833 | 39.19266 |
| 1.281759 | 33.05922 | 38.85092 |
| 6.289363 | 33.11689 | 39.66921 |
| 11.53018 | 33.20949 | 40.98328 |
| 16.3175 | 33.28617 | 42.07138 |
| 21.21153 | 33.52271 | 45.42813 |
| 25.57806 | 33.74701 | 48.6111 |
| 29.93436 | 34.09543 | 53.55537 |
| 33.54311 | 34.25852 | 55.8697 |
| 37.02847 | 34.62294 | 61.04109 |
| -34.0153 | 33.52418 | 40.76198 |
| -32.2504 | 34.59088 | 55.74995 |
| -28.038 | 34.25722 | 51.06184 |
| -23.8343 | 34.10873 | 48.9753 |
| -19.0168 | 33.77009 | 44.2172 |
| -14.2682 | 33.63357 | 42.29899 |
| -9.09768 | 33.43122 | 39.45578 |
| -4.08621 | 33.37841 | 38.71384 |
| 1.253791 | 33.41547 | 39.23443 |
| 6.236147 | 33.3652 | 38.52823 |
| 11.49529 | 33.51961 | 40.69769 |
| 16.26216 | 33.65085 | 42.54185 |
| 21.15861 | 33.81735 | 44.88131 |
| 25.59705 | 34.15797 | 49.66727 |
| 29.88058 | 34.40952 | 53.20178 |
| 33.50455 | 34.60229 | 55.91033 |
| 36.98398 | 34.95131 | 60.81429 |
| -32.2961 | 34.96609 | 56.22878 |
| -28.0919 | 34.63772 | 51.65988 |
| -23.8536 | 34.41138 | 48.51063 |
| -19.0581 | 34.13689 | 44.6915 |
| -14.2611 | 33.9268 | 41.76831 |
| -9.10876 | 33.71232 | 38.78417 |
| -4.09037 | 33.68773 | 38.44205 |
| 1.227109 | 33.69283 | 38.51299 |
| 6.238118 | 33.72346 | 38.93923 |
| 11.47516 | 33.87853 | 41.09681 |
| 16.26483 | 33.99754 | 42.75267 |
| 21.1447 | 34.17857 | 45.27142 |
| 25.61243 | 34.52218 | 50.05239 |
| 29.92107 | 34.83332 | 54.38145 |
| 33.57965 | 35.02554 | 57.05596 |
| 37.00045 | 35.33792 | 61.40233 |
| -32.4016 | 35.37497 | 57.16227 |
| -28.0854 | 34.94277 | 51.20695 |
| -23.8438 | 34.76077 | 48.69907 |
| -19.1249 | 34.5465 | 45.74652 |
| -14.322 | 34.30802 | 42.46041 |
| -9.17084 | 34.16482 | 40.48732 |
| -4.13534 | 34.06609 | 39.12691 |
| 1.201608 | 34.07449 | 39.24257 |
| 6.202023 | 34.06414 | 39.10003 |
| 11.42792 | 34.16608 | 40.50461 |
| 16.21753 | 34.29964 | 42.34494 |
| 21.14964 | 34.56734 | 46.03378 |
| 25.48333 | 34.73596 | 48.35715 |
| 29.85238 | 35.12631 | 53.73594 |
| 33.57249 | 35.39933 | 57.49794 |
| 37.10523 | 35.82409 | 63.3508 |
| -32.4599 | 35.78953 | 58.15543 |
| -28.1452 | 35.33494 | 51.95149 |
| -23.8741 | 35.0673 | 48.29886 |
| -19.1491 | 34.87837 | 45.72039 |
| -14.3249 | 34.62467 | 42.25814 |
| -9.1908 | 34.48876 | 40.40327 |
| -4.17534 | 34.41113 | 39.34381 |
| 1.173952 | 34.39471 | 39.11975 |
| 6.175749 | 34.46167 | 40.03353 |
| 11.41256 | 34.53482 | 41.03193 |
| 16.18806 | 34.64992 | 42.6027 |
| 21.17669 | 34.99134 | 47.26222 |
| 25.49913 | 35.10488 | 48.81178 |
| 29.78864 | 35.42393 | 53.1659 |
| 33.50827 | 35.71534 | 57.14295 |
| 36.87432 | 35.99231 | 60.92283 |
| -32.5859 | 36.26183 | 59.9103 |
| -28.2211 | 35.75227 | 53.02211 |
| -23.8226 | 35.35444 | 47.64421 |
| -19.1505 | 35.17171 | 45.17402 |
| -14.3496 | 34.98604 | 42.66409 |
| -9.23285 | 34.88746 | 41.33154 |
| -4.22182 | 34.81733 | 40.38344 |
| 1.143601 | 34.71105 | 38.94671 |
| 6.138025 | 34.78908 | 40.00159 |
| 11.40489 | 34.92389 | 41.82391 |
| 16.19976 | 35.02447 | 43.18359 |
| 21.11931 | 35.27721 | 46.60015 |
| 25.55059 | 35.56728 | 50.5214 |
| 29.77306 | 35.77673 | 53.35276 |
| 33.49147 | 36.04536 | 56.98413 |
| 36.84429 | 36.37317 | 61.4155 |
| -32.5836 | 36.60692 | 59.92882 |
| -28.2681 | 36.14775 | 53.7799 |
| -23.8929 | 35.72363 | 48.10051 |
| -19.1695 | 35.4984 | 45.08445 |
| -14.4043 | 35.344 | 43.01675 |
| -9.26227 | 35.23278 | 41.52748 |
| -4.19161 | 35.11545 | 39.95624 |
| 1.122839 | 35.09245 | 39.64829 |
| 6.109905 | 35.15469 | 40.48173 |
| 11.38836 | 35.30009 | 42.42874 |
| 16.15628 | 35.33918 | 42.95222 |
| 21.09685 | 35.62795 | 46.81923 |
| 25.47224 | 35.85231 | 49.82363 |
| 29.80354 | 36.18941 | 54.33786 |
| 33.40619 | 36.33494 | 56.28664 |
| 36.79241 | 36.74682 | 61.80219 |
| -32.239 | 36.62892 | 55.66055 |
| -28.3106 | 36.54709 | 54.57488 |
| -23.9654 | 36.14101 | 49.18764 |
| -19.1852 | 35.81938 | 44.92067 |
| -14.4553 | 35.7018 | 43.36077 |
| -9.27842 | 35.53428 | 41.13843 |
| -4.22815 | 35.42812 | 39.73003 |
| 1.095931 | 35.42247 | 39.65508 |
| 6.075497 | 35.51064 | 40.82476 |
| 11.3391 | 35.57019 | 41.61476 |
| 16.12092 | 35.66584 | 42.88378 |
| 21.08238 | 35.98895 | 47.17035 |
| 25.52523 | 36.27056 | 50.90628 |
| 29.69933 | 36.43517 | 53.09012 |
| 33.38018 | 36.6858 | 56.41515 |
| 36.27768 | 36.66135 | 56.09079 |
| -28.3035 | 36.90162 | 54.76631 |
| -23.9518 | 36.43211 | 48.59493 |
| -19.2648 | 36.2569 | 46.29191 |
| -14.4416 | 35.97915 | 42.641 |
| -9.29218 | 35.81605 | 40.49723 |
| -4.27087 | 35.77698 | 39.98368 |
| 1.069819 | 35.75856 | 39.74155 |
| 6.095261 | 35.80614 | 40.36689 |
| 11.29455 | 35.85646 | 41.02839 |
| 16.08614 | 36.0014 | 42.93352 |
| 21.05476 | 36.32368 | 47.16967 |
| 25.44457 | 36.55557 | 50.21767 |
| 29.67225 | 36.77937 | 53.15938 |
| 33.50074 | 37.19953 | 58.68211 |
| 35.19165 | 35.992 | 42.80992 |
| -28.1708 | 37.1061 | 52.99979 |
| -23.9495 | 36.72451 | 48.02992 |
| -19.2445 | 36.50845 | 45.21589 |
| -14.4797 | 36.38629 | 43.6248 |
| -9.32163 | 36.15006 | 40.54808 |
| -4.3491 | 36.19437 | 41.12527 |
| 1.042712 | 36.08015 | 39.63756 |
| 6.043668 | 36.04841 | 39.22415 |
| 11.28089 | 36.23524 | 41.65747 |
| 16.06856 | 36.37868 | 43.52576 |
| 21.0145 | 36.63961 | 46.9242 |
| 25.43606 | 36.88935 | 50.17681 |
| 29.69946 | 37.19168 | 54.11442 |
| 33.4518 | 37.52127 | 58.40707 |
| -28.4458 | 37.86495 | 58.42026 |
| -23.9543 | 37.06304 | 48.07048 |
| -19.2745 | 36.8505 | 45.32732 |
| -14.5304 | 36.74263 | 43.93517 |
| -9.35685 | 36.52088 | 41.07311 |
| -4.35775 | 36.55974 | 41.5747 |
| 1.007729 | 36.38612 | 39.3339 |
| 6.015555 | 36.44976 | 40.15531 |
| 11.25909 | 36.5833 | 41.87878 |
| 16.03299 | 36.71449 | 43.57199 |
| 21.01308 | 37.02159 | 47.53552 |
| 25.38375 | 37.21116 | 49.98221 |
| 29.64927 | 37.51003 | 53.83958 |
| 33.454 | 37.93535 | 59.32883 |
| -23.868 | 37.30629 | 46.89166 |
| -19.3428 | 37.26445 | 46.3565 |
| -14.5973 | 37.13907 | 44.75279 |
| -9.38704 | 36.87834 | 41.41795 |
| -4.36602 | 36.89079 | 41.57713 |
| 0.981423 | 36.74119 | 39.66365 |
| 5.988 | 36.84674 | 41.01379 |
| 11.23762 | 36.93957 | 42.20107 |
| 16.01458 | 37.08446 | 44.05429 |
| 21.0087 | 37.39846 | 48.07055 |
| 25.32969 | 37.5324 | 49.78379 |
| 29.56781 | 37.79146 | 53.0973 |
| 33.57039 | 38.4991 | 62.14854 |
| -23.8457 | 37.62218 | 46.65474 |
| -19.3508 | 37.56423 | 45.92005 |
| -14.622 | 37.42765 | 44.18861 |
| -9.40825 | 37.19284 | 41.21194 |
| -4.36226 | 37.11599 | 40.23775 |
| 0.950114 | 37.09982 | 40.03276 |
| 5.953501 | 37.19559 | 41.24683 |
| 11.22564 | 37.31983 | 42.82186 |
| 16.02072 | 37.4382 | 44.3224 |
| 20.98149 | 37.73492 | 48.0839 |
| 25.36175 | 37.88466 | 49.98216 |
| 29.59936 | 38.22519 | 54.29908 |
| 33.61074 | 38.98754 | 63.96328 |
| -23.8457 | 37.97959 | 46.94362 |
| -19.3859 | 37.91823 | 46.17262 |
| -14.6668 | 37.85485 | 45.37616 |
| -9.4501 | 37.58859 | 42.0305 |
| -4.4142 | 37.50513 | 40.98188 |
| 0.92192 | 37.41007 | 39.78741 |
| 5.912897 | 37.51187 | 41.06656 |
| 11.2023 | 37.66405 | 42.97871 |
| 16.00849 | 37.8388 | 45.17448 |
| 20.97665 | 38.10733 | 48.5487 |
| 25.33014 | 38.24647 | 50.29696 |
| 29.59577 | 38.63837 | 55.22134 |
| 33.71541 | 39.62106 | 67.56905 |
| -19.3947 | 38.21657 | 45.72738 |
| -14.6675 | 38.16477 | 45.08216 |
| -9.47736 | 37.92548 | 42.10167 |
| -4.44737 | 37.77275 | 40.19935 |
| 0.894158 | 37.74825 | 39.89417 |
| 5.90993 | 37.84282 | 41.07211 |
| 11.15629 | 37.94206 | 42.30827 |
| 15.97565 | 38.1142 | 44.45233 |
| 20.94811 | 38.42591 | 48.33488 |
| 25.31604 | 38.58207 | 50.27995 |
| 29.61889 | 39.10404 | 56.78133 |
| -19.4177 | 38.54864 | 45.70645 |
| -14.6715 | 38.48045 | 44.8644 |
| -9.52 | 38.31273 | 42.79348 |
| -4.45434 | 38.10018 | 40.16897 |
| 0.865992 | 38.12954 | 40.53145 |
| 5.877239 | 38.20036 | 41.40598 |
| 11.10643 | 38.21066 | 41.53315 |
| 15.90803 | 38.39641 | 43.82672 |
| 20.92696 | 38.75911 | 48.30529 |
| 25.31534 | 38.93458 | 50.47188 |
| 29.81743 | 39.82596 | 61.47841 |
| -19.4575 | 38.92092 | 46.17804 |
| -14.6843 | 38.81497 | 44.88107 |
| -9.55595 | 38.70056 | 43.4804 |
| -4.46852 | 38.4808 | 40.79021 |
| 0.84068 | 38.49301 | 40.93974 |
| 5.870891 | 38.52264 | 41.3024 |
| 11.06992 | 38.51604 | 41.2216 |
| 15.88588 | 38.78195 | 44.4768 |
| 20.91292 | 39.10458 | 48.42629 |
| 25.31899 | 39.32845 | 51.16695 |
| -19.5233 | 39.3678 | 47.54709 |
| -14.7654 | 39.23853 | 45.97807 |
| -9.57239 | 39.00915 | 43.19396 |
| -4.50624 | 38.83939 | 41.13354 |
| 0.814417 | 38.83271 | 41.05237 |
| 5.820954 | 38.77942 | 40.40556 |
| 11.0982 | 39.03583 | 43.51779 |
| 15.86899 | 39.0785 | 44.03564 |
| 20.93177 | 39.51397 | 49.32121 |
| 25.30171 | 39.68344 | 51.37812 |
| -19.5597 | 39.75862 | 48.21839 |
| -14.787 | 39.58647 | 46.14651 |
| -9.57472 | 39.26 | 42.21735 |
| -4.56021 | 39.29565 | 42.64644 |
| 0.783786 | 39.13903 | 40.76146 |
| 5.781624 | 39.11504 | 40.47279 |
| 11.09068 | 39.44239 | 44.41252 |
| 15.89368 | 39.48755 | 44.95598 |
| 20.89635 | 39.83192 | 49.10054 |
| 25.23387 | 40.03678 | 51.56607 |
| -19.6311 | 40.23296 | 49.87515 |
| -14.795 | 39.86002 | 45.42434 |
| -9.57632 | 39.50054 | 41.13414 |
| -4.59108 | 39.5395 | 41.59906 |
| 0.75605 | 39.47179 | 40.791 |
| 5.758052 | 39.52714 | 41.45161 |
| 11.03363 | 39.6762 | 43.23054 |
| 15.86569 | 39.91606 | 46.09308 |
| 20.83822 | 40.10961 | 48.40295 |
| 25.25288 | 40.47993 | 52.82255 |
| -14.9082 | 40.59562 | 50.18263 |
| -9.62686 | 39.97198 | 42.8016 |
| -4.60238 | 39.9019 | 41.97225 |
| 0.72754 | 39.80451 | 40.81959 |
| 5.758495 | 39.88257 | 41.74339 |
| 11.00644 | 40.01181 | 43.27305 |
| 15.83041 | 40.24592 | 46.04381 |
| 20.9004 | 40.63637 | 50.66495 |
| 25.27557 | 40.90954 | 53.89796 |
| -9.62906 | 40.30105 | 42.77056 |
| -4.61159 | 40.1921 | 41.49172 |
| 0.702015 | 40.13323 | 40.80066 |
| 5.719202 | 40.19426 | 41.5171 |
| 10.9835 | 40.38761 | 43.78663 |
| 20.83269 | 40.94981 | 50.38566 |
| 24.80102 | 40.42331 | 44.20564 |
| -9.64175 | 40.7573 | 44.22061 |
| -4.66353 | 40.54895 | 41.79499 |
| 0.675951 | 40.52049 | 41.4637 |
| 5.702821 | 40.46689 | 40.83966 |
| 10.9495 | 40.73509 | 43.96202 |
| 20.94769 | 41.61569 | 54.21413 |
| -4.64975 | 40.85145 | 41.46577 |
| 0.644983 | 40.80811 | 40.96524 |
| 5.663646 | 40.78764 | 40.72888 |
| 10.88973 | 41.03674 | 43.60544 |
| -4.661 | 41.03474 | 39.77629 |
| 0.609354 | 41.14064 | 40.98934 |
| 5.61957 | 41.26044 | 42.36167 |
| 10.7442 | 41.02934 | 39.71435 |
| 0.583451 | 41.44941 | 40.74298 |
| 5.576151 | 41.49547 | 41.26637 |
| 0.561708 | 42.06196 | 43.92532 |
| 5.609757 | 42.12343 | 44.6184 |
| -57.8228 | 11.22136 | 134.1583 |
| -57.1119 | 11.31389 | 134.9567 |
| -56.7779 | 11.38584 | 135.5777 |
| -56.7516 | 11.44755 | 139.2955 |
| -43.3217 | -31.5455 | 116.5598 |
| -43.1714 | -31.8848 | 119.019 |
| -34.8806 | -19.1988 | 29.44869 |
| -42.543 | -31.9206 | 116.1088 |
| -34.5022 | -18.9607 | 28.65086 |
| -42.9994 | -32.6743 | 125.5198 |
| -34.3318 | -18.85 | 30.2287 |
| -42.673 | -32.9053 | 126.0857 |
| -41.8102 | -32.6999 | 120.4472 |
| -41.85 | -33.1452 | 125.2594 |
| -36.9128 | 33.87789 | 71.53699 |
| -41.5424 | -33.3545 | 126.059 |
| -41.0399 | -33.4256 | 124.5577 |
| -40.3919 | -33.3722 | 121.2893 |
| -39.6993 | -33.3425 | 117.4256 |
| -39.4914 | -33.6157 | 119.4001 |
| -32.7808 | -17.083 | 41.53015 |
| -38.8841 | -33.5776 | 116.5022 |
| -32.6604 | -17.0534 | 43.96444 |
| -38.621 | -33.8374 | 117.8196 |
| -32.238 | -16.8434 | 42.66544 |
| -38.2004 | -33.9739 | 117.191 |
| -31.8066 | -16.638 | 41.23001 |
| -38.0306 | -34.2629 | 119.713 |
| -31.6191 | -16.5529 | 42.87324 |
| -37.3462 | -34.1721 | 115.7336 |
| -37.187 | -34.5074 | 118.4255 |
| -36.6839 | -34.5314 | 116.7107 |
| -36.6428 | -34.9364 | 121.004 |
| -30.3158 | -16.554 | 42.44702 |
| -36.358 | -35.1142 | 122.1603 |
| -31.0906 | -18.016 | 56.99053 |
| -36.2122 | -35.486 | 125.1912 |
| -28.5382 | -15.6748 | 27.08667 |
| -30.8669 | -17.93 | 58.38849 |
| -35.4116 | -35.215 | 119.477 |
| -35.1744 | -35.4385 | 121.2986 |
| -31.346 | 37.39967 | 73.95577 |
| -27.9224 | -15.3978 | 27.05352 |
| -30.1867 | -17.6402 | 58.07358 |
| -34.725 | -35.5101 | 120.2476 |
| -31.2546 | 37.73721 | 77.38877 |
| -27.5144 | -15.2007 | 25.65068 |
| -34.2229 | -35.5156 | 118.4488 |
| -30.5656 | 37.51379 | 72.54086 |
| -28.4533 | -15.6842 | 43.03533 |
| -29.5346 | -17.3716 | 58.13885 |
| -33.8967 | -35.6466 | 119.0709 |
| -28.1169 | -15.5725 | 42.7741 |
| -29.1901 | -17.2516 | 57.91305 |
| -33.7185 | -35.9266 | 121.7923 |
| -27.615 | -15.3883 | 40.14953 |
| -28.9968 | -17.2121 | 59.83635 |
| -33.3167 | -35.9803 | 121.3835 |
| -28.7305 | -17.131 | 60.74781 |
| -33.0938 | -36.1971 | 123.5399 |
| -28.3539 | -16.9894 | 60.07384 |
| -32.8254 | -36.4245 | 125.0794 |
| -27.9954 | -16.8468 | 59.65216 |
| -32.4816 | -36.4833 | 125.5435 |
| -27.5524 | -16.6902 | 57.96891 |
| -32.0392 | -36.5501 | 124.555 |
| -27.4052 | -16.6832 | 60.68512 |
| -31.8398 | -36.7951 | 127.188 |
| -31.3115 | -36.7275 | 124.8932 |
| -31.0015 | -36.904 | 125.892 |
| -30.5283 | -36.9023 | 124.3872 |
| -30.2375 | -37.0379 | 125.7031 |
| -25.4114 | -15.9259 | 55.26211 |
| -29.9441 | -37.2079 | 127.0079 |
| -25.1349 | -15.8811 | 56.06064 |
| -29.4632 | -37.1627 | 125.3409 |
| -29.2587 | -37.4761 | 128.1103 |
| -28.9525 | -37.5984 | 129.2773 |
| -28.5028 | -37.5713 | 128.0954 |
| -22.8191 | -13.7038 | 39.17039 |
| -28.1465 | -37.6358 | 128.45 |
| -22.4394 | -13.566 | 38.0765 |
| -27.8904 | -37.8616 | 130.5127 |
| -25.6944 | 42.20348 | 99.33408 |
| -22.1401 | -13.4727 | 38.33569 |
| -27.4456 | -37.875 | 129.3877 |
| -21.7959 | -13.4113 | 37.8205 |
| -23.1491 | -15.4955 | 61.32642 |
| -27.0888 | -37.9261 | 129.7629 |
| -21.4484 | -13.2853 | 37.23398 |
| -26.7133 | -37.9546 | 129.819 |
| -21.2058 | -13.246 | 38.50148 |
| -26.4313 | -38.2326 | 131.5407 |
| -20.9302 | -13.1922 | 39.20622 |
| -25.9624 | -38.1068 | 129.9362 |
| -20.5061 | -13.0625 | 37.21805 |
| -25.6122 | -38.2018 | 130.4589 |
| -23.5686 | 42.82799 | 99.66937 |
| -20.1209 | -12.9625 | 35.89651 |
| -25.2221 | -38.2807 | 130.256 |
| -19.7009 | -12.8301 | 33.88811 |
| -20.8134 | -14.7237 | 54.73711 |
| -24.8565 | -38.2976 | 130.5074 |
| -19.173 | -12.6961 | 29.7773 |
| -24.4989 | -38.4281 | 130.9164 |
| -18.7046 | -11.9642 | 26.70115 |
| -24.2479 | -38.6367 | 133.391 |
| -18.4067 | -11.936 | 26.86653 |
| -23.8689 | -38.6649 | 133.4338 |
| -18.1246 | -11.884 | 27.35117 |
| -23.3539 | -38.5181 | 130.787 |
| -17.8261 | -11.8157 | 27.51838 |
| -23.0431 | -38.6432 | 132.1636 |
| -17.5124 | -11.7383 | 27.38065 |
| -22.6361 | -38.674 | 131.6213 |
| -22.3399 | -38.8294 | 133.3509 |
| -21.9814 | -38.9043 | 133.8248 |
| -16.5714 | -11.5594 | 26.94946 |
| -21.768 | -39.2278 | 137.3988 |
| -21.2914 | -39.1197 | 135.4005 |
| -20.8042 | -38.9784 | 133.1099 |
| -15.613 | -11.3863 | 26.08857 |
| -20.3825 | -38.9448 | 132.2048 |
| -15.3208 | -11.3449 | 26.39906 |
| -20.0272 | -39.0251 | 132.7717 |
| -19.7088 | -39.1773 | 134.2034 |
| -19.3417 | -39.2052 | 134.5469 |
| -18.9031 | -39.1902 | 133.2047 |
| -18.4758 | -39.0857 | 132.0886 |
| -18.1989 | -39.3142 | 134.6263 |
| -17.8618 | -39.4363 | 135.7508 |
| -14.6565 | 41.28528 | 64.00105 |
| -17.4501 | -39.3979 | 135.0187 |
| -14.2312 | 41.08062 | 61.59826 |
| -13.0807 | -11.4281 | 31.82064 |
| -17.1642 | -39.6564 | 137.5138 |
| -13.7277 | 40.74183 | 57.0486 |
| -12.7398 | -11.3381 | 31.00534 |
| -16.7101 | -39.5417 | 135.6684 |
| -13.6333 | 41.3712 | 63.31459 |
| -12.4016 | -11.2512 | 30.23277 |
| -16.3686 | -39.6396 | 136.7795 |
| -13.4177 | 41.71454 | 66.50171 |
| -15.9867 | -39.6617 | 136.8252 |
| -13.125 | 41.83559 | 67.66522 |
| -15.6035 | -39.6849 | 136.837 |
| -11.3276 | -10.9708 | 26.03754 |
| -15.2537 | -39.7333 | 137.8003 |
| -11.1796 | -11.0736 | 30.62737 |
| -14.8461 | -39.7063 | 137.123 |
| -14.5233 | -39.8829 | 138.9342 |
| -14.0687 | -39.7915 | 136.8293 |
| -13.672 | -39.7773 | 136.4229 |
| -13.3006 | -39.7825 | 136.8022 |
| -12.7664 | -39.4811 | 131.9308 |
| -9.32549 | -10.3844 | 30.65379 |
| -12.5107 | -39.7577 | 136.0513 |
| -8.93959 | -10.2064 | 28.05478 |
| -12.1485 | -39.8082 | 136.7575 |
| -9.3039 | 40.92729 | 51.36124 |
| -8.68491 | -10.2135 | 29.88236 |
| -11.7806 | -39.8437 | 137.3037 |
| -8.39015 | -10.2478 | 30.37606 |
| -11.3998 | -39.8803 | 137.4132 |
| -8.03949 | -10.1139 | 28.85586 |
| -11.0474 | -39.999 | 138.5663 |
| -8.43808 | 40.34386 | 55.30155 |
| -7.73325 | -10.1274 | 28.91864 |
| -10.6831 | -40.0378 | 139.336 |
| -8.13976 | 40.46935 | 56.38329 |
| -10.3053 | -40.0574 | 139.6287 |
| -7.09529 | -10.024 | 28.04769 |
| -9.87665 | -39.9649 | 137.9273 |
| -6.78061 | -9.97848 | 27.74983 |
| -9.5416 | -40.1056 | 139.9323 |
| -9.17261 | -40.159 | 140.6354 |
| -8.75692 | -40.0623 | 139.3688 |
| -8.32798 | -39.9559 | 137.4349 |
| -7.91337 | -39.8814 | 136.0332 |
| -7.57392 | -40.031 | 138.1 |
| -7.20123 | -40.0469 | 138.6796 |
| -6.83561 | -40.1533 | 139.6588 |
| -4.91688 | 42.10793 | 58.97729 |
| -4.35878 | -10.0828 | 29.60679 |
| -6.42409 | -39.9815 | 138.2953 |
| -4.61843 | 42.25572 | 60.64843 |
| -4.04248 | -10.044 | 29.17774 |
| -6.00974 | -39.8969 | 136.6729 |
| -3.73874 | -10.0166 | 29.42772 |
| -5.64643 | -39.9535 | 137.8208 |
| -3.9407 | 42.20816 | 59.62414 |
| -3.42346 | -9.97287 | 29.01534 |
| -5.24904 | -39.9082 | 137.0471 |
| -3.61128 | 42.22796 | 59.62864 |
| -3.10037 | -9.98725 | 28.08552 |
| -4.86519 | -39.9186 | 137.0427 |
| -3.29008 | 42.28937 | 60.16377 |
| -2.79982 | -9.95499 | 28.52582 |
| -4.50296 | -39.9934 | 138.4332 |
| -2.49729 | -9.92614 | 28.87437 |
| -4.12549 | -40.0126 | 138.9253 |
| -2.18932 | -9.84438 | 28.8718 |
| -3.72891 | -39.932 | 138.1101 |
| -1.88001 | -9.85984 | 28.76851 |
| -3.3581 | -39.9781 | 139.1341 |
| -1.5739 | -9.82987 | 28.90545 |
| -3.00214 | -40.1502 | 141.4412 |
| -1.25914 | -9.82421 | 28.33795 |
| -2.60264 | -40.0501 | 140.3865 |
| -0.93835 | -9.72062 | 27.1649 |
| -2.23082 | -40.1211 | 141.6537 |
| -0.82237 | -11.4655 | 45.18097 |
| -1.854 | -40.1972 | 142.6155 |
| -0.50894 | -11.4608 | 45.78127 |
| -1.45374 | -40.077 | 141.3437 |
| -0.17897 | -11.4099 | 44.67177 |
| -1.05652 | -40.0244 | 140.2081 |
| 0.14978 | -11.3594 | 43.52327 |
| -0.64959 | -39.8127 | 137.7408 |
| 0.618271 | -9.13363 | 24.21357 |
| 0.468719 | -11.3339 | 43.42837 |
| -0.2592 | -39.763 | 136.9534 |
| 0.782044 | -11.3272 | 44.10735 |
| 0.10817 | -39.9091 | 139.2608 |
| 1.096144 | -11.3169 | 44.81403 |
| 0.482759 | -40.0474 | 140.9442 |
| 1.531526 | -9.08203 | 24.34134 |
| 1.412413 | -11.2984 | 45.31344 |
| 0.868853 | -39.9931 | 141.0174 |
| 1.737623 | -11.2471 | 44.15737 |
| 1.251861 | -40.0602 | 141.7312 |
| 2.055718 | -11.2308 | 44.28585 |
| 1.637928 | -40.0892 | 141.9636 |
| 2.371822 | -11.2327 | 45.02152 |
| 2.032438 | -39.8924 | 139.9264 |
| 2.750635 | -9.00907 | 24.30307 |
| 2.690271 | -11.2255 | 45.30048 |
| 2.421125 | -39.7986 | 138.921 |
| 3.008126 | -11.2255 | 46.03235 |
| 2.806085 | -39.7834 | 138.9818 |
| 3.326957 | -11.2309 | 46.79873 |
| 3.18907 | -39.8243 | 140.4411 |
| 3.648578 | -11.1768 | 45.34159 |
| 3.573796 | -39.9777 | 142.2969 |
| 3.968165 | -11.1242 | 43.73478 |
| 3.961354 | -39.863 | 140.8709 |
| 4.287061 | -11.1343 | 44.60212 |
| 4.348943 | -39.9736 | 142.6625 |
| 4.584614 | -9.39721 | 28.53654 |
| 4.607272 | -11.1469 | 45.54393 |
| 4.735957 | -39.9129 | 142.1358 |
| 4.895863 | -9.42814 | 30.28605 |
| 4.926313 | -11.133 | 45.26095 |
| 5.122684 | -39.838 | 141.8316 |
| 5.20508 | -9.44857 | 30.39519 |
| 5.246386 | -11.1309 | 45.50022 |
| 5.511427 | -39.8628 | 142.4206 |
| 5.512444 | -9.3416 | 29.92071 |
| 5.564873 | -11.1131 | 45.18028 |
| 5.894954 | -39.7373 | 141.2512 |
| 5.823491 | -9.36327 | 30.49728 |
| 5.885734 | -11.1096 | 45.54254 |
| 6.27093 | -39.5374 | 138.6528 |
| 6.132756 | -9.41605 | 30.53832 |
| 6.204592 | -11.0917 | 45.38602 |
| 6.65528 | -39.5209 | 138.5385 |
| 6.438882 | -9.31111 | 30.00232 |
| 6.526776 | -11.0896 | 45.8694 |
| 7.053912 | -39.6519 | 141.0312 |
| 6.7492 | -9.32396 | 30.25837 |
| 6.848594 | -11.0733 | 46.1855 |
| 7.427457 | -39.4603 | 138.944 |
| 7.057789 | -9.37098 | 30.2138 |
| 7.161956 | -11.0327 | 45.21977 |
| 7.821086 | -39.4984 | 140.1716 |
| 7.369257 | -9.38902 | 30.55402 |
| 7.477368 | -11.003 | 44.69929 |
| 8.216849 | -39.5683 | 141.4531 |
| 7.687603 | -9.31323 | 31.66145 |
| 7.796974 | -10.9931 | 44.76897 |
| 8.601915 | -39.5476 | 141.2372 |
| 8.005127 | -9.38415 | 32.50616 |
| 8.118347 | -10.9954 | 45.02316 |
| 8.993903 | -39.5015 | 141.8201 |
| 8.314467 | -9.38807 | 32.38976 |
| 8.435877 | -10.9922 | 44.84652 |
| 9.365483 | -39.3748 | 140.2253 |
| 8.630912 | -9.44657 | 32.96901 |
| 8.761135 | -11.0057 | 45.43327 |
| 9.738663 | -39.2513 | 138.9976 |
| 8.889265 | -9.28078 | 28.26479 |
| 9.093239 | -11.0399 | 46.56027 |
| 10.11518 | -39.1898 | 138.2235 |
| 9.417789 | -11.0578 | 46.91646 |
| 10.53329 | -39.3581 | 141.0514 |
| 9.737269 | -11.0617 | 46.82888 |
| 10.90196 | -39.1928 | 139.6242 |
| 9.768733 | -8.82453 | 25.01747 |
| 10.06913 | -11.0892 | 47.68471 |
| 11.28568 | -39.1153 | 139.4835 |
| 10.07469 | -8.8266 | 25.06974 |
| 10.39611 | -11.1 | 48.10525 |
| 11.70443 | -39.2809 | 141.8672 |
| 10.37754 | -8.81459 | 24.9058 |
| 10.71015 | -11.0905 | 47.60209 |
| 12.06966 | -39.1015 | 140.3712 |
| 11.02161 | -11.0815 | 46.97662 |
| 12.44191 | -39.0075 | 139.4684 |
| 11.36307 | -11.1207 | 48.27472 |
| 12.89368 | -39.271 | 143.5971 |
| 11.29023 | -8.79418 | 24.74185 |
| 11.68983 | -11.1364 | 48.58626 |
| 13.25362 | -39.0847 | 141.9006 |
| 11.596 | -8.79785 | 24.78374 |
| 11.96039 | -11.0755 | 45.65439 |
| 13.56107 | -38.6873 | 137.334 |
| 12.33099 | -11.1486 | 48.4576 |
| 13.97133 | -38.757 | 138.781 |
| 12.21307 | -8.80411 | 25.15234 |
| 12.66932 | -11.1742 | 49.34144 |
| 14.35764 | -38.7048 | 138.8512 |
| 12.95614 | -11.1342 | 47.52875 |
| 14.74891 | -38.664 | 139.1696 |
| 13.20423 | -11.0524 | 43.93027 |
| 15.16199 | -38.7357 | 140.5417 |
| 13.12645 | -8.81106 | 25.02964 |
| 13.48099 | -11.0149 | 41.94039 |
| 15.49181 | -38.5148 | 137.8513 |
| 13.43438 | -8.82179 | 25.1538 |
| 13.81131 | -11.0393 | 42.55096 |
| 15.92099 | -38.5692 | 139.9209 |
| 14.12638 | -11.0467 | 42.44124 |
| 16.31674 | -38.5692 | 140.3659 |
| 14.08868 | -9.35728 | 27.04654 |
| 14.44482 | -11.0514 | 42.48448 |
| 16.65579 | -38.328 | 138.3265 |
| 15.57355 | 43.61507 | 76.61392 |
| 14.49407 | -9.36603 | 31.20066 |
| 14.86156 | -11.163 | 46.66084 |
| 17.11105 | -38.5509 | 141.2959 |
| 16.04263 | 44.02653 | 81.83152 |
| 14.81742 | -9.39328 | 31.76412 |
| 15.18378 | -11.1753 | 46.73523 |
| 17.47504 | -38.4171 | 140.3656 |
| 16.34758 | 43.8266 | 80.23777 |
| 15.14399 | -9.45951 | 32.42437 |
| 15.47306 | -11.1594 | 45.49692 |
| 17.78021 | -38.1643 | 137.1503 |
| 16.69969 | 43.8317 | 80.55345 |
| 15.45577 | -9.46127 | 32.47678 |
| 15.73035 | -11.1102 | 43.08931 |
| 18.13917 | -38.0341 | 136.1884 |
| 17.0258 | 43.70954 | 79.87403 |
| 15.76033 | -9.39993 | 32.25497 |
| 16.13773 | -11.2016 | 46.4565 |
| 18.53749 | -37.9547 | 136.7583 |
| 17.35 | 43.61937 | 79.15951 |
| 16.09105 | -9.41082 | 33.00381 |
| 16.45332 | -11.2046 | 46.28539 |
| 18.94681 | -37.9926 | 137.7021 |
| 16.40078 | -9.43497 | 32.96491 |
| 16.7418 | -11.1876 | 45.15389 |
| 19.37367 | -38.0391 | 139.2257 |
| 16.69071 | -9.40682 | 32.23713 |
| 17.05844 | -11.1954 | 45.05952 |
| 19.68123 | -37.7998 | 136.5149 |
| 16.99738 | -9.42155 | 32.11391 |
| 20.09389 | -37.7769 | 137.5104 |
| 18.75276 | 43.5616 | 80.15712 |
| 17.30017 | -9.43506 | 31.86783 |
| 20.50746 | -37.8233 | 138.4902 |
| 19.06961 | 43.47966 | 79.27885 |
| 17.59934 | -9.49015 | 31.51511 |
| 20.85132 | -37.6329 | 137.1604 |
| 19.35136 | 43.26456 | 77.32477 |
| 17.91826 | -9.46154 | 31.80536 |
| 21.22486 | -37.5699 | 136.8328 |
| 19.71683 | 43.30861 | 78.05704 |
| 18.21996 | -9.49098 | 31.54802 |
| 21.57562 | -37.48 | 135.8114 |
| 18.39992 | -9.45981 | 27.59975 |
| 21.99464 | -37.4241 | 136.9095 |
| 22.38385 | -37.4141 | 137.0741 |
| 22.65328 | -37.13 | 133.7388 |
| 19.25054 | -8.96989 | 25.58502 |
| 23.19993 | -37.3555 | 138.4601 |
| 23.57795 | -37.3134 | 138.2708 |
| 19.87706 | -9.02876 | 26.00493 |
| 23.90594 | -37.1296 | 136.7143 |
| 20.21169 | -9.06632 | 26.77899 |
| 24.29281 | -37.0877 | 136.804 |
| 24.50749 | -36.7001 | 132.3344 |
| 20.86227 | -9.13524 | 27.75742 |
| 24.85609 | -36.5723 | 131.5062 |
| 21.16114 | -9.15396 | 27.54625 |
| 25.33014 | -36.6504 | 133.9085 |
| 21.43583 | -9.16012 | 26.73653 |
| 25.77904 | -36.6838 | 135.596 |
| 26.11415 | -36.5584 | 134.4223 |
| 22.09724 | -9.23 | 27.91212 |
| 26.55003 | -36.5528 | 135.7283 |
| 26.89321 | -36.3676 | 134.7838 |
| 27.28333 | -36.3319 | 134.9694 |
| 27.55614 | -36.0917 | 132.4464 |
| 27.99134 | -36.0603 | 133.6854 |
| 24.05219 | -10.0365 | 37.25654 |
| 28.39319 | -36.03 | 134.1414 |
| 24.38724 | -10.0879 | 37.72327 |
| 28.80593 | -36.0262 | 134.8227 |
| 24.8239 | -10.1655 | 40.37501 |
| 29.162 | -35.9099 | 134.2568 |
| 25.08051 | -10.2124 | 39.10899 |
| 29.52875 | -35.8269 | 133.9355 |
| 25.31077 | -10.216 | 37.32692 |
| 29.88167 | -35.6397 | 133.3333 |
| 25.71188 | -10.3055 | 39.13313 |
| 30.32254 | -35.6694 | 134.5689 |
| 26.11299 | -10.3681 | 40.88748 |
| 30.52368 | -35.3501 | 130.878 |
| 26.43314 | -10.4246 | 40.96388 |
| 30.91528 | -35.2681 | 131.1224 |
| 26.76847 | -10.465 | 41.33918 |
| 31.26957 | -35.1447 | 130.6199 |
| 27.21236 | -10.5426 | 43.82801 |
| 31.65281 | -35.0153 | 130.6976 |
| 32.03215 | -34.9523 | 130.6979 |
| 30.59929 | 41.14012 | 96.19228 |
| 32.28972 | -34.6626 | 128.3795 |
| 29.8803 | 39.34613 | 76.00966 |
| 32.72284 | -34.6197 | 129.4209 |
| 30.58477 | 39.77504 | 82.75088 |
| 27.63884 | -9.8849 | 28.1168 |
| 32.93696 | -34.314 | 126.3737 |
| 30.95746 | 39.70395 | 83.24476 |
| 27.99413 | -9.95855 | 28.99354 |
| 33.41996 | -34.3486 | 128.3271 |
| 31.29537 | 39.59917 | 83.09768 |
| 28.27873 | -9.99463 | 28.57032 |
| 33.95025 | -34.4341 | 131.086 |
| 29.16837 | -10.2833 | 38.95728 |
| 30.14869 | -12.8882 | 56.45521 |
| 34.22676 | -34.2305 | 129.2459 |
| 32.15932 | 39.65486 | 86.1307 |
| 28.92631 | -10.103 | 29.13709 |
| 30.50524 | -12.9528 | 56.97155 |
| 34.67153 | -34.1689 | 130.4176 |
| 32.36761 | 39.31971 | 83.69658 |
| 29.18936 | -10.1314 | 28.35243 |
| 30.86711 | -13.0212 | 57.56772 |
| 35.06603 | -34.078 | 130.6852 |
| 32.06981 | 38.29003 | 72.61464 |
| 29.51228 | -10.1868 | 28.61654 |
| 31.18495 | -13.0883 | 57.39205 |
| 35.37778 | -33.8761 | 129.5228 |
| 29.82601 | -10.2395 | 28.71831 |
| 31.62514 | -13.215 | 59.3005 |
| 35.75916 | -33.7588 | 129.5717 |
| 32.13202 | -13.3787 | 62.28408 |
| 36.22957 | -33.8151 | 131.1151 |
| 32.54692 | -13.486 | 63.67119 |
| 36.44549 | -33.4467 | 128.3973 |
| 32.89604 | -13.564 | 63.94669 |
| 36.84075 | -33.3452 | 128.6852 |
| 33.19929 | -13.6214 | 63.47182 |
| 37.05413 | -33.0897 | 126.0157 |
| 32.96427 | -11.6399 | 54.37245 |
| 33.34451 | -13.6043 | 60.47242 |
| 37.45364 | -32.9247 | 126.3922 |
| 33.33655 | -11.6678 | 55.10974 |
| 33.70957 | -13.693 | 61.02722 |
| 37.79228 | -32.8539 | 125.7947 |
| 33.62788 | -11.7253 | 54.56066 |
| 34.15688 | -13.8167 | 62.86042 |
| 37.9893 | -32.536 | 122.9884 |
| 34.27344 | -11.9447 | 59.52084 |
| 34.66547 | -13.9852 | 65.6045 |
| 38.33951 | -32.3617 | 122.6204 |
| 34.65975 | -12.0283 | 60.39419 |
| 34.88271 | -14.015 | 63.81689 |
| 38.55887 | -32.1175 | 120.2516 |
| 35.03785 | -12.1159 | 61.1242 |
| 35.18021 | -14.0923 | 63.28637 |
| 38.94746 | -31.934 | 120.5036 |
| 35.20624 | -12.1182 | 58.68742 |
| 35.56704 | -14.2058 | 64.10958 |
| 39.34901 | -31.8739 | 120.945 |
| 37.22217 | 37.16241 | 83.77675 |
| 35.56567 | -12.229 | 59.14241 |
| 35.95457 | -14.3294 | 64.92586 |
| 39.79326 | -31.7983 | 122.0122 |
| 37.4573 | 36.84815 | 82.13879 |
| 36.3824 | -14.471 | 66.31858 |
| 40.17719 | -31.6315 | 122.1697 |
| 38.37461 | 37.28958 | 90.47187 |
| 36.78102 | -14.5982 | 67.25721 |
| 40.4209 | -31.4301 | 120.2811 |
| 38.86237 | 37.25554 | 92.44217 |
| 37.25303 | -14.7492 | 69.23521 |
| 40.44256 | -30.9057 | 115.2289 |
| 39.03783 | 36.90269 | 89.91477 |
| 37.70271 | -14.8853 | 70.85483 |
| 41.06902 | -30.9751 | 118.9118 |
| 38.19317 | -15.0536 | 73.01882 |
| 41.29259 | -30.6755 | 116.8271 |
| 38.55138 | -15.1706 | 73.28921 |
| 41.66976 | -30.5271 | 116.9331 |
| 38.94931 | -15.3374 | 74.10504 |
| 42.07865 | -30.3445 | 117.4766 |
| 40.92576 | 36.61581 | 96.57222 |
| 39.07751 | -15.3682 | 71.20273 |
| 42.33185 | -30.1274 | 115.8726 |
| 41.14248 | 36.31268 | 94.68689 |
| 42.75986 | -29.9847 | 116.6757 |
| 39.532 | -15.5137 | 68.23015 |
| 43.01388 | -29.7358 | 115.1207 |
| 40.09692 | -15.7453 | 71.29058 |
| 43.21807 | -29.4187 | 112.9299 |
| 40.5183 | -15.915 | 72.39694 |
| 43.69918 | -29.3092 | 114.4399 |
| 40.95242 | -13.9579 | 73.6499 |
| 43.88381 | -28.9752 | 112.0395 |
| 41.09243 | -13.9234 | 71.06349 |
| 44.11899 | -28.6453 | 110.3387 |
| 41.47981 | -14.0435 | 71.70475 |
| 44.76093 | -28.6623 | 113.8994 |
| 41.92242 | -14.203 | 73.03851 |
| 44.84389 | -28.2289 | 110.2725 |
| 45.04336 | -27.815 | 108.1814 |
| 42.68035 | -14.5199 | 74.04196 |
| 45.37008 | -27.5185 | 107.7204 |
| 43.07601 | -14.671 | 74.73775 |
| 45.98746 | -27.431 | 110.8752 |
| 43.4115 | -14.7658 | 74.68119 |
| 46.1431 | -27.0677 | 108.2937 |
| 46.36523 | -26.714 | 106.5677 |
| 46.55192 | -26.2815 | 104.4421 |
| 46.83568 | -25.881 | 103.5166 |
| 45.15611 | -15.6864 | 79.25377 |
| 47.14022 | -25.477 | 102.8538 |
| 47.73536 | -25.3241 | 105.6299 |
| 45.67893 | -16.0792 | 77.33866 |
| 47.64302 | -24.5448 | 100.3173 |
| 56.07515 | 11.18679 | 194.0109 |
| 47.98794 | -24.2462 | 100.1653 |
| 55.15779 | 10.87597 | 178.5744 |
| 55.67403 | 10.91273 | 179.7545 |
| 55.74554 | 10.81815 | 175.8779 |
| 56.05541 | 10.8519 | 174.7414 |
| 56.718 | 10.90082 | 177.5556 |
| 57.41275 | 10.97348 | 180.6819 |
| 57.85981 | 11.00655 | 181.0408 |
| 58.23875 | 11.02281 | 180.652 |
| 58.37083 | 10.94845 | 177.5999 |

Since Figures 20, 21, 22 and 23 in this paper show the 3-dimensional displacement field of the NEPE test specimen, which has too many data points, only some of the data in Figure 20a are provided here. Displacement field data in the tensile direction at time 12 s in Fig. 20 (a):

| $X'$ | $Y'$ | $u_{t}$ | $X'$ | $Y'$ | $u_{t}$ | $X'$ | $Y'$ | $u_{t}$ |
| --- | --- | --- | --- | --- | --- | --- | --- | --- |
| 14.375 | 14.375 | 14.11111 | 14.11111 | 14.02083 | 13.82083 | 13.70417 | 14.02083 | 14.925 |
| 14.585 | 14.585 | 14.33333 | 14.33333 | 14.22917 | 14.02917 | 13.94583 | 14.22917 | 14.975 |
| 14.795 | 14.795 | 14.55556 | 14.55556 | 14.4375 | 14.2375 | 14.1875 | 14.4375 | 15.025 |
| 15.005 | 15.005 | 14.77778 | 14.77778 | 14.64583 | 14.44583 | 14.42917 | 14.64583 | 15.075 |
| 15.215 | 15.215 | 15 | 15 | 14.85417 | 14.65417 | 14.67083 | 14.85417 | 15.125 |
| 15.425 | 15.425 | 15.22222 | 15.22222 | 15.0625 | 14.8625 | 14.9125 | 15.0625 | 15.175 |
| 15.635 | 15.635 | 15.44444 | 15.44444 | 15.27083 | 15.07083 | 15.15417 | 15.27083 | 15.3 |
| 15.845 | 15.845 | 15.66667 | 15.66667 | 15.47917 | 15.27917 | 15.39583 | 15.47917 | 15.5 |
| 16.055 | 16.055 | 15.88889 | 15.88889 | 15.6875 | 15.4875 | 15.6375 | 15.6875 | 15.7 |
| 16.265 | 16.265 | 16.1 | 16.1 | 15.89583 | 15.69583 | 15.87917 | 15.89583 | 15.9 |
| 16.475 | 16.475 | 16.3 | 16.3 | 16.125 | 15.95 | 16.125 | 16.125 | 16.1 |
| 16.685 | 16.685 | 16.5 | 16.5 | 16.375 | 16.25 | 16.375 | 16.375 | 16.3 |
| 16.895 | 16.895 | 16.7 | 16.7 | 16.625 | 16.55 | 16.625 | 16.625 | 16.5 |
| 17.08333 | 17.08333 | 16.9 | 16.9 | 16.875 | 16.85 | 16.875 | 16.875 | 16.7 |
| 17.25 | 17.25 | 17.125 | 17.125 | 17.125 | 17.125 | 17.16667 | 17.16667 | 16.975 |
| 17.41667 | 17.41667 | 17.375 | 17.375 | 17.375 | 17.375 | 17.5 | 17.5 | 17.325 |
| 17.58333 | 17.58333 | 17.625 | 17.625 | 17.625 | 17.625 | 17.83333 | 17.83333 | 17.675 |
| 17.75 | 17.75 | 17.875 | 17.875 | 17.875 | 17.875 | 18.16667 | 18.16667 | 18.025 |
| 17.91667 | 17.91667 | 18.125 | 18.125 | 18.125 | 18.125 | 18.5 | 18.5 | 18.3 |
| 18.155 | 18.155 | 18.375 | 18.375 | 18.375 | 18.375 | 18.77778 | 18.77778 | 18.5 |
| 18.465 | 18.465 | 18.625 | 18.625 | 18.625 | 18.625 | 19 | 19 | 18.7 |
| 18.775 | 18.775 | 18.875 | 18.875 | 18.875 | 18.875 | 19.22222 | 19.22222 | 18.9 |
| 19.085 | 19.085 | 19.11429 | 19.11429 | 19.11429 | 19.12286 | 19.44444 | 19.44444 | 19.1 |
| 19.395 | 19.395 | 19.34286 | 19.34286 | 19.34286 | 19.36857 | 19.66667 | 19.66667 | 19.3 |
| 19.705 | 19.705 | 19.57143 | 19.57143 | 19.57143 | 19.61429 | 19.88889 | 19.88889 | 19.5 |
| 20.015 | 20.015 | 19.8 | 19.8 | 19.8 | 19.86 | 20.08333 | 20.08333 | 19.7 |
| 20.325 | 20.325 | 20.02857 | 20.02857 | 20.02857 | 20.10571 | 20.25 | 20.25 | 19.9 |
| 20.58857 | 20.58857 | 20.25714 | 20.25714 | 20.25714 | 20.35143 | 20.41667 | 20.41667 | 20.1 |
| 20.80571 | 20.80571 | 20.48571 | 20.48571 | 20.48571 | 20.59714 | 20.58333 | 20.58333 | 20.3 |
| 21.02286 | 21.02286 | 20.6875 | 20.6875 | 20.7 | 20.81143 | 20.75 | 20.75 | 20.5 |
| 21.24 | 21.24 | 20.8625 | 20.8625 | 20.9 | 20.99429 | 20.91667 | 20.91667 | 20.7 |
| 21.45714 | 21.45714 | 21.0375 | 21.0375 | 21.1 | 21.17714 | 21.1 | 21.1 | 20.9 |
| 21.67429 | 21.67429 | 21.2125 | 21.2125 | 21.3 | 21.36 | 21.3 | 21.3 | 21.125 |
| 21.89143 | 21.89143 | 21.3875 | 21.3875 | 21.5 | 21.54286 | 21.5 | 21.5 | 21.375 |
| 22.075 | 22.075 | 21.5625 | 21.5625 | 21.7 | 21.72571 | 21.7 | 21.7 | 21.625 |
| 22.225 | 22.225 | 21.7375 | 21.7375 | 21.9 | 21.90857 | 21.9 | 21.9 | 21.875 |
| 22.375 | 22.375 | 21.9125 | 21.9125 | 22.125 | 22.125 | 22.14583 | 22.14583 | 22.125 |
| 22.525 | 22.525 | 22.13889 | 22.13889 | 22.375 | 22.375 | 22.4375 | 22.4375 | 22.375 |
| 22.70667 | 22.70667 | 22.41667 | 22.41667 | 22.625 | 22.625 | 22.72917 | 22.72917 | 22.625 |
| 22.92 | 22.92 | 22.69444 | 22.69444 | 22.875 | 22.875 | 23.02083 | 23.02083 | 22.875 |
| 23.13333 | 23.13333 | 22.97222 | 22.97222 | 23.125 | 23.125 | 23.3125 | 23.3125 | 23.11429 |
| 23.34667 | 23.34667 | 23.25 | 23.25 | 23.375 | 23.375 | 23.60417 | 23.60417 | 23.34286 |
| 23.56 | 23.56 | 23.52778 | 23.52778 | 23.625 | 23.625 | 23.89583 | 23.89583 | 23.57143 |
| 23.7619 | 23.7619 | 23.77778 | 23.77778 | 23.875 | 23.875 | 24.1875 | 24.1875 | 23.8 |
| 23.95238 | 23.95238 | 24 | 24 | 24.11429 | 24.11429 | 24.40417 | 24.40417 | 24.02857 |
| 24.14286 | 24.14286 | 24.22222 | 24.22222 | 24.34286 | 24.34286 | 24.54583 | 24.54583 | 24.25714 |
| 24.33333 | 24.33333 | 24.44444 | 24.44444 | 24.57143 | 24.57143 | 24.6875 | 24.6875 | 24.48571 |
| 24.52381 | 24.52381 | 24.66667 | 24.66667 | 24.8 | 24.8 | 24.82917 | 24.82917 | 24.6875 |
| 24.71428 | 24.71428 | 24.88889 | 24.88889 | 25.02857 | 25.02857 | 24.97083 | 24.97083 | 24.8625 |
| 24.90476 | 24.90476 | 25.08333 | 25.08333 | 25.25714 | 25.25714 | 25.1125 | 25.1125 | 25.0375 |
| 25.09524 | 25.09524 | 25.25 | 25.25 | 25.48571 | 25.48571 | 25.25417 | 25.25417 | 25.2125 |
| 25.28571 | 25.28571 | 25.41667 | 25.41667 | 25.69394 | 25.69697 | 25.39583 | 25.39583 | 25.3875 |
| 25.47619 | 25.47619 | 25.58333 | 25.58333 | 25.88182 | 25.89091 | 25.5375 | 25.5375 | 25.5625 |
| 25.70635 | 25.70635 | 25.75 | 25.75 | 26.0697 | 26.08485 | 25.67917 | 25.67917 | 25.7375 |
| 25.97619 | 25.97619 | 25.91667 | 25.91667 | 26.25758 | 26.27879 | 25.875 | 25.875 | 25.9125 |
| 26.24603 | 26.24603 | 26.125 | 26.125 | 26.44545 | 26.47273 | 26.125 | 26.125 | 26.1175 |
| 26.51587 | 26.51587 | 26.375 | 26.375 | 26.63333 | 26.66667 | 26.375 | 26.375 | 26.3525 |
| 26.78571 | 26.78571 | 26.625 | 26.625 | 26.82121 | 26.86061 | 26.625 | 26.625 | 26.5875 |
| 27.05556 | 27.05556 | 26.875 | 26.875 | 27.00909 | 27.05454 | 26.875 | 26.875 | 26.8225 |
| 27.3254 | 27.3254 | 27.125 | 27.125 | 27.19697 | 27.24848 | 27.125 | 27.125 | 27.0575 |
| 27.59524 | 27.59524 | 27.375 | 27.375 | 27.38485 | 27.44242 | 27.375 | 27.375 | 27.2925 |
| 27.86508 | 27.86508 | 27.625 | 27.625 | 27.57273 | 27.63636 | 27.625 | 27.625 | 27.5275 |
| 28.06667 | 28.06667 | 27.875 | 27.875 | 27.775 | 27.825 | 27.83928 | 27.83928 | 27.7625 |
| 28.2 | 28.2 | 28.09722 | 28.09722 | 27.99167 | 28.00833 | 28.01786 | 28.01786 | 27.96 |
| 28.33333 | 28.33333 | 28.29167 | 28.29167 | 28.20833 | 28.19167 | 28.19643 | 28.19643 | 28.12 |
| 28.46667 | 28.46667 | 28.48611 | 28.48611 | 28.425 | 28.375 | 28.375 | 28.375 | 28.28 |
| 28.6 | 28.6 | 28.68056 | 28.68056 | 28.64167 | 28.55833 | 28.55357 | 28.55357 | 28.44 |
| 28.77778 | 28.77778 | 28.875 | 28.875 | 28.85833 | 28.74167 | 28.73214 | 28.73214 | 28.6 |
| 29 | 29 | 29.06944 | 29.06944 | 29.075 | 28.925 | 28.91072 | 28.91072 | 28.76 |
| 29.22222 | 29.22222 | 29.26389 | 29.26389 | 29.29167 | 29.10833 | 29.1 | 29.1 | 28.92 |
| 29.44444 | 29.44444 | 29.45833 | 29.45833 | 29.475 | 29.3 | 29.3 | 29.3 | 29.125 |
| 29.66667 | 29.66667 | 29.65278 | 29.65278 | 29.625 | 29.5 | 29.5 | 29.5 | 29.375 |
| 29.88889 | 29.88889 | 29.85417 | 29.85417 | 29.775 | 29.7 | 29.7 | 29.7 | 29.625 |
| 30.1 | 30.1 | 30.0625 | 30.0625 | 29.925 | 29.9 | 29.9 | 29.9 | 29.875 |
| 30.3 | 30.3 | 30.27083 | 30.27083 | 30.125 | 30.125 | 30.125 | 30.125 | 30.125 |
| 30.5 | 30.5 | 30.47917 | 30.47917 | 30.375 | 30.375 | 30.375 | 30.375 | 30.375 |
| 30.7 | 30.7 | 30.6875 | 30.6875 | 30.625 | 30.625 | 30.625 | 30.625 | 30.625 |
| 30.9 | 30.9 | 30.89583 | 30.89583 | 30.875 | 30.875 | 30.875 | 30.875 | 30.875 |
| 31.14 | 31.14 | 31.14 | 31.14 | 31.18 | 31.17333 | 31.16667 | 31.16 | 31.175 |
| 31.42 | 31.42 | 31.42 | 31.42 | 31.54 | 31.52 | 31.5 | 31.48 | 31.525 |
| 31.7 | 31.7 | 31.7 | 31.7 | 31.9 | 31.86667 | 31.83333 | 31.8 | 31.875 |
| 31.98 | 31.98 | 31.98 | 31.98 | 32.26 | 32.21333 | 32.16667 | 32.12 | 32.225 |
| 32.26 | 32.26 | 32.26 | 32.26 | 32.62 | 32.56 | 32.5 | 32.44 | 32.525 |
| 32.56 | 32.56 | 32.56 | 32.56 | 32.92 | 32.86 | 32.8 | 32.74 | 32.775 |
| 32.88 | 32.88 | 32.88 | 32.88 | 33.16 | 33.11333 | 33.06667 | 33.02 | 33.025 |
| 33.2 | 33.2 | 33.2 | 33.2 | 33.4 | 33.36667 | 33.33334 | 33.3 | 33.275 |
| 33.52 | 33.52 | 33.52 | 33.52 | 33.64 | 33.62 | 33.6 | 33.58 | 33.5127 |
| 33.84 | 33.84 | 33.84 | 33.84 | 33.88 | 33.87333 | 33.86667 | 33.86 | 33.7381 |
| 34.11428 | 34.11428 | 34.11428 | 34.11428 | 34.11428 | 34.11428 | 34.10417 | 34.10417 | 33.96349 |
| 34.34286 | 34.34286 | 34.34286 | 34.34286 | 34.34286 | 34.34286 | 34.3125 | 34.3125 | 34.18889 |
| 34.57143 | 34.57143 | 34.57143 | 34.57143 | 34.57143 | 34.57143 | 34.52083 | 34.52083 | 34.41428 |
| 34.8 | 34.8 | 34.8 | 34.8 | 34.8 | 34.8 | 34.72917 | 34.72917 | 34.63968 |
| 35.02857 | 35.02857 | 35.02857 | 35.02857 | 35.02857 | 35.02857 | 34.9375 | 34.9375 | 34.86508 |
| 35.25714 | 35.25714 | 35.25714 | 35.25714 | 35.25714 | 35.25714 | 35.14584 | 35.14584 | 35.09048 |
| 35.48571 | 35.48571 | 35.48571 | 35.48571 | 35.48571 | 35.48571 | 35.35417 | 35.35417 | 35.31587 |
| 35.68571 | 35.68571 | 35.68571 | 35.68571 | 35.71515 | 35.71212 | 35.5625 | 35.5625 | 35.54545 |
| 35.85714 | 35.85714 | 35.85714 | 35.85714 | 35.94545 | 35.93636 | 35.7729 | 35.7729 | 35.77922 |
| 36.02857 | 36.02857 | 36.02857 | 36.02857 | 36.17575 | 36.16061 | 35.98535 | 35.98535 | 36.01299 |
| 36.2 | 36.2 | 36.2 | 36.2 | 36.40606 | 36.38485 | 36.1978 | 36.1978 | 36.24675 |
| 36.37143 | 36.37143 | 36.37143 | 36.37143 | 36.63636 | 36.60909 | 36.41026 | 36.41026 | 36.48052 |
| 36.54285 | 36.54285 | 36.54285 | 36.54285 | 36.86666 | 36.83333 | 36.62271 | 36.62271 | 36.71429 |
| 36.71429 | 36.71429 | 36.71429 | 36.71429 | 37.09697 | 37.05758 | 36.83516 | 36.83516 | 36.94805 |
| 36.91666 | 36.91666 | 36.91666 | 36.91666 | 37.32727 | 37.28182 | 37.04762 | 37.04762 | 37.18182 |
| 37.15 | 37.15 | 37.15 | 37.15 | 37.55758 | 37.50606 | 37.26007 | 37.26007 | 37.41558 |
| 37.38333 | 37.38333 | 37.38333 | 37.38333 | 37.78788 | 37.7303 | 37.47253 | 37.47253 | 37.64935 |
| 37.61667 | 37.61667 | 37.61667 | 37.61667 | 38.01818 | 37.95454 | 37.68498 | 37.68498 | 37.88312 |
| 37.85 | 37.85 | 37.85 | 37.85 | 38.175 | 38.125 | 37.89743 | 37.89743 | 38.08333 |
| 38.08334 | 38.08334 | 38.08334 | 38.08334 | 38.25833 | 38.24166 | 38.10989 | 38.10989 | 38.25 |
| 38.31667 | 38.31667 | 38.31667 | 38.31667 | 38.34166 | 38.35833 | 38.32234 | 38.32234 | 38.41667 |
| 38.55 | 38.55 | 38.55 | 38.55 | 38.425 | 38.475 | 38.53809 | 38.53809 | 38.58333 |
| 38.74762 | 38.74762 | 38.7875 | 38.7875 | 38.50833 | 38.59167 | 38.75714 | 38.75714 | 38.75 |
| 38.90952 | 38.90952 | 39.02917 | 39.02917 | 38.59167 | 38.70833 | 38.97619 | 38.97619 | 38.91667 |
| 39.07143 | 39.07143 | 39.27083 | 39.27083 | 38.675 | 38.825 | 39.19524 | 39.19524 | 39.1125 |
| 39.23333 | 39.23333 | 39.5125 | 39.5125 | 38.75833 | 38.94167 | 39.41428 | 39.41428 | 39.3375 |
| 39.39524 | 39.39524 | 39.75417 | 39.75417 | 38.95833 | 39.13333 | 39.63334 | 39.63334 | 39.5625 |
| 39.55714 | 39.55714 | 39.99583 | 39.99583 | 39.275 | 39.4 | 39.85238 | 39.85238 | 39.7875 |
| 39.71905 | 39.71905 | 40.2375 | 40.2375 | 39.59167 | 39.66666 | 40.07143 | 40.07143 | 40.0125 |
| 39.91 | 39.91 | 40.47916 | 40.47916 | 39.90833 | 39.93333 | 40.29048 | 40.29048 | 40.2375 |
| 40.13 | 40.13 | 40.625 | 40.625 | 40.225 | 40.2 | 40.475 | 40.475 | 40.4875 |
| 40.35 | 40.35 | 40.675 | 40.675 | 40.54167 | 40.46667 | 40.625 | 40.625 | 40.7625 |
| 40.57 | 40.57 | 40.725 | 40.725 | 40.87857 | 40.77619 | 40.775 | 40.775 | 41.0375 |
| 40.79 | 40.79 | 40.775 | 40.775 | 41.23571 | 41.12857 | 40.925 | 40.925 | 41.3125 |
| 41.07889 | 41.07889 | 41 | 41 | 41.59286 | 41.48095 | 41.16667 | 41.16667 | 41.5875 |
| 41.43667 | 41.43667 | 41.4 | 41.4 | 41.95 | 41.83333 | 41.5 | 41.5 | 41.8625 |
| 41.79445 | 41.79445 | 41.8 | 41.8 | 42.30714 | 42.18571 | 41.83333 | 41.83333 | 42.125 |
| 42.15223 | 42.15223 | 42.17667 | 42.17667 | 42.66429 | 42.53809 | 42.15179 | 42.15179 | 42.375 |
| 42.51 | 42.51 | 42.53 | 42.53 | 43.02143 | 42.89048 | 42.45536 | 42.45536 | 42.625 |
| 42.86778 | 42.86778 | 42.88334 | 42.88334 | 43.28929 | 43.17619 | 42.75893 | 42.75893 | 42.875 |
| 43.22556 | 43.22556 | 43.23667 | 43.23667 | 43.46786 | 43.39524 | 43.0625 | 43.0625 | 43.225 |
| 43.58334 | 43.58334 | 43.59 | 43.59 | 43.64643 | 43.61428 | 43.36607 | 43.36607 | 43.675 |
| 43.94111 | 43.94111 | 43.94334 | 43.94334 | 43.825 | 43.83333 | 43.66964 | 43.66964 | 44.125 |
| 44.19714 | 44.19714 | 44.19714 | 44.19714 | 44.00357 | 44.05238 | 43.97321 | 43.97321 | 44.575 |
| 44.35143 | 44.35143 | 44.35143 | 44.35143 | 44.18214 | 44.27143 | 44.27678 | 44.27678 | 44.85714 |
| 44.50571 | 44.50571 | 44.50571 | 44.50571 | 44.36071 | 44.49047 | 44.51905 | 44.51905 | 44.97143 |
| 44.66 | 44.66 | 44.66 | 44.66 | 44.56071 | 44.7 | 44.7 | 44.7 | 45.08571 |
| 44.81429 | 44.81429 | 44.81429 | 44.81429 | 44.78214 | 44.9 | 44.88095 | 44.88095 | 45.2 |
| 44.96857 | 44.96857 | 44.96857 | 44.96857 | 45.00357 | 45.1 | 45.0619 | 45.0619 | 45.31429 |
| 45.12286 | 45.12286 | 45.12286 | 45.12286 | 45.225 | 45.3 | 45.24286 | 45.24286 | 45.42857 |
| 45.3 | 45.3 | 45.3 | 45.3 | 45.44643 | 45.5 | 45.42381 | 45.42381 | 45.54285 |
| 45.5 | 45.5 | 45.5 | 45.5 | 45.66786 | 45.7 | 45.60476 | 45.60476 | 45.6875 |
| 45.7 | 45.7 | 45.7 | 45.7 | 45.88929 | 45.9 | 45.78571 | 45.78571 | 45.8625 |
| 45.9 | 45.9 | 45.9 | 45.9 | 46.08333 | 46.08333 | 45.96666 | 45.96666 | 46.0375 |
| 46.10417 | 46.10417 | 46.1 | 46.1 | 46.25 | 46.25 | 46.14762 | 46.14762 | 46.2125 |
| 46.3125 | 46.3125 | 46.3 | 46.3 | 46.41667 | 46.41667 | 46.32857 | 46.32857 | 46.3875 |
| 46.52083 | 46.52083 | 46.5 | 46.5 | 46.58333 | 46.58333 | 46.50952 | 46.50952 | 46.5625 |
| 46.72917 | 46.72917 | 46.6875 | 46.6875 | 46.75 | 46.75 | 46.7 | 46.7 | 46.7375 |
| 46.9375 | 46.9375 | 46.8625 | 46.8625 | 46.91667 | 46.91667 | 46.9 | 46.9 | 46.9125 |
| 47.14584 | 47.14584 | 47.0375 | 47.0375 | 47.1 | 47.1 | 47.1 | 47.1 | 47.1 |
| 47.35417 | 47.35417 | 47.2125 | 47.2125 | 47.3 | 47.3 | 47.3 | 47.3 | 47.3 |
| 47.5625 | 47.5625 | 47.3875 | 47.3875 | 47.5 | 47.5 | 47.5 | 47.5 | 47.5 |
| 47.7875 | 47.7875 | 47.5625 | 47.5625 | 47.7 | 47.7 | 47.7 | 47.7 | 47.7 |
| 48.02917 | 48.02917 | 47.7375 | 47.7375 | 47.9 | 47.9 | 47.9 | 47.9 | 47.9 |
| 48.27083 | 48.27083 | 47.9125 | 47.9125 | 48.125 | 48.125 | 48.125 | 48.125 | 48.125 |
| 48.5125 | 48.5125 | 48.16 | 48.16 | 48.375 | 48.375 | 48.375 | 48.375 | 48.375 |
| 48.75417 | 48.75417 | 48.48 | 48.48 | 48.625 | 48.625 | 48.625 | 48.625 | 48.625 |
| 48.99583 | 48.99583 | 48.8 | 48.8 | 48.875 | 48.875 | 48.875 | 48.875 | 48.875 |
| 49.2375 | 49.2375 | 49.12 | 49.12 | 49.125 | 49.125 | 49.125 | 49.125 | 49.125 |
| 49.47916 | 49.47916 | 49.44 | 49.44 | 49.375 | 49.375 | 49.375 | 49.375 | 49.375 |
| 49.70158 | 49.70158 | 49.69583 | 49.69583 | 49.625 | 49.625 | 49.625 | 49.625 | 49.625 |
| 49.90476 | 49.90476 | 49.8875 | 49.8875 | 49.875 | 49.875 | 49.875 | 49.875 | 49.875 |
| 50.10793 | 50.10793 | 50.07917 | 50.07917 | 50.11428 | 50.11428 | 50.11428 | 50.11428 | 50.11428 |
| 50.31111 | 50.31111 | 50.27083 | 50.27083 | 50.34286 | 50.34286 | 50.34286 | 50.34286 | 50.34286 |
| 50.51428 | 50.51428 | 50.4625 | 50.4625 | 50.57143 | 50.57143 | 50.57143 | 50.57143 | 50.57143 |
| 50.71746 | 50.71746 | 50.65417 | 50.65417 | 50.8 | 50.8 | 50.8 | 50.8 | 50.8 |
| 50.92063 | 50.92063 | 50.83929 | 50.83929 | 51.02857 | 51.02857 | 51.02857 | 51.02857 | 51.02857 |
| 51.12381 | 51.12381 | 51.01786 | 51.01786 | 51.25714 | 51.25714 | 51.25714 | 51.25714 | 51.25714 |
| 51.32698 | 51.32698 | 51.19643 | 51.19643 | 51.48571 | 51.48571 | 51.48571 | 51.48571 | 51.48571 |
| 51.51714 | 51.51714 | 51.375 | 51.375 | 51.6875 | 51.6875 | 51.7 | 51.706 | 51.7 |
| 51.69428 | 51.69428 | 51.55357 | 51.55357 | 51.8625 | 51.8625 | 51.9 | 51.918 | 51.9 |
| 51.87143 | 51.87143 | 51.73214 | 51.73214 | 52.0375 | 52.0375 | 52.1 | 52.13 | 52.1 |
| 52.04857 | 52.04857 | 51.91071 | 51.91071 | 52.2125 | 52.2125 | 52.3 | 52.342 | 52.3 |
| 52.22572 | 52.22572 | 52.1 | 52.1 | 52.3875 | 52.3875 | 52.5 | 52.554 | 52.5 |
| 52.40285 | 52.40285 | 52.3 | 52.3 | 52.5625 | 52.5625 | 52.7 | 52.766 | 52.7 |
| 52.58 | 52.58 | 52.5 | 52.5 | 52.7375 | 52.7375 | 52.9 | 52.978 | 52.9 |
| 52.75714 | 52.75714 | 52.7 | 52.7 | 52.9125 | 52.9125 | 53.1 | 53.19 | 53.1 |
| 52.93429 | 52.93429 | 52.9 | 52.9 | 53.1 | 53.1 | 53.3 | 53.402 | 53.3 |
| 53.11143 | 53.11143 | 53.1 | 53.1 | 53.3 | 53.3 | 53.5 | 53.614 | 53.5 |
| 53.32222 | 53.32222 | 53.38 | 53.38 | 53.5 | 53.5 | 53.6875 | 53.8 | 53.7 |
| 53.56667 | 53.56667 | 53.74 | 53.74 | 53.7 | 53.7 | 53.8625 | 53.96 | 53.9 |
| 53.81111 | 53.81111 | 54.1 | 54.1 | 53.9 | 53.9 | 54.0375 | 54.12 | 54.1 |
| 54.05556 | 54.05556 | 54.46 | 54.46 | 54.1 | 54.1 | 54.2125 | 54.28 | 54.3 |
| 54.3 | 54.3 | 54.82 | 54.82 | 54.3 | 54.3 | 54.3875 | 54.44 | 54.5 |
| 54.54445 | 54.54445 | 55.025 | 55.025 | 54.5 | 54.5 | 54.5625 | 54.6 | 54.7 |
| 54.77778 | 54.77778 | 55.075 | 55.075 | 54.71 | 54.71 | 54.7375 | 54.76 | 54.9 |
| 55 | 55 | 55.125 | 55.125 | 54.93 | 54.93 | 54.9125 | 54.92 | 55.11428 |
| 55.22222 | 55.22222 | 55.175 | 55.175 | 55.15 | 55.15 | 55.1125 | 55.1225 | 55.34286 |
| 55.44445 | 55.44445 | 55.3125 | 55.3125 | 55.37 | 55.37 | 55.3375 | 55.3675 | 55.57143 |
| 55.66667 | 55.66667 | 55.5375 | 55.5375 | 55.59 | 55.59 | 55.5625 | 55.6125 | 55.8 |
| 55.88889 | 55.88889 | 55.7625 | 55.7625 | 55.81 | 55.81 | 55.7875 | 55.8575 | 56.02857 |
| 56.0925 | 56.0925 | 55.9875 | 55.9875 | 56.03 | 56.03 | 56.0125 | 56.1025 | 56.25714 |
| 56.2775 | 56.2775 | 56.2125 | 56.2125 | 56.25 | 56.25 | 56.2375 | 56.3475 | 56.48571 |
| 56.4625 | 56.4625 | 56.4375 | 56.4375 | 56.47 | 56.47 | 56.4625 | 56.5925 | 56.68611 |
| 56.6475 | 56.6475 | 56.6625 | 56.6625 | 56.69 | 56.69 | 56.6875 | 56.8375 | 56.85833 |
| 56.8325 | 56.8325 | 56.8875 | 56.8875 | 56.87143 | 56.88571 | 56.9 | 57.06285 | 57.03056 |
| 57.0175 | 57.0175 | 57.08148 | 57.08148 | 57.01429 | 57.05714 | 57.1 | 57.26857 | 57.20278 |
| 57.2025 | 57.2025 | 57.24445 | 57.24445 | 57.15714 | 57.22857 | 57.3 | 57.47429 | 57.375 |
| 57.3875 | 57.3875 | 57.40741 | 57.40741 | 57.3 | 57.4 | 57.5 | 57.68 | 57.54722 |
| 57.57428 | 57.57428 | 57.57037 | 57.57037 | 57.44286 | 57.57143 | 57.7 | 57.88572 | 57.71944 |
| 57.76286 | 57.76286 | 57.73333 | 57.73333 | 57.58571 | 57.74286 | 57.9 | 58.09143 | 57.89167 |
| 57.95143 | 57.95143 | 57.89629 | 57.89629 | 57.72857 | 57.91428 | 58.1 | 58.29715 | 58.06389 |
| 58.14 | 58.14 | 58.05926 | 58.05926 | 57.925 | 58.125 | 58.325 | 58.525 | 58.28214 |
| 58.32857 | 58.32857 | 58.22222 | 58.22222 | 58.175 | 58.375 | 58.575 | 58.775 | 58.54643 |
| 58.51714 | 58.51714 | 58.38518 | 58.38518 | 58.425 | 58.625 | 58.825 | 59.025 | 58.81071 |
| 58.70572 | 58.70572 | 58.62 | 58.62 | 58.675 | 58.875 | 59.075 | 59.275 | 59.075 |
| 58.95 | 58.95 | 58.92667 | 58.92667 | 58.92857 | 59.11428 | 59.29428 | 59.48571 | 59.33929 |
| 59.25 | 59.25 | 59.23333 | 59.23333 | 59.18571 | 59.34286 | 59.48286 | 59.65714 | 59.60357 |
| 59.55 | 59.55 | 59.54 | 59.54 | 59.44286 | 59.57143 | 59.67143 | 59.82857 | 59.86786 |
| 59.85 | 59.85 | 59.84666 | 59.84666 | 59.7 | 59.8 | 59.86 | 60 | 60.1 |
| 60.125 | 60.125 | 60.125 | 60.125 | 59.95714 | 60.02857 | 60.04857 | 60.17143 | 60.3 |
| 60.375 | 60.375 | 60.375 | 60.375 | 60.21428 | 60.25714 | 60.23714 | 60.34286 | 60.5 |
| 60.625 | 60.625 | 60.625 | 60.625 | 60.47143 | 60.48571 | 60.42572 | 60.51429 | 60.7 |
| 60.875 | 60.875 | 60.875 | 60.875 | 60.72 | 60.72 | 60.64389 | 60.71944 | 60.9 |
| 61.11428 | 61.11428 | 61.11428 | 61.11428 | 60.96 | 60.96 | 60.89167 | 60.95833 | 61.11428 |
| 61.34286 | 61.34286 | 61.34286 | 61.34286 | 61.2 | 61.2 | 61.13945 | 61.19722 | 61.34286 |
| 61.57143 | 61.57143 | 61.57143 | 61.57143 | 61.44 | 61.44 | 61.38722 | 61.43611 | 61.57143 |
| 61.8 | 61.8 | 61.8 | 61.8 | 61.68 | 61.68 | 61.635 | 61.675 | 61.8 |
| 62.02857 | 62.02857 | 62.02857 | 62.02857 | 61.92 | 61.92 | 61.88278 | 61.91389 | 62.02857 |
| 62.25714 | 62.25714 | 62.25714 | 62.25714 | 62.16 | 62.16 | 62.13055 | 62.15278 | 62.25714 |
| 62.48571 | 62.48571 | 62.48571 | 62.48571 | 62.4 | 62.4 | 62.37833 | 62.39167 | 62.48571 |
| 62.69394 | 62.69394 | 62.6875 | 62.6875 | 62.64 | 62.64 | 62.62611 | 62.63055 | 62.7 |
| 62.88182 | 62.88182 | 62.8625 | 62.8625 | 62.88 | 62.88 | 62.83929 | 62.83929 | 62.9 |
| 63.06969 | 63.06969 | 63.0375 | 63.0375 | 63.084 | 63.084 | 63.01786 | 63.01786 | 63.1 |
| 63.25757 | 63.25757 | 63.2125 | 63.2125 | 63.252 | 63.252 | 63.19643 | 63.19643 | 63.3 |
| 63.44545 | 63.44545 | 63.3875 | 63.3875 | 63.42 | 63.42 | 63.375 | 63.375 | 63.5 |
| 63.63333 | 63.63333 | 63.5625 | 63.5625 | 63.588 | 63.588 | 63.55357 | 63.55357 | 63.7 |
| 63.82121 | 63.82121 | 63.7375 | 63.7375 | 63.756 | 63.756 | 63.73214 | 63.73214 | 63.9 |
| 64.00909 | 64.00909 | 63.9125 | 63.9125 | 63.924 | 63.924 | 63.91071 | 63.91071 | 64.1 |
| 64.19697 | 64.19697 | 64.1 | 64.1 | 64.092 | 64.092 | 64.1 | 64.1 | 64.3 |
| 64.38485 | 64.38485 | 64.3 | 64.3 | 64.26 | 64.26 | 64.3 | 64.3 | 64.5 |
| 64.57272 | 64.57272 | 64.5 | 64.5 | 64.428 | 64.428 | 64.5 | 64.5 | 64.6875 |
| 64.75454 | 64.75454 | 64.7 | 64.7 | 64.596 | 64.596 | 64.7 | 64.7 | 64.8625 |
| 64.9303 | 64.9303 | 64.9 | 64.9 | 64.7625 | 64.7625 | 64.9 | 64.9 | 65.0375 |
| 65.10606 | 65.10606 | 65.1 | 65.1 | 64.9275 | 64.9275 | 65.1 | 65.1 | 65.2125 |
| 65.28181 | 65.28181 | 65.3 | 65.3 | 65.0925 | 65.0925 | 65.3 | 65.3 | 65.3875 |
| 65.45757 | 65.45757 | 65.5 | 65.5 | 65.2575 | 65.2575 | 65.5 | 65.5 | 65.5625 |
| 65.63333 | 65.63333 | 65.6875 | 65.6875 | 65.4225 | 65.4225 | 65.7 | 65.7 | 65.7375 |
| 65.80909 | 65.80909 | 65.8625 | 65.8625 | 65.5875 | 65.5875 | 65.9 | 65.9 | 65.9125 |
| 65.98485 | 65.98485 | 66.0375 | 66.0375 | 65.7525 | 65.7525 | 66.1 | 66.1 | 66.1 |
| 66.16061 | 66.16061 | 66.2125 | 66.2125 | 65.9175 | 65.9175 | 66.3 | 66.3 | 66.3 |
| 66.33636 | 66.33636 | 66.3875 | 66.3875 | 66.12 | 66.14 | 66.5 | 66.5 | 66.5 |
| 66.51212 | 66.51212 | 66.5625 | 66.5625 | 66.36 | 66.42 | 66.7 | 66.7 | 66.7 |
| 66.73333 | 66.73333 | 66.7375 | 66.7375 | 66.6 | 66.7 | 66.9 | 66.9 | 66.9 |
| 67 | 67 | 66.9125 | 66.9125 | 66.84 | 66.98 | 67.125 | 67.125 | 67.125 |
| 67.26666 | 67.26666 | 67.14545 | 67.14545 | 67.07999 | 67.26 | 67.375 | 67.375 | 67.375 |
| 67.53333 | 67.53333 | 67.43636 | 67.43636 | 67.425 | 67.6 | 67.625 | 67.625 | 67.625 |
| 67.8 | 67.8 | 67.72727 | 67.72727 | 67.875 | 68 | 67.875 | 67.875 | 67.875 |
| 68.06667 | 68.06667 | 68.01818 | 68.01818 | 68.325 | 68.4 | 68.14286 | 68.14286 | 68.14286 |
| 68.33333 | 68.33333 | 68.30909 | 68.30909 | 68.775 | 68.8 | 68.42857 | 68.42857 | 68.42857 |
| 68.6 | 68.6 | 68.6 | 68.6 | 69.225 | 69.2 | 68.71429 | 68.71429 | 68.71429 |
| 68.86667 | 68.86667 | 68.89091 | 68.89091 | 69.675 | 69.6 | 69 | 69 | 69 |
| 69.13333 | 69.13333 | 69.18182 | 69.18182 | 70.125 | 70 | 69.28571 | 69.28571 | 69.28571 |

Fig. 24 (a) Data on engineering principal strains in the direction of NEPE front and back stretching:

| x | front -y-strain | back-y-strain |
| --- | --- | --- |
| 1 | 0.003444 | 0.003995 |
| 2 | 0.017893 | 0.019548 |
| 3 | 0.038473 | 0.039991 |
| 4 | 0.059504 | 0.061239 |
| 5 | 0.081198 | 0.081728 |
| 6 | 0.101893 | 0.106513 |
| 7 | 0.123513 | 0.12824 |
| 8 | 0.144789 | 0.149239 |
| 12 | 0.233964 | 0.24996 |
| 16 | 0.321174 | 0.320562 |
| 20 | 0.402911 | 0.403961 |
| 24 | 0.486964 | 0.486307 |
| 28 | 0.575201 | 0.576553 |
| 32 | 0.665959 | 0.659607 |
| 36 | 0.757609 | 0.756009 |
| 40 | 0.847012 | 0.846831 |
| 44 | 0.935609 | 0.933796 |
| 48 | 1.020382 | 1.022865 |
| 52 | 1.102851 | 1.108425 |
| 56 | 1.177103 | 1.167985 |
| 60 | 1.244884 | 1.24207 |

Engineering principal strains in the width and thickness directions for NEPE in Fig. 24 (b):

| x | front-width-x-strain | back-width-x-strain | thickness-strain |
| --- | --- | --- | --- |
| 1 | -1.9E-16 | -0.00021 | -0.00276 |
| 2 | -0.00849 | -0.00945 | -0.00879 |
| 3 | -0.01878 | -0.0199 | -0.01944 |
| 4 | -0.02803 | -0.03096 | -0.02811 |
| 5 | -0.03899 | -0.04064 | -0.03706 |
| 6 | -0.04906 | -0.05241 | -0.04722 |
| 7 | -0.05747 | -0.06089 | -0.0564 |
| 8 | -0.06559 | -0.0749 | -0.06353 |
| 12 | -0.10076 | -0.11099 | -0.10044 |
| 16 | -0.13204 | -0.13687 | -0.12815 |
| 20 | -0.15824 | -0.16575 | -0.15437 |
| 24 | -0.18228 | -0.19035 | -0.17754 |
| 28 | -0.20539 | -0.21363 | -0.19965 |
| 32 | -0.22584 | -0.2332 | -0.21872 |
| 36 | -0.24315 | -0.25147 | -0.23564 |
| 40 | -0.2592 | -0.26664 | -0.25051 |
| 44 | -0.27302 | -0.28017 | -0.26353 |
| 48 | -0.28545 | -0.29241 | -0.27527 |
| 52 | -0.29557 | -0.30164 | -0.28447 |
| 56 | -0.30233 | -0.30771 | -0.29058 |
| 60 | -0.30889 | -0.3167 | -0.29794 |

Fig. 26 Specific data on volume rate of change and Poisson's ratio for NEPE:

| $\varepsilon_{e1}$ | 500mm/min$\frac{V}{V_{0}}$ | $\varepsilon_{e1}$ | 100mm/min$\frac{V}{V_{0}}$ | $\varepsilon_{e1}$ | 1mm/min$\frac{V}{V_{0}}$ | $\varepsilon_{e1}$ | 500mm/min$\nu$ | $\varepsilon_{e1}$ | 100mm/min$\nu$ | $\varepsilon_{e1}$ | 1mm/min$\nu$ |
| --- | --- | --- | --- | --- | --- | --- | --- | --- | --- | --- | --- |
| 0.027312 | 1.000417 | 0.003719 | 1.000847 | 0.00939 | 1.000956 | 0.027312 | 0.492262 | 0.01872 | 0.480879 | 0.00939 | 0.448887 |
| 0.126096 | 0.999952 | 0.01872 | 1.00071 | 0.024146 | 1.002366 | 0.126096 | 0.500201 | 0.039232 | 0.508801 | 0.024146 | 0.450481 |
| 0.230707 | 1.000598 | 0.039232 | 0.999323 | 0.034092 | 1.003021 | 0.230707 | 0.498561 | 0.060371 | 0.498535 | 0.034092 | 0.455009 |
| 0.330533 | 1.002683 | 0.060371 | 1.000172 | 0.047705 | 1.003885 | 0.330533 | 0.495308 | 0.081463 | 0.500469 | 0.047705 | 0.4584 |
| 0.441154 | 1.005927 | 0.081463 | 0.999927 | 0.0592 | 1.004501 | 0.441154 | 0.491914 | 0.104203 | 0.506638 | 0.0592 | 0.460961 |
| 0.555035 | 1.02125 | 0.104203 | 0.998685 | 0.070437 | 1.004797 | 0.555035 | 0.476186 | 0.125877 | 0.502052 | 0.070437 | 0.464849 |
| 0.666363 | 1.037503 | 0.125877 | 0.999514 | 0.082092 | 1.005232 | 0.666363 | 0.46395 | 0.147014 | 0.50477 | 0.082092 | 0.466928 |
| 0.782263 | 1.057102 | 0.147014 | 0.998692 | 0.093772 | 1.005007 | 0.782263 | 0.451953 | 0.241962 | 0.502469 | 0.093772 | 0.472138 |
| 0.893482 | 1.080235 | 0.241962 | 0.99893 | 0.145255 | 1.003659 | 0.893482 | 0.439555 | 0.320868 | 0.505838 | 0.145255 | 0.486535 |
| 1.000137 | 1.105497 | 0.320868 | 0.996756 | 0.207488 | 1.000036 | 1.000137 | 0.42766 | 0.403436 | 0.508082 | 0.207488 | 0.499904 |
| 1.1043 | 1.132895 | 0.403436 | 0.994537 | 0.25627 | 0.99613 | 1.1043 | 0.416143 | 0.486635 | 0.506466 | 0.25627 | 0.508498 |
| 1.19103 | 1.156104 | 0.486635 | 0.994885 | 0.311139 | 0.998302 | 1.19103 | 0.407534 | 0.575877 | 0.503292 | 0.311139 | 0.503136 |
| 1.274612 | 1.186202 | 0.575877 | 0.99701 | 0.36407 | 0.993534 | 1.274612 | 0.39611 | 0.662783 | 0.499084 | 0.36407 | 0.510447 |
|  |  | 0.662783 | 1.000932 | 0.420327 | 0.993815 |  |  | 0.756809 | 0.490529 | 0.420327 | 0.508841 |
|  |  | 0.756809 | 1.010731 | 0.47848 | 0.993859 |  |  | 0.846922 | 0.483628 | 0.47848 | 0.507876 |
|  |  | 0.846922 | 1.020292 | 0.538186 | 0.998291 |  |  | 0.934703 | 0.477055 | 0.538186 | 0.501986 |
|  |  | 0.934703 | 1.030748 | 0.599915 | 1.006504 |  |  | 1.021624 | 0.470906 | 0.599915 | 0.493103 |
|  |  | 1.021624 | 1.041809 | 0.659807 | 1.014243 |  |  | 1.105638 | 0.462935 | 0.659807 | 0.486045 |
|  |  | 1.105638 | 1.056751 | 0.724573 | 1.02138 |  |  | 1.172544 | 0.455716 | 0.724573 | 0.480592 |
|  |  | 1.172544 | 1.071136 | 0.784724 | 1.029576 |  |  | 1.243477 | 0.45101 | 0.784724 | 0.474841 |
|  |  | 1.243477 | 1.082389 | 0.857379 | 1.045295 |  |  |  |  | 0.857379 | 0.464227 |
|  |  |  |  | 0.926555 | 1.05738 |  |  |  |  | 0.926555 | 0.457456 |
|  |  |  |  | 0.992777 | 1.066322 |  |  |  |  | 0.992777 | 0.453435 |
|  |  |  |  | 1.050896 | 1.073233 |  |  |  |  | 1.050896 | 0.450802 |
|  |  |  |  | 1.148559 | 1.095066 |  |  |  |  | 1.148559 | 0.440629 |
|  |  |  |  | 1.212735 | 1.107565 |  |  |  |  | 1.212735 | 0.435684 |
|  |  |  |  | 1.281514 | 1.120916 |  |  |  |  | 1.281514 | 0.430807 |
|  |  |  |  | 1.348994 | 1.135245 |  |  |  |  | 1.348994 | 0.425732 |
|  |  |  |  | 1.415024 | 1.149239 |  |  |  |  | 1.415024 | 0.421119 |
|  |  |  |  | 1.482159 | 1.163736 |  |  |  |  | 1.482159 | 0.416604 |
|  |  |  |  | 1.552575 | 1.179973 |  |  |  |  | 1.552575 | 0.411701 |
|  |  |  |  | 1.620261 | 1.198818 |  |  |  |  | 1.620261 | 0.405875 |
|  |  |  |  | 1.689265 | 1.211185 |  |  |  |  | 1.689265 | 0.403161 |
|  |  |  |  | 1.759159 | 1.225194 |  |  |  |  | 1.759159 | 0.399944 |
|  |  |  |  | 1.827137 | 1.239533 |  |  |  |  | 1.827137 | 0.396689 |
|  |  |  |  | 1.89805 | 1.256279 |  |  |  |  | 1.89805 | 0.392789 |
|  |  |  |  | 1.965193 | 1.274822 |  |  |  |  | 1.965193 | 0.388308 |
|  |  |  |  | 2.035722 | 1.290515 |  |  |  |  | 2.035722 | 0.385163 |
|  |  |  |  | 2.099639 | 1.303715 |  |  |  |  | 2.099639 | 0.38278 |

True stress-strain curve data in Fig. 27 (a):

| $\varepsilon_{true}$ | $\sigma_{true}$500mm/min | $\varepsilon_{true}$ | $\sigma_{true}$100mm/min | $\varepsilon_{true}$ | $\sigma_{true}$ 100mm/min |
| --- | --- | --- | --- | --- | --- |
| 0.026945 | 0.076312 | 0.003712 | 0.012969 | 0.009346 | 0.003052 |
| 0.118757 | 0.186389 | 0.018547 | 0.028373 | 0.023859 | 0.010223 |
| 0.207589 | 0.345341 | 0.038482 | 0.045049 | 0.033524 | 0.017086 |
| 0.285579 | 0.490412 | 0.058619 | 0.061688 | 0.046602 | 0.024903 |
| 0.365444 | 0.620373 | 0.078315 | 0.078953 | 0.057514 | 0.032413 |
| 0.441498 | 0.735441 | 0.099124 | 0.098309 | 0.068067 | 0.04032 |
| 0.510644 | 0.845067 | 0.118562 | 0.116957 | 0.078896 | 0.048837 |
| 0.577884 | 0.946912 | 0.137162 | 0.138956 | 0.089632 | 0.057266 |
| 0.638417 | 1.002099 | 0.216692 | 0.223919 | 0.135627 | 0.093872 |
| 0.693216 | 1.037234 | 0.278289 | 0.316954 | 0.188542 | 0.136973 |
| 0.743983 | 1.054069 | 0.338923 | 0.415379 | 0.228147 | 0.185346 |
| 0.784372 | 1.057117 | 0.396515 | 0.498075 | 0.270896 | 0.230839 |
| 0.82181 | 1.053505 | 0.454812 | 0.596457 | 0.310473 | 0.270774 |
|  |  | 0.508493 | 0.692135 | 0.350887 | 0.307095 |
|  |  | 0.563499 | 0.795983 | 0.391015 | 0.341061 |
|  |  | 0.61352 | 0.858259 | 0.430604 | 0.37208 |
|  |  | 0.659954 | 0.908137 | 0.46995 | 0.400956 |
|  |  | 0.703901 | 0.94468 | 0.506701 | 0.426352 |
|  |  | 0.744618 | 0.968478 | 0.54498 | 0.450247 |
|  |  | 0.775899 | 0.97643 | 0.579264 | 0.470099 |
|  |  | 0.808027 | 0.983905 | 0.619166 | 0.48852 |
|  |  |  |  | 0.655734 | 0.506052 |
|  |  |  |  | 0.689529 | 0.522914 |
|  |  |  |  | 0.718277 | 0.53593 |
|  |  |  |  | 0.764798 | 0.551282 |
|  |  |  |  | 0.794229 | 0.563215 |
|  |  |  |  | 0.824839 | 0.575675 |
|  |  |  |  | 0.853987 | 0.585796 |
|  |  |  |  | 0.881709 | 0.595689 |
|  |  |  |  | 0.909129 | 0.60504 |
|  |  |  |  | 0.937103 | 0.614017 |
|  |  |  |  | 0.963274 | 0.623238 |
|  |  |  |  | 0.989268 | 0.634881 |
|  |  |  |  | 1.014926 | 0.64494 |
|  |  |  |  | 1.039265 | 0.654323 |
|  |  |  |  | 1.064038 | 0.663352 |
|  |  |  |  | 1.086942 | 0.669623 |
|  |  |  |  | 1.110449 | 0.676724 |
|  |  |  |  | 1.131286 | 0.674571 |

True stress-strain curve data in Fig. 27 (b):

| $\varepsilon_{true}$ | $\sigma_{true}$500mm/min | $\varepsilon_{true}$ | $\sigma_{true}$100mm/min | $\varepsilon_{true}$ | $\sigma_{true}$ 100mm/min |
| --- | --- | --- | --- | --- | --- |
| 0.001529 | 0.000582 | 0.02333 | 0.023959 | 0.01493 | 0.014058 |
| 0.001761 | 0.000569 | 0.03315 | 0.039577 | 0.02472 | 0.02655 |
| 0.001974 | 0.000653 | 0.04299 | 0.055271 | 0.03466 | 0.04536 |
| 0.002206 | 0.000614 | 0.05281 | 0.071706 | 0.04464 | 0.065304 |
| 0.002415 | 0.00055 | 0.06264 | 0.088141 | 0.05464 | 0.08451 |
| 0.002645 | 0.00115 | 0.07246 | 0.104576 | 0.06463 | 0.102438 |
| 0.002875 | 0.00118 | 0.08229 | 0.121391 | 0.07463 | 0.121248 |
| 0.003105 | 0.001065 | 0.09212 | 0.138035 | 0.08462 | 0.139194 |
| 0.003335 | 0.0012 | 0.10195 | 0.154432 | 0.09461 | 0.158526 |
| 0.003565 | 0.001176 | 0.11177 | 0.171095 | 0.10461 | 0.17775 |
| 0.003795 | 0.00169 | 0.1216 | 0.187416 | 0.1146 | 0.19692 |
| 0.004002 | 0.001355 | 0.13143 | 0.204212 | 0.1246 | 0.219366 |
| 0.004232 | 0.001457 | 0.14126 | 0.220343 | 0.13459 | 0.237582 |
| 0.004462 | 0.001499 | 0.15109 | 0.236512 | 0.14458 | 0.256266 |
| 0.004692 | 0.001792 | 0.16091 | 0.252301 | 0.15458 | 0.275022 |
| 0.004922 | 0.002062 | 0.17075 | 0.268071 | 0.16457 | 0.293778 |
| 0.005152 | 0.001711 | 0.18057 | 0.284183 | 0.17456 | 0.312444 |
| 0.005359 | 0.001922 | 0.1904 | 0.299345 | 0.18456 | 0.331992 |
| 0.005589 | 0.002295 | 0.20022 | 0.314602 | 0.19455 | 0.34884 |
| 0.005819 | 0.002105 | 0.21005 | 0.32984 | 0.20455 | 0.369342 |
| 0.006049 | 0.002369 | 0.21988 | 0.344907 | 0.21454 | 0.388296 |
| 0.006279 | 0.002369 | 0.22971 | 0.359632 | 0.22453 | 0.405504 |
| 0.006509 | 0.002507 | 0.23953 | 0.374509 | 0.23453 | 0.423576 |
| 0.006739 | 0.002737 | 0.24936 | 0.388607 | 0.24452 | 0.440442 |
| 0.006946 | 0.002829 | 0.25918 | 0.402895 | 0.25452 | 0.457704 |
| 0.007176 | 0.002852 | 0.26902 | 0.416746 | 0.26451 | 0.47421 |
| 0.007406 | 0.002829 | 0.27884 | 0.430407 | 0.2745 | 0.490698 |
| 0.007613 | 0.003151 | 0.28867 | 0.444182 | 0.2845 | 0.510318 |
| 0.007866 | 0.003128 | 0.29849 | 0.457558 | 0.29449 | 0.527022 |
| 0.008096 | 0.003243 | 0.30832 | 0.471048 | 0.30448 | 0.542718 |
| 0.008303 | 0.003404 | 0.31815 | 0.484025 | 0.31448 | 0.55917 |
| 0.008533 | 0.003519 | 0.32798 | 0.496812 | 0.32447 | 0.574074 |
| 0.008763 | 0.003657 | 0.33781 | 0.509048 | 0.33447 | 0.58986 |
| 0.00897 | 0.003634 | 0.34764 | 0.521303 | 0.34446 | 0.604314 |
| 0.0092 | 0.003749 | 0.35747 | 0.533691 | 0.35445 | 0.61974 |
| 0.009453 | 0.003979 | 0.36729 | 0.54587 | 0.36445 | 0.635166 |
| 0.00966 | 0.004117 | 0.37712 | 0.557574 | 0.37444 | 0.650628 |
| 0.00989 | 0.00391 | 0.38694 | 0.569164 | 0.38444 | 0.665154 |
| 0.01012 | 0.004094 | 0.39678 | 0.580051 | 0.39443 | 0.679248 |
| 0.010327 | 0.004117 | 0.4066 | 0.591413 | 0.40442 | 0.692676 |
| 0.010557 | 0.004439 | 0.41643 | 0.601996 | 0.41442 | 0.705852 |
| 0.010787 | 0.004669 | 0.42625 | 0.612978 | 0.42441 | 0.71937 |
| 0.011017 | 0.004531 | 0.43608 | 0.623086 | 0.4344 | 0.7326 |
| 0.011247 | 0.004577 | 0.44591 | 0.633745 | 0.4444 | 0.744516 |
| 0.011477 | 0.005106 | 0.45574 | 0.643359 | 0.45439 | 0.759132 |
| 0.011707 | 0.004669 | 0.46556 | 0.653049 | 0.46439 | 0.77094 |
| 0.011914 | 0.005244 | 0.47539 | 0.663081 | 0.47439 | 0.783792 |
| 0.012144 | 0.005083 | 0.48522 | 0.672334 | 0.48437 | 0.794178 |
| 0.012374 | 0.005497 | 0.49505 | 0.681454 | 0.49437 | 0.80514 |
| 0.012604 | 0.005451 | 0.50487 | 0.690479 | 0.50436 | 0.816642 |
| 0.012834 | 0.005543 | 0.5147 | 0.699086 | 0.51436 | 0.827424 |
| 0.013064 | 0.00575 | 0.52453 | 0.707408 | 0.52436 | 0.837612 |
| 0.013271 | 0.00598 | 0.53436 | 0.715768 | 0.53435 | 0.848898 |
| 0.013501 | 0.006095 | 0.54419 | 0.723767 | 0.54435 | 0.859914 |
| 0.013731 | 0.006003 | 0.55401 | 0.731728 | 0.55434 | 0.86832 |
| 0.013961 | 0.006233 | 0.56384 | 0.739252 | 0.56433 | 0.878166 |
| 0.014191 | 0.00644 | 0.57367 | 0.746757 | 0.57433 | 0.887472 |
| 0.014421 | 0.006601 | 0.5835 | 0.753825 | 0.58432 | 0.896598 |
| 0.014651 | 0.00667 | 0.59332 | 0.760665 | 0.59431 | 0.905022 |
| 0.014858 | 0.006486 | 0.60315 | 0.767524 | 0.60431 | 0.913968 |
| 0.015088 | 0.00667 | 0.61297 | 0.774136 | 0.6143 | 0.921816 |
| 0.015295 | 0.006946 | 0.62281 | 0.780767 | 0.6243 | 0.931122 |
| 0.015548 | 0.0069 | 0.63263 | 0.786714 | 0.63429 | 0.938412 |
| 0.015778 | 0.006992 | 0.64246 | 0.792661 | 0.64428 | 0.944424 |
| 0.015985 | 0.007199 | 0.65228 | 0.798722 | 0.65428 | 0.95229 |
| 0.016215 | 0.007521 | 0.66212 | 0.804023 | 0.66427 | 0.958374 |
| 0.016445 | 0.007843 | 0.67194 | 0.809495 | 0.67427 | 0.964206 |
| 0.016675 | 0.007728 | 0.68177 | 0.814454 | 0.68426 | 0.970776 |
| 0.016882 | 0.007544 | 0.6916 | 0.81966 | 0.69425 | 0.977616 |
| 0.017112 | 0.007958 | 0.70143 | 0.824315 | 0.70425 | 0.983826 |
| 0.017365 | 0.008441 | 0.71125 | 0.828723 | 0.71424 | 0.988722 |
| 0.017572 | 0.00805 | 0.72108 | 0.833454 | 0.72423 | 0.993168 |
| 0.017802 | 0.008142 | 0.73091 | 0.837235 | 0.73423 | 0.998262 |
| 0.018032 | 0.008257 | 0.74073 | 0.841282 | 0.74422 | 1.002942 |
| 0.018239 | 0.008579 | 0.75057 | 0.845044 | 0.75422 | 1.00764 |
| 0.018469 | 0.008763 | 0.76039 | 0.848445 | 0.76421 | 1.011384 |
| 0.018699 | 0.008602 | 0.77022 | 0.85196 | 0.7742 | 1.014822 |
| 0.018929 | 0.008763 | 0.78004 | 0.855 | 0.7842 | 1.018134 |
| 0.019159 | 0.009177 | 0.78988 | 0.858097 | 0.79419 | 1.021338 |
| 0.019389 | 0.009016 | 0.7997 | 0.860985 | 0.80419 | 1.025154 |
| 0.019619 | 0.009568 | 0.80953 | 0.86355 | 0.81418 | 1.027746 |
| 0.019826 | 0.009315 | 0.81935 | 0.865944 | 0.82417 | 1.029996 |
| 0.020056 | 0.009407 | 0.82918 | 0.868319 | 0.83417 | 1.032246 |
| 0.020286 | 0.009522 | 0.83901 | 0.870257 | 0.84416 | 1.033452 |
| 0.020516 | 0.009821 | 0.84884 | 0.872309 | 0.85416 | 1.03608 |
| 0.020746 | 0.009867 | 0.85866 | 0.873772 | 0.86415 | 1.03671 |
| 0.020976 | 0.01012 | 0.86849 | 0.875425 | 0.87415 | 1.039698 |
| 0.021183 | 0.010005 | 0.87832 | 0.877078 | 0.88415 | 1.040544 |
| 0.021413 | 0.01012 | 0.88815 | 0.877952 | 0.89413 | 1.040382 |
| 0.02162 | 0.010212 | 0.89797 | 0.879244 | 0.90412 | 1.041318 |
| 0.021873 | 0.010419 | 0.9078 | 0.880099 | 0.91412 | 1.041966 |
| 0.022103 | 0.010649 | 0.91763 | 0.881011 | 0.92411 | 1.042794 |
| 0.022333 | 0.01081 | 0.92746 | 0.881524 | 0.93411 | 1.042974 |
| 0.02254 | 0.010718 | 0.93729 | 0.881828 | 0.94411 | 1.042434 |
| 0.02277 | 0.010994 | 0.94711 | 0.882303 | 0.9541 | 1.042578 |
| 0.023 | 0.011316 | 0.95694 | 0.882417 | 0.9641 | 1.04211 |
| 0.023207 | 0.011316 | 0.96677 | 0.882455 | 0.97409 | 1.04184 |
| 0.02346 | 0.011362 | 0.9766 | 0.882493 | 0.98408 | 1.040814 |
| 0.02369 | 0.0115 | 0.98642 | 0.882056 | 0.99408 | 1.040598 |
| 0.023897 | 0.011753 | 0.99625 | 0.881809 | 1.00407 | 1.039338 |
| 0.024127 | 0.011592 | 1.00608 | 0.881524 | 1.01407 | 1.038312 |
| 0.024357 | 0.012098 | 1.01591 | 0.88103 | 1.02406 | 1.037232 |
| 0.024564 | 0.011845 | 1.02573 | 0.880118 | 1.03405 | 1.036512 |
| 0.024794 | 0.012075 | 1.03556 | 0.879909 | 1.04405 | 1.035144 |
| 0.025047 | 0.012443 | 1.04538 | 0.87875 | 1.05404 | 1.033614 |
| 0.025277 | 0.012512 | 1.05522 | 0.878142 | 1.06404 | 1.032894 |
| 0.025484 | 0.012374 | 1.06504 | 0.87704 | 1.07403 | 1.031058 |
| 0.025714 | 0.012581 | 1.07487 | 0.876128 | 1.08402 | 1.028538 |
| 0.025944 | 0.012903 | 1.0847 | 0.874266 | 1.09402 | 1.026918 |
| 0.026151 | 0.01288 | 1.09453 | 0.872898 | 1.10401 | 1.02618 |
| 0.026381 | 0.012949 | 1.10435 | 0.870979 | 1.114 | 1.023822 |
| 0.026611 | 0.013202 | 1.11418 | 0.869288 | 1.12401 | 1.022328 |
| 0.026841 | 0.013386 | 1.12401 | 0.867293 | 1.134 | 1.02078 |
| 0.027071 | 0.013639 | 1.13383 | 0.864576 | 1.14399 | 1.018926 |
| 0.027301 | 0.013501 | 1.14367 | 0.861802 | 1.15399 | 1.01709 |
| 0.027508 | 0.013409 | 1.15349 | 0.858116 | 1.16398 | 1.0143 |
| 0.027738 | 0.01357 | 1.16332 | 0.852511 | 1.17398 | 1.013598 |
| 0.027968 | 0.013938 |  |  | 1.18397 | 1.01088 |
| 0.028198 | 0.014007 |  |  | 1.19396 | 1.009242 |
| 0.028428 | 0.014007 |  |  | 1.20396 | 1.006452 |
| 0.028658 | 0.013984 |  |  | 1.21395 | 1.005048 |
| 0.028865 | 0.014582 |  |  | 1.22394 | 1.002924 |
| 0.029095 | 0.014651 |  |  | 1.23394 | 1.000404 |
| 0.029325 | 0.01449 |  |  | 1.24393 | 0.999036 |
| 0.029555 | 0.014628 |  |  | 1.25393 | 0.99702 |
| 0.029785 | 0.015065 |  |  | 1.26392 | 0.99423 |
| 0.030015 | 0.015364 |  |  | 1.27391 | 0.992304 |
| 0.030245 | 0.01518 |  |  | 1.28391 | 0.989406 |
| 0.030452 | 0.015111 |  |  | 1.2939 | 0.986958 |
| 0.030682 | 0.015594 |  |  | 1.3039 | 0.98523 |
| 0.030912 | 0.015617 |  |  | 1.31389 | 0.982566 |
| 0.031142 | 0.015893 |  |  | 1.32388 | 0.98001 |
| 0.031372 | 0.015663 |  |  | 1.33388 | 0.97578 |
| 0.031602 | 0.015962 |  |  | 1.34387 | 0.973692 |
| 0.031809 | 0.016353 |  |  | 1.35386 | 0.96615 |
| 0.032039 | 0.016192 |  |  |  |  |
| 0.032269 | 0.016261 |  |  |  |  |
| 0.032476 | 0.016353 |  |  |  |  |
| 0.032729 | 0.016698 |  |  |  |  |
| 0.032959 | 0.016744 |  |  |  |  |
| 0.033166 | 0.016813 |  |  |  |  |
| 0.033396 | 0.017066 |  |  |  |  |
| 0.033626 | 0.01725 |  |  |  |  |
| 0.033833 | 0.017434 |  |  |  |  |
| 0.034063 | 0.017365 |  |  |  |  |
| 0.034316 | 0.017434 |  |  |  |  |
| 0.034523 | 0.017687 |  |  |  |  |
| 0.034753 | 0.017917 |  |  |  |  |
| 0.034983 | 0.017894 |  |  |  |  |
| 0.03519 | 0.018285 |  |  |  |  |
| 0.03542 | 0.018262 |  |  |  |  |
| 0.03565 | 0.018653 |  |  |  |  |
| 0.03588 | 0.018745 |  |  |  |  |
| 0.03611 | 0.018561 |  |  |  |  |
| 0.03634 | 0.018561 |  |  |  |  |
| 0.03657 | 0.019021 |  |  |  |  |
| 0.036777 | 0.019067 |  |  |  |  |
| 0.037007 | 0.019435 |  |  |  |  |
| 0.037237 | 0.019228 |  |  |  |  |
| 0.037467 | 0.019596 |  |  |  |  |
| 0.037697 | 0.019458 |  |  |  |  |
| 0.037927 | 0.019734 |  |  |  |  |
| 0.038134 | 0.020056 |  |  |  |  |
| 0.038364 | 0.019849 |  |  |  |  |
| 0.038594 | 0.020332 |  |  |  |  |
| 0.038824 | 0.020125 |  |  |  |  |
| 0.039054 | 0.020309 |  |  |  |  |
| 0.039284 | 0.020608 |  |  |  |  |
| 0.039514 | 0.020723 |  |  |  |  |
| 0.039721 | 0.020907 |  |  |  |  |
| 0.039951 | 0.020792 |  |  |  |  |
| 0.040158 | 0.020999 |  |  |  |  |
| 0.040388 | 0.021183 |  |  |  |  |
| 0.040641 | 0.021574 |  |  |  |  |
| 0.040871 | 0.02162 |  |  |  |  |
| 0.041078 | 0.021528 |  |  |  |  |
| 0.041308 | 0.021597 |  |  |  |  |
| 0.041538 | 0.021965 |  |  |  |  |
| 0.041745 | 0.022057 |  |  |  |  |
| 0.041975 | 0.022011 |  |  |  |  |
| 0.042228 | 0.022448 |  |  |  |  |
| 0.042435 | 0.022563 |  |  |  |  |
| 0.042665 | 0.022701 |  |  |  |  |
| 0.042895 | 0.022586 |  |  |  |  |
| 0.043102 | 0.022793 |  |  |  |  |
| 0.043332 | 0.023069 |  |  |  |  |
| 0.043562 | 0.023368 |  |  |  |  |
| 0.043815 | 0.023345 |  |  |  |  |
| 0.044022 | 0.023368 |  |  |  |  |
| 0.044252 | 0.023253 |  |  |  |  |
| 0.044459 | 0.023598 |  |  |  |  |
| 0.044689 | 0.023644 |  |  |  |  |
| 0.044919 | 0.02392 |  |  |  |  |
| 0.045149 | 0.023989 |  |  |  |  |
| 0.045379 | 0.024311 |  |  |  |  |
| 0.045609 | 0.024472 |  |  |  |  |
| 0.045816 | 0.024357 |  |  |  |  |
| 0.046046 | 0.02461 |  |  |  |  |
| 0.046276 | 0.024725 |  |  |  |  |
| 0.046506 | 0.024863 |  |  |  |  |
| 0.046736 | 0.025116 |  |  |  |  |
| 0.046966 | 0.025047 |  |  |  |  |
| 0.047196 | 0.025415 |  |  |  |  |
| 0.047403 | 0.025369 |  |  |  |  |
| 0.047633 | 0.025369 |  |  |  |  |
| 0.047863 | 0.025691 |  |  |  |  |
| 0.04807 | 0.025875 |  |  |  |  |
| 0.048323 | 0.026289 |  |  |  |  |
| 0.048553 | 0.026404 |  |  |  |  |
| 0.04876 | 0.026197 |  |  |  |  |
| 0.04899 | 0.026542 |  |  |  |  |
| 0.04922 | 0.026588 |  |  |  |  |
| 0.04945 | 0.026611 |  |  |  |  |
| 0.049657 | 0.026772 |  |  |  |  |
| 0.04991 | 0.026749 |  |  |  |  |
| 0.05014 | 0.027347 |  |  |  |  |
| 0.050347 | 0.027255 |  |  |  |  |
| 0.050577 | 0.02737 |  |  |  |  |
| 0.050807 | 0.027508 |  |  |  |  |
| 0.051014 | 0.027715 |  |  |  |  |
| 0.051244 | 0.028014 |  |  |  |  |
| 0.051497 | 0.02783 |  |  |  |  |
| 0.051704 | 0.027945 |  |  |  |  |
| 0.051934 | 0.028359 |  |  |  |  |
| 0.052164 | 0.02852 |  |  |  |  |
| 0.052371 | 0.028543 |  |  |  |  |
| 0.052601 | 0.028635 |  |  |  |  |
| 0.052831 | 0.028681 |  |  |  |  |
| 0.053061 | 0.029256 |  |  |  |  |
| 0.053291 | 0.028865 |  |  |  |  |
| 0.053521 | 0.029164 |  |  |  |  |
| 0.053728 | 0.029141 |  |  |  |  |
| 0.053958 | 0.029371 |  |  |  |  |
| 0.054188 | 0.029693 |  |  |  |  |
| 0.054418 | 0.029624 |  |  |  |  |
| 0.054648 | 0.029808 |  |  |  |  |
| 0.054878 | 0.030061 |  |  |  |  |
| 0.055108 | 0.030153 |  |  |  |  |
| 0.055315 | 0.03036 |  |  |  |  |
| 0.055545 | 0.030383 |  |  |  |  |
| 0.055775 | 0.030728 |  |  |  |  |
| 0.056005 | 0.030843 |  |  |  |  |
| 0.056235 | 0.030866 |  |  |  |  |
| 0.056465 | 0.030981 |  |  |  |  |
| 0.056672 | 0.031257 |  |  |  |  |
| 0.056902 | 0.03151 |  |  |  |  |
| 0.057132 | 0.031671 |  |  |  |  |
| 0.057339 | 0.03174 |  |  |  |  |
| 0.057592 | 0.031694 |  |  |  |  |
| 0.057822 | 0.032016 |  |  |  |  |
| 0.058029 | 0.032062 |  |  |  |  |
| 0.058259 | 0.032361 |  |  |  |  |
| 0.058489 | 0.03243 |  |  |  |  |
| 0.058696 | 0.032568 |  |  |  |  |
| 0.058926 | 0.032568 |  |  |  |  |
| 0.059156 | 0.032798 |  |  |  |  |
| 0.059386 | 0.03289 |  |  |  |  |
| 0.059616 | 0.032982 |  |  |  |  |
| 0.059846 | 0.033419 |  |  |  |  |
| 0.060076 | 0.033281 |  |  |  |  |
| 0.060283 | 0.033396 |  |  |  |  |
| 0.060513 | 0.033603 |  |  |  |  |
| 0.060743 | 0.033902 |  |  |  |  |
| 0.060973 | 0.034063 |  |  |  |  |
| 0.061203 | 0.033718 |  |  |  |  |
| 0.061433 | 0.034224 |  |  |  |  |
| 0.06164 | 0.034132 |  |  |  |  |
| 0.06187 | 0.034546 |  |  |  |  |
| 0.0621 | 0.034523 |  |  |  |  |
| 0.06233 | 0.034638 |  |  |  |  |
| 0.06256 | 0.034799 |  |  |  |  |
| 0.06279 | 0.035098 |  |  |  |  |
| 0.06302 | 0.035213 |  |  |  |  |
| 0.063227 | 0.035144 |  |  |  |  |
| 0.063457 | 0.035489 |  |  |  |  |
| 0.063664 | 0.035673 |  |  |  |  |
| 0.063917 | 0.035765 |  |  |  |  |
| 0.064147 | 0.035903 |  |  |  |  |
| 0.064354 | 0.035972 |  |  |  |  |
| 0.064584 | 0.036202 |  |  |  |  |
| 0.064814 | 0.036478 |  |  |  |  |
| 0.065044 | 0.036616 |  |  |  |  |
| 0.065251 | 0.0368 |  |  |  |  |
| 0.065504 | 0.036731 |  |  |  |  |
| 0.065734 | 0.0368 |  |  |  |  |
| 0.065941 | 0.037053 |  |  |  |  |
| 0.066171 | 0.037237 |  |  |  |  |
| 0.066401 | 0.037237 |  |  |  |  |
| 0.066608 | 0.037789 |  |  |  |  |
| 0.066838 | 0.037559 |  |  |  |  |
| 0.067091 | 0.03772 |  |  |  |  |
| 0.067298 | 0.037766 |  |  |  |  |
| 0.067528 | 0.038203 |  |  |  |  |
| 0.067758 | 0.038226 |  |  |  |  |
| 0.067988 | 0.038157 |  |  |  |  |
| 0.068195 | 0.038456 |  |  |  |  |
| 0.068425 | 0.038709 |  |  |  |  |
| 0.068678 | 0.038847 |  |  |  |  |
| 0.068885 | 0.039054 |  |  |  |  |
| 0.069115 | 0.039077 |  |  |  |  |
| 0.069322 | 0.039169 |  |  |  |  |
| 0.069552 | 0.039399 |  |  |  |  |
| 0.069782 | 0.039675 |  |  |  |  |
| 0.070012 | 0.03956 |  |  |  |  |
| 0.070242 | 0.03979 |  |  |  |  |
| 0.070472 | 0.040112 |  |  |  |  |
| 0.070702 | 0.040158 |  |  |  |  |
| 0.070909 | 0.040181 |  |  |  |  |
| 0.071139 | 0.040296 |  |  |  |  |
| 0.071369 | 0.04071 |  |  |  |  |
| 0.071599 | 0.040802 |  |  |  |  |
| 0.071829 | 0.040825 |  |  |  |  |
| 0.072059 | 0.040848 |  |  |  |  |
| 0.072266 | 0.041469 |  |  |  |  |
| 0.072496 | 0.041331 |  |  |  |  |
| 0.072726 | 0.041561 |  |  |  |  |
| 0.072933 | 0.041446 |  |  |  |  |
| 0.073186 | 0.041607 |  |  |  |  |
| 0.073416 | 0.042182 |  |  |  |  |
| 0.073646 | 0.042044 |  |  |  |  |
| 0.073853 | 0.042182 |  |  |  |  |
| 0.074083 | 0.042021 |  |  |  |  |
| 0.074313 | 0.04255 |  |  |  |  |
| 0.07452 | 0.042826 |  |  |  |  |
| 0.074773 | 0.042734 |  |  |  |  |
| 0.075003 | 0.042941 |  |  |  |  |
| 0.07521 | 0.043263 |  |  |  |  |
| 0.07544 | 0.043125 |  |  |  |  |
| 0.07567 | 0.043424 |  |  |  |  |
| 0.075877 | 0.043401 |  |  |  |  |
| 0.076107 | 0.043585 |  |  |  |  |
| 0.07636 | 0.043838 |  |  |  |  |
| 0.076567 | 0.043953 |  |  |  |  |
| 0.076797 | 0.044045 |  |  |  |  |
| 0.077027 | 0.044091 |  |  |  |  |
| 0.077234 | 0.044482 |  |  |  |  |
| 0.077464 | 0.044735 |  |  |  |  |
| 0.077694 | 0.044735 |  |  |  |  |
| 0.077924 | 0.044735 |  |  |  |  |
| 0.078154 | 0.044965 |  |  |  |  |
| 0.078384 | 0.045126 |  |  |  |  |
| 0.078614 | 0.045333 |  |  |  |  |
| 0.078821 | 0.04531 |  |  |  |  |
| 0.079051 | 0.045724 |  |  |  |  |
| 0.079281 | 0.045885 |  |  |  |  |
| 0.079511 | 0.046 |  |  |  |  |
| 0.079741 | 0.045609 |  |  |  |  |
| 0.079971 | 0.046069 |  |  |  |  |
| 0.080178 | 0.046437 |  |  |  |  |
| 0.080408 | 0.046506 |  |  |  |  |
| 0.080638 | 0.046437 |  |  |  |  |
| 0.080868 | 0.046667 |  |  |  |  |
| 0.081098 | 0.047012 |  |  |  |  |
| 0.081328 | 0.047288 |  |  |  |  |
| 0.081558 | 0.047035 |  |  |  |  |
| 0.081765 | 0.047587 |  |  |  |  |
| 0.081995 | 0.047541 |  |  |  |  |
| 0.082202 | 0.047633 |  |  |  |  |
| 0.082432 | 0.047748 |  |  |  |  |
| 0.082685 | 0.04784 |  |  |  |  |
| 0.082892 | 0.048208 |  |  |  |  |
| 0.083122 | 0.048231 |  |  |  |  |
| 0.083352 | 0.0483 |  |  |  |  |
| 0.083559 | 0.048622 |  |  |  |  |
| 0.083789 | 0.048484 |  |  |  |  |
| 0.084019 | 0.049013 |  |  |  |  |
| 0.084272 | 0.048944 |  |  |  |  |
| 0.084479 | 0.049289 |  |  |  |  |
| 0.084709 | 0.049358 |  |  |  |  |
| 0.084939 | 0.049473 |  |  |  |  |
| 0.085146 | 0.049565 |  |  |  |  |
| 0.085376 | 0.049611 |  |  |  |  |
| 0.085606 | 0.049887 |  |  |  |  |
| 0.085836 | 0.050232 |  |  |  |  |
| 0.086066 | 0.050462 |  |  |  |  |
| 0.086296 | 0.050439 |  |  |  |  |
| 0.086503 | 0.050416 |  |  |  |  |
| 0.086733 | 0.050623 |  |  |  |  |
| 0.086963 | 0.051014 |  |  |  |  |
| 0.087193 | 0.051175 |  |  |  |  |
| 0.087423 | 0.051083 |  |  |  |  |
| 0.087653 | 0.051037 |  |  |  |  |
| 0.087883 | 0.051497 |  |  |  |  |
| 0.08809 | 0.051681 |  |  |  |  |
| 0.08832 | 0.051681 |  |  |  |  |
| 0.088527 | 0.051911 |  |  |  |  |
| 0.08878 | 0.052072 |  |  |  |  |
| 0.08901 | 0.052118 |  |  |  |  |
| 0.08924 | 0.05244 |  |  |  |  |
| 0.089447 | 0.052279 |  |  |  |  |
| 0.089677 | 0.052578 |  |  |  |  |
| 0.089907 | 0.052808 |  |  |  |  |
| 0.090114 | 0.052992 |  |  |  |  |
| 0.090367 | 0.052877 |  |  |  |  |
| 0.090597 | 0.053291 |  |  |  |  |
| 0.090804 | 0.053544 |  |  |  |  |
| 0.091034 | 0.053406 |  |  |  |  |
| 0.091264 | 0.053774 |  |  |  |  |
| 0.091471 | 0.053866 |  |  |  |  |
| 0.091701 | 0.054234 |  |  |  |  |
| 0.091954 | 0.05382 |  |  |  |  |
| 0.092184 | 0.054188 |  |  |  |  |
| 0.092391 | 0.05428 |  |  |  |  |
| 0.092621 | 0.054694 |  |  |  |  |
| 0.092828 | 0.055062 |  |  |  |  |
| 0.093058 | 0.055016 |  |  |  |  |
| 0.093288 | 0.055016 |  |  |  |  |
| 0.093541 | 0.055269 |  |  |  |  |
| 0.093748 | 0.055545 |  |  |  |  |
| 0.093978 | 0.05543 |  |  |  |  |
| 0.094208 | 0.055453 |  |  |  |  |
| 0.094415 | 0.055637 |  |  |  |  |
| 0.094645 | 0.055982 |  |  |  |  |
| 0.094875 | 0.056028 |  |  |  |  |
| 0.095105 | 0.056304 |  |  |  |  |
| 0.095335 | 0.056097 |  |  |  |  |
| 0.095565 | 0.056787 |  |  |  |  |
| 0.095772 | 0.056902 |  |  |  |  |
| 0.096002 | 0.056787 |  |  |  |  |
| 0.096232 | 0.057017 |  |  |  |  |
| 0.096462 | 0.057132 |  |  |  |  |
| 0.096692 | 0.057385 |  |  |  |  |
| 0.096922 | 0.057431 |  |  |  |  |
| 0.097129 | 0.057638 |  |  |  |  |
| 0.097359 | 0.057822 |  |  |  |  |
| 0.097589 | 0.058052 |  |  |  |  |
| 0.097819 | 0.058121 |  |  |  |  |
| 0.098049 | 0.058259 |  |  |  |  |
| 0.098279 | 0.058374 |  |  |  |  |
| 0.098509 | 0.058696 |  |  |  |  |
| 0.098716 | 0.05888 |  |  |  |  |
| 0.098946 | 0.058673 |  |  |  |  |
| 0.099153 | 0.05888 |  |  |  |  |
| 0.099383 | 0.059294 |  |  |  |  |
| 0.099636 | 0.059524 |  |  |  |  |
| 0.099866 | 0.059685 |  |  |  |  |
| 0.100073 | 0.059455 |  |  |  |  |
| 0.100303 | 0.059662 |  |  |  |  |
| 0.100533 | 0.060007 |  |  |  |  |
| 0.10074 | 0.060214 |  |  |  |  |
| 0.10097 | 0.060122 |  |  |  |  |
| 0.1012 | 0.06026 |  |  |  |  |
| 0.10143 | 0.060789 |  |  |  |  |
| 0.10166 | 0.060973 |  |  |  |  |
| 0.10189 | 0.060697 |  |  |  |  |
| 0.102097 | 0.060858 |  |  |  |  |
| 0.102327 | 0.061318 |  |  |  |  |
| 0.102557 | 0.061364 |  |  |  |  |
| 0.102787 | 0.061479 |  |  |  |  |
| 0.103017 | 0.061548 |  |  |  |  |
| 0.103247 | 0.061755 |  |  |  |  |
| 0.103477 | 0.061939 |  |  |  |  |
| 0.103684 | 0.061801 |  |  |  |  |
| 0.103914 | 0.062261 |  |  |  |  |
| 0.104144 | 0.062468 |  |  |  |  |
| 0.104374 | 0.06256 |  |  |  |  |
| 0.104604 | 0.062767 |  |  |  |  |
| 0.104834 | 0.062836 |  |  |  |  |
| 0.105041 | 0.062859 |  |  |  |  |
| 0.105271 | 0.063204 |  |  |  |  |
| 0.105501 | 0.063365 |  |  |  |  |
| 0.105731 | 0.063181 |  |  |  |  |
| 0.105961 | 0.063273 |  |  |  |  |
| 0.106191 | 0.063917 |  |  |  |  |
| 0.106398 | 0.06371 |  |  |  |  |
| 0.106628 | 0.064147 |  |  |  |  |
| 0.106858 | 0.064124 |  |  |  |  |
| 0.107065 | 0.064377 |  |  |  |  |
| 0.107295 | 0.06463 |  |  |  |  |
| 0.107548 | 0.064538 |  |  |  |  |
| 0.107755 | 0.064492 |  |  |  |  |
| 0.107985 | 0.064837 |  |  |  |  |
| 0.108215 | 0.065297 |  |  |  |  |
| 0.108445 | 0.06555 |  |  |  |  |
| 0.108652 | 0.065251 |  |  |  |  |
| 0.108882 | 0.065711 |  |  |  |  |
| 0.109135 | 0.065826 |  |  |  |  |
| 0.109342 | 0.066102 |  |  |  |  |
| 0.109572 | 0.065987 |  |  |  |  |
| 0.109802 | 0.06624 |  |  |  |  |
| 0.110009 | 0.066355 |  |  |  |  |
| 0.110239 | 0.0667 |  |  |  |  |
| 0.110469 | 0.066654 |  |  |  |  |
| 0.110699 | 0.0667 |  |  |  |  |
| 0.110929 | 0.066953 |  |  |  |  |
| 0.111159 | 0.067137 |  |  |  |  |
| 0.111389 | 0.067321 |  |  |  |  |
| 0.111596 | 0.067298 |  |  |  |  |
| 0.111826 | 0.067689 |  |  |  |  |
| 0.112056 | 0.068057 |  |  |  |  |
| 0.112286 | 0.067919 |  |  |  |  |
| 0.112516 | 0.068149 |  |  |  |  |
| 0.112723 | 0.067988 |  |  |  |  |
| 0.112953 | 0.068517 |  |  |  |  |
| 0.113183 | 0.068816 |  |  |  |  |
| 0.113413 | 0.06877 |  |  |  |  |
| 0.113643 | 0.068839 |  |  |  |  |
| 0.113873 | 0.069161 |  |  |  |  |
| 0.114103 | 0.06923 |  |  |  |  |
| 0.11431 | 0.069345 |  |  |  |  |
| 0.11454 | 0.069414 |  |  |  |  |
| 0.11477 | 0.069437 |  |  |  |  |
| 0.114977 | 0.069897 |  |  |  |  |
| 0.11523 | 0.070012 |  |  |  |  |
| 0.11546 | 0.070127 |  |  |  |  |
| 0.115667 | 0.07015 |  |  |  |  |
| 0.115897 | 0.070495 |  |  |  |  |
| 0.116127 | 0.070449 |  |  |  |  |
| 0.116357 | 0.070771 |  |  |  |  |
| 0.116564 | 0.070771 |  |  |  |  |
| 0.116817 | 0.071208 |  |  |  |  |
| 0.117024 | 0.071415 |  |  |  |  |
| 0.117254 | 0.071208 |  |  |  |  |
| 0.117484 | 0.071277 |  |  |  |  |
| 0.117714 | 0.071829 |  |  |  |  |
| 0.117921 | 0.071967 |  |  |  |  |
| 0.118151 | 0.072174 |  |  |  |  |
| 0.118381 | 0.072243 |  |  |  |  |
| 0.118611 | 0.072243 |  |  |  |  |
| 0.118841 | 0.072473 |  |  |  |  |
| 0.119071 | 0.072519 |  |  |  |  |
| 0.119278 | 0.072634 |  |  |  |  |
| 0.119508 | 0.072841 |  |  |  |  |
| 0.119738 | 0.073416 |  |  |  |  |
| 0.119968 | 0.073301 |  |  |  |  |
| 0.120198 | 0.073301 |  |  |  |  |
| 0.120428 | 0.073485 |  |  |  |  |
| 0.120635 | 0.073738 |  |  |  |  |
| 0.120865 | 0.074129 |  |  |  |  |
| 0.121095 | 0.073945 |  |  |  |  |
| 0.121325 | 0.073991 |  |  |  |  |
| 0.121555 | 0.074474 |  |  |  |  |
| 0.121785 | 0.074773 |  |  |  |  |
| 0.122015 | 0.074865 |  |  |  |  |
| 0.122222 | 0.074957 |  |  |  |  |
| 0.122452 | 0.075003 |  |  |  |  |
| 0.122682 | 0.075394 |  |  |  |  |
| 0.122912 | 0.075256 |  |  |  |  |
| 0.123142 | 0.07567 |  |  |  |  |
| 0.123349 | 0.075808 |  |  |  |  |
| 0.123579 | 0.075992 |  |  |  |  |
| 0.123809 | 0.076015 |  |  |  |  |
| 0.124039 | 0.07613 |  |  |  |  |
| 0.124246 | 0.076061 |  |  |  |  |
| 0.124476 | 0.076751 |  |  |  |  |
| 0.124729 | 0.076889 |  |  |  |  |
| 0.124936 | 0.076728 |  |  |  |  |
| 0.125166 | 0.077096 |  |  |  |  |
| 0.125396 | 0.076981 |  |  |  |  |
| 0.125603 | 0.077257 |  |  |  |  |
| 0.125833 | 0.07751 |  |  |  |  |
| 0.126063 | 0.077395 |  |  |  |  |
| 0.126293 | 0.077832 |  |  |  |  |
| 0.126523 | 0.0782 |  |  |  |  |
| 0.126753 | 0.078154 |  |  |  |  |
| 0.126983 | 0.078062 |  |  |  |  |
| 0.12719 | 0.077901 |  |  |  |  |
| 0.12742 | 0.078752 |  |  |  |  |
| 0.12765 | 0.078775 |  |  |  |  |
| 0.12788 | 0.078936 |  |  |  |  |
| 0.12811 | 0.078936 |  |  |  |  |
| 0.12834 | 0.079327 |  |  |  |  |
| 0.128547 | 0.079488 |  |  |  |  |
| 0.128777 | 0.079672 |  |  |  |  |
| 0.129007 | 0.079626 |  |  |  |  |
| 0.129237 | 0.079856 |  |  |  |  |
| 0.129467 | 0.079879 |  |  |  |  |
| 0.129697 | 0.079994 |  |  |  |  |
| 0.129904 | 0.080339 |  |  |  |  |
| 0.130134 | 0.080201 |  |  |  |  |
| 0.130364 | 0.080661 |  |  |  |  |
| 0.130571 | 0.080868 |  |  |  |  |
| 0.130824 | 0.080868 |  |  |  |  |
| 0.131054 | 0.081006 |  |  |  |  |
| 0.131261 | 0.081167 |  |  |  |  |
| 0.131491 | 0.081443 |  |  |  |  |
| 0.131721 | 0.081489 |  |  |  |  |
| 0.131928 | 0.081466 |  |  |  |  |
| 0.132158 | 0.082064 |  |  |  |  |
| 0.132411 | 0.08211 |  |  |  |  |
| 0.132641 | 0.082087 |  |  |  |  |
| 0.132848 | 0.082202 |  |  |  |  |
| 0.133078 | 0.082432 |  |  |  |  |
| 0.133308 | 0.08257 |  |  |  |  |
| 0.133515 | 0.083168 |  |  |  |  |
| 0.133745 | 0.082984 |  |  |  |  |
| 0.133998 | 0.083237 |  |  |  |  |
| 0.134205 | 0.083398 |  |  |  |  |
| 0.134435 | 0.083513 |  |  |  |  |
| 0.134665 | 0.083214 |  |  |  |  |
| 0.134872 | 0.083628 |  |  |  |  |
| 0.135102 | 0.083927 |  |  |  |  |
| 0.135332 | 0.084318 |  |  |  |  |
| 0.135562 | 0.08418 |  |  |  |  |
| 0.135792 | 0.084318 |  |  |  |  |
| 0.136022 | 0.084433 |  |  |  |  |
| 0.136229 | 0.084778 |  |  |  |  |
| 0.136459 | 0.084824 |  |  |  |  |
| 0.136689 | 0.08464 |  |  |  |  |
| 0.136919 | 0.085031 |  |  |  |  |
| 0.137149 | 0.08533 |  |  |  |  |
| 0.137379 | 0.085399 |  |  |  |  |
| 0.137609 | 0.085583 |  |  |  |  |
| 0.137816 | 0.085675 |  |  |  |  |
| 0.138046 | 0.086112 |  |  |  |  |
| 0.138276 | 0.085951 |  |  |  |  |
| 0.138506 | 0.086158 |  |  |  |  |
| 0.138736 | 0.086411 |  |  |  |  |
| 0.138966 | 0.086526 |  |  |  |  |
| 0.139173 | 0.086894 |  |  |  |  |
| 0.139403 | 0.086894 |  |  |  |  |
| 0.139633 | 0.086917 |  |  |  |  |
| 0.13984 | 0.087216 |  |  |  |  |
| 0.140093 | 0.08763 |  |  |  |  |
| 0.140323 | 0.087423 |  |  |  |  |
| 0.140553 | 0.087377 |  |  |  |  |
| 0.14076 | 0.087446 |  |  |  |  |
| 0.14099 | 0.087929 |  |  |  |  |
| 0.14122 | 0.087975 |  |  |  |  |
| 0.141427 | 0.088435 |  |  |  |  |
| 0.14168 | 0.088389 |  |  |  |  |
| 0.14191 | 0.08878 |  |  |  |  |
| 0.142117 | 0.088826 |  |  |  |  |
| 0.142347 | 0.08878 |  |  |  |  |
| 0.142577 | 0.088872 |  |  |  |  |
| 0.142784 | 0.08924 |  |  |  |  |
| 0.143014 | 0.08947 |  |  |  |  |
| 0.143244 | 0.089631 |  |  |  |  |
| 0.143474 | 0.089539 |  |  |  |  |
| 0.143704 | 0.0897 |  |  |  |  |
| 0.143934 | 0.089999 |  |  |  |  |
| 0.144141 | 0.090229 |  |  |  |  |
| 0.144371 | 0.090413 |  |  |  |  |
| 0.144601 | 0.090367 |  |  |  |  |
| 0.144831 | 0.090597 |  |  |  |  |
| 0.145061 | 0.090942 |  |  |  |  |
| 0.145291 | 0.091126 |  |  |  |  |
| 0.145498 | 0.091172 |  |  |  |  |
| 0.145728 | 0.091264 |  |  |  |  |
| 0.145958 | 0.091678 |  |  |  |  |
| 0.146188 | 0.091701 |  |  |  |  |
| 0.146418 | 0.091954 |  |  |  |  |
| 0.146648 | 0.091977 |  |  |  |  |
| 0.146878 | 0.092161 |  |  |  |  |
| 0.147085 | 0.092115 |  |  |  |  |
| 0.147315 | 0.092437 |  |  |  |  |
| 0.147545 | 0.092644 |  |  |  |  |
| 0.147775 | 0.093127 |  |  |  |  |
| 0.148005 | 0.093081 |  |  |  |  |
| 0.148235 | 0.093035 |  |  |  |  |
| 0.148442 | 0.09315 |  |  |  |  |
| 0.148672 | 0.093449 |  |  |  |  |
| 0.148902 | 0.093564 |  |  |  |  |
| 0.149109 | 0.093748 |  |  |  |  |
| 0.149339 | 0.094093 |  |  |  |  |
| 0.149592 | 0.093794 |  |  |  |  |
| 0.149799 | 0.094254 |  |  |  |  |
| 0.150029 | 0.094277 |  |  |  |  |
| 0.150259 | 0.094507 |  |  |  |  |
| 0.150466 | 0.094461 |  |  |  |  |
| 0.150696 | 0.095013 |  |  |  |  |
| 0.150926 | 0.094783 |  |  |  |  |
| 0.151179 | 0.095036 |  |  |  |  |
| 0.151386 | 0.095174 |  |  |  |  |
| 0.151616 | 0.095404 |  |  |  |  |
| 0.151846 | 0.095887 |  |  |  |  |
| 0.152053 | 0.095956 |  |  |  |  |
| 0.152283 | 0.096002 |  |  |  |  |
| 0.152513 | 0.096278 |  |  |  |  |
| 0.152743 | 0.096508 |  |  |  |  |
| 0.152973 | 0.096462 |  |  |  |  |
| 0.153203 | 0.096485 |  |  |  |  |
| 0.15341 | 0.096232 |  |  |  |  |
| 0.15364 | 0.097106 |  |  |  |  |
| 0.15387 | 0.097106 |  |  |  |  |
| 0.1541 | 0.097152 |  |  |  |  |
| 0.15433 | 0.097129 |  |  |  |  |
| 0.15456 | 0.097451 |  |  |  |  |
| 0.154767 | 0.09775 |  |  |  |  |
| 0.154997 | 0.097934 |  |  |  |  |
| 0.155227 | 0.097796 |  |  |  |  |
| 0.155434 | 0.098072 |  |  |  |  |
| 0.155687 | 0.09844 |  |  |  |  |
| 0.155917 | 0.098532 |  |  |  |  |
| 0.156124 | 0.098739 |  |  |  |  |
| 0.156354 | 0.098923 |  |  |  |  |
| 0.156584 | 0.099268 |  |  |  |  |
| 0.156814 | 0.099107 |  |  |  |  |
| 0.157021 | 0.099222 |  |  |  |  |
| 0.157274 | 0.099268 |  |  |  |  |
| 0.157504 | 0.099567 |  |  |  |  |
| 0.157711 | 0.099866 |  |  |  |  |
| 0.157941 | 0.09982 |  |  |  |  |
| 0.158171 | 0.099958 |  |  |  |  |
| 0.158378 | 0.100096 |  |  |  |  |
| 0.158608 | 0.100487 |  |  |  |  |
| 0.158861 | 0.100395 |  |  |  |  |
| 0.159068 | 0.100556 |  |  |  |  |
| 0.159298 | 0.10074 |  |  |  |  |
| 0.159528 | 0.101131 |  |  |  |  |
| 0.159758 | 0.101154 |  |  |  |  |
| 0.159965 | 0.101246 |  |  |  |  |
| 0.160195 | 0.101522 |  |  |  |  |
| 0.160425 | 0.101752 |  |  |  |  |
| 0.160655 | 0.101982 |  |  |  |  |
| 0.160885 | 0.101683 |  |  |  |  |
| 0.161092 | 0.101936 |  |  |  |  |
| 0.161322 | 0.102672 |  |  |  |  |
| 0.161552 | 0.102534 |  |  |  |  |
| 0.161782 | 0.102327 |  |  |  |  |
| 0.162012 | 0.102741 |  |  |  |  |
| 0.162242 | 0.10281 |  |  |  |  |
| 0.162472 | 0.103109 |  |  |  |  |
| 0.162679 | 0.103155 |  |  |  |  |
| 0.162909 | 0.10327 |  |  |  |  |
| 0.163139 | 0.103477 |  |  |  |  |
| 0.163369 | 0.103891 |  |  |  |  |
| 0.163599 | 0.103983 |  |  |  |  |
| 0.163829 | 0.103983 |  |  |  |  |
| 0.164036 | 0.10442 |  |  |  |  |
| 0.164266 | 0.10419 |  |  |  |  |
| 0.164496 | 0.104374 |  |  |  |  |
| 0.164726 | 0.10465 |  |  |  |  |
| 0.164956 | 0.104788 |  |  |  |  |
| 0.165186 | 0.10511 |  |  |  |  |
| 0.165416 | 0.105133 |  |  |  |  |
| 0.165623 | 0.105156 |  |  |  |  |
| 0.165853 | 0.104995 |  |  |  |  |
| 0.166083 | 0.105639 |  |  |  |  |
| 0.16629 | 0.105823 |  |  |  |  |
| 0.16652 | 0.105938 |  |  |  |  |
| 0.16675 | 0.105892 |  |  |  |  |
| 0.16698 | 0.106168 |  |  |  |  |
| 0.16721 | 0.106444 |  |  |  |  |
| 0.16744 | 0.106421 |  |  |  |  |
| 0.167647 | 0.106789 |  |  |  |  |
| 0.167877 | 0.10649 |  |  |  |  |
| 0.168107 | 0.106996 |  |  |  |  |
| 0.168337 | 0.107364 |  |  |  |  |
| 0.168567 | 0.107249 |  |  |  |  |
| 0.168797 | 0.107479 |  |  |  |  |
| 0.169004 | 0.107525 |  |  |  |  |
| 0.169234 | 0.108123 |  |  |  |  |
| 0.169464 | 0.108031 |  |  |  |  |
| 0.169694 | 0.107916 |  |  |  |  |
| 0.169924 | 0.108169 |  |  |  |  |
| 0.170154 | 0.108606 |  |  |  |  |
| 0.170384 | 0.108583 |  |  |  |  |
| 0.170591 | 0.108744 |  |  |  |  |
| 0.170821 | 0.108813 |  |  |  |  |
| 0.171051 | 0.10948 |  |  |  |  |
| 0.171281 | 0.109319 |  |  |  |  |
| 0.171511 | 0.109641 |  |  |  |  |
| 0.171741 | 0.109664 |  |  |  |  |
| 0.171948 | 0.109917 |  |  |  |  |
| 0.172178 | 0.110124 |  |  |  |  |
| 0.172408 | 0.110032 |  |  |  |  |
| 0.172615 | 0.110331 |  |  |  |  |
| 0.172868 | 0.110331 |  |  |  |  |
| 0.173098 | 0.110676 |  |  |  |  |
| 0.173305 | 0.111021 |  |  |  |  |
| 0.173535 | 0.110837 |  |  |  |  |
| 0.173765 | 0.11109 |  |  |  |  |
| 0.173972 | 0.111504 |  |  |  |  |
| 0.174202 | 0.111504 |  |  |  |  |
| 0.174455 | 0.111596 |  |  |  |  |
| 0.174662 | 0.111573 |  |  |  |  |
| 0.174892 | 0.112125 |  |  |  |  |
| 0.175122 | 0.11201 |  |  |  |  |
| 0.175352 | 0.112056 |  |  |  |  |
| 0.175559 | 0.112217 |  |  |  |  |
| 0.175789 | 0.112608 |  |  |  |  |
| 0.176042 | 0.112585 |  |  |  |  |
| 0.176249 | 0.112838 |  |  |  |  |
| 0.176479 | 0.112884 |  |  |  |  |
| 0.176709 | 0.113183 |  |  |  |  |
| 0.176916 | 0.113712 |  |  |  |  |
| 0.177146 | 0.11339 |  |  |  |  |
| 0.177376 | 0.113597 |  |  |  |  |
| 0.177606 | 0.113666 |  |  |  |  |
| 0.177836 | 0.11408 |  |  |  |  |
| 0.178066 | 0.113988 |  |  |  |  |
| 0.178273 | 0.114126 |  |  |  |  |
| 0.178503 | 0.114425 |  |  |  |  |
| 0.178733 | 0.114724 |  |  |  |  |
| 0.178963 | 0.114839 |  |  |  |  |
| 0.179193 | 0.114908 |  |  |  |  |
| 0.179423 | 0.114885 |  |  |  |  |
| 0.17963 | 0.115322 |  |  |  |  |
| 0.17986 | 0.115437 |  |  |  |  |
| 0.18009 | 0.115437 |  |  |  |  |
| 0.180297 | 0.115621 |  |  |  |  |
| 0.18055 | 0.115874 |  |  |  |  |
| 0.18078 | 0.116081 |  |  |  |  |
| 0.18101 | 0.116219 |  |  |  |  |
| 0.181217 | 0.11615 |  |  |  |  |
| 0.181447 | 0.11638 |  |  |  |  |
| 0.181677 | 0.116794 |  |  |  |  |
| 0.181884 | 0.116748 |  |  |  |  |
| 0.182137 | 0.1173 |  |  |  |  |
| 0.182367 | 0.116886 |  |  |  |  |
| 0.182574 | 0.1173 |  |  |  |  |
| 0.182804 | 0.117576 |  |  |  |  |
| 0.183034 | 0.117645 |  |  |  |  |
| 0.183241 | 0.117691 |  |  |  |  |
| 0.183471 | 0.117898 |  |  |  |  |
| 0.183724 | 0.118151 |  |  |  |  |
| 0.183954 | 0.118197 |  |  |  |  |
| 0.184161 | 0.118036 |  |  |  |  |
| 0.184391 | 0.118496 |  |  |  |  |
| 0.184598 | 0.118818 |  |  |  |  |
| 0.184828 | 0.118979 |  |  |  |  |
| 0.185058 | 0.118979 |  |  |  |  |
| 0.185288 | 0.119232 |  |  |  |  |
| 0.185518 | 0.11937 |  |  |  |  |
| 0.185748 | 0.120014 |  |  |  |  |
| 0.185978 | 0.119761 |  |  |  |  |
| 0.186185 | 0.119968 |  |  |  |  |
| 0.186415 | 0.120152 |  |  |  |  |
| 0.186645 | 0.120405 |  |  |  |  |
| 0.186875 | 0.120497 |  |  |  |  |
| 0.187082 | 0.120336 |  |  |  |  |
| 0.187312 | 0.120635 |  |  |  |  |
| 0.187542 | 0.121026 |  |  |  |  |
| 0.187772 | 0.120888 |  |  |  |  |
| 0.187979 | 0.121072 |  |  |  |  |
| 0.188232 | 0.121072 |  |  |  |  |
| 0.188462 | 0.121532 |  |  |  |  |
| 0.188669 | 0.121647 |  |  |  |  |
| 0.188899 | 0.121693 |  |  |  |  |
| 0.189129 | 0.121877 |  |  |  |  |
| 0.189336 | 0.122199 |  |  |  |  |
| 0.189566 | 0.122383 |  |  |  |  |
| 0.189819 | 0.122521 |  |  |  |  |
| 0.190026 | 0.122337 |  |  |  |  |
| 0.190256 | 0.122682 |  |  |  |  |
| 0.190486 | 0.122958 |  |  |  |  |
| 0.190693 | 0.123142 |  |  |  |  |
| 0.190923 | 0.123188 |  |  |  |  |
| 0.191153 | 0.123349 |  |  |  |  |
| 0.191383 | 0.12374 |  |  |  |  |
| 0.191613 | 0.123556 |  |  |  |  |
| 0.191843 | 0.123763 |  |  |  |  |
| 0.19205 | 0.124016 |  |  |  |  |
| 0.19228 | 0.124177 |  |  |  |  |
| 0.19251 | 0.124338 |  |  |  |  |
| 0.19274 | 0.124522 |  |  |  |  |
| 0.19297 | 0.124407 |  |  |  |  |
| 0.1932 | 0.124545 |  |  |  |  |
| 0.19343 | 0.124982 |  |  |  |  |
| 0.193637 | 0.125166 |  |  |  |  |
| 0.193867 | 0.12512 |  |  |  |  |
| 0.194097 | 0.125672 |  |  |  |  |
| 0.194327 | 0.125695 |  |  |  |  |
| 0.194557 | 0.125672 |  |  |  |  |
| 0.194787 | 0.125764 |  |  |  |  |
| 0.194994 | 0.126063 |  |  |  |  |
| 0.195224 | 0.126247 |  |  |  |  |
| 0.195454 | 0.126293 |  |  |  |  |
| 0.195661 | 0.126408 |  |  |  |  |
| 0.195891 | 0.126477 |  |  |  |  |
| 0.196144 | 0.126845 |  |  |  |  |
| 0.196351 | 0.126799 |  |  |  |  |
| 0.196581 | 0.127029 |  |  |  |  |
| 0.196811 | 0.127121 |  |  |  |  |
| 0.197018 | 0.127512 |  |  |  |  |
| 0.197248 | 0.127788 |  |  |  |  |
| 0.197478 | 0.127903 |  |  |  |  |
| 0.197708 | 0.128041 |  |  |  |  |
| 0.197938 | 0.127926 |  |  |  |  |
| 0.198168 | 0.128386 |  |  |  |  |
| 0.198398 | 0.128478 |  |  |  |  |
| 0.198605 | 0.128386 |  |  |  |  |
| 0.198835 | 0.128616 |  |  |  |  |
| 0.199065 | 0.129053 |  |  |  |  |
| 0.199295 | 0.128961 |  |  |  |  |
| 0.199525 | 0.12903 |  |  |  |  |
| 0.199755 | 0.129007 |  |  |  |  |
| 0.199962 | 0.129605 |  |  |  |  |
| 0.200192 | 0.129651 |  |  |  |  |
| 0.200422 | 0.129812 |  |  |  |  |
| 0.200652 | 0.129789 |  |  |  |  |
| 0.200882 | 0.130019 |  |  |  |  |
| 0.201112 | 0.130479 |  |  |  |  |
| 0.201342 | 0.130525 |  |  |  |  |
| 0.201549 | 0.130594 |  |  |  |  |
| 0.201779 | 0.130755 |  |  |  |  |
| 0.201986 | 0.131146 |  |  |  |  |
| 0.202239 | 0.131031 |  |  |  |  |
| 0.202469 | 0.131123 |  |  |  |  |
| 0.202676 | 0.131353 |  |  |  |  |
| 0.202906 | 0.131698 |  |  |  |  |
| 0.203136 | 0.131813 |  |  |  |  |
| 0.203366 | 0.131744 |  |  |  |  |
| 0.203573 | 0.131928 |  |  |  |  |
| 0.203826 | 0.132388 |  |  |  |  |
| 0.204056 | 0.132434 |  |  |  |  |
| 0.204263 | 0.132618 |  |  |  |  |
| 0.204493 | 0.132388 |  |  |  |  |
| 0.204723 | 0.132848 |  |  |  |  |
| 0.20493 | 0.133147 |  |  |  |  |
| 0.20516 | 0.133078 |  |  |  |  |
| 0.205413 | 0.133216 |  |  |  |  |
| 0.20562 | 0.133446 |  |  |  |  |
| 0.20585 | 0.133722 |  |  |  |  |
| 0.20608 | 0.133837 |  |  |  |  |
| 0.20631 | 0.133883 |  |  |  |  |
| 0.206517 | 0.133906 |  |  |  |  |
| 0.206747 | 0.134481 |  |  |  |  |
| 0.206977 | 0.134274 |  |  |  |  |
| 0.207207 | 0.134504 |  |  |  |  |
| 0.207437 | 0.134941 |  |  |  |  |
| 0.207667 | 0.134941 |  |  |  |  |
| 0.207874 | 0.135332 |  |  |  |  |
| 0.208104 | 0.135424 |  |  |  |  |
| 0.208334 | 0.135539 |  |  |  |  |
| 0.208564 | 0.135516 |  |  |  |  |
| 0.208794 | 0.135746 |  |  |  |  |
| 0.209024 | 0.135631 |  |  |  |  |
| 0.209231 | 0.135999 |  |  |  |  |
| 0.209461 | 0.136022 |  |  |  |  |
| 0.209691 | 0.136436 |  |  |  |  |
| 0.209921 | 0.136413 |  |  |  |  |
| 0.210151 | 0.136689 |  |  |  |  |
| 0.210381 | 0.136735 |  |  |  |  |
| 0.210588 | 0.136804 |  |  |  |  |
| 0.210818 | 0.13708 |  |  |  |  |
| 0.211048 | 0.13708 |  |  |  |  |
| 0.211255 | 0.137356 |  |  |  |  |
| 0.211508 | 0.137793 |  |  |  |  |
| 0.211738 | 0.138 |  |  |  |  |
| 0.211968 | 0.138 |  |  |  |  |
| 0.212175 | 0.137908 |  |  |  |  |
| 0.212405 | 0.138115 |  |  |  |  |
| 0.212635 | 0.138552 |  |  |  |  |
| 0.212842 | 0.138575 |  |  |  |  |
| 0.213095 | 0.138805 |  |  |  |  |
| 0.213302 | 0.138897 |  |  |  |  |
| 0.213532 | 0.13892 |  |  |  |  |
| 0.213762 | 0.139265 |  |  |  |  |
| 0.213992 | 0.139196 |  |  |  |  |
| 0.214199 | 0.139035 |  |  |  |  |
| 0.214429 | 0.139725 |  |  |  |  |
| 0.214659 | 0.139932 |  |  |  |  |
| 0.214889 | 0.140047 |  |  |  |  |
| 0.215119 | 0.140139 |  |  |  |  |
| 0.215349 | 0.1403 |  |  |  |  |
| 0.215556 | 0.140714 |  |  |  |  |
| 0.215786 | 0.140714 |  |  |  |  |
| 0.216016 | 0.140806 |  |  |  |  |
| 0.216246 | 0.140622 |  |  |  |  |
| 0.216476 | 0.140967 |  |  |  |  |
| 0.216706 | 0.141243 |  |  |  |  |
| 0.216936 | 0.14122 |  |  |  |  |
| 0.217143 | 0.141312 |  |  |  |  |
| 0.217373 | 0.141979 |  |  |  |  |
| 0.217603 | 0.141933 |  |  |  |  |
| 0.217833 | 0.142209 |  |  |  |  |
| 0.218063 | 0.141979 |  |  |  |  |
| 0.218293 | 0.14214 |  |  |  |  |
| 0.2185 | 0.1426 |  |  |  |  |
| 0.21873 | 0.142577 |  |  |  |  |
| 0.21896 | 0.142761 |  |  |  |  |
| 0.219167 | 0.142876 |  |  |  |  |
| 0.21942 | 0.143336 |  |  |  |  |
| 0.21965 | 0.143359 |  |  |  |  |
| 0.21988 | 0.143543 |  |  |  |  |
| 0.220087 | 0.143451 |  |  |  |  |
| 0.220317 | 0.143957 |  |  |  |  |
| 0.220524 | 0.143681 |  |  |  |  |
| 0.220754 | 0.143934 |  |  |  |  |
| 0.221007 | 0.143957 |  |  |  |  |
| 0.221214 | 0.144417 |  |  |  |  |
| 0.221444 | 0.144486 |  |  |  |  |
| 0.221674 | 0.144601 |  |  |  |  |
| 0.221881 | 0.144532 |  |  |  |  |
| 0.222111 | 0.144785 |  |  |  |  |
| 0.222341 | 0.145084 |  |  |  |  |
| 0.222594 | 0.145291 |  |  |  |  |
| 0.222801 | 0.145314 |  |  |  |  |
| 0.223031 | 0.145383 |  |  |  |  |
| 0.223261 | 0.145797 |  |  |  |  |
| 0.223468 | 0.146073 |  |  |  |  |
| 0.223698 | 0.146234 |  |  |  |  |
| 0.223928 | 0.146303 |  |  |  |  |
| 0.224158 | 0.146556 |  |  |  |  |
| 0.224388 | 0.146694 |  |  |  |  |
| 0.224618 | 0.146878 |  |  |  |  |
| 0.224825 | 0.146625 |  |  |  |  |
| 0.225055 | 0.14697 |  |  |  |  |
| 0.225285 | 0.147269 |  |  |  |  |
| 0.225515 | 0.147361 |  |  |  |  |
| 0.225745 | 0.147338 |  |  |  |  |
| 0.225975 | 0.147568 |  |  |  |  |
| 0.226182 | 0.147729 |  |  |  |  |
| 0.226412 | 0.148005 |  |  |  |  |
| 0.226642 | 0.147982 |  |  |  |  |
| 0.226849 | 0.147959 |  |  |  |  |
| 0.227102 | 0.148626 |  |  |  |  |
| 0.227332 | 0.148695 |  |  |  |  |
| 0.227562 | 0.148764 |  |  |  |  |
| 0.227769 | 0.148856 |  |  |  |  |
| 0.227999 | 0.149201 |  |  |  |  |
| 0.228229 | 0.149224 |  |  |  |  |
| 0.228436 | 0.149454 |  |  |  |  |
| 0.228689 | 0.149339 |  |  |  |  |
| 0.228919 | 0.149661 |  |  |  |  |
| 0.229126 | 0.149937 |  |  |  |  |
| 0.229356 | 0.15019 |  |  |  |  |
| 0.229586 | 0.15042 |  |  |  |  |
| 0.229793 | 0.150351 |  |  |  |  |
| 0.230023 | 0.150328 |  |  |  |  |
| 0.230276 | 0.150512 |  |  |  |  |
| 0.230506 | 0.150857 |  |  |  |  |
| 0.230713 | 0.150995 |  |  |  |  |
| 0.230943 | 0.151294 |  |  |  |  |
| 0.231173 | 0.151501 |  |  |  |  |
| 0.23138 | 0.151524 |  |  |  |  |
| 0.23161 | 0.151409 |  |  |  |  |
| 0.231863 | 0.15157 |  |  |  |  |
| 0.23207 | 0.152007 |  |  |  |  |
| 0.2323 | 0.151869 |  |  |  |  |
| 0.23253 | 0.152283 |  |  |  |  |
| 0.232737 | 0.152375 |  |  |  |  |
| 0.232967 | 0.152467 |  |  |  |  |
| 0.233197 | 0.152766 |  |  |  |  |
| 0.233427 | 0.152674 |  |  |  |  |
| 0.233657 | 0.152651 |  |  |  |  |
| 0.233887 | 0.153065 |  |  |  |  |
| 0.234094 | 0.153226 |  |  |  |  |
| 0.234324 | 0.153456 |  |  |  |  |
| 0.234554 | 0.153433 |  |  |  |  |
| 0.234784 | 0.153732 |  |  |  |  |
| 0.235014 | 0.153893 |  |  |  |  |
| 0.235244 | 0.153824 |  |  |  |  |
| 0.235451 | 0.154192 |  |  |  |  |
| 0.235681 | 0.154353 |  |  |  |  |
| 0.235911 | 0.154698 |  |  |  |  |
| 0.236141 | 0.154721 |  |  |  |  |
| 0.236371 | 0.154698 |  |  |  |  |
| 0.236601 | 0.15479 |  |  |  |  |
| 0.236831 | 0.155043 |  |  |  |  |
| 0.237038 | 0.155135 |  |  |  |  |
| 0.237268 | 0.155411 |  |  |  |  |
| 0.237498 | 0.155572 |  |  |  |  |
| 0.237705 | 0.155825 |  |  |  |  |
| 0.237935 | 0.155871 |  |  |  |  |
| 0.238188 | 0.156055 |  |  |  |  |
| 0.238395 | 0.156124 |  |  |  |  |
| 0.238625 | 0.156331 |  |  |  |  |
| 0.238855 | 0.15663 |  |  |  |  |
| 0.239062 | 0.156607 |  |  |  |  |
| 0.239292 | 0.156722 |  |  |  |  |
| 0.239522 | 0.156837 |  |  |  |  |
| 0.239752 | 0.157159 |  |  |  |  |
| 0.239982 | 0.157504 |  |  |  |  |
| 0.240212 | 0.157343 |  |  |  |  |
| 0.240419 | 0.157504 |  |  |  |  |
| 0.240649 | 0.157711 |  |  |  |  |
| 0.240879 | 0.157757 |  |  |  |  |
| 0.241109 | 0.157642 |  |  |  |  |
| 0.241339 | 0.157734 |  |  |  |  |
| 0.241569 | 0.158355 |  |  |  |  |
| 0.241799 | 0.15847 |  |  |  |  |
| 0.242006 | 0.1587 |  |  |  |  |
| 0.242236 | 0.15893 |  |  |  |  |
| 0.242466 | 0.159068 |  |  |  |  |
| 0.242696 | 0.159068 |  |  |  |  |
| 0.242926 | 0.159091 |  |  |  |  |
| 0.243156 | 0.159367 |  |  |  |  |
| 0.243363 | 0.159666 |  |  |  |  |
| 0.243593 | 0.159873 |  |  |  |  |
| 0.243823 | 0.160103 |  |  |  |  |
| 0.24403 | 0.160034 |  |  |  |  |
| 0.244283 | 0.160379 |  |  |  |  |
| 0.244513 | 0.160448 |  |  |  |  |
| 0.24472 | 0.160287 |  |  |  |  |
| 0.24495 | 0.160724 |  |  |  |  |
| 0.24518 | 0.160448 |  |  |  |  |
| 0.245387 | 0.160954 |  |  |  |  |
| 0.245617 | 0.161368 |  |  |  |  |
| 0.24587 | 0.161368 |  |  |  |  |
| 0.246077 | 0.161253 |  |  |  |  |
| 0.246307 | 0.161529 |  |  |  |  |
| 0.246537 | 0.161759 |  |  |  |  |
| 0.246767 | 0.161966 |  |  |  |  |
| 0.246974 | 0.162311 |  |  |  |  |
| 0.247204 | 0.162173 |  |  |  |  |
| 0.247457 | 0.16238 |  |  |  |  |
| 0.247664 | 0.16307 |  |  |  |  |
| 0.247894 | 0.162748 |  |  |  |  |
| 0.248124 | 0.162978 |  |  |  |  |
| 0.248331 | 0.163116 |  |  |  |  |
| 0.248561 | 0.163323 |  |  |  |  |
| 0.248791 | 0.163369 |  |  |  |  |
| 0.249021 | 0.16353 |  |  |  |  |
| 0.249251 | 0.163691 |  |  |  |  |
| 0.249481 | 0.163921 |  |  |  |  |
| 0.249711 | 0.164197 |  |  |  |  |
| 0.249918 | 0.164105 |  |  |  |  |
| 0.250148 | 0.164059 |  |  |  |  |
| 0.250378 | 0.164519 |  |  |  |  |
| 0.250608 | 0.164703 |  |  |  |  |
| 0.250838 | 0.164703 |  |  |  |  |
| 0.251045 | 0.164749 |  |  |  |  |
| 0.251275 | 0.165048 |  |  |  |  |
| 0.251505 | 0.165209 |  |  |  |  |
| 0.251735 | 0.165393 |  |  |  |  |
| 0.251965 | 0.165416 |  |  |  |  |
| 0.252195 | 0.165577 |  |  |  |  |
| 0.252425 | 0.16606 |  |  |  |  |
| 0.252632 | 0.165899 |  |  |  |  |
| 0.252862 | 0.166152 |  |  |  |  |
| 0.253092 | 0.166313 |  |  |  |  |
| 0.253299 | 0.166543 |  |  |  |  |
| 0.253552 | 0.16652 |  |  |  |  |
| 0.253782 | 0.166474 |  |  |  |  |
| 0.253989 | 0.166704 |  |  |  |  |
| 0.254219 | 0.167233 |  |  |  |  |
| 0.254449 | 0.167233 |  |  |  |  |
| 0.254679 | 0.167256 |  |  |  |  |
| 0.254886 | 0.167463 |  |  |  |  |
| 0.255139 | 0.1679 |  |  |  |  |
| 0.255369 | 0.167946 |  |  |  |  |
| 0.255576 | 0.168107 |  |  |  |  |
| 0.255806 | 0.168107 |  |  |  |  |
| 0.256036 | 0.168291 |  |  |  |  |
| 0.256243 | 0.16859 |  |  |  |  |
| 0.256473 | 0.168659 |  |  |  |  |
| 0.256703 | 0.168521 |  |  |  |  |
| 0.256933 | 0.168797 |  |  |  |  |
| 0.257163 | 0.169372 |  |  |  |  |
| 0.257393 | 0.169188 |  |  |  |  |
| 0.2576 | 0.169303 |  |  |  |  |
| 0.25783 | 0.169349 |  |  |  |  |
| 0.25806 | 0.169579 |  |  |  |  |
| 0.25829 | 0.170062 |  |  |  |  |
| 0.25852 | 0.170039 |  |  |  |  |
| 0.25875 | 0.169924 |  |  |  |  |
| 0.258957 | 0.170407 |  |  |  |  |
| 0.259187 | 0.170706 |  |  |  |  |
| 0.259417 | 0.170844 |  |  |  |  |
| 0.259647 | 0.170775 |  |  |  |  |
| 0.259877 | 0.171051 |  |  |  |  |
| 0.260107 | 0.171442 |  |  |  |  |
| 0.260337 | 0.171396 |  |  |  |  |
| 0.260544 | 0.171258 |  |  |  |  |
| 0.260774 | 0.171603 |  |  |  |  |
| 0.261004 | 0.172109 |  |  |  |  |
| 0.261211 | 0.171994 |  |  |  |  |
| 0.261464 | 0.172063 |  |  |  |  |
| 0.261694 | 0.17227 |  |  |  |  |
| 0.261901 | 0.172454 |  |  |  |  |
| 0.262131 | 0.17273 |  |  |  |  |
| 0.262361 | 0.172753 |  |  |  |  |
| 0.262568 | 0.172753 |  |  |  |  |
| 0.262798 | 0.17296 |  |  |  |  |
| 0.263051 | 0.173236 |  |  |  |  |
| 0.263258 | 0.17319 |  |  |  |  |
| 0.263488 | 0.17342 |  |  |  |  |
| 0.263718 | 0.173443 |  |  |  |  |
| 0.263925 | 0.173742 |  |  |  |  |
| 0.264155 | 0.17411 |  |  |  |  |
| 0.264385 | 0.174179 |  |  |  |  |
| 0.264615 | 0.174087 |  |  |  |  |
| 0.264845 | 0.174432 |  |  |  |  |
| 0.265075 | 0.174708 |  |  |  |  |
| 0.265305 | 0.174593 |  |  |  |  |
| 0.265512 | 0.174754 |  |  |  |  |
| 0.265742 | 0.17503 |  |  |  |  |
| 0.265972 | 0.175122 |  |  |  |  |
| 0.266202 | 0.175375 |  |  |  |  |
| 0.266432 | 0.175306 |  |  |  |  |
| 0.266662 | 0.175651 |  |  |  |  |
| 0.266869 | 0.175789 |  |  |  |  |
| 0.267099 | 0.176226 |  |  |  |  |
| 0.267329 | 0.175927 |  |  |  |  |
| 0.267559 | 0.17618 |  |  |  |  |
| 0.267789 | 0.176594 |  |  |  |  |
| 0.268019 | 0.17664 |  |  |  |  |
| 0.268249 | 0.176916 |  |  |  |  |
| 0.268456 | 0.176663 |  |  |  |  |
| 0.268686 | 0.177146 |  |  |  |  |
| 0.268893 | 0.177284 |  |  |  |  |
| 0.269146 | 0.177353 |  |  |  |  |
| 0.269376 | 0.177629 |  |  |  |  |
| 0.269583 | 0.177767 |  |  |  |  |
| 0.269813 | 0.178089 |  |  |  |  |
| 0.270043 | 0.177974 |  |  |  |  |
| 0.27025 | 0.17802 |  |  |  |  |
| 0.27048 | 0.178204 |  |  |  |  |
| 0.270733 | 0.178687 |  |  |  |  |
| 0.270963 | 0.178618 |  |  |  |  |
| 0.27117 | 0.178457 |  |  |  |  |
| 0.2714 | 0.178664 |  |  |  |  |
| 0.27163 | 0.179055 |  |  |  |  |
| 0.271837 | 0.179193 |  |  |  |  |
| 0.272067 | 0.179469 |  |  |  |  |
| 0.27232 | 0.179377 |  |  |  |  |
| 0.272527 | 0.179722 |  |  |  |  |
| 0.272757 | 0.179745 |  |  |  |  |
| 0.272987 | 0.180044 |  |  |  |  |
| 0.273194 | 0.179975 |  |  |  |  |
| 0.273424 | 0.180458 |  |  |  |  |
| 0.273654 | 0.180711 |  |  |  |  |
| 0.273907 | 0.180412 |  |  |  |  |
| 0.274114 | 0.180596 |  |  |  |  |
| 0.274344 | 0.180665 |  |  |  |  |
| 0.274551 | 0.181102 |  |  |  |  |
| 0.274781 | 0.181332 |  |  |  |  |
| 0.275011 | 0.181332 |  |  |  |  |
| 0.275241 | 0.181378 |  |  |  |  |
| 0.275471 | 0.181516 |  |  |  |  |
| 0.275701 | 0.181953 |  |  |  |  |
| 0.275931 | 0.181999 |  |  |  |  |
| 0.276138 | 0.181884 |  |  |  |  |
| 0.276368 | 0.182252 |  |  |  |  |
| 0.276598 | 0.182574 |  |  |  |  |
| 0.276828 | 0.182436 |  |  |  |  |
| 0.277058 | 0.182643 |  |  |  |  |
| 0.277288 | 0.182689 |  |  |  |  |
| 0.277495 | 0.183195 |  |  |  |  |
| 0.277725 | 0.183195 |  |  |  |  |
| 0.277955 | 0.183149 |  |  |  |  |
| 0.278162 | 0.18331 |  |  |  |  |
| 0.278415 | 0.183724 |  |  |  |  |
| 0.278645 | 0.183747 |  |  |  |  |
| 0.278875 | 0.183977 |  |  |  |  |
| 0.279082 | 0.183816 |  |  |  |  |
| 0.279312 | 0.184253 |  |  |  |  |
| 0.279519 | 0.184483 |  |  |  |  |
| 0.279749 | 0.184437 |  |  |  |  |
| 0.279979 | 0.184506 |  |  |  |  |
| 0.280232 | 0.184782 |  |  |  |  |
| 0.280439 | 0.185012 |  |  |  |  |
| 0.280669 | 0.185173 |  |  |  |  |
| 0.280876 | 0.185311 |  |  |  |  |
| 0.281106 | 0.185334 |  |  |  |  |
| 0.281336 | 0.18561 |  |  |  |  |
| 0.281566 | 0.185794 |  |  |  |  |
| 0.281796 | 0.185564 |  |  |  |  |
| 0.282026 | 0.186047 |  |  |  |  |
| 0.282256 | 0.186392 |  |  |  |  |
| 0.282463 | 0.186323 |  |  |  |  |
| 0.282693 | 0.186599 |  |  |  |  |
| 0.282923 | 0.186622 |  |  |  |  |
| 0.283153 | 0.18676 |  |  |  |  |
| 0.283383 | 0.187013 |  |  |  |  |
| 0.283613 | 0.186921 |  |  |  |  |
| 0.28382 | 0.18699 |  |  |  |  |
| 0.28405 | 0.187358 |  |  |  |  |
| 0.28428 | 0.187634 |  |  |  |  |
| 0.28451 | 0.18791 |  |  |  |  |
| 0.28474 | 0.187864 |  |  |  |  |
| 0.28497 | 0.188117 |  |  |  |  |
| 0.2852 | 0.188393 |  |  |  |  |
| 0.285407 | 0.18837 |  |  |  |  |
| 0.285637 | 0.188462 |  |  |  |  |
| 0.285867 | 0.188646 |  |  |  |  |
| 0.286074 | 0.188784 |  |  |  |  |
| 0.286327 | 0.188991 |  |  |  |  |
| 0.286557 | 0.18883 |  |  |  |  |
| 0.286764 | 0.189083 |  |  |  |  |
| 0.286994 | 0.189474 |  |  |  |  |
| 0.287224 | 0.18975 |  |  |  |  |
| 0.287431 | 0.189842 |  |  |  |  |
| 0.287661 | 0.189888 |  |  |  |  |
| 0.287914 | 0.189865 |  |  |  |  |
| 0.288121 | 0.190509 |  |  |  |  |
| 0.288351 | 0.190164 |  |  |  |  |
| 0.288581 | 0.190417 |  |  |  |  |
| 0.288788 | 0.190578 |  |  |  |  |
| 0.289018 | 0.190923 |  |  |  |  |
| 0.289248 | 0.191038 |  |  |  |  |
| 0.289501 | 0.19113 |  |  |  |  |
| 0.289708 | 0.191084 |  |  |  |  |
| 0.289938 | 0.191383 |  |  |  |  |
| 0.290168 | 0.191498 |  |  |  |  |
| 0.290375 | 0.191774 |  |  |  |  |
| 0.290605 | 0.19182 |  |  |  |  |
| 0.290835 | 0.19205 |  |  |  |  |
| 0.291065 | 0.192234 |  |  |  |  |
| 0.291295 | 0.192165 |  |  |  |  |
| 0.291525 | 0.192257 |  |  |  |  |
| 0.291732 | 0.192717 |  |  |  |  |
| 0.291962 | 0.192947 |  |  |  |  |
| 0.292192 | 0.192947 |  |  |  |  |
| 0.292422 | 0.193039 |  |  |  |  |
| 0.292652 | 0.193154 |  |  |  |  |
| 0.292882 | 0.193407 |  |  |  |  |
| 0.293089 | 0.193453 |  |  |  |  |
| 0.293319 | 0.193476 |  |  |  |  |
| 0.293549 | 0.193614 |  |  |  |  |
| 0.293756 | 0.193844 |  |  |  |  |
| 0.294009 | 0.194005 |  |  |  |  |
| 0.294239 | 0.194258 |  |  |  |  |
| 0.294446 | 0.194258 |  |  |  |  |
| 0.294676 | 0.194327 |  |  |  |  |
| 0.294906 | 0.194741 |  |  |  |  |
| 0.295136 | 0.194948 |  |  |  |  |
| 0.295343 | 0.194787 |  |  |  |  |
| 0.295596 | 0.194925 |  |  |  |  |
| 0.295826 | 0.195385 |  |  |  |  |
| 0.296033 | 0.195431 |  |  |  |  |
| 0.296263 | 0.195592 |  |  |  |  |
| 0.296493 | 0.195408 |  |  |  |  |
| 0.2967 | 0.196121 |  |  |  |  |
| 0.29693 | 0.196121 |  |  |  |  |
| 0.297183 | 0.196305 |  |  |  |  |
| 0.29739 | 0.196213 |  |  |  |  |
| 0.29762 | 0.196535 |  |  |  |  |
| 0.29785 | 0.19688 |  |  |  |  |
| 0.298057 | 0.196558 |  |  |  |  |
| 0.298287 | 0.196788 |  |  |  |  |
| 0.298517 | 0.196995 |  |  |  |  |
| 0.298747 | 0.197386 |  |  |  |  |
| 0.298977 | 0.197478 |  |  |  |  |
| 0.299207 | 0.197478 |  |  |  |  |
| 0.299414 | 0.197363 |  |  |  |  |
| 0.299644 | 0.197892 |  |  |  |  |
| 0.299874 | 0.197892 |  |  |  |  |
| 0.300104 | 0.19803 |  |  |  |  |
| 0.300334 | 0.198329 |  |  |  |  |
| 0.300564 | 0.198559 |  |  |  |  |
| 0.300794 | 0.198628 |  |  |  |  |
| 0.301001 | 0.198927 |  |  |  |  |
| 0.301231 | 0.198973 |  |  |  |  |
| 0.301461 | 0.198927 |  |  |  |  |
| 0.301691 | 0.199364 |  |  |  |  |
| 0.301921 | 0.199456 |  |  |  |  |
| 0.302151 | 0.199548 |  |  |  |  |
| 0.302358 | 0.199663 |  |  |  |  |
| 0.302588 | 0.200054 |  |  |  |  |
| 0.302818 | 0.200031 |  |  |  |  |
| 0.303048 | 0.200077 |  |  |  |  |
| 0.303255 | 0.200146 |  |  |  |  |
| 0.303508 | 0.200514 |  |  |  |  |
| 0.303715 | 0.200491 |  |  |  |  |
| 0.303945 | 0.20056 |  |  |  |  |
| 0.304175 | 0.200951 |  |  |  |  |
| 0.304405 | 0.200974 |  |  |  |  |
| 0.304612 | 0.201273 |  |  |  |  |
| 0.304842 | 0.201273 |  |  |  |  |
| 0.305072 | 0.201411 |  |  |  |  |
| 0.305302 | 0.201733 |  |  |  |  |
| 0.305532 | 0.201894 |  |  |  |  |
| 0.305762 | 0.201963 |  |  |  |  |
| 0.305969 | 0.202124 |  |  |  |  |
| 0.306199 | 0.202308 |  |  |  |  |
| 0.306429 | 0.202722 |  |  |  |  |
| 0.306659 | 0.202469 |  |  |  |  |
| 0.306889 | 0.202952 |  |  |  |  |
| 0.307119 | 0.202768 |  |  |  |  |
| 0.307326 | 0.203044 |  |  |  |  |
| 0.307556 | 0.203412 |  |  |  |  |
| 0.307786 | 0.203458 |  |  |  |  |
| 0.308016 | 0.203297 |  |  |  |  |
| 0.308246 | 0.203573 |  |  |  |  |
| 0.308476 | 0.203803 |  |  |  |  |
| 0.308706 | 0.203964 |  |  |  |  |
| 0.308913 | 0.203941 |  |  |  |  |
| 0.309143 | 0.203941 |  |  |  |  |
| 0.309373 | 0.204677 |  |  |  |  |
| 0.309603 | 0.204562 |  |  |  |  |
| 0.309833 | 0.204539 |  |  |  |  |
| 0.310063 | 0.204792 |  |  |  |  |
| 0.31027 | 0.204838 |  |  |  |  |
| 0.3105 | 0.20516 |  |  |  |  |
| 0.31073 | 0.205091 |  |  |  |  |
| 0.310937 | 0.205137 |  |  |  |  |
| 0.31119 | 0.205413 |  |  |  |  |
| 0.31142 | 0.20585 |  |  |  |  |
| 0.311627 | 0.205666 |  |  |  |  |
| 0.311857 | 0.205735 |  |  |  |  |
| 0.312087 | 0.205988 |  |  |  |  |
| 0.312294 | 0.206264 |  |  |  |  |
| 0.312524 | 0.20654 |  |  |  |  |
| 0.312777 | 0.206448 |  |  |  |  |
| 0.312984 | 0.206517 |  |  |  |  |
| 0.313214 | 0.206954 |  |  |  |  |
| 0.313444 | 0.207115 |  |  |  |  |
| 0.313674 | 0.206977 |  |  |  |  |
| 0.313881 | 0.207276 |  |  |  |  |
| 0.314111 | 0.207552 |  |  |  |  |
| 0.314364 | 0.207621 |  |  |  |  |
| 0.314571 | 0.20769 |  |  |  |  |
| 0.314801 | 0.207736 |  |  |  |  |
| 0.315031 | 0.208104 |  |  |  |  |
| 0.315238 | 0.208058 |  |  |  |  |
| 0.315468 | 0.208288 |  |  |  |  |
| 0.315698 | 0.208449 |  |  |  |  |
| 0.315928 | 0.208541 |  |  |  |  |
| 0.316158 | 0.209139 |  |  |  |  |
| 0.316388 | 0.209001 |  |  |  |  |
| 0.316595 | 0.208886 |  |  |  |  |
| 0.316825 | 0.209024 |  |  |  |  |
| 0.317055 | 0.209415 |  |  |  |  |
| 0.317285 | 0.209714 |  |  |  |  |
| 0.317515 | 0.209829 |  |  |  |  |
| 0.317745 | 0.209668 |  |  |  |  |
| 0.317952 | 0.209898 |  |  |  |  |
| 0.318182 | 0.210312 |  |  |  |  |
| 0.318412 | 0.210473 |  |  |  |  |
| 0.318619 | 0.210404 |  |  |  |  |
| 0.318872 | 0.21068 |  |  |  |  |
| 0.319102 | 0.210864 |  |  |  |  |
| 0.319332 | 0.210818 |  |  |  |  |
| 0.319539 | 0.211094 |  |  |  |  |
| 0.319769 | 0.211094 |  |  |  |  |
| 0.319999 | 0.2116 |  |  |  |  |
| 0.320206 | 0.211439 |  |  |  |  |
| 0.320459 | 0.2116 |  |  |  |  |
| 0.320689 | 0.211623 |  |  |  |  |
| 0.320896 | 0.212037 |  |  |  |  |
| 0.321126 | 0.212359 |  |  |  |  |
| 0.321356 | 0.212083 |  |  |  |  |
| 0.321563 | 0.212152 |  |  |  |  |
| 0.321793 | 0.212382 |  |  |  |  |
| 0.322023 | 0.21298 |  |  |  |  |
| 0.322276 | 0.21275 |  |  |  |  |
| 0.322483 | 0.212957 |  |  |  |  |
| 0.322713 | 0.212865 |  |  |  |  |
| 0.32292 | 0.213578 |  |  |  |  |
| 0.32315 | 0.213532 |  |  |  |  |
| 0.32338 | 0.213509 |  |  |  |  |
| 0.32361 | 0.21367 |  |  |  |  |
| 0.32384 | 0.213854 |  |  |  |  |
| 0.32407 | 0.214199 |  |  |  |  |
| 0.3243 | 0.213923 |  |  |  |  |
| 0.324507 | 0.214084 |  |  |  |  |
| 0.324737 | 0.214406 |  |  |  |  |
| 0.324967 | 0.214613 |  |  |  |  |
| 0.325197 | 0.214659 |  |  |  |  |
| 0.325427 | 0.214797 |  |  |  |  |
| 0.325657 | 0.214935 |  |  |  |  |
| 0.325864 | 0.215188 |  |  |  |  |
| 0.326094 | 0.215257 |  |  |  |  |
| 0.326324 | 0.215556 |  |  |  |  |
| 0.326554 | 0.215464 |  |  |  |  |
| 0.326784 | 0.215901 |  |  |  |  |
| 0.327014 | 0.216223 |  |  |  |  |
| 0.327244 | 0.216085 |  |  |  |  |
| 0.327451 | 0.216407 |  |  |  |  |
| 0.327681 | 0.216361 |  |  |  |  |
| 0.327911 | 0.216798 |  |  |  |  |
| 0.328118 | 0.216752 |  |  |  |  |
| 0.328371 | 0.21643 |  |  |  |  |
| 0.328601 | 0.217074 |  |  |  |  |
| 0.328808 | 0.217074 |  |  |  |  |
| 0.329038 | 0.21758 |  |  |  |  |
| 0.329245 | 0.217235 |  |  |  |  |
| 0.329475 | 0.217419 |  |  |  |  |
| 0.329705 | 0.217741 |  |  |  |  |
| 0.329958 | 0.217764 |  |  |  |  |
| 0.330165 | 0.21781 |  |  |  |  |
| 0.330395 | 0.217994 |  |  |  |  |
| 0.330625 | 0.218224 |  |  |  |  |
| 0.330832 | 0.218316 |  |  |  |  |
| 0.331062 | 0.2185 |  |  |  |  |
| 0.331292 | 0.218661 |  |  |  |  |
| 0.331522 | 0.218799 |  |  |  |  |
| 0.331752 | 0.219075 |  |  |  |  |
| 0.331982 | 0.219144 |  |  |  |  |
| 0.332189 | 0.219213 |  |  |  |  |
| 0.332419 | 0.219328 |  |  |  |  |
| 0.332649 | 0.219627 |  |  |  |  |
| 0.332879 | 0.219719 |  |  |  |  |
| 0.333109 | 0.219765 |  |  |  |  |
| 0.333339 | 0.219742 |  |  |  |  |
| 0.333569 | 0.220271 |  |  |  |  |
| 0.333776 | 0.220156 |  |  |  |  |
| 0.334006 | 0.22034 |  |  |  |  |
| 0.334236 | 0.220616 |  |  |  |  |
| 0.334466 | 0.220478 |  |  |  |  |
| 0.334696 | 0.220846 |  |  |  |  |
| 0.334926 | 0.220754 |  |  |  |  |
| 0.335133 | 0.220846 |  |  |  |  |
| 0.335363 | 0.221329 |  |  |  |  |
| 0.335593 | 0.221789 |  |  |  |  |
| 0.3358 | 0.221881 |  |  |  |  |
| 0.336053 | 0.221628 |  |  |  |  |
| 0.336283 | 0.221651 |  |  |  |  |
| 0.33649 | 0.22218 |  |  |  |  |
| 0.33672 | 0.222456 |  |  |  |  |
| 0.33695 | 0.222226 |  |  |  |  |
| 0.337157 | 0.222364 |  |  |  |  |
| 0.337387 | 0.222571 |  |  |  |  |
| 0.33764 | 0.222824 |  |  |  |  |
| 0.33787 | 0.222847 |  |  |  |  |
| 0.338077 | 0.223031 |  |  |  |  |
| 0.338307 | 0.223399 |  |  |  |  |
| 0.338537 | 0.223376 |  |  |  |  |
| 0.338744 | 0.223399 |  |  |  |  |
| 0.338974 | 0.223652 |  |  |  |  |
| 0.339227 | 0.223606 |  |  |  |  |
| 0.339434 | 0.224204 |  |  |  |  |
| 0.339664 | 0.223974 |  |  |  |  |
| 0.339894 | 0.22402 |  |  |  |  |
| 0.340101 | 0.224135 |  |  |  |  |
| 0.340331 | 0.224641 |  |  |  |  |
| 0.340561 | 0.224779 |  |  |  |  |
| 0.340791 | 0.22448 |  |  |  |  |
| 0.341021 | 0.224802 |  |  |  |  |
| 0.341251 | 0.225216 |  |  |  |  |
| 0.341458 | 0.225193 |  |  |  |  |
| 0.341688 | 0.225285 |  |  |  |  |
| 0.341918 | 0.225515 |  |  |  |  |
| 0.342148 | 0.225423 |  |  |  |  |
| 0.342378 | 0.225699 |  |  |  |  |
| 0.342608 | 0.22586 |  |  |  |  |
| 0.342815 | 0.225814 |  |  |  |  |
| 0.343045 | 0.226205 |  |  |  |  |
| 0.343275 | 0.22655 |  |  |  |  |
| 0.343505 | 0.226458 |  |  |  |  |
| 0.343735 | 0.226573 |  |  |  |  |
| 0.343965 | 0.226573 |  |  |  |  |
| 0.344195 | 0.226803 |  |  |  |  |
| 0.344402 | 0.227194 |  |  |  |  |
| 0.344632 | 0.227033 |  |  |  |  |
| 0.344862 | 0.227194 |  |  |  |  |
| 0.345069 | 0.227332 |  |  |  |  |
| 0.345299 | 0.227585 |  |  |  |  |
| 0.345552 | 0.227654 |  |  |  |  |
| 0.345759 | 0.227608 |  |  |  |  |
| 0.345989 | 0.227999 |  |  |  |  |
| 0.346219 | 0.228206 |  |  |  |  |
| 0.346449 | 0.228459 |  |  |  |  |
| 0.346656 | 0.228574 |  |  |  |  |
| 0.346886 | 0.228505 |  |  |  |  |
| 0.347139 | 0.228873 |  |  |  |  |
| 0.347346 | 0.228804 |  |  |  |  |
| 0.347576 | 0.229103 |  |  |  |  |
| 0.347783 | 0.228942 |  |  |  |  |
| 0.348013 | 0.229287 |  |  |  |  |
| 0.348243 | 0.22954 |  |  |  |  |
| 0.348473 | 0.229425 |  |  |  |  |
| 0.348703 | 0.229839 |  |  |  |  |
| 0.348933 | 0.229839 |  |  |  |  |
| 0.349163 | 0.230437 |  |  |  |  |
| 0.34937 | 0.23023 |  |  |  |  |
| 0.3496 | 0.230345 |  |  |  |  |
| 0.34983 | 0.230644 |  |  |  |  |
| 0.35006 | 0.23069 |  |  |  |  |
| 0.35029 | 0.23092 |  |  |  |  |
| 0.35052 | 0.230782 |  |  |  |  |
| 0.350727 | 0.230989 |  |  |  |  |
| 0.350957 | 0.231403 |  |  |  |  |
| 0.351187 | 0.231564 |  |  |  |  |
| 0.351417 | 0.231541 |  |  |  |  |
| 0.351647 | 0.231633 |  |  |  |  |
| 0.351877 | 0.23184 |  |  |  |  |
| 0.352107 | 0.231886 |  |  |  |  |
| 0.352314 | 0.232093 |  |  |  |  |
| 0.352544 | 0.232001 |  |  |  |  |
| 0.352774 | 0.232185 |  |  |  |  |
| 0.352981 | 0.232553 |  |  |  |  |
| 0.353234 | 0.232691 |  |  |  |  |
| 0.353464 | 0.232829 |  |  |  |  |
| 0.353671 | 0.232737 |  |  |  |  |
| 0.353901 | 0.233266 |  |  |  |  |
| 0.354131 | 0.233128 |  |  |  |  |
| 0.354338 | 0.233197 |  |  |  |  |
| 0.354568 | 0.233381 |  |  |  |  |
| 0.354821 | 0.233818 |  |  |  |  |
| 0.355028 | 0.233772 |  |  |  |  |
| 0.355258 | 0.233841 |  |  |  |  |
| 0.355488 | 0.234117 |  |  |  |  |
| 0.355695 | 0.234255 |  |  |  |  |
| 0.355925 | 0.234393 |  |  |  |  |
| 0.356155 | 0.234554 |  |  |  |  |
| 0.356385 | 0.234623 |  |  |  |  |
| 0.356615 | 0.2346 |  |  |  |  |
| 0.356845 | 0.234807 |  |  |  |  |
| 0.357075 | 0.235106 |  |  |  |  |
| 0.357282 | 0.235106 |  |  |  |  |
| 0.357512 | 0.23529 |  |  |  |  |
| 0.357742 | 0.235704 |  |  |  |  |
| 0.357972 | 0.235704 |  |  |  |  |
| 0.358202 | 0.235911 |  |  |  |  |
| 0.358432 | 0.235727 |  |  |  |  |
| 0.358639 | 0.236141 |  |  |  |  |
| 0.358869 | 0.236325 |  |  |  |  |
| 0.359099 | 0.236279 |  |  |  |  |
| 0.359329 | 0.236371 |  |  |  |  |
| 0.359559 | 0.236693 |  |  |  |  |
| 0.359789 | 0.23667 |  |  |  |  |
| 0.359996 | 0.237038 |  |  |  |  |
| 0.360226 | 0.2369 |  |  |  |  |
| 0.360456 | 0.237061 |  |  |  |  |
| 0.360663 | 0.237383 |  |  |  |  |
| 0.360916 | 0.237636 |  |  |  |  |
| 0.361146 | 0.237636 |  |  |  |  |
| 0.361353 | 0.23782 |  |  |  |  |
| 0.361583 | 0.237912 |  |  |  |  |
| 0.361813 | 0.238119 |  |  |  |  |
| 0.362043 | 0.238096 |  |  |  |  |
| 0.36225 | 0.238142 |  |  |  |  |
| 0.362503 | 0.238418 |  |  |  |  |
| 0.362733 | 0.238648 |  |  |  |  |
| 0.36294 | 0.238717 |  |  |  |  |
| 0.36317 | 0.238763 |  |  |  |  |
| 0.3634 | 0.238924 |  |  |  |  |
| 0.363607 | 0.239292 |  |  |  |  |
| 0.363837 | 0.239062 |  |  |  |  |
| 0.364067 | 0.239361 |  |  |  |  |
| 0.364297 | 0.239476 |  |  |  |  |
| 0.364527 | 0.239729 |  |  |  |  |
| 0.364757 | 0.239752 |  |  |  |  |
| 0.364987 | 0.239959 |  |  |  |  |
| 0.365194 | 0.240097 |  |  |  |  |
| 0.365424 | 0.240419 |  |  |  |  |
| 0.365654 | 0.240465 |  |  |  |  |
| 0.365884 | 0.24058 |  |  |  |  |
| 0.366114 | 0.240488 |  |  |  |  |
| 0.366321 | 0.240948 |  |  |  |  |
| 0.366551 | 0.241086 |  |  |  |  |
| 0.366781 | 0.241385 |  |  |  |  |
| 0.367011 | 0.241316 |  |  |  |  |
| 0.367241 | 0.241569 |  |  |  |  |
| 0.367471 | 0.241592 |  |  |  |  |
| 0.367701 | 0.241845 |  |  |  |  |
| 0.367908 | 0.24196 |  |  |  |  |
| 0.368138 | 0.241937 |  |  |  |  |
| 0.368368 | 0.242098 |  |  |  |  |
| 0.368598 | 0.242144 |  |  |  |  |
| 0.368828 | 0.242374 |  |  |  |  |
| 0.369058 | 0.24242 |  |  |  |  |
| 0.369265 | 0.242558 |  |  |  |  |
| 0.369495 | 0.242926 |  |  |  |  |
| 0.369725 | 0.242857 |  |  |  |  |
| 0.369932 | 0.243087 |  |  |  |  |
| 0.370162 | 0.243179 |  |  |  |  |
| 0.370415 | 0.243547 |  |  |  |  |
| 0.370645 | 0.243616 |  |  |  |  |
| 0.370852 | 0.244076 |  |  |  |  |
| 0.371082 | 0.243593 |  |  |  |  |
| 0.371289 | 0.244099 |  |  |  |  |
| 0.371519 | 0.244214 |  |  |  |  |
| 0.371749 | 0.244214 |  |  |  |  |
| 0.371979 | 0.244191 |  |  |  |  |
| 0.372209 | 0.244628 |  |  |  |  |
| 0.372439 | 0.244697 |  |  |  |  |
| 0.372669 | 0.244766 |  |  |  |  |
| 0.372876 | 0.244789 |  |  |  |  |
| 0.373106 | 0.245065 |  |  |  |  |
| 0.373336 | 0.245272 |  |  |  |  |
| 0.373566 | 0.245203 |  |  |  |  |
| 0.373796 | 0.245226 |  |  |  |  |
| 0.374026 | 0.245456 |  |  |  |  |
| 0.374233 | 0.24587 |  |  |  |  |
| 0.374463 | 0.245686 |  |  |  |  |
| 0.374693 | 0.24587 |  |  |  |  |
| 0.374923 | 0.245962 |  |  |  |  |
| 0.375153 | 0.246123 |  |  |  |  |
| 0.375383 | 0.246192 |  |  |  |  |
| 0.375613 | 0.24656 |  |  |  |  |
| 0.37582 | 0.246422 |  |  |  |  |
| 0.37605 | 0.246951 |  |  |  |  |
| 0.376257 | 0.247135 |  |  |  |  |
| 0.37651 | 0.24702 |  |  |  |  |
| 0.37674 | 0.247089 |  |  |  |  |
| 0.376947 | 0.247411 |  |  |  |  |
| 0.377177 | 0.247664 |  |  |  |  |
| 0.377407 | 0.247641 |  |  |  |  |
| 0.377637 | 0.247756 |  |  |  |  |
| 0.377844 | 0.248032 |  |  |  |  |
| 0.378097 | 0.2484 |  |  |  |  |
| 0.378327 | 0.248262 |  |  |  |  |
| 0.378534 | 0.248101 |  |  |  |  |
| 0.378764 | 0.248331 |  |  |  |  |
| 0.378994 | 0.249182 |  |  |  |  |
| 0.379201 | 0.248929 |  |  |  |  |
| 0.379431 | 0.249067 |  |  |  |  |
| 0.379684 | 0.249067 |  |  |  |  |
| 0.379891 | 0.249228 |  |  |  |  |
| 0.380121 | 0.24932 |  |  |  |  |
| 0.380351 | 0.249412 |  |  |  |  |
| 0.380558 | 0.249527 |  |  |  |  |
| 0.380788 | 0.249619 |  |  |  |  |
| 0.381018 | 0.250033 |  |  |  |  |
| 0.381271 | 0.249918 |  |  |  |  |
| 0.381478 | 0.25024 |  |  |  |  |
| 0.381708 | 0.250286 |  |  |  |  |
| 0.381938 | 0.250493 |  |  |  |  |
| 0.382145 | 0.250562 |  |  |  |  |
| 0.382375 | 0.250447 |  |  |  |  |
| 0.382605 | 0.250631 |  |  |  |  |
| 0.382835 | 0.250907 |  |  |  |  |
| 0.383065 | 0.251068 |  |  |  |  |
| 0.383295 | 0.25116 |  |  |  |  |
| 0.383502 | 0.251114 |  |  |  |  |
| 0.383732 | 0.251275 |  |  |  |  |
| 0.383962 | 0.251689 |  |  |  |  |
| 0.384192 | 0.251827 |  |  |  |  |
| 0.384422 | 0.251758 |  |  |  |  |
| 0.384652 | 0.251988 |  |  |  |  |
| 0.384859 | 0.252425 |  |  |  |  |
| 0.385089 | 0.252563 |  |  |  |  |
| 0.385319 | 0.252448 |  |  |  |  |
| 0.385526 | 0.252586 |  |  |  |  |
| 0.385779 | 0.252793 |  |  |  |  |
| 0.386009 | 0.252931 |  |  |  |  |
| 0.386239 | 0.252908 |  |  |  |  |
| 0.386446 | 0.252954 |  |  |  |  |
| 0.386676 | 0.253207 |  |  |  |  |
| 0.386906 | 0.25346 |  |  |  |  |
| 0.387113 | 0.253621 |  |  |  |  |
| 0.387343 | 0.253713 |  |  |  |  |
| 0.387596 | 0.253759 |  |  |  |  |
| 0.387803 | 0.254242 |  |  |  |  |
| 0.388033 | 0.254196 |  |  |  |  |
| 0.388263 | 0.254403 |  |  |  |  |
| 0.38847 | 0.254196 |  |  |  |  |
| 0.3887 | 0.254771 |  |  |  |  |
| 0.38893 | 0.25484 |  |  |  |  |
| 0.38916 | 0.254909 |  |  |  |  |
| 0.38939 | 0.254771 |  |  |  |  |
| 0.38962 | 0.255438 |  |  |  |  |
| 0.389827 | 0.2553 |  |  |  |  |
| 0.390057 | 0.255277 |  |  |  |  |
| 0.390287 | 0.255438 |  |  |  |  |
| 0.390517 | 0.255553 |  |  |  |  |
| 0.390747 | 0.256013 |  |  |  |  |
| 0.390977 | 0.255944 |  |  |  |  |
| 0.391184 | 0.255944 |  |  |  |  |
| 0.391414 | 0.256312 |  |  |  |  |
| 0.391644 | 0.256404 |  |  |  |  |
| 0.391874 | 0.256496 |  |  |  |  |
| 0.392104 | 0.256772 |  |  |  |  |
| 0.392334 | 0.256703 |  |  |  |  |
| 0.392564 | 0.257048 |  |  |  |  |
| 0.392771 | 0.25714 |  |  |  |  |
| 0.393001 | 0.257071 |  |  |  |  |
| 0.393231 | 0.257416 |  |  |  |  |
| 0.393438 | 0.25737 |  |  |  |  |
| 0.393691 | 0.257692 |  |  |  |  |
| 0.393921 | 0.257715 |  |  |  |  |
| 0.394128 | 0.25783 |  |  |  |  |
| 0.394358 | 0.258175 |  |  |  |  |
| 0.394588 | 0.258428 |  |  |  |  |
| 0.394818 | 0.258336 |  |  |  |  |
| 0.395025 | 0.258497 |  |  |  |  |
| 0.395278 | 0.258589 |  |  |  |  |
| 0.395508 | 0.258888 |  |  |  |  |
| 0.395715 | 0.258934 |  |  |  |  |
| 0.395945 | 0.259049 |  |  |  |  |
| 0.396152 | 0.25898 |  |  |  |  |
| 0.396382 | 0.259095 |  |  |  |  |
| 0.396612 | 0.25967 |  |  |  |  |
| 0.396865 | 0.259831 |  |  |  |  |
| 0.397072 | 0.259509 |  |  |  |  |
| 0.397302 | 0.260061 |  |  |  |  |
| 0.397532 | 0.260153 |  |  |  |  |
| 0.397739 | 0.260199 |  |  |  |  |
| 0.397969 | 0.26036 |  |  |  |  |
| 0.398199 | 0.260429 |  |  |  |  |
| 0.398429 | 0.260613 |  |  |  |  |
| 0.398659 | 0.260636 |  |  |  |  |
| 0.398889 | 0.260751 |  |  |  |  |
| 0.399096 | 0.26105 |  |  |  |  |
| 0.399326 | 0.261303 |  |  |  |  |
| 0.399556 | 0.261188 |  |  |  |  |
| 0.399786 | 0.261372 |  |  |  |  |
| 0.400016 | 0.261602 |  |  |  |  |
| 0.400246 | 0.261786 |  |  |  |  |
| 0.400453 | 0.261924 |  |  |  |  |
| 0.400683 | 0.261993 |  |  |  |  |
| 0.400913 | 0.262131 |  |  |  |  |
| 0.401143 | 0.262361 |  |  |  |  |
| 0.401373 | 0.262407 |  |  |  |  |
| 0.401603 | 0.262568 |  |  |  |  |
| 0.401833 | 0.262775 |  |  |  |  |
| 0.40204 | 0.262683 |  |  |  |  |
| 0.40227 | 0.262936 |  |  |  |  |
| 0.4025 | 0.263028 |  |  |  |  |
| 0.402707 | 0.263028 |  |  |  |  |
| 0.40296 | 0.263281 |  |  |  |  |
| 0.40319 | 0.263511 |  |  |  |  |
| 0.403397 | 0.263741 |  |  |  |  |
| 0.403627 | 0.26381 |  |  |  |  |
| 0.403857 | 0.263833 |  |  |  |  |
| 0.404064 | 0.264109 |  |  |  |  |
| 0.404294 | 0.264247 |  |  |  |  |
| 0.404547 | 0.264339 |  |  |  |  |
| 0.404754 | 0.264431 |  |  |  |  |
| 0.404984 | 0.264799 |  |  |  |  |
| 0.405214 | 0.264569 |  |  |  |  |
| 0.405444 | 0.26496 |  |  |  |  |
| 0.405651 | 0.264753 |  |  |  |  |
| 0.405881 | 0.265121 |  |  |  |  |
| 0.406111 | 0.265397 |  |  |  |  |
| 0.406341 | 0.265282 |  |  |  |  |
| 0.406571 | 0.265305 |  |  |  |  |
| 0.406801 | 0.265673 |  |  |  |  |
| 0.407008 | 0.265788 |  |  |  |  |
| 0.407238 | 0.266225 |  |  |  |  |
| 0.407468 | 0.26588 |  |  |  |  |
| 0.407698 | 0.266202 |  |  |  |  |
| 0.407928 | 0.266478 |  |  |  |  |
| 0.408158 | 0.2668 |  |  |  |  |
| 0.408365 | 0.266823 |  |  |  |  |
| 0.408595 | 0.266662 |  |  |  |  |
| 0.408825 | 0.267007 |  |  |  |  |
| 0.409055 | 0.267076 |  |  |  |  |
| 0.409285 | 0.267283 |  |  |  |  |
| 0.409515 | 0.267122 |  |  |  |  |
| 0.409722 | 0.267398 |  |  |  |  |
| 0.409952 | 0.267628 |  |  |  |  |
| 0.410182 | 0.267628 |  |  |  |  |
| 0.410412 | 0.26772 |  |  |  |  |
| 0.410642 | 0.267927 |  |  |  |  |
| 0.410872 | 0.268042 |  |  |  |  |
| 0.411102 | 0.268364 |  |  |  |  |
| 0.411309 | 0.268203 |  |  |  |  |
| 0.411539 | 0.268387 |  |  |  |  |
| 0.411769 | 0.268686 |  |  |  |  |
| 0.411976 | 0.268709 |  |  |  |  |
| 0.412206 | 0.268847 |  |  |  |  |
| 0.412459 | 0.268801 |  |  |  |  |
| 0.412666 | 0.269054 |  |  |  |  |
| 0.412896 | 0.26956 |  |  |  |  |
| 0.413126 | 0.26933 |  |  |  |  |
| 0.413356 | 0.269629 |  |  |  |  |
| 0.413563 | 0.269468 |  |  |  |  |
| 0.413793 | 0.269951 |  |  |  |  |
| 0.414023 | 0.270296 |  |  |  |  |
| 0.414253 | 0.269905 |  |  |  |  |
| 0.414483 | 0.270296 |  |  |  |  |
| 0.41469 | 0.270296 |  |  |  |  |
| 0.41492 | 0.270618 |  |  |  |  |
| 0.41515 | 0.270825 |  |  |  |  |
| 0.41538 | 0.270664 |  |  |  |  |
| 0.41561 | 0.270779 |  |  |  |  |
| 0.41584 | 0.271009 |  |  |  |  |
| 0.41607 | 0.271078 |  |  |  |  |
| 0.416277 | 0.271377 |  |  |  |  |
| 0.416507 | 0.271331 |  |  |  |  |
| 0.416737 | 0.271584 |  |  |  |  |
| 0.416967 | 0.271699 |  |  |  |  |
| 0.417197 | 0.271768 |  |  |  |  |
| 0.417427 | 0.271538 |  |  |  |  |
| 0.417634 | 0.272182 |  |  |  |  |
| 0.417864 | 0.272228 |  |  |  |  |
| 0.418094 | 0.272596 |  |  |  |  |
| 0.418301 | 0.272481 |  |  |  |  |
| 0.418554 | 0.272941 |  |  |  |  |
| 0.418784 | 0.272711 |  |  |  |  |
| 0.419014 | 0.272849 |  |  |  |  |
| 0.419221 | 0.272826 |  |  |  |  |
| 0.419451 | 0.273033 |  |  |  |  |
| 0.419658 | 0.273424 |  |  |  |  |
| 0.419888 | 0.273562 |  |  |  |  |
| 0.420141 | 0.273401 |  |  |  |  |
| 0.420348 | 0.273562 |  |  |  |  |
| 0.420578 | 0.273953 |  |  |  |  |
| 0.420808 | 0.273999 |  |  |  |  |
| 0.421038 | 0.274045 |  |  |  |  |
| 0.421245 | 0.274045 |  |  |  |  |
| 0.421475 | 0.274229 |  |  |  |  |
| 0.421728 | 0.274712 |  |  |  |  |
| 0.421935 | 0.274735 |  |  |  |  |
| 0.422165 | 0.274643 |  |  |  |  |
| 0.422395 | 0.274758 |  |  |  |  |
| 0.422602 | 0.275402 |  |  |  |  |
| 0.422832 | 0.275333 |  |  |  |  |
| 0.423062 | 0.275517 |  |  |  |  |
| 0.423292 | 0.27531 |  |  |  |  |
| 0.423522 | 0.275632 |  |  |  |  |
| 0.423752 | 0.275678 |  |  |  |  |
| 0.423982 | 0.275862 |  |  |  |  |
| 0.424189 | 0.275655 |  |  |  |  |
| 0.424419 | 0.275954 |  |  |  |  |
| 0.424649 | 0.276322 |  |  |  |  |
| 0.424879 | 0.276207 |  |  |  |  |
| 0.425109 | 0.276161 |  |  |  |  |
| 0.425339 | 0.276575 |  |  |  |  |
| 0.425546 | 0.276897 |  |  |  |  |
| 0.425776 | 0.27692 |  |  |  |  |
| 0.426006 | 0.277058 |  |  |  |  |
| 0.426236 | 0.277035 |  |  |  |  |
| 0.426466 | 0.277495 |  |  |  |  |
| 0.426696 | 0.277403 |  |  |  |  |
| 0.426903 | 0.277587 |  |  |  |  |
| 0.427133 | 0.277702 |  |  |  |  |
| 0.427363 | 0.277955 |  |  |  |  |
| 0.42757 | 0.278001 |  |  |  |  |
| 0.427823 | 0.277932 |  |  |  |  |
| 0.428053 | 0.278277 |  |  |  |  |
| 0.42826 | 0.278415 |  |  |  |  |
| 0.42849 | 0.278783 |  |  |  |  |
| 0.42872 | 0.27876 |  |  |  |  |
| 0.428927 | 0.278668 |  |  |  |  |
| 0.429157 | 0.278829 |  |  |  |  |
| 0.429387 | 0.279151 |  |  |  |  |
| 0.42964 | 0.27922 |  |  |  |  |
| 0.429847 | 0.279358 |  |  |  |  |
| 0.430077 | 0.279266 |  |  |  |  |
| 0.430307 | 0.279772 |  |  |  |  |
| 0.430514 | 0.279726 |  |  |  |  |
| 0.430744 | 0.27991 |  |  |  |  |
| 0.430974 | 0.279887 |  |  |  |  |
| 0.431204 | 0.280048 |  |  |  |  |
| 0.431434 | 0.28037 |  |  |  |  |
| 0.431664 | 0.280393 |  |  |  |  |
| 0.431871 | 0.280531 |  |  |  |  |
| 0.432101 | 0.280623 |  |  |  |  |
| 0.432331 | 0.28106 |  |  |  |  |
| 0.432561 | 0.280945 |  |  |  |  |
| 0.432791 | 0.281267 |  |  |  |  |
| 0.433021 | 0.281382 |  |  |  |  |
| 0.433228 | 0.281359 |  |  |  |  |
| 0.433458 | 0.281819 |  |  |  |  |
| 0.433688 | 0.281244 |  |  |  |  |
| 0.433918 | 0.281796 |  |  |  |  |
| 0.434148 | 0.282118 |  |  |  |  |
| 0.434378 | 0.281934 |  |  |  |  |
| 0.434608 | 0.282003 |  |  |  |  |
| 0.434815 | 0.282164 |  |  |  |  |
| 0.435045 | 0.282371 |  |  |  |  |
| 0.435275 | 0.282647 |  |  |  |  |
| 0.435482 | 0.282555 |  |  |  |  |
| 0.435735 | 0.282624 |  |  |  |  |
| 0.435965 | 0.282854 |  |  |  |  |
| 0.436172 | 0.282785 |  |  |  |  |
| 0.436402 | 0.283084 |  |  |  |  |
| 0.436632 | 0.282877 |  |  |  |  |
| 0.436839 | 0.283107 |  |  |  |  |
| 0.437069 | 0.283544 |  |  |  |  |
| 0.437322 | 0.283567 |  |  |  |  |
| 0.437529 | 0.283406 |  |  |  |  |
| 0.437759 | 0.284004 |  |  |  |  |
| 0.437989 | 0.283866 |  |  |  |  |
| 0.438196 | 0.28428 |  |  |  |  |
| 0.438426 | 0.284234 |  |  |  |  |
| 0.438656 | 0.284418 |  |  |  |  |
| 0.438886 | 0.284418 |  |  |  |  |
| 0.439116 | 0.284487 |  |  |  |  |
| 0.439346 | 0.284878 |  |  |  |  |
| 0.439553 | 0.28497 |  |  |  |  |
| 0.439783 | 0.28497 |  |  |  |  |
| 0.440013 | 0.285246 |  |  |  |  |
| 0.440243 | 0.285292 |  |  |  |  |
| 0.440473 | 0.285315 |  |  |  |  |
| 0.440703 | 0.28543 |  |  |  |  |
| 0.440933 | 0.285752 |  |  |  |  |
| 0.44114 | 0.285821 |  |  |  |  |
| 0.44137 | 0.285844 |  |  |  |  |
| 0.4416 | 0.285936 |  |  |  |  |
| 0.44183 | 0.286373 |  |  |  |  |
| 0.44206 | 0.286235 |  |  |  |  |
| 0.44229 | 0.286488 |  |  |  |  |
| 0.442497 | 0.286557 |  |  |  |  |
| 0.442727 | 0.286695 |  |  |  |  |
| 0.442957 | 0.286902 |  |  |  |  |
| 0.443187 | 0.28681 |  |  |  |  |
| 0.443417 | 0.286994 |  |  |  |  |
| 0.443647 | 0.287362 |  |  |  |  |
| 0.443854 | 0.287477 |  |  |  |  |
| 0.444084 | 0.287615 |  |  |  |  |
| 0.444314 | 0.287753 |  |  |  |  |
| 0.444521 | 0.287707 |  |  |  |  |
| 0.444751 | 0.28819 |  |  |  |  |
| 0.445004 | 0.288121 |  |  |  |  |
| 0.445234 | 0.28819 |  |  |  |  |
| 0.445441 | 0.288374 |  |  |  |  |
| 0.445671 | 0.288558 |  |  |  |  |
| 0.445901 | 0.288834 |  |  |  |  |
| 0.446108 | 0.288949 |  |  |  |  |
| 0.446338 | 0.288903 |  |  |  |  |
| 0.446591 | 0.289156 |  |  |  |  |
| 0.446798 | 0.28934 |  |  |  |  |
| 0.447028 | 0.289317 |  |  |  |  |
| 0.447258 | 0.289409 |  |  |  |  |
| 0.447465 | 0.289478 |  |  |  |  |
| 0.447695 | 0.289639 |  |  |  |  |
| 0.447925 | 0.289915 |  |  |  |  |
| 0.448155 | 0.289846 |  |  |  |  |
| 0.448385 | 0.289961 |  |  |  |  |
| 0.448615 | 0.290076 |  |  |  |  |
| 0.448822 | 0.290582 |  |  |  |  |
| 0.449052 | 0.290444 |  |  |  |  |
| 0.449282 | 0.290467 |  |  |  |  |
| 0.449512 | 0.290766 |  |  |  |  |
| 0.449742 | 0.290927 |  |  |  |  |
| 0.449972 | 0.290835 |  |  |  |  |
| 0.450202 | 0.29118 |  |  |  |  |
| 0.450409 | 0.290996 |  |  |  |  |
| 0.450639 | 0.291686 |  |  |  |  |
| 0.450869 | 0.29164 |  |  |  |  |
| 0.451099 | 0.291318 |  |  |  |  |
| 0.451329 | 0.291433 |  |  |  |  |
| 0.451559 | 0.291962 |  |  |  |  |
| 0.451766 | 0.292238 |  |  |  |  |
| 0.451996 | 0.292077 |  |  |  |  |
| 0.452226 | 0.292192 |  |  |  |  |
| 0.452433 | 0.292675 |  |  |  |  |
| 0.452686 | 0.292629 |  |  |  |  |
| 0.452916 | 0.292767 |  |  |  |  |
| 0.453123 | 0.292813 |  |  |  |  |
| 0.453353 | 0.29279 |  |  |  |  |
| 0.453583 | 0.293227 |  |  |  |  |
| 0.453813 | 0.293204 |  |  |  |  |
| 0.45402 | 0.293273 |  |  |  |  |
| 0.45425 | 0.293411 |  |  |  |  |
| 0.454503 | 0.293687 |  |  |  |  |
| 0.45471 | 0.293756 |  |  |  |  |
| 0.45494 | 0.293894 |  |  |  |  |
| 0.45517 | 0.293549 |  |  |  |  |
| 0.455377 | 0.294078 |  |  |  |  |
| 0.455607 | 0.294239 |  |  |  |  |
| 0.455837 | 0.2944 |  |  |  |  |
| 0.456067 | 0.294515 |  |  |  |  |
| 0.456297 | 0.294584 |  |  |  |  |
| 0.456527 | 0.294814 |  |  |  |  |
| 0.456734 | 0.294745 |  |  |  |  |
| 0.456964 | 0.294952 |  |  |  |  |
| 0.457194 | 0.295067 |  |  |  |  |
| 0.457424 | 0.295274 |  |  |  |  |
| 0.457654 | 0.295389 |  |  |  |  |
| 0.457884 | 0.295481 |  |  |  |  |
| 0.458091 | 0.295458 |  |  |  |  |
| 0.458321 | 0.295987 |  |  |  |  |
| 0.458551 | 0.296125 |  |  |  |  |
| 0.458781 | 0.295849 |  |  |  |  |
| 0.459011 | 0.296217 |  |  |  |  |
| 0.459241 | 0.296263 |  |  |  |  |
| 0.459471 | 0.296332 |  |  |  |  |
| 0.459678 | 0.296585 |  |  |  |  |
| 0.459908 | 0.296585 |  |  |  |  |
| 0.460138 | 0.296815 |  |  |  |  |
| 0.460345 | 0.29693 |  |  |  |  |
| 0.460598 | 0.296884 |  |  |  |  |
| 0.460828 | 0.297206 |  |  |  |  |
| 0.461035 | 0.297482 |  |  |  |  |
| 0.461265 | 0.297689 |  |  |  |  |
| 0.461495 | 0.297758 |  |  |  |  |
| 0.461725 | 0.297597 |  |  |  |  |
| 0.461932 | 0.297988 |  |  |  |  |
| 0.462185 | 0.297965 |  |  |  |  |
| 0.462392 | 0.298103 |  |  |  |  |
| 0.462622 | 0.298264 |  |  |  |  |
| 0.462852 | 0.29808 |  |  |  |  |
| 0.463059 | 0.298471 |  |  |  |  |
| 0.463289 | 0.298701 |  |  |  |  |
| 0.463519 | 0.298724 |  |  |  |  |
| 0.463749 | 0.29877 |  |  |  |  |
| 0.463979 | 0.299023 |  |  |  |  |
| 0.464209 | 0.299437 |  |  |  |  |
| 0.464439 | 0.29923 |  |  |  |  |
| 0.464646 | 0.299138 |  |  |  |  |
| 0.464876 | 0.299368 |  |  |  |  |
| 0.465106 | 0.299782 |  |  |  |  |
| 0.465336 | 0.299897 |  |  |  |  |
| 0.465566 | 0.299897 |  |  |  |  |
| 0.465796 | 0.299828 |  |  |  |  |
| 0.466003 | 0.30015 |  |  |  |  |
| 0.466233 | 0.300219 |  |  |  |  |
| 0.466463 | 0.300656 |  |  |  |  |
| 0.466693 | 0.300495 |  |  |  |  |
| 0.466923 | 0.300518 |  |  |  |  |
| 0.467153 | 0.301093 |  |  |  |  |
| 0.467383 | 0.301024 |  |  |  |  |
| 0.46759 | 0.300771 |  |  |  |  |
| 0.46782 | 0.301139 |  |  |  |  |
| 0.468027 | 0.301231 |  |  |  |  |
| 0.46828 | 0.301162 |  |  |  |  |
| 0.46851 | 0.301415 |  |  |  |  |
| 0.468717 | 0.301645 |  |  |  |  |
| 0.468947 | 0.301852 |  |  |  |  |
| 0.469177 | 0.301921 |  |  |  |  |
| 0.469407 | 0.301645 |  |  |  |  |
| 0.469614 | 0.302105 |  |  |  |  |
| 0.469867 | 0.302289 |  |  |  |  |
| 0.470097 | 0.302427 |  |  |  |  |
| 0.470304 | 0.302542 |  |  |  |  |
| 0.470534 | 0.302473 |  |  |  |  |
| 0.470764 | 0.302887 |  |  |  |  |
| 0.470971 | 0.303025 |  |  |  |  |
| 0.471201 | 0.303048 |  |  |  |  |
| 0.471454 | 0.303094 |  |  |  |  |
| 0.471661 | 0.303186 |  |  |  |  |
| 0.471891 | 0.303623 |  |  |  |  |
| 0.472121 | 0.30337 |  |  |  |  |
| 0.472351 | 0.303485 |  |  |  |  |
| 0.472558 | 0.303577 |  |  |  |  |
| 0.472788 | 0.303922 |  |  |  |  |
| 0.473018 | 0.304106 |  |  |  |  |
| 0.473248 | 0.304267 |  |  |  |  |
| 0.473478 | 0.304221 |  |  |  |  |
| 0.473708 | 0.304244 |  |  |  |  |
| 0.473915 | 0.304704 |  |  |  |  |
| 0.474145 | 0.304842 |  |  |  |  |
| 0.474375 | 0.304681 |  |  |  |  |
| 0.474605 | 0.304888 |  |  |  |  |
| 0.474835 | 0.305233 |  |  |  |  |
| 0.475065 | 0.305417 |  |  |  |  |
| 0.475272 | 0.305141 |  |  |  |  |
| 0.475502 | 0.305486 |  |  |  |  |
| 0.475732 | 0.305532 |  |  |  |  |
| 0.475962 | 0.305762 |  |  |  |  |
| 0.476192 | 0.305946 |  |  |  |  |
| 0.476422 | 0.305877 |  |  |  |  |
| 0.476629 | 0.306291 |  |  |  |  |
| 0.476859 | 0.306429 |  |  |  |  |
| 0.477089 | 0.306383 |  |  |  |  |
| 0.477296 | 0.306406 |  |  |  |  |
| 0.477526 | 0.306521 |  |  |  |  |
| 0.477779 | 0.306567 |  |  |  |  |
| 0.478009 | 0.306958 |  |  |  |  |
| 0.478216 | 0.306981 |  |  |  |  |
| 0.478446 | 0.307004 |  |  |  |  |
| 0.478676 | 0.30705 |  |  |  |  |
| 0.478883 | 0.307556 |  |  |  |  |
| 0.479113 | 0.307257 |  |  |  |  |
| 0.479366 | 0.307487 |  |  |  |  |
| 0.479573 | 0.307832 |  |  |  |  |
| 0.479803 | 0.307924 |  |  |  |  |
| 0.480033 | 0.30774 |  |  |  |  |
| 0.48024 | 0.307947 |  |  |  |  |
| 0.48047 | 0.308039 |  |  |  |  |
| 0.4807 | 0.308384 |  |  |  |  |
| 0.48093 | 0.308361 |  |  |  |  |
| 0.48116 | 0.308384 |  |  |  |  |
| 0.48139 | 0.308614 |  |  |  |  |
| 0.481597 | 0.308913 |  |  |  |  |
| 0.481827 | 0.308913 |  |  |  |  |
| 0.482057 | 0.309028 |  |  |  |  |
| 0.482287 | 0.30912 |  |  |  |  |
| 0.482517 | 0.309281 |  |  |  |  |
| 0.482747 | 0.309373 |  |  |  |  |
| 0.482977 | 0.309258 |  |  |  |  |
| 0.483184 | 0.309442 |  |  |  |  |
| 0.483414 | 0.30981 |  |  |  |  |
| 0.483644 | 0.309925 |  |  |  |  |
| 0.483874 | 0.309764 |  |  |  |  |
| 0.484104 | 0.310132 |  |  |  |  |
| 0.484334 | 0.310431 |  |  |  |  |
| 0.484541 | 0.310477 |  |  |  |  |
| 0.484771 | 0.310592 |  |  |  |  |
| 0.485001 | 0.310523 |  |  |  |  |
| 0.485208 | 0.310523 |  |  |  |  |
| 0.485461 | 0.310891 |  |  |  |  |
| 0.485691 | 0.310799 |  |  |  |  |
| 0.485898 | 0.310799 |  |  |  |  |
| 0.486128 | 0.311213 |  |  |  |  |
| 0.486358 | 0.311305 |  |  |  |  |
| 0.486565 | 0.311489 |  |  |  |  |
| 0.486795 | 0.311535 |  |  |  |  |
| 0.487048 | 0.311351 |  |  |  |  |
| 0.487255 | 0.31188 |  |  |  |  |
| 0.487485 | 0.311949 |  |  |  |  |
| 0.487715 | 0.31188 |  |  |  |  |
| 0.487922 | 0.312294 |  |  |  |  |
| 0.488152 | 0.312248 |  |  |  |  |
| 0.488382 | 0.312593 |  |  |  |  |
| 0.488635 | 0.312524 |  |  |  |  |
| 0.488842 | 0.312662 |  |  |  |  |
| 0.489072 | 0.312754 |  |  |  |  |
| 0.489302 | 0.312892 |  |  |  |  |
| 0.489509 | 0.31326 |  |  |  |  |
| 0.489739 | 0.313053 |  |  |  |  |
| 0.489969 | 0.313237 |  |  |  |  |
| 0.490199 | 0.313605 |  |  |  |  |
| 0.490429 | 0.313628 |  |  |  |  |
| 0.490659 | 0.313582 |  |  |  |  |
| 0.490866 | 0.313881 |  |  |  |  |
| 0.491096 | 0.313858 |  |  |  |  |
| 0.491326 | 0.314088 |  |  |  |  |
| 0.491556 | 0.314226 |  |  |  |  |
| 0.491786 | 0.314111 |  |  |  |  |
| 0.492016 | 0.314456 |  |  |  |  |
| 0.492246 | 0.314502 |  |  |  |  |
| 0.492453 | 0.31464 |  |  |  |  |
| 0.492683 | 0.31464 |  |  |  |  |
| 0.49289 | 0.314548 |  |  |  |  |
| 0.493143 | 0.314962 |  |  |  |  |
| 0.493373 | 0.315146 |  |  |  |  |
| 0.493603 | 0.315169 |  |  |  |  |
| 0.49381 | 0.315031 |  |  |  |  |
| 0.49404 | 0.315399 |  |  |  |  |
| 0.49427 | 0.315422 |  |  |  |  |
| 0.494477 | 0.315583 |  |  |  |  |
| 0.49473 | 0.315675 |  |  |  |  |
| 0.49496 | 0.315974 |  |  |  |  |
| 0.495167 | 0.316434 |  |  |  |  |
| 0.495397 | 0.316112 |  |  |  |  |
| 0.495627 | 0.316273 |  |  |  |  |
| 0.495834 | 0.316434 |  |  |  |  |
| 0.496064 | 0.316664 |  |  |  |  |
| 0.496294 | 0.316894 |  |  |  |  |
| 0.496547 | 0.316503 |  |  |  |  |
| 0.496754 | 0.31671 |  |  |  |  |
| 0.496984 | 0.316986 |  |  |  |  |
| 0.497214 | 0.317101 |  |  |  |  |
| 0.497421 | 0.317377 |  |  |  |  |
| 0.497651 | 0.317285 |  |  |  |  |
| 0.497881 | 0.317676 |  |  |  |  |
| 0.498111 | 0.317607 |  |  |  |  |
| 0.498341 | 0.317745 |  |  |  |  |
| 0.498571 | 0.31786 |  |  |  |  |
| 0.498778 | 0.318182 |  |  |  |  |
| 0.499008 | 0.318067 |  |  |  |  |
| 0.499238 | 0.318228 |  |  |  |  |
| 0.499468 | 0.318481 |  |  |  |  |
| 0.499698 | 0.318205 |  |  |  |  |
| 0.499928 | 0.318504 |  |  |  |  |
| 0.500135 | 0.318619 |  |  |  |  |
| 0.500365 | 0.318941 |  |  |  |  |
| 0.500595 | 0.318481 |  |  |  |  |
| 0.500802 | 0.31901 |  |  |  |  |
| 0.501055 | 0.319102 |  |  |  |  |
| 0.501285 | 0.319102 |  |  |  |  |
| 0.501492 | 0.319148 |  |  |  |  |
| 0.501722 | 0.319447 |  |  |  |  |
| 0.501952 | 0.319631 |  |  |  |  |
| 0.502182 | 0.319608 |  |  |  |  |
| 0.502389 | 0.319746 |  |  |  |  |
| 0.502642 | 0.319654 |  |  |  |  |
| 0.502872 | 0.320321 |  |  |  |  |
| 0.503079 | 0.32016 |  |  |  |  |
| 0.503309 | 0.320298 |  |  |  |  |
| 0.503539 | 0.320505 |  |  |  |  |
| 0.503746 | 0.320712 |  |  |  |  |
| 0.503976 | 0.320781 |  |  |  |  |
| 0.504229 | 0.320758 |  |  |  |  |
| 0.504436 | 0.320942 |  |  |  |  |
| 0.504666 | 0.321264 |  |  |  |  |
| 0.504896 | 0.32108 |  |  |  |  |
| 0.505103 | 0.321655 |  |  |  |  |
| 0.505333 | 0.321425 |  |  |  |  |
| 0.505563 | 0.321425 |  |  |  |  |
| 0.505793 | 0.321862 |  |  |  |  |
| 0.506023 | 0.321931 |  |  |  |  |
| 0.506253 | 0.321862 |  |  |  |  |
| 0.50646 | 0.321954 |  |  |  |  |
| 0.50669 | 0.322414 |  |  |  |  |
| 0.50692 | 0.322598 |  |  |  |  |
| 0.50715 | 0.32246 |  |  |  |  |
| 0.50738 | 0.32269 |  |  |  |  |
| 0.50761 | 0.32269 |  |  |  |  |
| 0.50784 | 0.322989 |  |  |  |  |
| 0.508047 | 0.322782 |  |  |  |  |
| 0.508277 | 0.322966 |  |  |  |  |
| 0.508507 | 0.323127 |  |  |  |  |
| 0.508737 | 0.323495 |  |  |  |  |
| 0.508967 | 0.323242 |  |  |  |  |
| 0.509197 | 0.323403 |  |  |  |  |
| 0.509404 | 0.323472 |  |  |  |  |
| 0.509634 | 0.323978 |  |  |  |  |
| 0.509864 | 0.324139 |  |  |  |  |
| 0.510094 | 0.324139 |  |  |  |  |
| 0.510324 | 0.324116 |  |  |  |  |
| 0.510554 | 0.324323 |  |  |  |  |
| 0.510761 | 0.324576 |  |  |  |  |
| 0.510991 | 0.324438 |  |  |  |  |
| 0.511221 | 0.324553 |  |  |  |  |
| 0.511428 | 0.324829 |  |  |  |  |
| 0.511658 | 0.325059 |  |  |  |  |
| 0.511911 | 0.324967 |  |  |  |  |
| 0.512118 | 0.325013 |  |  |  |  |
| 0.512348 | 0.325128 |  |  |  |  |
| 0.512578 | 0.325358 |  |  |  |  |
| 0.512808 | 0.325496 |  |  |  |  |
| 0.513015 | 0.325611 |  |  |  |  |
| 0.513245 | 0.325726 |  |  |  |  |
| 0.513498 | 0.325956 |  |  |  |  |
| 0.513705 | 0.325818 |  |  |  |  |
| 0.513935 | 0.326048 |  |  |  |  |
| 0.514165 | 0.326048 |  |  |  |  |
| 0.514372 | 0.32614 |  |  |  |  |
| 0.514602 | 0.326416 |  |  |  |  |
| 0.514832 | 0.326439 |  |  |  |  |
| 0.515062 | 0.326623 |  |  |  |  |
| 0.515292 | 0.326554 |  |  |  |  |
| 0.515522 | 0.327037 |  |  |  |  |
| 0.515752 | 0.326876 |  |  |  |  |
| 0.515959 | 0.326945 |  |  |  |  |
| 0.516189 | 0.327152 |  |  |  |  |
| 0.516419 | 0.327497 |  |  |  |  |
| 0.516649 | 0.327589 |  |  |  |  |
| 0.516879 | 0.327566 |  |  |  |  |
| 0.517086 | 0.327543 |  |  |  |  |
| 0.517316 | 0.327589 |  |  |  |  |
| 0.517546 | 0.328233 |  |  |  |  |
| 0.517776 | 0.32798 |  |  |  |  |
| 0.518006 | 0.32821 |  |  |  |  |
| 0.518236 | 0.328463 |  |  |  |  |
| 0.518466 | 0.328578 |  |  |  |  |
| 0.518673 | 0.328601 |  |  |  |  |
| 0.518903 | 0.328509 |  |  |  |  |
| 0.519133 | 0.328762 |  |  |  |  |
| 0.51934 | 0.329153 |  |  |  |  |
| 0.51957 | 0.329199 |  |  |  |  |
| 0.519823 | 0.329061 |  |  |  |  |
| 0.52003 | 0.32936 |  |  |  |  |
| 0.52026 | 0.329544 |  |  |  |  |
| 0.52049 | 0.329659 |  |  |  |  |
| 0.52072 | 0.329774 |  |  |  |  |
| 0.520927 | 0.329797 |  |  |  |  |
| 0.521157 | 0.329912 |  |  |  |  |
| 0.52141 | 0.330027 |  |  |  |  |
| 0.521617 | 0.330119 |  |  |  |  |
| 0.521847 | 0.330142 |  |  |  |  |
| 0.522077 | 0.330464 |  |  |  |  |
| 0.522284 | 0.330901 |  |  |  |  |
| 0.522514 | 0.330579 |  |  |  |  |
| 0.522744 | 0.330786 |  |  |  |  |
| 0.522974 | 0.330855 |  |  |  |  |
| 0.523204 | 0.330993 |  |  |  |  |
| 0.523434 | 0.331016 |  |  |  |  |
| 0.523641 | 0.331177 |  |  |  |  |
| 0.523871 | 0.331384 |  |  |  |  |
| 0.524101 | 0.331545 |  |  |  |  |
| 0.524331 | 0.331821 |  |  |  |  |
| 0.524561 | 0.331752 |  |  |  |  |
| 0.524791 | 0.331936 |  |  |  |  |
| 0.524998 | 0.331867 |  |  |  |  |
| 0.525228 | 0.33212 |  |  |  |  |
| 0.525458 | 0.332097 |  |  |  |  |
| 0.525665 | 0.332212 |  |  |  |  |
| 0.525918 | 0.332672 |  |  |  |  |
| 0.526148 | 0.332741 |  |  |  |  |
| 0.526378 | 0.332764 |  |  |  |  |
| 0.526585 | 0.332649 |  |  |  |  |
| 0.526815 | 0.332603 |  |  |  |  |
| 0.527045 | 0.333109 |  |  |  |  |
| 0.527252 | 0.333063 |  |  |  |  |
| 0.527505 | 0.33327 |  |  |  |  |
| 0.527735 | 0.333385 |  |  |  |  |
| 0.527942 | 0.333316 |  |  |  |  |
| 0.528172 | 0.333684 |  |  |  |  |
| 0.528402 | 0.3335 |  |  |  |  |
| 0.528609 | 0.3335 |  |  |  |  |
| 0.528839 | 0.333983 |  |  |  |  |
| 0.529092 | 0.333914 |  |  |  |  |
| 0.529299 | 0.334167 |  |  |  |  |
| 0.529529 | 0.334121 |  |  |  |  |
| 0.529759 | 0.334259 |  |  |  |  |
| 0.529966 | 0.33465 |  |  |  |  |
| 0.530196 | 0.334581 |  |  |  |  |
| 0.530426 | 0.334604 |  |  |  |  |
| 0.530656 | 0.334811 |  |  |  |  |
| 0.530886 | 0.335225 |  |  |  |  |
| 0.531116 | 0.335248 |  |  |  |  |
| 0.531346 | 0.335156 |  |  |  |  |
| 0.531553 | 0.335248 |  |  |  |  |
| 0.531783 | 0.335294 |  |  |  |  |
| 0.532013 | 0.335363 |  |  |  |  |
| 0.532243 | 0.335478 |  |  |  |  |
| 0.532473 | 0.335478 |  |  |  |  |
| 0.532703 | 0.335823 |  |  |  |  |
| 0.53291 | 0.335938 |  |  |  |  |
| 0.53314 | 0.336122 |  |  |  |  |
| 0.53337 | 0.335938 |  |  |  |  |
| 0.5336 | 0.336122 |  |  |  |  |
| 0.53383 | 0.336467 |  |  |  |  |
| 0.53406 | 0.336444 |  |  |  |  |
| 0.534267 | 0.336628 |  |  |  |  |
| 0.534497 | 0.336697 |  |  |  |  |
| 0.534727 | 0.337088 |  |  |  |  |
| 0.534934 | 0.337088 |  |  |  |  |
| 0.535187 | 0.337134 |  |  |  |  |
| 0.535417 | 0.337042 |  |  |  |  |
| 0.535647 | 0.337479 |  |  |  |  |
| 0.535877 | 0.337824 |  |  |  |  |
| 0.536084 | 0.33741 |  |  |  |  |
| 0.536314 | 0.337663 |  |  |  |  |
| 0.536544 | 0.337801 |  |  |  |  |
| 0.536774 | 0.338146 |  |  |  |  |
| 0.537004 | 0.337916 |  |  |  |  |
| 0.537234 | 0.338238 |  |  |  |  |
| 0.537441 | 0.338215 |  |  |  |  |
| 0.537671 | 0.338698 |  |  |  |  |
| 0.537901 | 0.338514 |  |  |  |  |
| 0.538131 | 0.33856 |  |  |  |  |
| 0.538338 | 0.33879 |  |  |  |  |
| 0.538591 | 0.338813 |  |  |  |  |
| 0.538821 | 0.339112 |  |  |  |  |
| 0.539028 | 0.338951 |  |  |  |  |
| 0.539258 | 0.339158 |  |  |  |  |
| 0.539465 | 0.339388 |  |  |  |  |
| 0.539695 | 0.339572 |  |  |  |  |
| 0.539925 | 0.339733 |  |  |  |  |
| 0.540178 | 0.339549 |  |  |  |  |
| 0.540408 | 0.339871 |  |  |  |  |
| 0.540615 | 0.339963 |  |  |  |  |
| 0.540845 | 0.340239 |  |  |  |  |
| 0.541052 | 0.339963 |  |  |  |  |
| 0.541282 | 0.339963 |  |  |  |  |
| 0.541512 | 0.340561 |  |  |  |  |
| 0.541742 | 0.340515 |  |  |  |  |
| 0.541972 | 0.340676 |  |  |  |  |
| 0.542202 | 0.340883 |  |  |  |  |
| 0.542409 | 0.340814 |  |  |  |  |
| 0.542639 | 0.341205 |  |  |  |  |
| 0.542869 | 0.340883 |  |  |  |  |
| 0.543099 | 0.34132 |  |  |  |  |
| 0.543329 | 0.34109 |  |  |  |  |
| 0.543559 | 0.341665 |  |  |  |  |
| 0.543789 | 0.341366 |  |  |  |  |
| 0.543996 | 0.341435 |  |  |  |  |
| 0.544226 | 0.341849 |  |  |  |  |
| 0.544456 | 0.341918 |  |  |  |  |
| 0.544686 | 0.341987 |  |  |  |  |
| 0.544916 | 0.34201 |  |  |  |  |
| 0.545146 | 0.341964 |  |  |  |  |
| 0.545353 | 0.342516 |  |  |  |  |
| 0.545583 | 0.342562 |  |  |  |  |
| 0.545813 | 0.342378 |  |  |  |  |
| 0.54602 | 0.342677 |  |  |  |  |
| 0.546273 | 0.342631 |  |  |  |  |
| 0.546503 | 0.343068 |  |  |  |  |
| 0.54671 | 0.34316 |  |  |  |  |
| 0.54694 | 0.343045 |  |  |  |  |
| 0.54717 | 0.343229 |  |  |  |  |
| 0.547377 | 0.343459 |  |  |  |  |
| 0.547607 | 0.343528 |  |  |  |  |
| 0.54786 | 0.343735 |  |  |  |  |
| 0.548067 | 0.343551 |  |  |  |  |
| 0.548297 | 0.344218 |  |  |  |  |
| 0.548527 | 0.343712 |  |  |  |  |
| 0.548757 | 0.344126 |  |  |  |  |
| 0.548964 | 0.344218 |  |  |  |  |
| 0.549194 | 0.34431 |  |  |  |  |
| 0.549447 | 0.344379 |  |  |  |  |
| 0.549654 | 0.344701 |  |  |  |  |
| 0.549884 | 0.344586 |  |  |  |  |
| 0.550114 | 0.345 |  |  |  |  |
| 0.550321 | 0.345092 |  |  |  |  |
| 0.550551 | 0.345092 |  |  |  |  |
| 0.550781 | 0.345092 |  |  |  |  |
| 0.551011 | 0.345299 |  |  |  |  |
| 0.551241 | 0.345368 |  |  |  |  |
| 0.551471 | 0.345667 |  |  |  |  |
| 0.551678 | 0.345667 |  |  |  |  |
| 0.551908 | 0.345644 |  |  |  |  |
| 0.552138 | 0.345943 |  |  |  |  |
| 0.552368 | 0.34592 |  |  |  |  |
| 0.552598 | 0.346058 |  |  |  |  |
| 0.552828 | 0.346104 |  |  |  |  |
| 0.553035 | 0.346173 |  |  |  |  |
| 0.553265 | 0.346334 |  |  |  |  |
| 0.553495 | 0.346403 |  |  |  |  |
| 0.553725 | 0.346725 |  |  |  |  |
| 0.553955 | 0.346633 |  |  |  |  |
| 0.554185 | 0.347093 |  |  |  |  |
| 0.554415 | 0.347116 |  |  |  |  |
| 0.554622 | 0.346932 |  |  |  |  |
| 0.554852 | 0.3473 |  |  |  |  |
| 0.555082 | 0.347438 |  |  |  |  |
| 0.555289 | 0.347254 |  |  |  |  |
| 0.555542 | 0.34776 |  |  |  |  |
| 0.555772 | 0.347599 |  |  |  |  |
| 0.555979 | 0.347806 |  |  |  |  |
| 0.556209 | 0.34799 |  |  |  |  |
| 0.556439 | 0.348174 |  |  |  |  |
| 0.556646 | 0.347944 |  |  |  |  |
| 0.556876 | 0.348243 |  |  |  |  |
| 0.557106 | 0.348197 |  |  |  |  |
| 0.557359 | 0.348519 |  |  |  |  |
| 0.557566 | 0.348427 |  |  |  |  |
| 0.557796 | 0.348726 |  |  |  |  |
| 0.558026 | 0.34868 |  |  |  |  |
| 0.558233 | 0.34914 |  |  |  |  |
| 0.558463 | 0.349025 |  |  |  |  |
| 0.558693 | 0.349255 |  |  |  |  |
| 0.558923 | 0.349393 |  |  |  |  |
| 0.559153 | 0.349508 |  |  |  |  |
| 0.559383 | 0.349508 |  |  |  |  |
| 0.55959 | 0.349462 |  |  |  |  |
| 0.55982 | 0.34983 |  |  |  |  |
| 0.56005 | 0.349623 |  |  |  |  |
| 0.56028 | 0.349761 |  |  |  |  |
| 0.56051 | 0.349899 |  |  |  |  |
| 0.56074 | 0.350083 |  |  |  |  |
| 0.560947 | 0.350244 |  |  |  |  |
| 0.561177 | 0.350451 |  |  |  |  |
| 0.561407 | 0.350566 |  |  |  |  |
| 0.561614 | 0.350681 |  |  |  |  |
| 0.561867 | 0.350612 |  |  |  |  |
| 0.562097 | 0.350773 |  |  |  |  |
| 0.562327 | 0.35098 |  |  |  |  |
| 0.562534 | 0.351118 |  |  |  |  |
| 0.562764 | 0.351233 |  |  |  |  |
| 0.562971 | 0.35121 |  |  |  |  |
| 0.563201 | 0.351371 |  |  |  |  |
| 0.563454 | 0.351601 |  |  |  |  |
| 0.563684 | 0.351716 |  |  |  |  |
| 0.563891 | 0.351946 |  |  |  |  |
| 0.564121 | 0.351946 |  |  |  |  |
| 0.564351 | 0.352015 |  |  |  |  |
| 0.564558 | 0.351785 |  |  |  |  |
| 0.564788 | 0.352084 |  |  |  |  |
| 0.565041 | 0.352521 |  |  |  |  |
| 0.565248 | 0.352521 |  |  |  |  |
| 0.565478 | 0.352613 |  |  |  |  |
| 0.565708 | 0.352843 |  |  |  |  |
| 0.565915 | 0.352866 |  |  |  |  |
| 0.566145 | 0.352935 |  |  |  |  |
| 0.566375 | 0.352912 |  |  |  |  |
| 0.566605 | 0.353303 |  |  |  |  |
| 0.566835 | 0.353326 |  |  |  |  |
| 0.567065 | 0.353464 |  |  |  |  |
| 0.567272 | 0.353303 |  |  |  |  |
| 0.567502 | 0.353671 |  |  |  |  |
| 0.567732 | 0.353832 |  |  |  |  |
| 0.567962 | 0.353855 |  |  |  |  |
| 0.568192 | 0.353763 |  |  |  |  |
| 0.568422 | 0.353947 |  |  |  |  |
| 0.568652 | 0.35443 |  |  |  |  |
| 0.568859 | 0.354453 |  |  |  |  |
| 0.569089 | 0.35443 |  |  |  |  |
| 0.569319 | 0.354407 |  |  |  |  |
| 0.569549 | 0.354522 |  |  |  |  |
| 0.569779 | 0.354752 |  |  |  |  |
| 0.570009 | 0.35489 |  |  |  |  |
| 0.570216 | 0.354798 |  |  |  |  |
| 0.570446 | 0.355005 |  |  |  |  |
| 0.570676 | 0.355074 |  |  |  |  |
| 0.570883 | 0.355258 |  |  |  |  |
| 0.571136 | 0.355442 |  |  |  |  |
| 0.571366 | 0.355304 |  |  |  |  |
| 0.571573 | 0.355833 |  |  |  |  |
| 0.571803 | 0.355787 |  |  |  |  |
| 0.572033 | 0.355787 |  |  |  |  |
| 0.57224 | 0.355787 |  |  |  |  |
| 0.57247 | 0.355948 |  |  |  |  |
| 0.572723 | 0.356109 |  |  |  |  |
| 0.572953 | 0.356178 |  |  |  |  |
| 0.57316 | 0.356293 |  |  |  |  |
| 0.57339 | 0.356592 |  |  |  |  |
| 0.57362 | 0.356638 |  |  |  |  |
| 0.573827 | 0.356661 |  |  |  |  |
| 0.574057 | 0.356845 |  |  |  |  |
| 0.574287 | 0.356753 |  |  |  |  |
| 0.574517 | 0.357144 |  |  |  |  |
| 0.574747 | 0.357006 |  |  |  |  |
| 0.574977 | 0.357305 |  |  |  |  |
| 0.575184 | 0.357466 |  |  |  |  |
| 0.575414 | 0.357443 |  |  |  |  |
| 0.575644 | 0.357788 |  |  |  |  |
| 0.575874 | 0.357581 |  |  |  |  |
| 0.576104 | 0.35788 |  |  |  |  |
| 0.576334 | 0.357903 |  |  |  |  |
| 0.576541 | 0.358018 |  |  |  |  |
| 0.576771 | 0.358225 |  |  |  |  |
| 0.577001 | 0.358386 |  |  |  |  |
| 0.577231 | 0.358271 |  |  |  |  |
| 0.577461 | 0.358432 |  |  |  |  |
| 0.577691 | 0.358639 |  |  |  |  |
| 0.577921 | 0.358685 |  |  |  |  |
| 0.578128 | 0.358846 |  |  |  |  |
| 0.578358 | 0.359076 |  |  |  |  |
| 0.578588 | 0.358846 |  |  |  |  |
| 0.578818 | 0.359099 |  |  |  |  |
| 0.579048 | 0.359145 |  |  |  |  |
| 0.579278 | 0.359421 |  |  |  |  |
| 0.579485 | 0.359513 |  |  |  |  |
| 0.579715 | 0.359513 |  |  |  |  |
| 0.579945 | 0.359651 |  |  |  |  |
| 0.580152 | 0.359582 |  |  |  |  |
| 0.580382 | 0.359628 |  |  |  |  |
| 0.580635 | 0.359812 |  |  |  |  |
| 0.580842 | 0.359927 |  |  |  |  |
| 0.581072 | 0.360341 |  |  |  |  |
| 0.581302 | 0.360364 |  |  |  |  |
| 0.581532 | 0.360387 |  |  |  |  |
| 0.581739 | 0.360525 |  |  |  |  |
| 0.581969 | 0.360548 |  |  |  |  |
| 0.582199 | 0.3611 |  |  |  |  |
| 0.582429 | 0.360893 |  |  |  |  |
| 0.582659 | 0.360893 |  |  |  |  |
| 0.582866 | 0.360939 |  |  |  |  |
| 0.583096 | 0.361422 |  |  |  |  |
| 0.583326 | 0.36156 |  |  |  |  |
| 0.583556 | 0.361629 |  |  |  |  |
| 0.583786 | 0.361537 |  |  |  |  |
| 0.584016 | 0.361905 |  |  |  |  |
| 0.584246 | 0.361928 |  |  |  |  |
| 0.584453 | 0.361997 |  |  |  |  |
| 0.584683 | 0.361859 |  |  |  |  |
| 0.584913 | 0.362089 |  |  |  |  |
| 0.585143 | 0.362572 |  |  |  |  |
| 0.585373 | 0.362503 |  |  |  |  |
| 0.585603 | 0.362503 |  |  |  |  |
| 0.58581 | 0.362365 |  |  |  |  |
| 0.58604 | 0.362802 |  |  |  |  |
| 0.58627 | 0.362871 |  |  |  |  |
| 0.5865 | 0.36294 |  |  |  |  |
| 0.58673 | 0.362917 |  |  |  |  |
| 0.58696 | 0.363216 |  |  |  |  |
| 0.58719 | 0.363377 |  |  |  |  |
| 0.587397 | 0.363308 |  |  |  |  |
| 0.587627 | 0.363308 |  |  |  |  |
| 0.587857 | 0.363791 |  |  |  |  |
| 0.588064 | 0.363653 |  |  |  |  |
| 0.588317 | 0.363814 |  |  |  |  |
| 0.588547 | 0.363515 |  |  |  |  |
| 0.588754 | 0.363791 |  |  |  |  |
| 0.588984 | 0.364113 |  |  |  |  |
| 0.589214 | 0.364067 |  |  |  |  |
| 0.589421 | 0.363975 |  |  |  |  |
| 0.589651 | 0.364366 |  |  |  |  |
| 0.589904 | 0.364435 |  |  |  |  |
| 0.590111 | 0.364734 |  |  |  |  |
| 0.590341 | 0.364711 |  |  |  |  |
| 0.590571 | 0.36478 |  |  |  |  |
| 0.590778 | 0.364872 |  |  |  |  |
| 0.591008 | 0.365102 |  |  |  |  |
| 0.591238 | 0.365171 |  |  |  |  |
| 0.591468 | 0.365056 |  |  |  |  |
| 0.591698 | 0.365263 |  |  |  |  |
| 0.591928 | 0.365516 |  |  |  |  |
| 0.592158 | 0.36547 |  |  |  |  |
| 0.592365 | 0.365539 |  |  |  |  |
| 0.592595 | 0.366068 |  |  |  |  |
| 0.592825 | 0.366045 |  |  |  |  |
| 0.593055 | 0.366252 |  |  |  |  |
| 0.593285 | 0.366137 |  |  |  |  |
| 0.593515 | 0.36616 |  |  |  |  |
| 0.593722 | 0.366298 |  |  |  |  |
| 0.593952 | 0.366597 |  |  |  |  |
| 0.594182 | 0.366459 |  |  |  |  |
| 0.594412 | 0.366505 |  |  |  |  |
| 0.594642 | 0.366551 |  |  |  |  |
| 0.594872 | 0.366942 |  |  |  |  |
| 0.595079 | 0.36731 |  |  |  |  |
| 0.595309 | 0.367034 |  |  |  |  |
| 0.595539 | 0.36731 |  |  |  |  |
| 0.595746 | 0.36754 |  |  |  |  |
| 0.595999 | 0.367563 |  |  |  |  |
| 0.596229 | 0.367425 |  |  |  |  |
| 0.596436 | 0.367494 |  |  |  |  |
| 0.596666 | 0.367839 |  |  |  |  |
| 0.596896 | 0.368299 |  |  |  |  |
| 0.597126 | 0.367908 |  |  |  |  |
| 0.597333 | 0.36823 |  |  |  |  |
| 0.597586 | 0.368207 |  |  |  |  |
| 0.597816 | 0.368414 |  |  |  |  |
| 0.598023 | 0.368713 |  |  |  |  |
| 0.598253 | 0.368575 |  |  |  |  |
| 0.598483 | 0.368782 |  |  |  |  |
| 0.59869 | 0.368943 |  |  |  |  |
| 0.59892 | 0.369012 |  |  |  |  |
| 0.59915 | 0.368828 |  |  |  |  |
| 0.59938 | 0.369058 |  |  |  |  |
| 0.59961 | 0.369357 |  |  |  |  |
| 0.59984 | 0.36938 |  |  |  |  |
| 0.600047 | 0.369587 |  |  |  |  |
| 0.600277 | 0.369403 |  |  |  |  |
| 0.600507 | 0.369633 |  |  |  |  |
| 0.600737 | 0.369679 |  |  |  |  |
| 0.600967 | 0.369633 |  |  |  |  |
| 0.601197 | 0.369794 |  |  |  |  |
| 0.601404 | 0.369932 |  |  |  |  |
| 0.601634 | 0.370231 |  |  |  |  |
| 0.601864 | 0.370323 |  |  |  |  |
| 0.602094 | 0.370139 |  |  |  |  |
| 0.602324 | 0.370599 |  |  |  |  |
| 0.602554 | 0.370576 |  |  |  |  |
| 0.602784 | 0.370944 |  |  |  |  |
| 0.602991 | 0.370622 |  |  |  |  |
| 0.603221 | 0.37099 |  |  |  |  |
| 0.603451 | 0.37122 |  |  |  |  |
| 0.603658 | 0.371197 |  |  |  |  |
| 0.603911 | 0.370898 |  |  |  |  |
| 0.604141 | 0.371197 |  |  |  |  |
| 0.604348 | 0.371381 |  |  |  |  |
| 0.604578 | 0.371749 |  |  |  |  |
| 0.604808 | 0.371542 |  |  |  |  |
| 0.605015 | 0.371841 |  |  |  |  |
| 0.605245 | 0.37168 |  |  |  |  |
| 0.605498 | 0.371864 |  |  |  |  |
| 0.605728 | 0.371979 |  |  |  |  |
| 0.605935 | 0.372025 |  |  |  |  |
| 0.606165 | 0.372255 |  |  |  |  |
| 0.606395 | 0.372462 |  |  |  |  |
| 0.606602 | 0.372439 |  |  |  |  |
| 0.606832 | 0.372393 |  |  |  |  |
| 0.607062 | 0.372623 |  |  |  |  |
| 0.607292 | 0.372922 |  |  |  |  |
| 0.607522 | 0.372968 |  |  |  |  |
| 0.607752 | 0.373014 |  |  |  |  |
| 0.607959 | 0.373175 |  |  |  |  |
| 0.608189 | 0.373244 |  |  |  |  |
| 0.608419 | 0.373313 |  |  |  |  |
| 0.608649 | 0.373336 |  |  |  |  |
| 0.608879 | 0.373267 |  |  |  |  |
| 0.609109 | 0.373428 |  |  |  |  |
| 0.609316 | 0.373865 |  |  |  |  |
| 0.609546 | 0.373957 |  |  |  |  |
| 0.609776 | 0.373957 |  |  |  |  |
| 0.610006 | 0.374187 |  |  |  |  |
| 0.610236 | 0.374302 |  |  |  |  |
| 0.610466 | 0.374279 |  |  |  |  |
| 0.610696 | 0.374348 |  |  |  |  |
| 0.610903 | 0.374049 |  |  |  |  |
| 0.611133 | 0.374647 |  |  |  |  |
| 0.611363 | 0.374716 |  |  |  |  |
| 0.611593 | 0.374923 |  |  |  |  |
| 0.611823 | 0.374693 |  |  |  |  |
| 0.612053 | 0.3749 |  |  |  |  |
| 0.61226 | 0.375475 |  |  |  |  |
| 0.61249 | 0.375268 |  |  |  |  |
| 0.61272 | 0.375061 |  |  |  |  |
| 0.612927 | 0.375521 |  |  |  |  |
| 0.61318 | 0.375728 |  |  |  |  |
| 0.61341 | 0.375935 |  |  |  |  |
| 0.613617 | 0.375636 |  |  |  |  |
| 0.613847 | 0.375958 |  |  |  |  |
| 0.614077 | 0.376234 |  |  |  |  |
| 0.614284 | 0.376142 |  |  |  |  |
| 0.614514 | 0.37628 |  |  |  |  |
| 0.614767 | 0.376395 |  |  |  |  |
| 0.614974 | 0.376487 |  |  |  |  |
| 0.615204 | 0.376579 |  |  |  |  |
| 0.615434 | 0.376671 |  |  |  |  |
| 0.615641 | 0.376602 |  |  |  |  |
| 0.615871 | 0.376993 |  |  |  |  |
| 0.616101 | 0.377223 |  |  |  |  |
| 0.616354 | 0.377269 |  |  |  |  |
| 0.616561 | 0.376901 |  |  |  |  |
| 0.616791 | 0.377085 |  |  |  |  |
| 0.617021 | 0.377223 |  |  |  |  |
| 0.617228 | 0.377499 |  |  |  |  |
| 0.617458 | 0.377407 |  |  |  |  |
| 0.617688 | 0.377614 |  |  |  |  |
| 0.617918 | 0.377844 |  |  |  |  |
| 0.618148 | 0.377936 |  |  |  |  |
| 0.618378 | 0.378074 |  |  |  |  |
| 0.618585 | 0.378028 |  |  |  |  |
| 0.618815 | 0.378166 |  |  |  |  |
| 0.619045 | 0.378465 |  |  |  |  |
| 0.619275 | 0.378442 |  |  |  |  |
| 0.619505 | 0.378235 |  |  |  |  |
| 0.619735 | 0.378718 |  |  |  |  |
| 0.619942 | 0.378833 |  |  |  |  |
| 0.620172 | 0.378833 |  |  |  |  |
| 0.620402 | 0.37904 |  |  |  |  |
| 0.620609 | 0.378948 |  |  |  |  |
| 0.620862 | 0.379247 |  |  |  |  |
| 0.621092 | 0.379155 |  |  |  |  |
| 0.621322 | 0.379385 |  |  |  |  |
| 0.621529 | 0.379385 |  |  |  |  |
| 0.621759 | 0.3795 |  |  |  |  |
| 0.621989 | 0.379546 |  |  |  |  |
| 0.622196 | 0.37973 |  |  |  |  |
| 0.622426 | 0.379546 |  |  |  |  |
| 0.622679 | 0.379684 |  |  |  |  |
| 0.622886 | 0.38019 |  |  |  |  |
| 0.623116 | 0.380512 |  |  |  |  |
| 0.623346 | 0.380075 |  |  |  |  |
| 0.623553 | 0.380098 |  |  |  |  |
| 0.623783 | 0.380742 |  |  |  |  |
| 0.624013 | 0.380834 |  |  |  |  |
| 0.624266 | 0.380489 |  |  |  |  |
| 0.624473 | 0.380719 |  |  |  |  |
| 0.624703 | 0.380742 |  |  |  |  |
| 0.62491 | 0.381156 |  |  |  |  |
| 0.62514 | 0.381294 |  |  |  |  |
| 0.62537 | 0.380995 |  |  |  |  |
| 0.6256 | 0.381041 |  |  |  |  |
| 0.62583 | 0.381386 |  |  |  |  |
| 0.62606 | 0.381662 |  |  |  |  |
| 0.626267 | 0.381271 |  |  |  |  |
| 0.626497 | 0.38157 |  |  |  |  |
| 0.626727 | 0.381823 |  |  |  |  |
| 0.626957 | 0.381915 |  |  |  |  |
| 0.627187 | 0.381892 |  |  |  |  |
| 0.627417 | 0.381984 |  |  |  |  |
| 0.627647 | 0.38226 |  |  |  |  |
| 0.627854 | 0.382628 |  |  |  |  |
| 0.628084 | 0.382283 |  |  |  |  |
| 0.628314 | 0.382237 |  |  |  |  |
| 0.628521 | 0.382766 |  |  |  |  |
| 0.628774 | 0.38272 |  |  |  |  |
| 0.629004 | 0.382927 |  |  |  |  |
| 0.629211 | 0.382904 |  |  |  |  |
| 0.629441 | 0.382835 |  |  |  |  |
| 0.629671 | 0.383249 |  |  |  |  |
| 0.629901 | 0.383134 |  |  |  |  |
| 0.630108 | 0.383318 |  |  |  |  |
| 0.630361 | 0.383456 |  |  |  |  |
| 0.630591 | 0.383663 |  |  |  |  |
| 0.630798 | 0.383778 |  |  |  |  |
| 0.631028 | 0.383456 |  |  |  |  |
| 0.631235 | 0.383985 |  |  |  |  |
| 0.631465 | 0.383847 |  |  |  |  |
| 0.631695 | 0.384169 |  |  |  |  |
| 0.631948 | 0.384123 |  |  |  |  |
| 0.632155 | 0.384123 |  |  |  |  |
| 0.632385 | 0.384261 |  |  |  |  |
| 0.632615 | 0.384376 |  |  |  |  |
| 0.632822 | 0.384721 |  |  |  |  |
| 0.633052 | 0.384652 |  |  |  |  |
| 0.633282 | 0.384698 |  |  |  |  |
| 0.633512 | 0.384698 |  |  |  |  |
| 0.633742 | 0.384974 |  |  |  |  |
| 0.633972 | 0.384836 |  |  |  |  |
| 0.634179 | 0.385319 |  |  |  |  |
| 0.634409 | 0.385158 |  |  |  |  |
| 0.634639 | 0.385112 |  |  |  |  |
| 0.634869 | 0.385319 |  |  |  |  |
| 0.635099 | 0.385365 |  |  |  |  |
| 0.635329 | 0.38571 |  |  |  |  |
| 0.635559 | 0.385848 |  |  |  |  |
| 0.635766 | 0.385871 |  |  |  |  |
| 0.635996 | 0.385733 |  |  |  |  |
| 0.636226 | 0.386078 |  |  |  |  |
| 0.636456 | 0.386377 |  |  |  |  |
| 0.636686 | 0.386354 |  |  |  |  |
| 0.636916 | 0.386262 |  |  |  |  |
| 0.637123 | 0.386446 |  |  |  |  |
| 0.637353 | 0.386699 |  |  |  |  |
| 0.637583 | 0.386745 |  |  |  |  |
| 0.63779 | 0.38663 |  |  |  |  |
| 0.638043 | 0.386722 |  |  |  |  |
| 0.638273 | 0.386952 |  |  |  |  |
| 0.63848 | 0.387159 |  |  |  |  |
| 0.63871 | 0.386837 |  |  |  |  |
| 0.63894 | 0.387044 |  |  |  |  |
| 0.639147 | 0.387297 |  |  |  |  |
| 0.639377 | 0.387251 |  |  |  |  |
| 0.63963 | 0.387389 |  |  |  |  |
| 0.639837 | 0.387366 |  |  |  |  |
| 0.640067 | 0.387481 |  |  |  |  |
| 0.640297 | 0.387918 |  |  |  |  |
| 0.640527 | 0.388033 |  |  |  |  |
| 0.640734 | 0.387872 |  |  |  |  |
| 0.640964 | 0.388171 |  |  |  |  |
| 0.641194 | 0.388378 |  |  |  |  |
| 0.641424 | 0.388447 |  |  |  |  |
| 0.641654 | 0.388608 |  |  |  |  |
| 0.641884 | 0.388332 |  |  |  |  |
| 0.642091 | 0.388723 |  |  |  |  |
| 0.642321 | 0.388861 |  |  |  |  |
| 0.642551 | 0.388562 |  |  |  |  |
| 0.642781 | 0.388861 |  |  |  |  |
| 0.643011 | 0.388999 |  |  |  |  |
| 0.643241 | 0.389183 |  |  |  |  |
| 0.643448 | 0.389252 |  |  |  |  |
| 0.643678 | 0.389275 |  |  |  |  |
| 0.643908 | 0.389206 |  |  |  |  |
| 0.644138 | 0.389459 |  |  |  |  |
| 0.644368 | 0.389551 |  |  |  |  |
| 0.644598 | 0.389758 |  |  |  |  |
| 0.644805 | 0.389712 |  |  |  |  |
| 0.645035 | 0.390103 |  |  |  |  |
| 0.645265 | 0.390011 |  |  |  |  |
| 0.645495 | 0.390126 |  |  |  |  |
| 0.645702 | 0.389804 |  |  |  |  |
| 0.645955 | 0.390471 |  |  |  |  |
| 0.646185 | 0.390517 |  |  |  |  |
| 0.646392 | 0.390563 |  |  |  |  |
| 0.646622 | 0.39054 |  |  |  |  |
| 0.646852 | 0.390563 |  |  |  |  |
| 0.647059 | 0.390816 |  |  |  |  |
| 0.647289 | 0.390954 |  |  |  |  |
| 0.647542 | 0.390885 |  |  |  |  |
| 0.647749 | 0.391207 |  |  |  |  |
| 0.647979 | 0.391368 |  |  |  |  |
| 0.648209 | 0.391621 |  |  |  |  |
| 0.648416 | 0.391391 |  |  |  |  |
| 0.648646 | 0.391437 |  |  |  |  |
| 0.648876 | 0.391736 |  |  |  |  |
| 0.649106 | 0.39169 |  |  |  |  |
| 0.649336 | 0.391805 |  |  |  |  |
| 0.649566 | 0.391897 |  |  |  |  |
| 0.649773 | 0.392035 |  |  |  |  |
| 0.650003 | 0.392288 |  |  |  |  |
| 0.650233 | 0.392127 |  |  |  |  |
| 0.650463 | 0.39238 |  |  |  |  |
| 0.650693 | 0.392472 |  |  |  |  |
| 0.650923 | 0.392679 |  |  |  |  |
| 0.651153 | 0.392518 |  |  |  |  |
| 0.65136 | 0.392748 |  |  |  |  |
| 0.65159 | 0.392748 |  |  |  |  |
| 0.65182 | 0.393277 |  |  |  |  |
| 0.65205 | 0.393001 |  |  |  |  |
| 0.65228 | 0.393162 |  |  |  |  |
| 0.65251 | 0.393024 |  |  |  |  |
| 0.652717 | 0.393277 |  |  |  |  |
| 0.652947 | 0.393346 |  |  |  |  |
| 0.653177 | 0.393254 |  |  |  |  |
| 0.653384 | 0.393599 |  |  |  |  |
| 0.653637 | 0.393576 |  |  |  |  |
| 0.653867 | 0.393898 |  |  |  |  |
| 0.654097 | 0.393967 |  |  |  |  |
| 0.654304 | 0.394013 |  |  |  |  |
| 0.654534 | 0.39422 |  |  |  |  |
| 0.654741 | 0.394381 |  |  |  |  |
| 0.654971 | 0.394588 |  |  |  |  |
| 0.655224 | 0.394174 |  |  |  |  |
| 0.655431 | 0.394473 |  |  |  |  |
| 0.655661 | 0.394749 |  |  |  |  |
| 0.655891 | 0.394657 |  |  |  |  |
| 0.656121 | 0.394818 |  |  |  |  |
| 0.656328 | 0.394818 |  |  |  |  |
| 0.656558 | 0.395048 |  |  |  |  |
| 0.656811 | 0.395278 |  |  |  |  |
| 0.657018 | 0.395278 |  |  |  |  |
| 0.657248 | 0.395278 |  |  |  |  |
| 0.657478 | 0.395416 |  |  |  |  |
| 0.657685 | 0.395761 |  |  |  |  |
| 0.657915 | 0.395554 |  |  |  |  |
| 0.658145 | 0.395669 |  |  |  |  |
| 0.658375 | 0.395715 |  |  |  |  |
| 0.658605 | 0.396014 |  |  |  |  |
| 0.658835 | 0.396221 |  |  |  |  |
| 0.659065 | 0.396336 |  |  |  |  |
| 0.659272 | 0.396175 |  |  |  |  |
| 0.659502 | 0.396543 |  |  |  |  |
| 0.659732 | 0.396497 |  |  |  |  |
| 0.659962 | 0.396589 |  |  |  |  |
| 0.660192 | 0.396543 |  |  |  |  |
| 0.660422 | 0.396727 |  |  |  |  |
| 0.660629 | 0.396865 |  |  |  |  |
| 0.660859 | 0.397118 |  |  |  |  |
| 0.661089 | 0.397049 |  |  |  |  |
| 0.661319 | 0.396934 |  |  |  |  |
| 0.661549 | 0.397095 |  |  |  |  |
| 0.661779 | 0.397302 |  |  |  |  |
| 0.661986 | 0.397371 |  |  |  |  |
| 0.662216 | 0.397279 |  |  |  |  |
| 0.662446 | 0.397739 |  |  |  |  |
| 0.662653 | 0.397739 |  |  |  |  |
| 0.662906 | 0.397785 |  |  |  |  |
| 0.663136 | 0.397762 |  |  |  |  |
| 0.663343 | 0.39813 |  |  |  |  |
| 0.663573 | 0.398291 |  |  |  |  |
| 0.663803 | 0.398084 |  |  |  |  |
| 0.66401 | 0.398245 |  |  |  |  |
| 0.66424 | 0.398751 |  |  |  |  |
| 0.66447 | 0.398981 |  |  |  |  |
| 0.664723 | 0.398636 |  |  |  |  |
| 0.66493 | 0.398682 |  |  |  |  |
| 0.66516 | 0.398843 |  |  |  |  |
| 0.66539 | 0.399004 |  |  |  |  |
| 0.665597 | 0.399395 |  |  |  |  |
| 0.665827 | 0.399349 |  |  |  |  |
| 0.666057 | 0.399188 |  |  |  |  |
| 0.666287 | 0.39928 |  |  |  |  |
| 0.666517 | 0.399441 |  |  |  |  |
| 0.666747 | 0.39951 |  |  |  |  |
| 0.666954 | 0.399441 |  |  |  |  |
| 0.667184 | 0.399625 |  |  |  |  |
| 0.667414 | 0.399947 |  |  |  |  |
| 0.667644 | 0.399947 |  |  |  |  |
| 0.667874 | 0.399993 |  |  |  |  |
| 0.668104 | 0.400085 |  |  |  |  |
| 0.668311 | 0.400269 |  |  |  |  |
| 0.668541 | 0.400453 |  |  |  |  |
| 0.668771 | 0.400545 |  |  |  |  |
| 0.668978 | 0.400591 |  |  |  |  |
| 0.669231 | 0.400545 |  |  |  |  |
| 0.669461 | 0.400982 |  |  |  |  |
| 0.669691 | 0.400821 |  |  |  |  |
| 0.669898 | 0.401028 |  |  |  |  |
| 0.670128 | 0.401097 |  |  |  |  |
| 0.670358 | 0.401488 |  |  |  |  |
| 0.670565 | 0.40135 |  |  |  |  |
| 0.670818 | 0.40135 |  |  |  |  |
| 0.671048 | 0.401488 |  |  |  |  |
| 0.671255 | 0.401534 |  |  |  |  |
| 0.671485 | 0.401718 |  |  |  |  |
| 0.671715 | 0.401649 |  |  |  |  |
| 0.671922 | 0.401787 |  |  |  |  |
| 0.672152 | 0.402178 |  |  |  |  |
| 0.672405 | 0.401764 |  |  |  |  |
| 0.672635 | 0.401971 |  |  |  |  |
| 0.672842 | 0.402316 |  |  |  |  |
| 0.673072 | 0.402385 |  |  |  |  |
| 0.673279 | 0.402247 |  |  |  |  |
| 0.673509 | 0.402477 |  |  |  |  |
| 0.673739 | 0.402316 |  |  |  |  |
| 0.673992 | 0.402845 |  |  |  |  |
| 0.674199 | 0.402707 |  |  |  |  |
| 0.674429 | 0.402776 |  |  |  |  |
| 0.674659 | 0.40273 |  |  |  |  |
| 0.674866 | 0.403121 |  |  |  |  |
| 0.675096 | 0.403305 |  |  |  |  |
| 0.675326 | 0.403213 |  |  |  |  |
| 0.675556 | 0.403351 |  |  |  |  |
| 0.675786 | 0.403351 |  |  |  |  |
| 0.676016 | 0.403719 |  |  |  |  |
| 0.676223 | 0.403742 |  |  |  |  |
| 0.676453 | 0.403788 |  |  |  |  |
| 0.676683 | 0.403673 |  |  |  |  |
| 0.676913 | 0.404248 |  |  |  |  |
| 0.677143 | 0.404041 |  |  |  |  |
| 0.677373 | 0.404179 |  |  |  |  |
| 0.67758 | 0.404064 |  |  |  |  |
| 0.67781 | 0.404294 |  |  |  |  |
| 0.67804 | 0.404639 |  |  |  |  |
| 0.67827 | 0.404501 |  |  |  |  |
| 0.6785 | 0.404294 |  |  |  |  |
| 0.67873 | 0.404685 |  |  |  |  |
| 0.678937 | 0.404708 |  |  |  |  |
| 0.679167 | 0.404846 |  |  |  |  |
| 0.679397 | 0.404961 |  |  |  |  |
| 0.679604 | 0.404915 |  |  |  |  |
| 0.679834 | 0.405145 |  |  |  |  |
| 0.680087 | 0.405283 |  |  |  |  |
| 0.680317 | 0.405398 |  |  |  |  |
| 0.680524 | 0.405513 |  |  |  |  |
| 0.680754 | 0.405559 |  |  |  |  |
| 0.680984 | 0.405651 |  |  |  |  |
| 0.681191 | 0.40572 |  |  |  |  |
| 0.681421 | 0.405858 |  |  |  |  |
| 0.681674 | 0.405927 |  |  |  |  |
| 0.681881 | 0.40618 |  |  |  |  |
| 0.682111 | 0.40618 |  |  |  |  |
| 0.682341 | 0.406341 |  |  |  |  |
| 0.682548 | 0.406364 |  |  |  |  |
| 0.682778 | 0.406617 |  |  |  |  |
| 0.683008 | 0.406916 |  |  |  |  |
| 0.683238 | 0.406594 |  |  |  |  |
| 0.683468 | 0.406617 |  |  |  |  |
| 0.683698 | 0.406709 |  |  |  |  |
| 0.683928 | 0.406893 |  |  |  |  |
| 0.684135 | 0.407077 |  |  |  |  |
| 0.684365 | 0.406939 |  |  |  |  |
| 0.684595 | 0.406985 |  |  |  |  |
| 0.684825 | 0.407537 |  |  |  |  |
| 0.685055 | 0.40733 |  |  |  |  |
| 0.685285 | 0.407514 |  |  |  |  |
| 0.685492 | 0.407721 |  |  |  |  |
| 0.685722 | 0.407951 |  |  |  |  |
| 0.685952 | 0.407859 |  |  |  |  |
| 0.686182 | 0.40802 |  |  |  |  |
| 0.686412 | 0.40779 |  |  |  |  |
| 0.686642 | 0.407974 |  |  |  |  |
| 0.686849 | 0.408503 |  |  |  |  |
| 0.687079 | 0.408296 |  |  |  |  |
| 0.687309 | 0.408227 |  |  |  |  |
| 0.687516 | 0.408526 |  |  |  |  |
| 0.687746 | 0.40871 |  |  |  |  |
| 0.687999 | 0.408457 |  |  |  |  |
| 0.688206 | 0.408779 |  |  |  |  |
| 0.688436 | 0.408779 |  |  |  |  |
| 0.688666 | 0.408986 |  |  |  |  |
| 0.688896 | 0.409285 |  |  |  |  |
| 0.689103 | 0.409101 |  |  |  |  |
| 0.689333 | 0.409216 |  |  |  |  |
| 0.689586 | 0.409492 |  |  |  |  |
| 0.689793 | 0.409492 |  |  |  |  |
| 0.690023 | 0.409676 |  |  |  |  |
| 0.690253 | 0.409745 |  |  |  |  |
| 0.69046 | 0.409929 |  |  |  |  |
| 0.69069 | 0.41009 |  |  |  |  |
| 0.69092 | 0.409883 |  |  |  |  |
| 0.69115 | 0.409653 |  |  |  |  |
| 0.69138 | 0.410136 |  |  |  |  |
| 0.69161 | 0.410205 |  |  |  |  |
| 0.691817 | 0.410389 |  |  |  |  |
| 0.692047 | 0.410205 |  |  |  |  |
| 0.692277 | 0.410642 |  |  |  |  |
| 0.692507 | 0.41055 |  |  |  |  |
| 0.692737 | 0.41078 |  |  |  |  |
| 0.692967 | 0.410573 |  |  |  |  |
| 0.693174 | 0.41101 |  |  |  |  |
| 0.693404 | 0.410872 |  |  |  |  |
| 0.693634 | 0.411148 |  |  |  |  |
| 0.693864 | 0.411148 |  |  |  |  |
| 0.694094 | 0.41124 |  |  |  |  |
| 0.694324 | 0.411355 |  |  |  |  |
| 0.694554 | 0.41147 |  |  |  |  |
| 0.694761 | 0.411447 |  |  |  |  |
| 0.694991 | 0.41147 |  |  |  |  |
| 0.695221 | 0.411723 |  |  |  |  |
| 0.695428 | 0.411746 |  |  |  |  |
| 0.695681 | 0.412114 |  |  |  |  |
| 0.695911 | 0.411838 |  |  |  |  |
| 0.696118 | 0.412137 |  |  |  |  |
| 0.696348 | 0.412298 |  |  |  |  |
| 0.696578 | 0.412022 |  |  |  |  |
| 0.696785 | 0.412413 |  |  |  |  |
| 0.697015 | 0.412229 |  |  |  |  |
| 0.697268 | 0.412551 |  |  |  |  |
| 0.697475 | 0.412712 |  |  |  |  |
| 0.697705 | 0.412551 |  |  |  |  |
| 0.697935 | 0.412689 |  |  |  |  |
| 0.698142 | 0.412827 |  |  |  |  |
| 0.698372 | 0.413264 |  |  |  |  |
| 0.698602 | 0.413126 |  |  |  |  |
| 0.698832 | 0.413218 |  |  |  |  |
| 0.699062 | 0.413356 |  |  |  |  |
| 0.699292 | 0.413494 |  |  |  |  |
| 0.699522 | 0.413586 |  |  |  |  |
| 0.699729 | 0.413563 |  |  |  |  |
| 0.699959 | 0.413678 |  |  |  |  |
| 0.700189 | 0.414023 |  |  |  |  |
| 0.700419 | 0.413908 |  |  |  |  |
| 0.700649 | 0.413908 |  |  |  |  |
| 0.700879 | 0.414138 |  |  |  |  |
| 0.701086 | 0.414299 |  |  |  |  |
| 0.701316 | 0.414483 |  |  |  |  |
| 0.701546 | 0.414437 |  |  |  |  |
| 0.701776 | 0.414345 |  |  |  |  |
| 0.702006 | 0.414644 |  |  |  |  |
| 0.702236 | 0.414736 |  |  |  |  |
| 0.702466 | 0.414713 |  |  |  |  |
| 0.702673 | 0.414759 |  |  |  |  |
| 0.702903 | 0.414713 |  |  |  |  |
| 0.703133 | 0.415173 |  |  |  |  |
| 0.703363 | 0.415288 |  |  |  |  |
| 0.703593 | 0.415265 |  |  |  |  |
| 0.7038 | 0.415127 |  |  |  |  |
| 0.70403 | 0.415472 |  |  |  |  |
| 0.70426 | 0.415656 |  |  |  |  |
| 0.70449 | 0.415863 |  |  |  |  |
| 0.704697 | 0.415541 |  |  |  |  |
| 0.70495 | 0.415679 |  |  |  |  |
| 0.70518 | 0.416001 |  |  |  |  |
| 0.705387 | 0.415909 |  |  |  |  |
| 0.705617 | 0.415909 |  |  |  |  |
| 0.705847 | 0.416139 |  |  |  |  |
| 0.706054 | 0.416001 |  |  |  |  |
| 0.706284 | 0.416392 |  |  |  |  |
| 0.706514 | 0.4163 |  |  |  |  |
| 0.706744 | 0.41653 |  |  |  |  |
| 0.706974 | 0.417036 |  |  |  |  |
| 0.707204 | 0.416852 |  |  |  |  |
| 0.707434 | 0.41676 |  |  |  |  |
| 0.707641 | 0.416829 |  |  |  |  |
| 0.707871 | 0.416944 |  |  |  |  |
| 0.708101 | 0.417174 |  |  |  |  |
| 0.708331 | 0.417151 |  |  |  |  |
| 0.708561 | 0.417358 |  |  |  |  |
| 0.708791 | 0.417381 |  |  |  |  |
| 0.708998 | 0.417634 |  |  |  |  |
| 0.709228 | 0.417588 |  |  |  |  |
| 0.709458 | 0.417703 |  |  |  |  |
| 0.709688 | 0.417818 |  |  |  |  |
| 0.709918 | 0.41814 |  |  |  |  |
| 0.710148 | 0.418048 |  |  |  |  |
| 0.710355 | 0.418163 |  |  |  |  |
| 0.710585 | 0.418002 |  |  |  |  |
| 0.710815 | 0.418301 |  |  |  |  |
| 0.711022 | 0.418393 |  |  |  |  |
| 0.711275 | 0.418393 |  |  |  |  |
| 0.711505 | 0.418508 |  |  |  |  |
| 0.711712 | 0.418738 |  |  |  |  |
| 0.711942 | 0.418899 |  |  |  |  |
| 0.712172 | 0.418968 |  |  |  |  |
| 0.712379 | 0.418738 |  |  |  |  |
| 0.712609 | 0.418922 |  |  |  |  |
| 0.712862 | 0.419152 |  |  |  |  |
| 0.713092 | 0.419267 |  |  |  |  |
| 0.713299 | 0.419221 |  |  |  |  |
| 0.713529 | 0.419244 |  |  |  |  |
| 0.713759 | 0.419543 |  |  |  |  |
| 0.713966 | 0.41952 |  |  |  |  |
| 0.714196 | 0.419612 |  |  |  |  |
| 0.714449 | 0.419474 |  |  |  |  |
| 0.714656 | 0.419888 |  |  |  |  |
| 0.714886 | 0.420049 |  |  |  |  |
| 0.715116 | 0.419911 |  |  |  |  |
| 0.715323 | 0.419865 |  |  |  |  |
| 0.715553 | 0.420486 |  |  |  |  |
| 0.715783 | 0.420509 |  |  |  |  |
| 0.716013 | 0.420302 |  |  |  |  |
| 0.716243 | 0.420417 |  |  |  |  |
| 0.716473 | 0.420486 |  |  |  |  |
| 0.71668 | 0.420808 |  |  |  |  |
| 0.71691 | 0.420808 |  |  |  |  |
| 0.71714 | 0.420762 |  |  |  |  |
| 0.71737 | 0.420992 |  |  |  |  |
| 0.7176 | 0.421176 |  |  |  |  |
| 0.71783 | 0.421337 |  |  |  |  |
| 0.71806 | 0.421291 |  |  |  |  |
| 0.718267 | 0.421107 |  |  |  |  |
| 0.718497 | 0.421406 |  |  |  |  |
| 0.718727 | 0.42159 |  |  |  |  |
| 0.718957 | 0.421682 |  |  |  |  |
| 0.719187 | 0.421636 |  |  |  |  |
| 0.719417 | 0.421774 |  |  |  |  |
| 0.719624 | 0.422027 |  |  |  |  |
| 0.719854 | 0.422234 |  |  |  |  |
| 0.720084 | 0.422119 |  |  |  |  |
| 0.720291 | 0.422073 |  |  |  |  |
| 0.720544 | 0.422487 |  |  |  |  |
| 0.720774 | 0.422487 |  |  |  |  |
| 0.721004 | 0.422165 |  |  |  |  |
| 0.721211 | 0.422441 |  |  |  |  |
| 0.721441 | 0.422786 |  |  |  |  |
| 0.721648 | 0.42297 |  |  |  |  |
| 0.721878 | 0.42297 |  |  |  |  |
| 0.722131 | 0.422855 |  |  |  |  |
| 0.722338 | 0.422763 |  |  |  |  |
| 0.722568 | 0.423315 |  |  |  |  |
| 0.722798 | 0.423177 |  |  |  |  |
| 0.723005 | 0.423223 |  |  |  |  |
| 0.723235 | 0.423361 |  |  |  |  |
| 0.723465 | 0.423568 |  |  |  |  |
| 0.723718 | 0.423499 |  |  |  |  |
| 0.723925 | 0.423499 |  |  |  |  |
| 0.724155 | 0.423729 |  |  |  |  |
| 0.724385 | 0.424028 |  |  |  |  |
| 0.724592 | 0.424074 |  |  |  |  |
| 0.724822 | 0.423821 |  |  |  |  |
| 0.725052 | 0.423959 |  |  |  |  |
| 0.725282 | 0.424304 |  |  |  |  |
| 0.725512 | 0.424212 |  |  |  |  |
| 0.725742 | 0.42435 |  |  |  |  |
| 0.725949 | 0.424258 |  |  |  |  |
| 0.726179 | 0.424373 |  |  |  |  |
| 0.726409 | 0.424327 |  |  |  |  |
| 0.726639 | 0.424511 |  |  |  |  |
| 0.726869 | 0.424626 |  |  |  |  |
| 0.727099 | 0.424695 |  |  |  |  |
| 0.727329 | 0.425109 |  |  |  |  |
| 0.727536 | 0.425017 |  |  |  |  |
| 0.727766 | 0.425086 |  |  |  |  |
| 0.727973 | 0.425201 |  |  |  |  |
| 0.728226 | 0.425431 |  |  |  |  |
| 0.728456 | 0.425477 |  |  |  |  |
| 0.728686 | 0.42573 |  |  |  |  |
| 0.728893 | 0.425707 |  |  |  |  |
| 0.729123 | 0.425684 |  |  |  |  |
| 0.729353 | 0.425822 |  |  |  |  |
| 0.72956 | 0.425707 |  |  |  |  |
| 0.72979 | 0.425845 |  |  |  |  |
| 0.730043 | 0.425845 |  |  |  |  |
| 0.73025 | 0.426351 |  |  |  |  |
| 0.73048 | 0.426374 |  |  |  |  |
| 0.73071 | 0.426328 |  |  |  |  |
| 0.730917 | 0.426558 |  |  |  |  |
| 0.731147 | 0.426535 |  |  |  |  |
| 0.731377 | 0.426581 |  |  |  |  |
| 0.73163 | 0.426581 |  |  |  |  |
| 0.731837 | 0.426558 |  |  |  |  |
| 0.732067 | 0.426834 |  |  |  |  |
| 0.732297 | 0.427156 |  |  |  |  |
| 0.732504 | 0.426926 |  |  |  |  |
| 0.732734 | 0.426949 |  |  |  |  |
| 0.732964 | 0.426903 |  |  |  |  |
| 0.733194 | 0.427248 |  |  |  |  |
| 0.733424 | 0.427386 |  |  |  |  |
| 0.733654 | 0.427409 |  |  |  |  |
| 0.733861 | 0.427524 |  |  |  |  |
| 0.734091 | 0.427823 |  |  |  |  |
| 0.734321 | 0.427731 |  |  |  |  |
| 0.734551 | 0.427662 |  |  |  |  |
| 0.734781 | 0.427869 |  |  |  |  |
| 0.735011 | 0.42803 |  |  |  |  |
| 0.735218 | 0.428191 |  |  |  |  |
| 0.735448 | 0.427961 |  |  |  |  |
| 0.735678 | 0.428168 |  |  |  |  |
| 0.735885 | 0.42849 |  |  |  |  |
| 0.736138 | 0.428766 |  |  |  |  |
| 0.736368 | 0.42849 |  |  |  |  |
| 0.736575 | 0.428352 |  |  |  |  |
| 0.736805 | 0.428283 |  |  |  |  |
| 0.737035 | 0.428789 |  |  |  |  |
| 0.737265 | 0.42872 |  |  |  |  |
| 0.737472 | 0.428812 |  |  |  |  |
| 0.737725 | 0.428789 |  |  |  |  |
| 0.737955 | 0.429088 |  |  |  |  |
| 0.738162 | 0.429387 |  |  |  |  |
| 0.738392 | 0.429387 |  |  |  |  |
| 0.738622 | 0.42941 |  |  |  |  |
| 0.738829 | 0.429571 |  |  |  |  |
| 0.739059 | 0.429594 |  |  |  |  |
| 0.739312 | 0.429594 |  |  |  |  |
| 0.739519 | 0.429686 |  |  |  |  |
| 0.739749 | 0.429778 |  |  |  |  |
| 0.739979 | 0.430123 |  |  |  |  |
| 0.740186 | 0.430054 |  |  |  |  |
| 0.740416 | 0.42987 |  |  |  |  |
| 0.740646 | 0.430008 |  |  |  |  |
| 0.740876 | 0.430215 |  |  |  |  |
| 0.741106 | 0.430376 |  |  |  |  |
| 0.741336 | 0.430376 |  |  |  |  |
| 0.741543 | 0.43033 |  |  |  |  |
| 0.741773 | 0.430813 |  |  |  |  |
| 0.742003 | 0.430629 |  |  |  |  |
| 0.742233 | 0.430721 |  |  |  |  |
| 0.742463 | 0.430767 |  |  |  |  |
| 0.742693 | 0.430882 |  |  |  |  |
| 0.742923 | 0.431043 |  |  |  |  |
| 0.74313 | 0.431066 |  |  |  |  |
| 0.74336 | 0.431181 |  |  |  |  |
| 0.74359 | 0.431342 |  |  |  |  |
| 0.74382 | 0.431549 |  |  |  |  |
| 0.74405 | 0.431365 |  |  |  |  |
| 0.74428 | 0.431342 |  |  |  |  |
| 0.744487 | 0.43148 |  |  |  |  |
| 0.744717 | 0.431894 |  |  |  |  |
| 0.744947 | 0.431917 |  |  |  |  |
| 0.745154 | 0.43171 |  |  |  |  |
| 0.745407 | 0.432032 |  |  |  |  |
| 0.745637 | 0.431871 |  |  |  |  |
| 0.745844 | 0.431917 |  |  |  |  |
| 0.746074 | 0.432032 |  |  |  |  |
| 0.746304 | 0.432009 |  |  |  |  |
| 0.746511 | 0.4324 |  |  |  |  |
| 0.746741 | 0.432561 |  |  |  |  |
| 0.746994 | 0.432561 |  |  |  |  |
| 0.747201 | 0.432377 |  |  |  |  |
| 0.747431 | 0.432699 |  |  |  |  |
| 0.747661 | 0.432791 |  |  |  |  |
| 0.747891 | 0.43286 |  |  |  |  |
| 0.748098 | 0.432653 |  |  |  |  |
| 0.748328 | 0.432768 |  |  |  |  |
| 0.748558 | 0.433113 |  |  |  |  |
| 0.748788 | 0.433067 |  |  |  |  |
| 0.749018 | 0.433205 |  |  |  |  |
| 0.749248 | 0.433159 |  |  |  |  |
| 0.749455 | 0.433435 |  |  |  |  |
| 0.749685 | 0.433412 |  |  |  |  |
| 0.749915 | 0.433412 |  |  |  |  |
| 0.750145 | 0.433619 |  |  |  |  |
| 0.750375 | 0.433895 |  |  |  |  |
| 0.750605 | 0.433757 |  |  |  |  |
| 0.750835 | 0.433826 |  |  |  |  |
| 0.751042 | 0.433849 |  |  |  |  |
| 0.751272 | 0.43401 |  |  |  |  |
| 0.751502 | 0.434125 |  |  |  |  |
| 0.751732 | 0.434148 |  |  |  |  |
| 0.751962 | 0.434263 |  |  |  |  |
| 0.752169 | 0.434424 |  |  |  |  |
| 0.752399 | 0.434838 |  |  |  |  |
| 0.752629 | 0.434723 |  |  |  |  |
| 0.752859 | 0.435045 |  |  |  |  |
| 0.753089 | 0.43493 |  |  |  |  |
| 0.753319 | 0.434838 |  |  |  |  |
| 0.753549 | 0.434976 |  |  |  |  |
| 0.753756 | 0.435068 |  |  |  |  |
| 0.753986 | 0.43516 |  |  |  |  |
| 0.754216 | 0.43516 |  |  |  |  |
| 0.754423 | 0.435482 |  |  |  |  |
| 0.754653 | 0.435528 |  |  |  |  |
| 0.754906 | 0.435367 |  |  |  |  |
| 0.755113 | 0.435505 |  |  |  |  |
| 0.755343 | 0.435643 |  |  |  |  |
| 0.755573 | 0.435965 |  |  |  |  |
| 0.755803 | 0.435896 |  |  |  |  |
| 0.75601 | 0.435758 |  |  |  |  |
| 0.75624 | 0.436172 |  |  |  |  |
| 0.756493 | 0.436057 |  |  |  |  |
| 0.7567 | 0.436172 |  |  |  |  |
| 0.75693 | 0.436402 |  |  |  |  |
| 0.75716 | 0.436126 |  |  |  |  |
| 0.757367 | 0.436586 |  |  |  |  |
| 0.757597 | 0.436425 |  |  |  |  |
| 0.757827 | 0.436563 |  |  |  |  |
| 0.758057 | 0.436747 |  |  |  |  |
| 0.758287 | 0.436862 |  |  |  |  |
| 0.758517 | 0.436839 |  |  |  |  |
| 0.758724 | 0.436931 |  |  |  |  |
| 0.758954 | 0.436954 |  |  |  |  |
| 0.759184 | 0.437161 |  |  |  |  |
| 0.759414 | 0.437437 |  |  |  |  |
| 0.759644 | 0.437207 |  |  |  |  |
| 0.759874 | 0.437368 |  |  |  |  |
| 0.760081 | 0.437322 |  |  |  |  |
| 0.760311 | 0.437598 |  |  |  |  |
| 0.760541 | 0.437851 |  |  |  |  |
| 0.760748 | 0.437621 |  |  |  |  |
| 0.761001 | 0.437966 |  |  |  |  |
| 0.761231 | 0.437943 |  |  |  |  |
| 0.761461 | 0.438127 |  |  |  |  |
| 0.761668 | 0.438081 |  |  |  |  |
| 0.761898 | 0.437966 |  |  |  |  |
| 0.762128 | 0.438288 |  |  |  |  |
| 0.762335 | 0.438334 |  |  |  |  |
| 0.762588 | 0.438564 |  |  |  |  |
| 0.762818 | 0.438472 |  |  |  |  |
| 0.763025 | 0.438633 |  |  |  |  |
| 0.763255 | 0.438702 |  |  |  |  |
| 0.763485 | 0.438932 |  |  |  |  |
| 0.763692 | 0.438771 |  |  |  |  |
| 0.763922 | 0.439116 |  |  |  |  |
| 0.764175 | 0.439254 |  |  |  |  |
| 0.764382 | 0.439323 |  |  |  |  |
| 0.764612 | 0.439162 |  |  |  |  |
| 0.764842 | 0.439116 |  |  |  |  |
| 0.765049 | 0.439415 |  |  |  |  |
| 0.765279 | 0.439668 |  |  |  |  |
| 0.765509 | 0.439806 |  |  |  |  |
| 0.765739 | 0.439714 |  |  |  |  |
| 0.765969 | 0.440036 |  |  |  |  |
| 0.766199 | 0.43976 |  |  |  |  |
| 0.766429 | 0.440105 |  |  |  |  |
| 0.766636 | 0.439921 |  |  |  |  |
| 0.766866 | 0.44022 |  |  |  |  |
| 0.767096 | 0.440128 |  |  |  |  |
| 0.767326 | 0.440289 |  |  |  |  |
| 0.767556 | 0.440289 |  |  |  |  |
| 0.767786 | 0.440565 |  |  |  |  |
| 0.767993 | 0.440611 |  |  |  |  |
| 0.768223 | 0.440726 |  |  |  |  |
| 0.768453 | 0.440772 |  |  |  |  |
| 0.768683 | 0.440565 |  |  |  |  |
| 0.768913 | 0.440933 |  |  |  |  |
| 0.769143 | 0.440887 |  |  |  |  |
| 0.76935 | 0.441002 |  |  |  |  |
| 0.76958 | 0.44114 |  |  |  |  |
| 0.76981 | 0.441209 |  |  |  |  |
| 0.770017 | 0.441577 |  |  |  |  |
| 0.77027 | 0.441301 |  |  |  |  |
| 0.7705 | 0.441531 |  |  |  |  |
| 0.770707 | 0.441646 |  |  |  |  |
| 0.770937 | 0.441922 |  |  |  |  |
| 0.771167 | 0.441692 |  |  |  |  |
| 0.771397 | 0.441807 |  |  |  |  |
| 0.771604 | 0.441715 |  |  |  |  |
| 0.771834 | 0.442152 |  |  |  |  |
| 0.772087 | 0.442037 |  |  |  |  |
| 0.772294 | 0.442152 |  |  |  |  |
| 0.772524 | 0.442152 |  |  |  |  |
| 0.772754 | 0.442336 |  |  |  |  |
| 0.772961 | 0.442359 |  |  |  |  |
| 0.773191 | 0.442566 |  |  |  |  |
| 0.773421 | 0.442313 |  |  |  |  |
| 0.773651 | 0.44275 |  |  |  |  |
| 0.773881 | 0.442796 |  |  |  |  |
| 0.774111 | 0.442658 |  |  |  |  |
| 0.774318 | 0.442911 |  |  |  |  |
| 0.774548 | 0.442865 |  |  |  |  |
| 0.774778 | 0.443233 |  |  |  |  |
| 0.775008 | 0.443325 |  |  |  |  |
| 0.775238 | 0.443187 |  |  |  |  |
| 0.775468 | 0.443394 |  |  |  |  |
| 0.775698 | 0.443463 |  |  |  |  |
| 0.775905 | 0.443509 |  |  |  |  |
| 0.776135 | 0.44367 |  |  |  |  |
| 0.776365 | 0.443509 |  |  |  |  |
| 0.776595 | 0.443647 |  |  |  |  |
| 0.776825 | 0.444176 |  |  |  |  |
| 0.777055 | 0.443854 |  |  |  |  |
| 0.777262 | 0.444038 |  |  |  |  |
| 0.777492 | 0.443992 |  |  |  |  |
| 0.777722 | 0.444245 |  |  |  |  |
| 0.777929 | 0.444291 |  |  |  |  |
| 0.778182 | 0.44413 |  |  |  |  |
| 0.778412 | 0.444153 |  |  |  |  |
| 0.778619 | 0.444544 |  |  |  |  |
| 0.778849 | 0.444751 |  |  |  |  |
| 0.779079 | 0.444475 |  |  |  |  |
| 0.779286 | 0.444475 |  |  |  |  |
| 0.779516 | 0.444774 |  |  |  |  |
| 0.779769 | 0.444935 |  |  |  |  |
| 0.779999 | 0.444751 |  |  |  |  |
| 0.780206 | 0.444912 |  |  |  |  |
| 0.780436 | 0.44528 |  |  |  |  |
| 0.780666 | 0.445395 |  |  |  |  |
| 0.780873 | 0.445096 |  |  |  |  |
| 0.781103 | 0.445349 |  |  |  |  |
| 0.781356 | 0.445372 |  |  |  |  |
| 0.781563 | 0.445602 |  |  |  |  |
| 0.781793 | 0.445441 |  |  |  |  |
| 0.782023 | 0.445533 |  |  |  |  |
| 0.78223 | 0.445602 |  |  |  |  |
| 0.78246 | 0.445924 |  |  |  |  |
| 0.78269 | 0.445809 |  |  |  |  |
| 0.78292 | 0.445786 |  |  |  |  |
| 0.78315 | 0.446154 |  |  |  |  |
| 0.78338 | 0.446361 |  |  |  |  |
| 0.783587 | 0.446361 |  |  |  |  |
| 0.783817 | 0.446384 |  |  |  |  |
| 0.784047 | 0.446269 |  |  |  |  |
| 0.784277 | 0.446338 |  |  |  |  |
| 0.784507 | 0.446637 |  |  |  |  |
| 0.784737 | 0.446798 |  |  |  |  |
| 0.784944 | 0.446591 |  |  |  |  |
| 0.785174 | 0.446821 |  |  |  |  |
| 0.785404 | 0.447005 |  |  |  |  |
| 0.785634 | 0.44689 |  |  |  |  |
| 0.785864 | 0.446959 |  |  |  |  |
| 0.786094 | 0.447028 |  |  |  |  |
| 0.786324 | 0.447327 |  |  |  |  |
| 0.786531 | 0.447419 |  |  |  |  |
| 0.786761 | 0.447212 |  |  |  |  |
| 0.786991 | 0.447603 |  |  |  |  |
| 0.787198 | 0.447534 |  |  |  |  |
| 0.787451 | 0.44758 |  |  |  |  |
| 0.787681 | 0.447741 |  |  |  |  |
| 0.787888 | 0.447879 |  |  |  |  |
| 0.788118 | 0.447994 |  |  |  |  |
| 0.788348 | 0.44827 |  |  |  |  |
| 0.788555 | 0.448132 |  |  |  |  |
| 0.788785 | 0.448178 |  |  |  |  |
| 0.789038 | 0.448063 |  |  |  |  |
| 0.789245 | 0.448454 |  |  |  |  |
| 0.789475 | 0.4485 |  |  |  |  |
| 0.789705 | 0.448247 |  |  |  |  |
| 0.789912 | 0.448454 |  |  |  |  |
| 0.790142 | 0.448684 |  |  |  |  |
| 0.790372 | 0.448799 |  |  |  |  |
| 0.790602 | 0.448799 |  |  |  |  |
| 0.790832 | 0.448776 |  |  |  |  |
| 0.791062 | 0.449052 |  |  |  |  |
| 0.791292 | 0.449328 |  |  |  |  |
| 0.791499 | 0.449167 |  |  |  |  |
| 0.791729 | 0.44896 |  |  |  |  |
| 0.791959 | 0.44942 |  |  |  |  |
| 0.792189 | 0.449489 |  |  |  |  |
| 0.792419 | 0.449604 |  |  |  |  |
| 0.792649 | 0.449374 |  |  |  |  |
| 0.792856 | 0.449673 |  |  |  |  |
| 0.793086 | 0.449857 |  |  |  |  |
| 0.793316 | 0.44988 |  |  |  |  |
| 0.793546 | 0.44988 |  |  |  |  |
| 0.793776 | 0.449903 |  |  |  |  |
| 0.794006 | 0.450156 |  |  |  |  |
| 0.794213 | 0.450294 |  |  |  |  |
| 0.794443 | 0.450018 |  |  |  |  |
| 0.794673 | 0.450202 |  |  |  |  |
| 0.79488 | 0.450179 |  |  |  |  |
| 0.795133 | 0.450616 |  |  |  |  |
| 0.795363 | 0.450662 |  |  |  |  |
| 0.79557 | 0.450639 |  |  |  |  |
| 0.7958 | 0.450616 |  |  |  |  |
| 0.79603 | 0.451007 |  |  |  |  |
| 0.79626 | 0.451076 |  |  |  |  |
| 0.796467 | 0.450961 |  |  |  |  |
| 0.796697 | 0.45103 |  |  |  |  |
| 0.79695 | 0.451168 |  |  |  |  |
| 0.797157 | 0.451421 |  |  |  |  |
| 0.797387 | 0.45126 |  |  |  |  |
| 0.797617 | 0.45126 |  |  |  |  |
| 0.797824 | 0.451628 |  |  |  |  |
| 0.798054 | 0.451605 |  |  |  |  |
| 0.798284 | 0.451398 |  |  |  |  |
| 0.798514 | 0.451467 |  |  |  |  |
| 0.798744 | 0.451812 |  |  |  |  |
| 0.798974 | 0.45195 |  |  |  |  |
| 0.799204 | 0.451881 |  |  |  |  |
| 0.799411 | 0.451789 |  |  |  |  |
| 0.799641 | 0.452019 |  |  |  |  |
| 0.799871 | 0.452249 |  |  |  |  |
| 0.800101 | 0.452364 |  |  |  |  |
| 0.800331 | 0.452134 |  |  |  |  |
| 0.800538 | 0.452226 |  |  |  |  |
| 0.800768 | 0.452686 |  |  |  |  |
| 0.800998 | 0.452709 |  |  |  |  |
| 0.801228 | 0.452502 |  |  |  |  |
| 0.801458 | 0.452663 |  |  |  |  |
| 0.801688 | 0.452847 |  |  |  |  |
| 0.801918 | 0.45287 |  |  |  |  |
| 0.802125 | 0.453054 |  |  |  |  |
| 0.802355 | 0.452962 |  |  |  |  |
| 0.802585 | 0.453031 |  |  |  |  |
| 0.802792 | 0.453192 |  |  |  |  |
| 0.803045 | 0.453445 |  |  |  |  |
| 0.803275 | 0.453445 |  |  |  |  |
| 0.803482 | 0.453399 |  |  |  |  |
| 0.803712 | 0.453445 |  |  |  |  |
| 0.803942 | 0.453514 |  |  |  |  |
| 0.804172 | 0.453537 |  |  |  |  |
| 0.804379 | 0.453836 |  |  |  |  |
| 0.804632 | 0.454135 |  |  |  |  |
| 0.804862 | 0.453951 |  |  |  |  |
| 0.805069 | 0.454204 |  |  |  |  |
| 0.805299 | 0.454158 |  |  |  |  |
| 0.805529 | 0.454204 |  |  |  |  |
| 0.805736 | 0.45425 |  |  |  |  |
| 0.805966 | 0.454503 |  |  |  |  |
| 0.806219 | 0.454273 |  |  |  |  |
| 0.806426 | 0.454595 |  |  |  |  |
| 0.806656 | 0.454756 |  |  |  |  |
| 0.806886 | 0.454986 |  |  |  |  |
| 0.807093 | 0.454848 |  |  |  |  |
| 0.807323 | 0.454572 |  |  |  |  |
| 0.807553 | 0.45517 |  |  |  |  |
| 0.807783 | 0.45517 |  |  |  |  |
| 0.808013 | 0.455124 |  |  |  |  |
| 0.808243 | 0.455239 |  |  |  |  |
| 0.80845 | 0.455561 |  |  |  |  |
| 0.80868 | 0.455469 |  |  |  |  |
| 0.80891 | 0.455446 |  |  |  |  |
| 0.80914 | 0.455377 |  |  |  |  |
| 0.80937 | 0.45563 |  |  |  |  |
| 0.8096 | 0.455561 |  |  |  |  |
| 0.80983 | 0.455975 |  |  |  |  |
| 0.810037 | 0.455952 |  |  |  |  |
| 0.810267 | 0.455768 |  |  |  |  |
| 0.810497 | 0.45609 |  |  |  |  |
| 0.810727 | 0.456205 |  |  |  |  |
| 0.810957 | 0.455998 |  |  |  |  |
| 0.811187 | 0.456159 |  |  |  |  |
| 0.811394 | 0.456573 |  |  |  |  |
| 0.811624 | 0.456596 |  |  |  |  |
| 0.811854 | 0.456458 |  |  |  |  |
| 0.812061 | 0.456481 |  |  |  |  |
| 0.812314 | 0.456688 |  |  |  |  |
| 0.812544 | 0.45678 |  |  |  |  |
| 0.812751 | 0.456734 |  |  |  |  |
| 0.812981 | 0.456872 |  |  |  |  |
| 0.813211 | 0.456895 |  |  |  |  |
| 0.813418 | 0.456987 |  |  |  |  |
| 0.813648 | 0.457148 |  |  |  |  |
| 0.813878 | 0.457102 |  |  |  |  |
| 0.814108 | 0.457286 |  |  |  |  |
| 0.814338 | 0.457539 |  |  |  |  |
| 0.814568 | 0.457516 |  |  |  |  |
| 0.814798 | 0.457585 |  |  |  |  |
| 0.815005 | 0.457355 |  |  |  |  |
| 0.815235 | 0.457723 |  |  |  |  |
| 0.815465 | 0.457815 |  |  |  |  |
| 0.815695 | 0.457976 |  |  |  |  |
| 0.815925 | 0.457907 |  |  |  |  |
| 0.816155 | 0.457976 |  |  |  |  |
| 0.816362 | 0.45816 |  |  |  |  |
| 0.816592 | 0.458459 |  |  |  |  |
| 0.816822 | 0.458321 |  |  |  |  |
| 0.817052 | 0.458505 |  |  |  |  |
| 0.817282 | 0.458735 |  |  |  |  |
| 0.817512 | 0.458344 |  |  |  |  |
| 0.817742 | 0.458528 |  |  |  |  |
| 0.817949 | 0.458666 |  |  |  |  |
| 0.818179 | 0.459149 |  |  |  |  |
| 0.818409 | 0.459011 |  |  |  |  |
| 0.818639 | 0.458735 |  |  |  |  |
| 0.818869 | 0.458873 |  |  |  |  |
| 0.819076 | 0.459126 |  |  |  |  |
| 0.819306 | 0.459126 |  |  |  |  |
| 0.819536 | 0.45908 |  |  |  |  |
| 0.819766 | 0.459264 |  |  |  |  |
| 0.819973 | 0.45931 |  |  |  |  |
| 0.820226 | 0.459793 |  |  |  |  |
| 0.820456 | 0.459471 |  |  |  |  |
| 0.820663 | 0.45977 |  |  |  |  |
| 0.820893 | 0.459632 |  |  |  |  |
| 0.821123 | 0.459908 |  |  |  |  |
| 0.82133 | 0.46 |  |  |  |  |
| 0.82156 | 0.459977 |  |  |  |  |
| 0.821813 | 0.459839 |  |  |  |  |
| 0.82202 | 0.460345 |  |  |  |  |
| 0.82225 | 0.46023 |  |  |  |  |
| 0.82248 | 0.460207 |  |  |  |  |
| 0.822687 | 0.460253 |  |  |  |  |
| 0.822917 | 0.460253 |  |  |  |  |
| 0.823147 | 0.460598 |  |  |  |  |
| 0.8234 | 0.460621 |  |  |  |  |
| 0.823607 | 0.460828 |  |  |  |  |
| 0.823837 | 0.460713 |  |  |  |  |
| 0.824044 | 0.460897 |  |  |  |  |
| 0.824274 | 0.461012 |  |  |  |  |
| 0.824504 | 0.460989 |  |  |  |  |
| 0.824734 | 0.46115 |  |  |  |  |
| 0.824964 | 0.461334 |  |  |  |  |
| 0.825194 | 0.46115 |  |  |  |  |
| 0.825424 | 0.461242 |  |  |  |  |
| 0.825631 | 0.461196 |  |  |  |  |
| 0.825861 | 0.461748 |  |  |  |  |
| 0.826091 | 0.461886 |  |  |  |  |
| 0.826321 | 0.461909 |  |  |  |  |
| 0.826551 | 0.461518 |  |  |  |  |
| 0.826781 | 0.461886 |  |  |  |  |
| 0.826988 | 0.462116 |  |  |  |  |
| 0.827218 | 0.462024 |  |  |  |  |
| 0.827448 | 0.462093 |  |  |  |  |
| 0.827655 | 0.462185 |  |  |  |  |
| 0.827908 | 0.462392 |  |  |  |  |
| 0.828138 | 0.462208 |  |  |  |  |
| 0.828368 | 0.462415 |  |  |  |  |
| 0.828575 | 0.462576 |  |  |  |  |
| 0.828805 | 0.462898 |  |  |  |  |
| 0.829012 | 0.462898 |  |  |  |  |
| 0.829242 | 0.462668 |  |  |  |  |
| 0.829495 | 0.462668 |  |  |  |  |
| 0.829725 | 0.463243 |  |  |  |  |
| 0.829932 | 0.463105 |  |  |  |  |
| 0.830162 | 0.463082 |  |  |  |  |
| 0.830392 | 0.463289 |  |  |  |  |
| 0.830599 | 0.463266 |  |  |  |  |
| 0.830829 | 0.463312 |  |  |  |  |
| 0.831082 | 0.463335 |  |  |  |  |
| 0.831289 | 0.463381 |  |  |  |  |
| 0.831519 | 0.463726 |  |  |  |  |
| 0.831749 | 0.46368 |  |  |  |  |
| 0.831956 | 0.46391 |  |  |  |  |
| 0.832186 | 0.463772 |  |  |  |  |
| 0.832416 | 0.463772 |  |  |  |  |
| 0.832646 | 0.464094 |  |  |  |  |
| 0.832876 | 0.463933 |  |  |  |  |
| 0.833106 | 0.463933 |  |  |  |  |
| 0.833313 | 0.464048 |  |  |  |  |
| 0.833543 | 0.464186 |  |  |  |  |
| 0.833773 | 0.464278 |  |  |  |  |
| 0.834003 | 0.4646 |  |  |  |  |
| 0.834233 | 0.464186 |  |  |  |  |
| 0.834463 | 0.464416 |  |  |  |  |
| 0.834693 | 0.464968 |  |  |  |  |
| 0.8349 | 0.464761 |  |  |  |  |
| 0.83513 | 0.464922 |  |  |  |  |
| 0.83536 | 0.464807 |  |  |  |  |
| 0.83559 | 0.464991 |  |  |  |  |
| 0.83582 | 0.465175 |  |  |  |  |
| 0.83605 | 0.464991 |  |  |  |  |
| 0.836257 | 0.465037 |  |  |  |  |
| 0.836487 | 0.465129 |  |  |  |  |
| 0.836717 | 0.465313 |  |  |  |  |
| 0.836924 | 0.465474 |  |  |  |  |
| 0.837177 | 0.465543 |  |  |  |  |
| 0.837407 | 0.465474 |  |  |  |  |
| 0.837614 | 0.465796 |  |  |  |  |
| 0.837844 | 0.465911 |  |  |  |  |
| 0.838074 | 0.465796 |  |  |  |  |
| 0.838281 | 0.465934 |  |  |  |  |
| 0.838511 | 0.466026 |  |  |  |  |
| 0.838741 | 0.466095 |  |  |  |  |
| 0.838994 | 0.466095 |  |  |  |  |
| 0.839201 | 0.46598 |  |  |  |  |
| 0.839431 | 0.46621 |  |  |  |  |
| 0.839661 | 0.466118 |  |  |  |  |
| 0.839868 | 0.466417 |  |  |  |  |
| 0.840098 | 0.466279 |  |  |  |  |
| 0.840328 | 0.466601 |  |  |  |  |
| 0.840558 | 0.466923 |  |  |  |  |
| 0.840788 | 0.466831 |  |  |  |  |
| 0.841018 | 0.466601 |  |  |  |  |
| 0.841225 | 0.4669 |  |  |  |  |
| 0.841455 | 0.467061 |  |  |  |  |
| 0.841685 | 0.467107 |  |  |  |  |
| 0.841915 | 0.467015 |  |  |  |  |
| 0.842145 | 0.467107 |  |  |  |  |
| 0.842375 | 0.467268 |  |  |  |  |
| 0.842582 | 0.467176 |  |  |  |  |
| 0.842812 | 0.467222 |  |  |  |  |
| 0.843042 | 0.46736 |  |  |  |  |
| 0.843249 | 0.467314 |  |  |  |  |
| 0.843502 | 0.467659 |  |  |  |  |
| 0.843732 | 0.46759 |  |  |  |  |
| 0.843939 | 0.46759 |  |  |  |  |
| 0.844169 | 0.467889 |  |  |  |  |
| 0.844399 | 0.467889 |  |  |  |  |
| 0.844629 | 0.468004 |  |  |  |  |
| 0.844836 | 0.467774 |  |  |  |  |
| 0.845089 | 0.467981 |  |  |  |  |
| 0.845319 | 0.468257 |  |  |  |  |
| 0.845526 | 0.468326 |  |  |  |  |
| 0.845756 | 0.467889 |  |  |  |  |
| 0.845986 | 0.468188 |  |  |  |  |
| 0.846193 | 0.468395 |  |  |  |  |
| 0.846423 | 0.468441 |  |  |  |  |
| 0.846676 | 0.468533 |  |  |  |  |
| 0.846883 | 0.468602 |  |  |  |  |
| 0.847113 | 0.468717 |  |  |  |  |
| 0.847343 | 0.468625 |  |  |  |  |
| 0.847573 | 0.468786 |  |  |  |  |
| 0.84778 | 0.468993 |  |  |  |  |
| 0.84801 | 0.468924 |  |  |  |  |
| 0.848263 | 0.469292 |  |  |  |  |
| 0.84847 | 0.469338 |  |  |  |  |
| 0.8487 | 0.468993 |  |  |  |  |
| 0.84893 | 0.469154 |  |  |  |  |
| 0.849137 | 0.469706 |  |  |  |  |
| 0.849367 | 0.469798 |  |  |  |  |
| 0.849597 | 0.46943 |  |  |  |  |
| 0.849827 | 0.469752 |  |  |  |  |
| 0.850057 | 0.46966 |  |  |  |  |
| 0.850287 | 0.46989 |  |  |  |  |
| 0.850494 | 0.469752 |  |  |  |  |
| 0.850724 | 0.469936 |  |  |  |  |
| 0.850954 | 0.469936 |  |  |  |  |
| 0.851184 | 0.470281 |  |  |  |  |
| 0.851414 | 0.470166 |  |  |  |  |
| 0.851644 | 0.469959 |  |  |  |  |
| 0.851851 | 0.470442 |  |  |  |  |
| 0.852081 | 0.470626 |  |  |  |  |
| 0.852311 | 0.470534 |  |  |  |  |
| 0.852541 | 0.470557 |  |  |  |  |
| 0.852771 | 0.470373 |  |  |  |  |
| 0.853001 | 0.470833 |  |  |  |  |
| 0.853231 | 0.470902 |  |  |  |  |
| 0.853438 | 0.471017 |  |  |  |  |
| 0.853668 | 0.470879 |  |  |  |  |
| 0.853898 | 0.471132 |  |  |  |  |
| 0.854105 | 0.470994 |  |  |  |  |
| 0.854358 | 0.471385 |  |  |  |  |
| 0.854588 | 0.471063 |  |  |  |  |
| 0.854795 | 0.471201 |  |  |  |  |
| 0.855025 | 0.471592 |  |  |  |  |
| 0.855255 | 0.471569 |  |  |  |  |
| 0.855462 | 0.471201 |  |  |  |  |
| 0.855692 | 0.4715 |  |  |  |  |
| 0.855922 | 0.471707 |  |  |  |  |
| 0.856152 | 0.47196 |  |  |  |  |
| 0.856382 | 0.471684 |  |  |  |  |
| 0.856612 | 0.47173 |  |  |  |  |
| 0.856819 | 0.471891 |  |  |  |  |
| 0.857049 | 0.472397 |  |  |  |  |
| 0.857279 | 0.472075 |  |  |  |  |
| 0.857509 | 0.472075 |  |  |  |  |
| 0.857739 | 0.472397 |  |  |  |  |
| 0.857969 | 0.472627 |  |  |  |  |
| 0.858199 | 0.472374 |  |  |  |  |
| 0.858406 | 0.472466 |  |  |  |  |
| 0.858636 | 0.472535 |  |  |  |  |
| 0.858866 | 0.472788 |  |  |  |  |
| 0.859096 | 0.472811 |  |  |  |  |
| 0.859326 | 0.472788 |  |  |  |  |
| 0.859556 | 0.473018 |  |  |  |  |
| 0.859763 | 0.473133 |  |  |  |  |
| 0.859993 | 0.473432 |  |  |  |  |
| 0.860223 | 0.473087 |  |  |  |  |
| 0.860453 | 0.472903 |  |  |  |  |
| 0.860683 | 0.473409 |  |  |  |  |
| 0.860913 | 0.473455 |  |  |  |  |
| 0.86112 | 0.473271 |  |  |  |  |
| 0.86135 | 0.473524 |  |  |  |  |
| 0.86158 | 0.473432 |  |  |  |  |
| 0.861787 | 0.473708 |  |  |  |  |
| 0.862017 | 0.473823 |  |  |  |  |
| 0.86227 | 0.473708 |  |  |  |  |
| 0.862477 | 0.473961 |  |  |  |  |
| 0.862707 | 0.474191 |  |  |  |  |
| 0.862937 | 0.473823 |  |  |  |  |
| 0.863167 | 0.474076 |  |  |  |  |
| 0.863374 | 0.474283 |  |  |  |  |
| 0.863604 | 0.474329 |  |  |  |  |
| 0.863857 | 0.474513 |  |  |  |  |
| 0.864064 | 0.474697 |  |  |  |  |
| 0.864294 | 0.474605 |  |  |  |  |
| 0.864524 | 0.474697 |  |  |  |  |
| 0.864731 | 0.474858 |  |  |  |  |
| 0.864961 | 0.474858 |  |  |  |  |
| 0.865191 | 0.474835 |  |  |  |  |
| 0.865421 | 0.474559 |  |  |  |  |
| 0.865651 | 0.47518 |  |  |  |  |
| 0.865881 | 0.475157 |  |  |  |  |
| 0.866111 | 0.475157 |  |  |  |  |
| 0.866318 | 0.475019 |  |  |  |  |
| 0.866548 | 0.47541 |  |  |  |  |
| 0.866778 | 0.475341 |  |  |  |  |
| 0.867008 | 0.475456 |  |  |  |  |
| 0.867238 | 0.475456 |  |  |  |  |
| 0.867445 | 0.475663 |  |  |  |  |
| 0.867675 | 0.475732 |  |  |  |  |
| 0.867905 | 0.475801 |  |  |  |  |
| 0.868135 | 0.475709 |  |  |  |  |
| 0.868365 | 0.475962 |  |  |  |  |
| 0.868595 | 0.476169 |  |  |  |  |
| 0.868825 | 0.475939 |  |  |  |  |
| 0.869032 | 0.475962 |  |  |  |  |
| 0.869262 | 0.47633 |  |  |  |  |
| 0.869492 | 0.476307 |  |  |  |  |
| 0.869699 | 0.476606 |  |  |  |  |
| 0.869952 | 0.476606 |  |  |  |  |
| 0.870182 | 0.476491 |  |  |  |  |
| 0.870389 | 0.476767 |  |  |  |  |
| 0.870619 | 0.476928 |  |  |  |  |
| 0.870849 | 0.476629 |  |  |  |  |
| 0.871056 | 0.476652 |  |  |  |  |
| 0.871286 | 0.477112 |  |  |  |  |
| 0.871539 | 0.476882 |  |  |  |  |
| 0.871769 | 0.477112 |  |  |  |  |
| 0.871976 | 0.476928 |  |  |  |  |
| 0.872206 | 0.477066 |  |  |  |  |
| 0.872413 | 0.477319 |  |  |  |  |
| 0.872643 | 0.477158 |  |  |  |  |
| 0.872873 | 0.477503 |  |  |  |  |
| 0.873103 | 0.477434 |  |  |  |  |
| 0.873333 | 0.477526 |  |  |  |  |
| 0.873563 | 0.477733 |  |  |  |  |
| 0.873793 | 0.477434 |  |  |  |  |
| 0.874 | 0.477687 |  |  |  |  |
| 0.87423 | 0.477894 |  |  |  |  |
| 0.87446 | 0.477871 |  |  |  |  |
| 0.87469 | 0.478032 |  |  |  |  |
| 0.87492 | 0.477917 |  |  |  |  |
| 0.87515 | 0.477871 |  |  |  |  |
| 0.875357 | 0.478285 |  |  |  |  |
| 0.875587 | 0.478101 |  |  |  |  |
| 0.875817 | 0.478492 |  |  |  |  |
| 0.876047 | 0.478285 |  |  |  |  |
| 0.876277 | 0.4784 |  |  |  |  |
| 0.876507 | 0.478607 |  |  |  |  |
| 0.876737 | 0.478492 |  |  |  |  |
| 0.876944 | 0.478423 |  |  |  |  |
| 0.877174 | 0.47886 |  |  |  |  |
| 0.877404 | 0.478998 |  |  |  |  |
| 0.877634 | 0.478722 |  |  |  |  |
| 0.877864 | 0.478561 |  |  |  |  |
| 0.878094 | 0.478768 |  |  |  |  |
| 0.878301 | 0.479113 |  |  |  |  |
| 0.878531 | 0.479044 |  |  |  |  |
| 0.878761 | 0.47886 |  |  |  |  |
| 0.878968 | 0.47886 |  |  |  |  |
| 0.879221 | 0.479343 |  |  |  |  |
| 0.879451 | 0.479527 |  |  |  |  |
| 0.879658 | 0.479573 |  |  |  |  |
| 0.879888 | 0.479366 |  |  |  |  |
| 0.880118 | 0.479527 |  |  |  |  |
| 0.880325 | 0.479872 |  |  |  |  |
| 0.880555 | 0.479481 |  |  |  |  |
| 0.880785 | 0.47978 |  |  |  |  |
| 0.881015 | 0.479596 |  |  |  |  |
| 0.881245 | 0.479711 |  |  |  |  |
| 0.881475 | 0.479918 |  |  |  |  |
| 0.881682 | 0.479849 |  |  |  |  |
| 0.881912 | 0.479964 |  |  |  |  |
| 0.882142 | 0.480148 |  |  |  |  |
| 0.882372 | 0.480286 |  |  |  |  |
| 0.882602 | 0.480171 |  |  |  |  |
| 0.882832 | 0.480194 |  |  |  |  |
| 0.883062 | 0.480516 |  |  |  |  |
| 0.883269 | 0.480493 |  |  |  |  |
| 0.883499 | 0.480539 |  |  |  |  |
| 0.883729 | 0.480378 |  |  |  |  |
| 0.883959 | 0.480677 |  |  |  |  |
| 0.884189 | 0.4807 |  |  |  |  |
| 0.884419 | 0.480585 |  |  |  |  |
| 0.884626 | 0.481022 |  |  |  |  |
| 0.884856 | 0.481068 |  |  |  |  |
| 0.885086 | 0.481137 |  |  |  |  |
| 0.885293 | 0.481298 |  |  |  |  |
| 0.885546 | 0.481114 |  |  |  |  |
| 0.885776 | 0.481298 |  |  |  |  |
| 0.885983 | 0.481321 |  |  |  |  |
| 0.886213 | 0.481298 |  |  |  |  |
| 0.886443 | 0.481413 |  |  |  |  |
| 0.88665 | 0.481367 |  |  |  |  |
| 0.88688 | 0.481689 |  |  |  |  |
| 0.887133 | 0.481942 |  |  |  |  |
| 0.887363 | 0.48185 |  |  |  |  |
| 0.88757 | 0.481873 |  |  |  |  |
| 0.8878 | 0.48185 |  |  |  |  |
| 0.88803 | 0.482241 |  |  |  |  |
| 0.888237 | 0.482103 |  |  |  |  |
| 0.888467 | 0.482057 |  |  |  |  |
| 0.88872 | 0.482264 |  |  |  |  |
| 0.888927 | 0.48231 |  |  |  |  |
| 0.889157 | 0.482333 |  |  |  |  |
| 0.889387 | 0.482333 |  |  |  |  |
| 0.889594 | 0.482264 |  |  |  |  |
| 0.889824 | 0.482678 |  |  |  |  |
| 0.890054 | 0.48277 |  |  |  |  |
| 0.890307 | 0.48277 |  |  |  |  |
| 0.890514 | 0.482839 |  |  |  |  |
| 0.890744 | 0.482862 |  |  |  |  |
| 0.890951 | 0.482977 |  |  |  |  |
| 0.891181 | 0.482954 |  |  |  |  |
| 0.891411 | 0.482701 |  |  |  |  |
| 0.891641 | 0.483207 |  |  |  |  |
| 0.891871 | 0.483184 |  |  |  |  |
| 0.892101 | 0.48323 |  |  |  |  |
| 0.892308 | 0.483161 |  |  |  |  |
| 0.892538 | 0.483161 |  |  |  |  |
| 0.892768 | 0.483391 |  |  |  |  |
| 0.892998 | 0.483506 |  |  |  |  |
| 0.893228 | 0.483437 |  |  |  |  |
| 0.893458 | 0.483644 |  |  |  |  |
| 0.893688 | 0.483759 |  |  |  |  |
| 0.893895 | 0.484035 |  |  |  |  |
| 0.894125 | 0.483782 |  |  |  |  |
| 0.894355 | 0.48369 |  |  |  |  |
| 0.894562 | 0.483966 |  |  |  |  |
| 0.894815 | 0.484104 |  |  |  |  |
| 0.895045 | 0.483966 |  |  |  |  |
| 0.895252 | 0.484196 |  |  |  |  |
| 0.895482 | 0.484334 |  |  |  |  |
| 0.895712 | 0.484403 |  |  |  |  |
| 0.895942 | 0.484449 |  |  |  |  |
| 0.896149 | 0.484357 |  |  |  |  |
| 0.896402 | 0.484426 |  |  |  |  |
| 0.896632 | 0.484725 |  |  |  |  |
| 0.896839 | 0.484794 |  |  |  |  |
| 0.897069 | 0.484702 |  |  |  |  |
| 0.897276 | 0.484794 |  |  |  |  |
| 0.897506 | 0.484886 |  |  |  |  |
| 0.897736 | 0.485001 |  |  |  |  |
| 0.897989 | 0.485346 |  |  |  |  |
| 0.898196 | 0.484886 |  |  |  |  |
| 0.898426 | 0.485024 |  |  |  |  |
| 0.898656 | 0.485231 |  |  |  |  |
| 0.898863 | 0.485277 |  |  |  |  |
| 0.899093 | 0.485162 |  |  |  |  |
| 0.899323 | 0.485277 |  |  |  |  |
| 0.899553 | 0.485898 |  |  |  |  |
| 0.899783 | 0.485461 |  |  |  |  |
| 0.900013 | 0.48553 |  |  |  |  |
| 0.90022 | 0.485415 |  |  |  |  |
| 0.90045 | 0.485852 |  |  |  |  |
| 0.90068 | 0.485714 |  |  |  |  |
| 0.90091 | 0.485806 |  |  |  |  |
| 0.90114 | 0.485898 |  |  |  |  |
| 0.90137 | 0.486151 |  |  |  |  |
| 0.9016 | 0.485967 |  |  |  |  |
| 0.901807 | 0.486059 |  |  |  |  |
| 0.902037 | 0.486197 |  |  |  |  |
| 0.902267 | 0.486312 |  |  |  |  |
| 0.902497 | 0.486312 |  |  |  |  |
| 0.902727 | 0.486404 |  |  |  |  |
| 0.902957 | 0.486519 |  |  |  |  |
| 0.903164 | 0.486657 |  |  |  |  |
| 0.903394 | 0.486565 |  |  |  |  |
| 0.903624 | 0.486864 |  |  |  |  |
| 0.903831 | 0.48668 |  |  |  |  |
| 0.904061 | 0.487025 |  |  |  |  |
| 0.904314 | 0.48714 |  |  |  |  |
| 0.904521 | 0.487094 |  |  |  |  |
| 0.904751 | 0.486887 |  |  |  |  |
| 0.904981 | 0.487025 |  |  |  |  |
| 0.905188 | 0.48737 |  |  |  |  |
| 0.905418 | 0.487462 |  |  |  |  |
| 0.905648 | 0.487508 |  |  |  |  |
| 0.905878 | 0.487163 |  |  |  |  |
| 0.906108 | 0.487416 |  |  |  |  |
| 0.906338 | 0.487692 |  |  |  |  |
| 0.906568 | 0.48783 |  |  |  |  |
| 0.906775 | 0.487761 |  |  |  |  |
| 0.907005 | 0.487738 |  |  |  |  |
| 0.907235 | 0.487991 |  |  |  |  |
| 0.907465 | 0.487922 |  |  |  |  |
| 0.907695 | 0.488083 |  |  |  |  |
| 0.907925 | 0.487991 |  |  |  |  |
| 0.908132 | 0.488267 |  |  |  |  |
| 0.908362 | 0.488152 |  |  |  |  |
| 0.908592 | 0.488221 |  |  |  |  |
| 0.908822 | 0.488221 |  |  |  |  |
| 0.909052 | 0.488405 |  |  |  |  |
| 0.909282 | 0.488589 |  |  |  |  |
| 0.909489 | 0.48875 |  |  |  |  |
| 0.909719 | 0.488612 |  |  |  |  |
| 0.909949 | 0.488796 |  |  |  |  |
| 0.910156 | 0.488957 |  |  |  |  |
| 0.910409 | 0.488727 |  |  |  |  |
| 0.910639 | 0.488658 |  |  |  |  |
| 0.910846 | 0.488704 |  |  |  |  |
| 0.911076 | 0.489187 |  |  |  |  |
| 0.911306 | 0.48921 |  |  |  |  |
| 0.911536 | 0.489141 |  |  |  |  |
| 0.911743 | 0.488888 |  |  |  |  |
| 0.911996 | 0.489072 |  |  |  |  |
| 0.912226 | 0.489187 |  |  |  |  |
| 0.912433 | 0.489394 |  |  |  |  |
| 0.912663 | 0.489233 |  |  |  |  |
| 0.912893 | 0.489486 |  |  |  |  |
| 0.9131 | 0.489716 |  |  |  |  |
| 0.91333 | 0.489647 |  |  |  |  |
| 0.913583 | 0.489578 |  |  |  |  |
| 0.91379 | 0.489693 |  |  |  |  |
| 0.91402 | 0.489877 |  |  |  |  |
| 0.91425 | 0.490038 |  |  |  |  |
| 0.91448 | 0.489946 |  |  |  |  |
| 0.914687 | 0.489946 |  |  |  |  |
| 0.914917 | 0.490176 |  |  |  |  |
| 0.915147 | 0.490176 |  |  |  |  |
| 0.915377 | 0.490245 |  |  |  |  |
| 0.915607 | 0.490383 |  |  |  |  |
| 0.915814 | 0.490383 |  |  |  |  |
| 0.916044 | 0.490291 |  |  |  |  |
| 0.916274 | 0.490544 |  |  |  |  |
| 0.916504 | 0.490544 |  |  |  |  |
| 0.916734 | 0.490682 |  |  |  |  |
| 0.916964 | 0.491096 |  |  |  |  |
| 0.917194 | 0.490728 |  |  |  |  |
| 0.917401 | 0.490843 |  |  |  |  |
| 0.917631 | 0.490866 |  |  |  |  |
| 0.917861 | 0.491211 |  |  |  |  |
| 0.918091 | 0.49105 |  |  |  |  |
| 0.918321 | 0.491211 |  |  |  |  |
| 0.918551 | 0.49105 |  |  |  |  |
| 0.918758 | 0.491556 |  |  |  |  |
| 0.918988 | 0.491625 |  |  |  |  |
| 0.919218 | 0.491096 |  |  |  |  |
| 0.919425 | 0.491326 |  |  |  |  |
| 0.919678 | 0.491487 |  |  |  |  |
| 0.919908 | 0.491487 |  |  |  |  |
| 0.920138 | 0.491809 |  |  |  |  |
| 0.920345 | 0.491533 |  |  |  |  |
| 0.920575 | 0.491901 |  |  |  |  |
| 0.920782 | 0.492085 |  |  |  |  |
| 0.921012 | 0.491993 |  |  |  |  |
| 0.921265 | 0.492108 |  |  |  |  |
| 0.921472 | 0.4922 |  |  |  |  |
| 0.921702 | 0.492384 |  |  |  |  |
| 0.921932 | 0.492292 |  |  |  |  |
| 0.922162 | 0.492292 |  |  |  |  |
| 0.922369 | 0.49243 |  |  |  |  |
| 0.922599 | 0.492476 |  |  |  |  |
| 0.922829 | 0.492706 |  |  |  |  |
| 0.923059 | 0.492407 |  |  |  |  |
| 0.923289 | 0.49266 |  |  |  |  |
| 0.923519 | 0.492959 |  |  |  |  |
| 0.923726 | 0.492867 |  |  |  |  |
| 0.923956 | 0.492959 |  |  |  |  |
| 0.924186 | 0.492798 |  |  |  |  |
| 0.924416 | 0.493028 |  |  |  |  |
| 0.924646 | 0.493074 |  |  |  |  |
| 0.924876 | 0.493281 |  |  |  |  |
| 0.925106 | 0.493235 |  |  |  |  |
| 0.925313 | 0.49335 |  |  |  |  |
| 0.925543 | 0.49335 |  |  |  |  |
| 0.925773 | 0.493327 |  |  |  |  |
| 0.926003 | 0.49335 |  |  |  |  |
| 0.926233 | 0.493787 |  |  |  |  |
| 0.926463 | 0.493649 |  |  |  |  |
| 0.92667 | 0.493603 |  |  |  |  |
| 0.9269 | 0.493833 |  |  |  |  |
| 0.92713 | 0.493948 |  |  |  |  |
| 0.927337 | 0.493902 |  |  |  |  |
| 0.92759 | 0.494155 |  |  |  |  |
| 0.92782 | 0.494201 |  |  |  |  |
| 0.928027 | 0.494155 |  |  |  |  |
| 0.928257 | 0.493971 |  |  |  |  |
| 0.928487 | 0.494178 |  |  |  |  |
| 0.928694 | 0.494316 |  |  |  |  |
| 0.928924 | 0.49404 |  |  |  |  |
| 0.929177 | 0.494362 |  |  |  |  |
| 0.929384 | 0.494638 |  |  |  |  |
| 0.929614 | 0.49496 |  |  |  |  |
| 0.929844 | 0.494822 |  |  |  |  |
| 0.930051 | 0.494477 |  |  |  |  |
| 0.930281 | 0.494707 |  |  |  |  |
| 0.930511 | 0.49496 |  |  |  |  |
| 0.930764 | 0.495075 |  |  |  |  |
| 0.930971 | 0.494937 |  |  |  |  |
| 0.931201 | 0.49519 |  |  |  |  |
| 0.931431 | 0.495259 |  |  |  |  |
| 0.931638 | 0.49542 |  |  |  |  |
| 0.931868 | 0.495259 |  |  |  |  |
| 0.932098 | 0.495144 |  |  |  |  |
| 0.932328 | 0.495512 |  |  |  |  |
| 0.932558 | 0.495489 |  |  |  |  |
| 0.932788 | 0.495512 |  |  |  |  |
| 0.932995 | 0.495305 |  |  |  |  |
| 0.933225 | 0.495489 |  |  |  |  |
| 0.933455 | 0.495811 |  |  |  |  |
| 0.933685 | 0.495811 |  |  |  |  |
| 0.933915 | 0.495627 |  |  |  |  |
| 0.934145 | 0.495811 |  |  |  |  |
| 0.934352 | 0.496064 |  |  |  |  |
| 0.934582 | 0.495857 |  |  |  |  |
| 0.934812 | 0.496202 |  |  |  |  |
| 0.935019 | 0.495995 |  |  |  |  |
| 0.935272 | 0.496225 |  |  |  |  |
| 0.935502 | 0.496271 |  |  |  |  |
| 0.935732 | 0.496248 |  |  |  |  |
| 0.935939 | 0.496202 |  |  |  |  |
| 0.936169 | 0.496248 |  |  |  |  |
| 0.936399 | 0.496731 |  |  |  |  |
| 0.936606 | 0.496685 |  |  |  |  |
| 0.936859 | 0.496662 |  |  |  |  |
| 0.937089 | 0.496685 |  |  |  |  |
| 0.937296 | 0.496961 |  |  |  |  |
| 0.937526 | 0.497053 |  |  |  |  |
| 0.937756 | 0.49703 |  |  |  |  |
| 0.937963 | 0.4968 |  |  |  |  |
| 0.938193 | 0.497283 |  |  |  |  |
| 0.938446 | 0.497306 |  |  |  |  |
| 0.938653 | 0.497191 |  |  |  |  |
| 0.938883 | 0.497283 |  |  |  |  |
| 0.939113 | 0.497444 |  |  |  |  |
| 0.93932 | 0.497283 |  |  |  |  |
| 0.93955 | 0.497582 |  |  |  |  |
| 0.93978 | 0.497536 |  |  |  |  |
| 0.94001 | 0.497605 |  |  |  |  |
| 0.94024 | 0.497904 |  |  |  |  |
| 0.94047 | 0.497858 |  |  |  |  |
| 0.9407 | 0.497835 |  |  |  |  |
| 0.940907 | 0.498019 |  |  |  |  |
| 0.941137 | 0.498226 |  |  |  |  |
| 0.941367 | 0.498272 |  |  |  |  |
| 0.941597 | 0.498157 |  |  |  |  |
| 0.941827 | 0.498042 |  |  |  |  |
| 0.942057 | 0.498456 |  |  |  |  |
| 0.942264 | 0.498249 |  |  |  |  |
| 0.942494 | 0.498456 |  |  |  |  |
| 0.942724 | 0.498226 |  |  |  |  |
| 0.942954 | 0.498548 |  |  |  |  |
| 0.943184 | 0.498571 |  |  |  |  |
| 0.943414 | 0.498594 |  |  |  |  |
| 0.943621 | 0.498801 |  |  |  |  |
| 0.943851 | 0.498525 |  |  |  |  |
| 0.944081 | 0.499008 |  |  |  |  |
| 0.944311 | 0.498893 |  |  |  |  |
| 0.944541 | 0.498962 |  |  |  |  |
| 0.944771 | 0.499238 |  |  |  |  |
| 0.945001 | 0.499192 |  |  |  |  |
| 0.945208 | 0.499376 |  |  |  |  |
| 0.945438 | 0.499261 |  |  |  |  |
| 0.945645 | 0.499215 |  |  |  |  |
| 0.945875 | 0.499422 |  |  |  |  |
| 0.946105 | 0.49933 |  |  |  |  |
| 0.946358 | 0.499767 |  |  |  |  |
| 0.946565 | 0.499652 |  |  |  |  |
| 0.946795 | 0.499514 |  |  |  |  |
| 0.947025 | 0.499767 |  |  |  |  |
| 0.947232 | 0.499629 |  |  |  |  |
| 0.947462 | 0.499951 |  |  |  |  |
| 0.947692 | 0.499813 |  |  |  |  |
| 0.947922 | 0.500066 |  |  |  |  |
| 0.948152 | 0.500112 |  |  |  |  |
| 0.948382 | 0.499974 |  |  |  |  |
| 0.948589 | 0.50002 |  |  |  |  |
| 0.948819 | 0.50025 |  |  |  |  |
| 0.949049 | 0.500457 |  |  |  |  |
| 0.949279 | 0.500434 |  |  |  |  |
| 0.949509 | 0.500342 |  |  |  |  |
| 0.949739 | 0.500549 |  |  |  |  |
| 0.949946 | 0.50071 |  |  |  |  |
| 0.950176 | 0.500986 |  |  |  |  |
| 0.950406 | 0.500756 |  |  |  |  |
| 0.950636 | 0.500802 |  |  |  |  |
| 0.950866 | 0.501124 |  |  |  |  |
| 0.951096 | 0.500986 |  |  |  |  |
| 0.951326 | 0.500963 |  |  |  |  |
| 0.951533 | 0.501285 |  |  |  |  |
| 0.951763 | 0.50117 |  |  |  |  |
| 0.951993 | 0.501285 |  |  |  |  |
| 0.9522 | 0.501147 |  |  |  |  |
| 0.952453 | 0.501308 |  |  |  |  |
| 0.952683 | 0.501653 |  |  |  |  |
| 0.95289 | 0.501607 |  |  |  |  |
| 0.95312 | 0.501745 |  |  |  |  |
| 0.95335 | 0.501561 |  |  |  |  |
| 0.953557 | 0.501515 |  |  |  |  |
| 0.953787 | 0.50186 |  |  |  |  |
| 0.95404 | 0.501837 |  |  |  |  |
| 0.954247 | 0.501722 |  |  |  |  |
| 0.954477 | 0.501998 |  |  |  |  |
| 0.954707 | 0.502021 |  |  |  |  |
| 0.954937 | 0.502205 |  |  |  |  |
| 0.955144 | 0.502113 |  |  |  |  |
| 0.955374 | 0.501998 |  |  |  |  |
| 0.955627 | 0.502527 |  |  |  |  |
| 0.955834 | 0.502412 |  |  |  |  |
| 0.956064 | 0.502297 |  |  |  |  |
| 0.956294 | 0.502481 |  |  |  |  |
| 0.956501 | 0.502596 |  |  |  |  |
| 0.956731 | 0.502803 |  |  |  |  |
| 0.956961 | 0.502734 |  |  |  |  |
| 0.957191 | 0.502642 |  |  |  |  |
| 0.957421 | 0.502665 |  |  |  |  |
| 0.957651 | 0.502987 |  |  |  |  |
| 0.957858 | 0.502941 |  |  |  |  |
| 0.958088 | 0.502803 |  |  |  |  |
| 0.958318 | 0.502918 |  |  |  |  |
| 0.958548 | 0.503263 |  |  |  |  |
| 0.958778 | 0.503286 |  |  |  |  |
| 0.959008 | 0.502987 |  |  |  |  |
| 0.959215 | 0.503079 |  |  |  |  |
| 0.959445 | 0.503401 |  |  |  |  |
| 0.959675 | 0.503493 |  |  |  |  |
| 0.959905 | 0.503654 |  |  |  |  |
| 0.960135 | 0.503516 |  |  |  |  |
| 0.960365 | 0.503608 |  |  |  |  |
| 0.960595 | 0.503631 |  |  |  |  |
| 0.960802 | 0.503539 |  |  |  |  |
| 0.961032 | 0.503723 |  |  |  |  |
| 0.961262 | 0.503608 |  |  |  |  |
| 0.961469 | 0.503838 |  |  |  |  |
| 0.961722 | 0.503907 |  |  |  |  |
| 0.961952 | 0.503976 |  |  |  |  |
| 0.962159 | 0.503953 |  |  |  |  |
| 0.962389 | 0.504206 |  |  |  |  |
| 0.962619 | 0.504068 |  |  |  |  |
| 0.962849 | 0.504045 |  |  |  |  |
| 0.963056 | 0.503861 |  |  |  |  |
| 0.963309 | 0.504459 |  |  |  |  |
| 0.963516 | 0.504574 |  |  |  |  |
| 0.963746 | 0.504344 |  |  |  |  |
| 0.963976 | 0.504436 |  |  |  |  |
| 0.964183 | 0.504689 |  |  |  |  |
| 0.964413 | 0.504666 |  |  |  |  |
| 0.964643 | 0.504896 |  |  |  |  |
| 0.964873 | 0.504666 |  |  |  |  |
| 0.965103 | 0.504758 |  |  |  |  |
| 0.965333 | 0.505126 |  |  |  |  |
| 0.965563 | 0.505034 |  |  |  |  |
| 0.96577 | 0.505034 |  |  |  |  |
| 0.966 | 0.505011 |  |  |  |  |
| 0.96623 | 0.505632 |  |  |  |  |
| 0.96646 | 0.505287 |  |  |  |  |
| 0.96669 | 0.505287 |  |  |  |  |
| 0.96692 | 0.505011 |  |  |  |  |
| 0.967127 | 0.505517 |  |  |  |  |
| 0.967357 | 0.505425 |  |  |  |  |
| 0.967587 | 0.505448 |  |  |  |  |
| 0.967817 | 0.505356 |  |  |  |  |
| 0.968047 | 0.505701 |  |  |  |  |
| 0.968277 | 0.505816 |  |  |  |  |
| 0.968507 | 0.506069 |  |  |  |  |
| 0.968714 | 0.505885 |  |  |  |  |
| 0.968944 | 0.506 |  |  |  |  |
| 0.969174 | 0.50623 |  |  |  |  |
| 0.969381 | 0.506092 |  |  |  |  |
| 0.969634 | 0.506253 |  |  |  |  |
| 0.969841 | 0.506299 |  |  |  |  |
| 0.970071 | 0.506598 |  |  |  |  |
| 0.970301 | 0.506414 |  |  |  |  |
| 0.970531 | 0.506529 |  |  |  |  |
| 0.970738 | 0.506506 |  |  |  |  |
| 0.970968 | 0.506529 |  |  |  |  |
| 0.971221 | 0.506713 |  |  |  |  |
| 0.971428 | 0.506943 |  |  |  |  |
| 0.971658 | 0.506782 |  |  |  |  |
| 0.971888 | 0.506759 |  |  |  |  |
| 0.972095 | 0.50715 |  |  |  |  |
| 0.972325 | 0.507081 |  |  |  |  |
| 0.972555 | 0.506989 |  |  |  |  |
| 0.972785 | 0.507012 |  |  |  |  |
| 0.973015 | 0.507196 |  |  |  |  |
| 0.973245 | 0.507081 |  |  |  |  |
| 0.973475 | 0.507104 |  |  |  |  |
| 0.973682 | 0.507058 |  |  |  |  |
| 0.973912 | 0.507357 |  |  |  |  |
| 0.974142 | 0.507449 |  |  |  |  |
| 0.974372 | 0.50761 |  |  |  |  |
| 0.974602 | 0.507564 |  |  |  |  |
| 0.974832 | 0.507633 |  |  |  |  |
| 0.975039 | 0.507633 |  |  |  |  |
| 0.975269 | 0.507817 |  |  |  |  |
| 0.975499 | 0.507656 |  |  |  |  |
| 0.975729 | 0.507909 |  |  |  |  |
| 0.975959 | 0.508001 |  |  |  |  |
| 0.976189 | 0.507886 |  |  |  |  |
| 0.976396 | 0.507978 |  |  |  |  |
| 0.976626 | 0.508162 |  |  |  |  |
| 0.976856 | 0.508093 |  |  |  |  |
| 0.977063 | 0.508116 |  |  |  |  |
| 0.977316 | 0.508185 |  |  |  |  |
| 0.977546 | 0.508231 |  |  |  |  |
| 0.977753 | 0.508369 |  |  |  |  |
| 0.977983 | 0.508576 |  |  |  |  |
| 0.978213 | 0.508852 |  |  |  |  |
| 0.97842 | 0.508346 |  |  |  |  |
| 0.97865 | 0.508461 |  |  |  |  |
| 0.978903 | 0.508691 |  |  |  |  |
| 0.979133 | 0.508714 |  |  |  |  |
| 0.97934 | 0.508806 |  |  |  |  |
| 0.97957 | 0.508714 |  |  |  |  |
| 0.9798 | 0.50899 |  |  |  |  |
| 0.980007 | 0.508967 |  |  |  |  |
| 0.980237 | 0.509082 |  |  |  |  |
| 0.98049 | 0.509174 |  |  |  |  |
| 0.980697 | 0.509335 |  |  |  |  |
| 0.980927 | 0.509358 |  |  |  |  |
| 0.981157 | 0.509335 |  |  |  |  |
| 0.981364 | 0.509243 |  |  |  |  |
| 0.981594 | 0.509496 |  |  |  |  |
| 0.981824 | 0.509519 |  |  |  |  |
| 0.982077 | 0.509496 |  |  |  |  |
| 0.982284 | 0.509588 |  |  |  |  |
| 0.982514 | 0.509795 |  |  |  |  |
| 0.982721 | 0.510002 |  |  |  |  |
| 0.982951 | 0.509634 |  |  |  |  |
| 0.983181 | 0.509841 |  |  |  |  |
| 0.983411 | 0.509565 |  |  |  |  |
| 0.983641 | 0.510094 |  |  |  |  |
| 0.983871 | 0.510163 |  |  |  |  |
| 0.984101 | 0.510094 |  |  |  |  |
| 0.984308 | 0.510232 |  |  |  |  |
| 0.984538 | 0.510278 |  |  |  |  |
| 0.984768 | 0.510393 |  |  |  |  |
| 0.984998 | 0.510301 |  |  |  |  |
| 0.985228 | 0.510278 |  |  |  |  |
| 0.985458 | 0.510393 |  |  |  |  |
| 0.985665 | 0.51083 |  |  |  |  |
| 0.985895 | 0.5106 |  |  |  |  |
| 0.986125 | 0.510761 |  |  |  |  |
| 0.986332 | 0.510623 |  |  |  |  |
| 0.986585 | 0.510991 |  |  |  |  |
| 0.986815 | 0.510738 |  |  |  |  |
| 0.987045 | 0.510968 |  |  |  |  |
| 0.987252 | 0.510945 |  |  |  |  |
| 0.987482 | 0.510968 |  |  |  |  |
| 0.987689 | 0.51129 |  |  |  |  |
| 0.987919 | 0.511037 |  |  |  |  |
| 0.988149 | 0.511382 |  |  |  |  |
| 0.988379 | 0.511244 |  |  |  |  |
| 0.988609 | 0.51152 |  |  |  |  |
| 0.988839 | 0.511658 |  |  |  |  |
| 0.989069 | 0.511382 |  |  |  |  |
| 0.989276 | 0.511635 |  |  |  |  |
| 0.989506 | 0.511497 |  |  |  |  |
| 0.989736 | 0.511865 |  |  |  |  |
| 0.989966 | 0.511566 |  |  |  |  |
| 0.990196 | 0.511658 |  |  |  |  |
| 0.990426 | 0.511865 |  |  |  |  |
| 0.990633 | 0.511911 |  |  |  |  |
| 0.990863 | 0.511934 |  |  |  |  |
| 0.991093 | 0.511911 |  |  |  |  |
| 0.991323 | 0.512095 |  |  |  |  |
| 0.991553 | 0.512164 |  |  |  |  |
| 0.991783 | 0.511957 |  |  |  |  |
| 0.99199 | 0.511888 |  |  |  |  |
| 0.99222 | 0.512187 |  |  |  |  |
| 0.99245 | 0.512647 |  |  |  |  |
| 0.99268 | 0.512141 |  |  |  |  |
| 0.99291 | 0.512302 |  |  |  |  |
| 0.99314 | 0.512348 |  |  |  |  |
| 0.99337 | 0.512785 |  |  |  |  |
| 0.993577 | 0.512578 |  |  |  |  |
| 0.993807 | 0.512509 |  |  |  |  |
| 0.994014 | 0.512693 |  |  |  |  |
| 0.994244 | 0.513176 |  |  |  |  |
| 0.994497 | 0.5129 |  |  |  |  |
| 0.994727 | 0.512946 |  |  |  |  |
| 0.994934 | 0.512992 |  |  |  |  |
| 0.995164 | 0.513015 |  |  |  |  |
| 0.995394 | 0.512992 |  |  |  |  |
| 0.995601 | 0.513337 |  |  |  |  |
| 0.995831 | 0.513268 |  |  |  |  |
| 0.996084 | 0.513383 |  |  |  |  |
| 0.996291 | 0.513429 |  |  |  |  |
| 0.996521 | 0.513705 |  |  |  |  |
| 0.996751 | 0.513498 |  |  |  |  |
| 0.996958 | 0.513429 |  |  |  |  |
| 0.997188 | 0.514004 |  |  |  |  |
| 0.997418 | 0.513728 |  |  |  |  |
| 0.997671 | 0.513705 |  |  |  |  |
| 0.997878 | 0.513613 |  |  |  |  |
| 0.998108 | 0.51382 |  |  |  |  |
| 0.998338 | 0.513866 |  |  |  |  |
| 0.998545 | 0.513912 |  |  |  |  |
| 0.998775 | 0.514004 |  |  |  |  |
| 0.999005 | 0.514441 |  |  |  |  |
| 0.999235 | 0.514395 |  |  |  |  |
| 0.999465 | 0.514349 |  |  |  |  |
| 0.999695 | 0.514234 |  |  |  |  |
| 0.999902 | 0.514441 |  |  |  |  |
| 1.000132 | 0.514717 |  |  |  |  |
| 1.000362 | 0.514418 |  |  |  |  |
| 1.000592 | 0.514395 |  |  |  |  |
| 1.000822 | 0.514395 |  |  |  |  |
| 1.001052 | 0.514763 |  |  |  |  |
| 1.001259 | 0.514694 |  |  |  |  |
| 1.001489 | 0.514671 |  |  |  |  |
| 1.001719 | 0.514579 |  |  |  |  |
| 1.001926 | 0.515039 |  |  |  |  |
| 1.002179 | 0.514947 |  |  |  |  |
| 1.002409 | 0.514878 |  |  |  |  |
| 1.002616 | 0.514947 |  |  |  |  |
| 1.002846 | 0.515361 |  |  |  |  |
| 1.003076 | 0.515131 |  |  |  |  |
| 1.003306 | 0.515292 |  |  |  |  |
| 1.003513 | 0.5152 |  |  |  |  |
| 1.003766 | 0.515062 |  |  |  |  |
| 1.003996 | 0.515338 |  |  |  |  |
| 1.004203 | 0.515545 |  |  |  |  |
| 1.004433 | 0.51566 |  |  |  |  |
| 1.004663 | 0.515453 |  |  |  |  |
| 1.00487 | 0.515683 |  |  |  |  |
| 1.0051 | 0.515752 |  |  |  |  |
| 1.005353 | 0.515706 |  |  |  |  |
| 1.00556 | 0.515775 |  |  |  |  |
| 1.00579 | 0.515982 |  |  |  |  |
| 1.00602 | 0.516051 |  |  |  |  |
| 1.006227 | 0.516051 |  |  |  |  |
| 1.006457 | 0.516005 |  |  |  |  |
| 1.006687 | 0.516051 |  |  |  |  |
| 1.006917 | 0.51635 |  |  |  |  |
| 1.007147 | 0.516373 |  |  |  |  |
| 1.007377 | 0.516051 |  |  |  |  |
| 1.007584 | 0.51612 |  |  |  |  |
| 1.007814 | 0.516511 |  |  |  |  |
| 1.008044 | 0.516603 |  |  |  |  |
| 1.008274 | 0.516672 |  |  |  |  |
| 1.008504 | 0.516603 |  |  |  |  |
| 1.008734 | 0.516764 |  |  |  |  |
| 1.008964 | 0.516925 |  |  |  |  |
| 1.009171 | 0.516994 |  |  |  |  |
| 1.009401 | 0.516787 |  |  |  |  |
| 1.009631 | 0.516787 |  |  |  |  |
| 1.009861 | 0.517201 |  |  |  |  |
| 1.010091 | 0.516879 |  |  |  |  |
| 1.010321 | 0.516948 |  |  |  |  |
| 1.010528 | 0.516971 |  |  |  |  |
| 1.010758 | 0.517362 |  |  |  |  |
| 1.010988 | 0.517408 |  |  |  |  |
| 1.011218 | 0.517316 |  |  |  |  |
| 1.011425 | 0.517178 |  |  |  |  |
| 1.011678 | 0.517615 |  |  |  |  |
| 1.011885 | 0.517477 |  |  |  |  |
| 1.012115 | 0.517569 |  |  |  |  |
| 1.012345 | 0.517546 |  |  |  |  |
| 1.012575 | 0.517569 |  |  |  |  |
| 1.012782 | 0.51773 |  |  |  |  |
| 1.013012 | 0.517776 |  |  |  |  |
| 1.013242 | 0.517891 |  |  |  |  |
| 1.013472 | 0.517822 |  |  |  |  |
| 1.013702 | 0.518213 |  |  |  |  |
| 1.013932 | 0.517891 |  |  |  |  |
| 1.014139 | 0.517822 |  |  |  |  |
| 1.014369 | 0.518167 |  |  |  |  |
| 1.014599 | 0.518075 |  |  |  |  |
| 1.014829 | 0.518328 |  |  |  |  |
| 1.015059 | 0.518466 |  |  |  |  |
| 1.015289 | 0.518305 |  |  |  |  |
| 1.015496 | 0.518397 |  |  |  |  |
| 1.015726 | 0.518512 |  |  |  |  |
| 1.015956 | 0.518581 |  |  |  |  |
| 1.016186 | 0.518489 |  |  |  |  |
| 1.016416 | 0.518581 |  |  |  |  |
| 1.016646 | 0.518857 |  |  |  |  |
| 1.016876 | 0.518903 |  |  |  |  |
| 1.017083 | 0.518742 |  |  |  |  |
| 1.017313 | 0.51888 |  |  |  |  |
| 1.017543 | 0.519041 |  |  |  |  |
| 1.017773 | 0.519156 |  |  |  |  |
| 1.018003 | 0.518949 |  |  |  |  |
| 1.01821 | 0.519133 |  |  |  |  |
| 1.01844 | 0.519455 |  |  |  |  |
| 1.01867 | 0.519156 |  |  |  |  |
| 1.0189 | 0.519248 |  |  |  |  |
| 1.019107 | 0.51911 |  |  |  |  |
| 1.01936 | 0.519363 |  |  |  |  |
| 1.01959 | 0.519409 |  |  |  |  |
| 1.019797 | 0.519363 |  |  |  |  |
| 1.020027 | 0.519248 |  |  |  |  |
| 1.020257 | 0.519708 |  |  |  |  |
| 1.020464 | 0.5198 |  |  |  |  |
| 1.020694 | 0.519708 |  |  |  |  |
| 1.020947 | 0.519524 |  |  |  |  |
| 1.021154 | 0.5198 |  |  |  |  |
| 1.021384 | 0.519984 |  |  |  |  |
| 1.021614 | 0.520214 |  |  |  |  |
| 1.021844 | 0.519984 |  |  |  |  |
| 1.022051 | 0.520283 |  |  |  |  |
| 1.022281 | 0.520122 |  |  |  |  |
| 1.022534 | 0.520329 |  |  |  |  |
| 1.022741 | 0.520467 |  |  |  |  |
| 1.022971 | 0.520398 |  |  |  |  |
| 1.023178 | 0.52026 |  |  |  |  |
| 1.023408 | 0.520444 |  |  |  |  |
| 1.023638 | 0.520559 |  |  |  |  |
| 1.023868 | 0.520421 |  |  |  |  |
| 1.024098 | 0.520628 |  |  |  |  |
| 1.024328 | 0.520628 |  |  |  |  |
| 1.024558 | 0.520674 |  |  |  |  |
| 1.024765 | 0.520398 |  |  |  |  |
| 1.024995 | 0.520444 |  |  |  |  |
| 1.025225 | 0.520651 |  |  |  |  |
| 1.025455 | 0.521226 |  |  |  |  |
| 1.025685 | 0.520927 |  |  |  |  |
| 1.025915 | 0.521042 |  |  |  |  |
| 1.026122 | 0.521088 |  |  |  |  |
| 1.026352 | 0.521318 |  |  |  |  |
| 1.026582 | 0.521249 |  |  |  |  |
| 1.026812 | 0.52118 |  |  |  |  |
| 1.027042 | 0.521295 |  |  |  |  |
| 1.027272 | 0.521548 |  |  |  |  |
| 1.027502 | 0.521502 |  |  |  |  |
| 1.027709 | 0.521387 |  |  |  |  |
| 1.027939 | 0.521479 |  |  |  |  |
| 1.028169 | 0.521824 |  |  |  |  |
| 1.028376 | 0.521732 |  |  |  |  |
| 1.028629 | 0.521709 |  |  |  |  |
| 1.028859 | 0.521732 |  |  |  |  |
| 1.029066 | 0.521709 |  |  |  |  |
| 1.029296 | 0.521709 |  |  |  |  |
| 1.029526 | 0.521847 |  |  |  |  |
| 1.029733 | 0.521824 |  |  |  |  |
| 1.029963 | 0.522238 |  |  |  |  |
| 1.030193 | 0.522376 |  |  |  |  |
| 1.030423 | 0.521985 |  |  |  |  |
| 1.030653 | 0.522169 |  |  |  |  |
| 1.030883 | 0.522215 |  |  |  |  |
| 1.03109 | 0.522123 |  |  |  |  |
| 1.03132 | 0.522514 |  |  |  |  |
| 1.03155 | 0.522307 |  |  |  |  |
| 1.03178 | 0.522422 |  |  |  |  |
| 1.03201 | 0.522721 |  |  |  |  |
| 1.03224 | 0.52256 |  |  |  |  |
| 1.03247 | 0.522675 |  |  |  |  |
| 1.032677 | 0.522606 |  |  |  |  |
| 1.032907 | 0.52279 |  |  |  |  |
| 1.033137 | 0.522905 |  |  |  |  |
| 1.033367 | 0.522882 |  |  |  |  |
| 1.033597 | 0.522813 |  |  |  |  |
| 1.033827 | 0.522905 |  |  |  |  |
| 1.034034 | 0.522882 |  |  |  |  |
| 1.034264 | 0.522974 |  |  |  |  |
| 1.034494 | 0.523066 |  |  |  |  |
| 1.034701 | 0.522859 |  |  |  |  |
| 1.034954 | 0.523572 |  |  |  |  |
| 1.035184 | 0.523204 |  |  |  |  |
| 1.035391 | 0.523181 |  |  |  |  |
| 1.035621 | 0.523204 |  |  |  |  |
| 1.035851 | 0.523595 |  |  |  |  |
| 1.036058 | 0.523641 |  |  |  |  |
| 1.036288 | 0.523549 |  |  |  |  |
| 1.036541 | 0.523503 |  |  |  |  |
| 1.036748 | 0.523917 |  |  |  |  |
| 1.036978 | 0.524078 |  |  |  |  |
| 1.037208 | 0.523756 |  |  |  |  |
| 1.037438 | 0.523802 |  |  |  |  |
| 1.037645 | 0.524055 |  |  |  |  |
| 1.037875 | 0.524078 |  |  |  |  |
| 1.038128 | 0.524193 |  |  |  |  |
| 1.038335 | 0.523825 |  |  |  |  |
| 1.038565 | 0.523963 |  |  |  |  |
| 1.038795 | 0.524262 |  |  |  |  |
| 1.039002 | 0.523986 |  |  |  |  |
| 1.039232 | 0.524216 |  |  |  |  |
| 1.039462 | 0.524331 |  |  |  |  |
| 1.039692 | 0.524446 |  |  |  |  |
| 1.039922 | 0.524446 |  |  |  |  |
| 1.040152 | 0.524883 |  |  |  |  |
| 1.040359 | 0.524469 |  |  |  |  |
| 1.040589 | 0.524354 |  |  |  |  |
| 1.040819 | 0.524722 |  |  |  |  |
| 1.041049 | 0.524814 |  |  |  |  |
| 1.041279 | 0.524699 |  |  |  |  |
| 1.041509 | 0.524791 |  |  |  |  |
| 1.041739 | 0.525044 |  |  |  |  |
| 1.041946 | 0.524883 |  |  |  |  |
| 1.042176 | 0.524837 |  |  |  |  |
| 1.042383 | 0.524883 |  |  |  |  |
| 1.042636 | 0.525182 |  |  |  |  |
| 1.042866 | 0.525297 |  |  |  |  |
| 1.043096 | 0.525297 |  |  |  |  |
| 1.043303 | 0.525297 |  |  |  |  |
| 1.043533 | 0.525274 |  |  |  |  |
| 1.043763 | 0.52555 |  |  |  |  |
| 1.04397 | 0.525412 |  |  |  |  |
| 1.044223 | 0.525389 |  |  |  |  |
| 1.044453 | 0.525504 |  |  |  |  |
| 1.04466 | 0.525711 |  |  |  |  |
| 1.04489 | 0.525734 |  |  |  |  |
| 1.04512 | 0.52555 |  |  |  |  |
| 1.045327 | 0.525504 |  |  |  |  |
| 1.045557 | 0.526102 |  |  |  |  |
| 1.04581 | 0.525941 |  |  |  |  |
| 1.04604 | 0.525941 |  |  |  |  |
| 1.046247 | 0.525803 |  |  |  |  |
| 1.046477 | 0.52601 |  |  |  |  |
| 1.046707 | 0.526125 |  |  |  |  |
| 1.046914 | 0.526171 |  |  |  |  |
| 1.047144 | 0.525964 |  |  |  |  |
| 1.047397 | 0.526079 |  |  |  |  |
| 1.047604 | 0.526516 |  |  |  |  |
| 1.047834 | 0.526263 |  |  |  |  |
| 1.048064 | 0.526332 |  |  |  |  |
| 1.048271 | 0.526217 |  |  |  |  |
| 1.048501 | 0.5267 |  |  |  |  |
| 1.048731 | 0.526539 |  |  |  |  |
| 1.048961 | 0.526746 |  |  |  |  |
| 1.049191 | 0.526493 |  |  |  |  |
| 1.049421 | 0.526608 |  |  |  |  |
| 1.049628 | 0.526976 |  |  |  |  |
| 1.049858 | 0.526677 |  |  |  |  |
| 1.050088 | 0.52693 |  |  |  |  |
| 1.050318 | 0.527137 |  |  |  |  |
| 1.050548 | 0.527045 |  |  |  |  |
| 1.050778 | 0.526976 |  |  |  |  |
| 1.050985 | 0.526884 |  |  |  |  |
| 1.051215 | 0.527114 |  |  |  |  |
| 1.051445 | 0.527413 |  |  |  |  |
| 1.051675 | 0.527413 |  |  |  |  |
| 1.051905 | 0.527137 |  |  |  |  |
| 1.052135 | 0.527275 |  |  |  |  |
| 1.052365 | 0.527436 |  |  |  |  |
| 1.052572 | 0.527597 |  |  |  |  |
| 1.052802 | 0.527321 |  |  |  |  |
| 1.053032 | 0.52739 |  |  |  |  |
| 1.053239 | 0.527689 |  |  |  |  |
| 1.053469 | 0.527735 |  |  |  |  |
| 1.053722 | 0.527758 |  |  |  |  |
| 1.053929 | 0.527597 |  |  |  |  |
| 1.054159 | 0.527781 |  |  |  |  |
| 1.054389 | 0.527942 |  |  |  |  |
| 1.054596 | 0.52785 |  |  |  |  |
| 1.054826 | 0.527988 |  |  |  |  |
| 1.055056 | 0.528011 |  |  |  |  |
| 1.055286 | 0.528149 |  |  |  |  |
| 1.055516 | 0.528264 |  |  |  |  |
| 1.055746 | 0.52808 |  |  |  |  |
| 1.055953 | 0.528172 |  |  |  |  |
| 1.056183 | 0.528379 |  |  |  |  |
| 1.056413 | 0.528218 |  |  |  |  |
| 1.056643 | 0.528264 |  |  |  |  |
| 1.056873 | 0.528333 |  |  |  |  |
| 1.057103 | 0.528425 |  |  |  |  |
| 1.057333 | 0.528632 |  |  |  |  |
| 1.05754 | 0.528425 |  |  |  |  |
| 1.05777 | 0.528218 |  |  |  |  |
| 1.058 | 0.528609 |  |  |  |  |
| 1.05823 | 0.52877 |  |  |  |  |
| 1.05846 | 0.528701 |  |  |  |  |
| 1.05869 | 0.528632 |  |  |  |  |
| 1.058897 | 0.528954 |  |  |  |  |
| 1.059127 | 0.528885 |  |  |  |  |
| 1.059357 | 0.528885 |  |  |  |  |
| 1.059587 | 0.529 |  |  |  |  |
| 1.059817 | 0.529046 |  |  |  |  |
| 1.060047 | 0.529368 |  |  |  |  |
| 1.060254 | 0.529276 |  |  |  |  |
| 1.060484 | 0.529138 |  |  |  |  |
| 1.060714 | 0.529345 |  |  |  |  |
| 1.060944 | 0.529414 |  |  |  |  |
| 1.061151 | 0.529598 |  |  |  |  |
| 1.061404 | 0.529483 |  |  |  |  |
| 1.061634 | 0.52946 |  |  |  |  |
| 1.061841 | 0.52946 |  |  |  |  |
| 1.062071 | 0.529805 |  |  |  |  |
| 1.062301 | 0.529736 |  |  |  |  |
| 1.062508 | 0.529736 |  |  |  |  |
| 1.062738 | 0.529644 |  |  |  |  |
| 1.062991 | 0.530104 |  |  |  |  |
| 1.063198 | 0.530196 |  |  |  |  |
| 1.063428 | 0.52992 |  |  |  |  |
| 1.063658 | 0.530173 |  |  |  |  |
| 1.063865 | 0.530058 |  |  |  |  |
| 1.064095 | 0.530035 |  |  |  |  |
| 1.064325 | 0.530242 |  |  |  |  |
| 1.064555 | 0.530334 |  |  |  |  |
| 1.064785 | 0.530334 |  |  |  |  |
| 1.065015 | 0.530449 |  |  |  |  |
| 1.065245 | 0.530541 |  |  |  |  |
| 1.065452 | 0.53038 |  |  |  |  |
| 1.065682 | 0.530633 |  |  |  |  |
| 1.065912 | 0.53061 |  |  |  |  |
| 1.066142 | 0.530863 |  |  |  |  |
| 1.066372 | 0.530725 |  |  |  |  |
| 1.066579 | 0.530932 |  |  |  |  |
| 1.066809 | 0.530886 |  |  |  |  |
| 1.067039 | 0.531024 |  |  |  |  |
| 1.067269 | 0.530817 |  |  |  |  |
| 1.067499 | 0.530863 |  |  |  |  |
| 1.067729 | 0.530978 |  |  |  |  |
| 1.067959 | 0.531277 |  |  |  |  |
| 1.068166 | 0.531254 |  |  |  |  |
| 1.068396 | 0.531139 |  |  |  |  |
| 1.068626 | 0.531139 |  |  |  |  |
| 1.068833 | 0.531323 |  |  |  |  |
| 1.069086 | 0.531484 |  |  |  |  |
| 1.069316 | 0.531599 |  |  |  |  |
| 1.069523 | 0.531415 |  |  |  |  |
| 1.069753 | 0.53199 |  |  |  |  |
| 1.069983 | 0.531691 |  |  |  |  |
| 1.070213 | 0.53153 |  |  |  |  |
| 1.07042 | 0.531783 |  |  |  |  |
| 1.070673 | 0.531691 |  |  |  |  |
| 1.070903 | 0.531714 |  |  |  |  |
| 1.07111 | 0.532105 |  |  |  |  |
| 1.07134 | 0.531852 |  |  |  |  |
| 1.07157 | 0.53199 |  |  |  |  |
| 1.071777 | 0.532289 |  |  |  |  |
| 1.072007 | 0.532082 |  |  |  |  |
| 1.072237 | 0.532289 |  |  |  |  |
| 1.072467 | 0.532266 |  |  |  |  |
| 1.072697 | 0.532588 |  |  |  |  |
| 1.072927 | 0.532174 |  |  |  |  |
| 1.073134 | 0.53222 |  |  |  |  |
| 1.073364 | 0.532381 |  |  |  |  |
| 1.073594 | 0.532657 |  |  |  |  |
| 1.073824 | 0.532772 |  |  |  |  |
| 1.074054 | 0.532956 |  |  |  |  |
| 1.074284 | 0.532611 |  |  |  |  |
| 1.074491 | 0.532703 |  |  |  |  |
| 1.074721 | 0.532703 |  |  |  |  |
| 1.074951 | 0.532726 |  |  |  |  |
| 1.075181 | 0.532542 |  |  |  |  |
| 1.075411 | 0.532657 |  |  |  |  |
| 1.075641 | 0.532979 |  |  |  |  |
| 1.075871 | 0.533094 |  |  |  |  |
| 1.076078 | 0.533002 |  |  |  |  |
| 1.076308 | 0.532933 |  |  |  |  |
| 1.076538 | 0.533462 |  |  |  |  |
| 1.076768 | 0.533163 |  |  |  |  |
| 1.076998 | 0.533071 |  |  |  |  |
| 1.077228 | 0.533232 |  |  |  |  |
| 1.077435 | 0.533301 |  |  |  |  |
| 1.077665 | 0.533462 |  |  |  |  |
| 1.077895 | 0.533324 |  |  |  |  |
| 1.078102 | 0.533623 |  |  |  |  |
| 1.078332 | 0.533669 |  |  |  |  |
| 1.078585 | 0.533899 |  |  |  |  |
| 1.078792 | 0.533692 |  |  |  |  |
| 1.079022 | 0.533784 |  |  |  |  |
| 1.079252 | 0.533715 |  |  |  |  |
| 1.079459 | 0.533807 |  |  |  |  |
| 1.079689 | 0.534014 |  |  |  |  |
| 1.079919 | 0.533922 |  |  |  |  |
| 1.080149 | 0.533692 |  |  |  |  |
| 1.080379 | 0.534037 |  |  |  |  |
| 1.080609 | 0.534198 |  |  |  |  |
| 1.080839 | 0.534198 |  |  |  |  |
| 1.081046 | 0.534129 |  |  |  |  |
| 1.081276 | 0.534359 |  |  |  |  |
| 1.081506 | 0.534681 |  |  |  |  |
| 1.081736 | 0.534773 |  |  |  |  |
| 1.081966 | 0.534221 |  |  |  |  |
| 1.082196 | 0.534566 |  |  |  |  |
| 1.082403 | 0.534796 |  |  |  |  |
| 1.082633 | 0.53475 |  |  |  |  |
| 1.082863 | 0.534474 |  |  |  |  |
| 1.083093 | 0.534635 |  |  |  |  |
| 1.083323 | 0.534842 |  |  |  |  |
| 1.083553 | 0.534865 |  |  |  |  |
| 1.083783 | 0.534773 |  |  |  |  |
| 1.08399 | 0.534957 |  |  |  |  |
| 1.08422 | 0.535187 |  |  |  |  |
| 1.084427 | 0.535302 |  |  |  |  |
| 1.08468 | 0.535187 |  |  |  |  |
| 1.08491 | 0.535072 |  |  |  |  |
| 1.085117 | 0.535164 |  |  |  |  |
| 1.085347 | 0.535463 |  |  |  |  |
| 1.085577 | 0.535348 |  |  |  |  |
| 1.085807 | 0.535486 |  |  |  |  |
| 1.086014 | 0.535417 |  |  |  |  |
| 1.086267 | 0.535739 |  |  |  |  |
| 1.086497 | 0.535555 |  |  |  |  |
| 1.086704 | 0.535601 |  |  |  |  |
| 1.086934 | 0.535601 |  |  |  |  |
| 1.087164 | 0.535831 |  |  |  |  |
| 1.087371 | 0.535785 |  |  |  |  |
| 1.087601 | 0.535923 |  |  |  |  |
| 1.087854 | 0.535854 |  |  |  |  |
| 1.088061 | 0.536061 |  |  |  |  |
| 1.088291 | 0.536107 |  |  |  |  |
| 1.088521 | 0.536222 |  |  |  |  |
| 1.088728 | 0.535992 |  |  |  |  |
| 1.088958 | 0.536015 |  |  |  |  |
| 1.089188 | 0.53636 |  |  |  |  |
| 1.089441 | 0.536222 |  |  |  |  |
| 1.089648 | 0.536176 |  |  |  |  |
| 1.089878 | 0.536613 |  |  |  |  |
| 1.090108 | 0.53659 |  |  |  |  |
| 1.090315 | 0.536521 |  |  |  |  |
| 1.090545 | 0.53659 |  |  |  |  |
| 1.090775 | 0.536521 |  |  |  |  |
| 1.091005 | 0.536912 |  |  |  |  |
| 1.091235 | 0.537096 |  |  |  |  |
| 1.091465 | 0.536751 |  |  |  |  |
| 1.091672 | 0.536475 |  |  |  |  |
| 1.091902 | 0.536774 |  |  |  |  |
| 1.092132 | 0.537211 |  |  |  |  |
| 1.092362 | 0.536981 |  |  |  |  |
| 1.092592 | 0.536705 |  |  |  |  |
| 1.092822 | 0.537004 |  |  |  |  |
| 1.093029 | 0.537142 |  |  |  |  |
| 1.093259 | 0.537004 |  |  |  |  |
| 1.093489 | 0.537165 |  |  |  |  |
| 1.093696 | 0.53705 |  |  |  |  |
| 1.093949 | 0.537234 |  |  |  |  |
| 1.094179 | 0.537326 |  |  |  |  |
| 1.094409 | 0.537441 |  |  |  |  |
| 1.094616 | 0.537188 |  |  |  |  |
| 1.094846 | 0.537579 |  |  |  |  |
| 1.095076 | 0.537533 |  |  |  |  |
| 1.095283 | 0.537602 |  |  |  |  |
| 1.095513 | 0.537556 |  |  |  |  |
| 1.095766 | 0.537717 |  |  |  |  |
| 1.095973 | 0.537602 |  |  |  |  |
| 1.096203 | 0.537832 |  |  |  |  |
| 1.096433 | 0.537924 |  |  |  |  |
| 1.09664 | 0.537924 |  |  |  |  |
| 1.09687 | 0.538131 |  |  |  |  |
| 1.0971 | 0.537924 |  |  |  |  |
| 1.09733 | 0.538016 |  |  |  |  |
| 1.09756 | 0.537924 |  |  |  |  |
| 1.09779 | 0.538292 |  |  |  |  |
| 1.097997 | 0.538407 |  |  |  |  |
| 1.098227 | 0.538292 |  |  |  |  |
| 1.098457 | 0.538476 |  |  |  |  |
| 1.098687 | 0.538177 |  |  |  |  |
| 1.098917 | 0.538614 |  |  |  |  |
| 1.099147 | 0.538499 |  |  |  |  |
| 1.099377 | 0.538499 |  |  |  |  |
| 1.099607 | 0.538752 |  |  |  |  |
| 1.099814 | 0.539005 |  |  |  |  |
| 1.100044 | 0.538683 |  |  |  |  |
| 1.100274 | 0.538568 |  |  |  |  |
| 1.100504 | 0.538775 |  |  |  |  |
| 1.100734 | 0.539074 |  |  |  |  |
| 1.100964 | 0.539235 |  |  |  |  |
| 1.101171 | 0.539028 |  |  |  |  |
| 1.101401 | 0.539005 |  |  |  |  |
| 1.101631 | 0.539396 |  |  |  |  |
| 1.101861 | 0.539166 |  |  |  |  |
| 1.102091 | 0.539212 |  |  |  |  |
| 1.102321 | 0.539281 |  |  |  |  |
| 1.102528 | 0.539258 |  |  |  |  |
| 1.102758 | 0.539626 |  |  |  |  |
| 1.102988 | 0.539442 |  |  |  |  |
| 1.103218 | 0.53935 |  |  |  |  |
| 1.103448 | 0.539603 |  |  |  |  |
| 1.103678 | 0.539626 |  |  |  |  |
| 1.103908 | 0.539672 |  |  |  |  |
| 1.104115 | 0.53958 |  |  |  |  |
| 1.104345 | 0.539695 |  |  |  |  |
| 1.104575 | 0.539925 |  |  |  |  |
| 1.104782 | 0.539787 |  |  |  |  |
| 1.105035 | 0.53981 |  |  |  |  |
| 1.105265 | 0.539994 |  |  |  |  |
| 1.105472 | 0.540063 |  |  |  |  |
| 1.105702 | 0.539994 |  |  |  |  |
| 1.105932 | 0.540132 |  |  |  |  |
| 1.106139 | 0.539948 |  |  |  |  |
| 1.106369 | 0.540155 |  |  |  |  |
| 1.106622 | 0.540224 |  |  |  |  |
| 1.106852 | 0.540201 |  |  |  |  |
| 1.107059 | 0.540316 |  |  |  |  |
| 1.107289 | 0.540247 |  |  |  |  |
| 1.107519 | 0.540339 |  |  |  |  |
| 1.107726 | 0.540615 |  |  |  |  |
| 1.107956 | 0.540546 |  |  |  |  |
| 1.108186 | 0.540569 |  |  |  |  |
| 1.108416 | 0.540776 |  |  |  |  |
| 1.108646 | 0.540615 |  |  |  |  |
| 1.108876 | 0.540592 |  |  |  |  |
| 1.109083 | 0.540362 |  |  |  |  |
| 1.109313 | 0.540845 |  |  |  |  |
| 1.109543 | 0.541121 |  |  |  |  |
| 1.109773 | 0.540845 |  |  |  |  |
| 1.110003 | 0.540776 |  |  |  |  |
| 1.110233 | 0.540891 |  |  |  |  |
| 1.11044 | 0.541236 |  |  |  |  |
| 1.11067 | 0.541098 |  |  |  |  |
| 1.1109 | 0.541006 |  |  |  |  |
| 1.11113 | 0.540868 |  |  |  |  |
| 1.11136 | 0.541305 |  |  |  |  |
| 1.11159 | 0.541213 |  |  |  |  |
| 1.11182 | 0.541052 |  |  |  |  |
| 1.112027 | 0.541282 |  |  |  |  |
| 1.112257 | 0.541351 |  |  |  |  |
| 1.112487 | 0.541489 |  |  |  |  |
| 1.112717 | 0.541581 |  |  |  |  |
| 1.112947 | 0.541282 |  |  |  |  |
| 1.113177 | 0.541489 |  |  |  |  |
| 1.113384 | 0.541857 |  |  |  |  |
| 1.113614 | 0.541627 |  |  |  |  |
| 1.113844 | 0.541673 |  |  |  |  |
| 1.114051 | 0.541443 |  |  |  |  |
| 1.114281 | 0.541857 |  |  |  |  |
| 1.114534 | 0.542179 |  |  |  |  |
| 1.114741 | 0.54165 |  |  |  |  |
| 1.114971 | 0.54188 |  |  |  |  |
| 1.115201 | 0.542202 |  |  |  |  |
| 1.115408 | 0.542041 |  |  |  |  |
| 1.115638 | 0.542225 |  |  |  |  |
| 1.115868 | 0.542087 |  |  |  |  |
| 1.116098 | 0.542018 |  |  |  |  |
| 1.116328 | 0.542363 |  |  |  |  |
| 1.116558 | 0.542317 |  |  |  |  |
| 1.116765 | 0.542018 |  |  |  |  |
| 1.116995 | 0.542386 |  |  |  |  |
| 1.117225 | 0.542501 |  |  |  |  |
| 1.117455 | 0.542317 |  |  |  |  |
| 1.117685 | 0.54234 |  |  |  |  |
| 1.117915 | 0.542708 |  |  |  |  |
| 1.118145 | 0.542501 |  |  |  |  |
| 1.118352 | 0.542363 |  |  |  |  |
| 1.118582 | 0.542823 |  |  |  |  |
| 1.118812 | 0.542731 |  |  |  |  |
| 1.119042 | 0.542869 |  |  |  |  |
| 1.119272 | 0.542915 |  |  |  |  |
| 1.119502 | 0.542961 |  |  |  |  |
| 1.119709 | 0.542892 |  |  |  |  |
| 1.119939 | 0.542915 |  |  |  |  |
| 1.120169 | 0.543122 |  |  |  |  |
| 1.120399 | 0.543007 |  |  |  |  |
| 1.120629 | 0.543168 |  |  |  |  |
| 1.120859 | 0.542938 |  |  |  |  |
| 1.121066 | 0.543168 |  |  |  |  |
| 1.121296 | 0.543306 |  |  |  |  |
| 1.121526 | 0.542869 |  |  |  |  |
| 1.121733 | 0.54303 |  |  |  |  |
| 1.121963 | 0.543398 |  |  |  |  |
| 1.122216 | 0.543513 |  |  |  |  |
| 1.122446 | 0.543398 |  |  |  |  |
| 1.122653 | 0.543651 |  |  |  |  |
| 1.122883 | 0.543582 |  |  |  |  |
| 1.123113 | 0.543651 |  |  |  |  |
| 1.12332 | 0.543743 |  |  |  |  |
| 1.12355 | 0.543697 |  |  |  |  |
| 1.123803 | 0.543674 |  |  |  |  |
| 1.12401 | 0.544019 |  |  |  |  |
| 1.12424 | 0.54395 |  |  |  |  |
| 1.12447 | 0.54395 |  |  |  |  |
| 1.124677 | 0.544019 |  |  |  |  |
| 1.124907 | 0.544203 |  |  |  |  |
| 1.125137 | 0.543927 |  |  |  |  |
| 1.12539 | 0.543996 |  |  |  |  |
| 1.125597 | 0.544042 |  |  |  |  |
| 1.125827 | 0.544341 |  |  |  |  |
| 1.126034 | 0.544249 |  |  |  |  |
| 1.126264 | 0.544157 |  |  |  |  |
| 1.126494 | 0.544272 |  |  |  |  |
| 1.126724 | 0.544594 |  |  |  |  |
| 1.126954 | 0.544525 |  |  |  |  |
| 1.127184 | 0.544502 |  |  |  |  |
| 1.127414 | 0.54464 |  |  |  |  |
| 1.127621 | 0.544571 |  |  |  |  |
| 1.127851 | 0.54464 |  |  |  |  |
| 1.128081 | 0.544893 |  |  |  |  |
| 1.128311 | 0.54441 |  |  |  |  |
| 1.128541 | 0.54487 |  |  |  |  |
| 1.128771 | 0.545008 |  |  |  |  |
| 1.128978 | 0.544939 |  |  |  |  |
| 1.129208 | 0.545031 |  |  |  |  |
| 1.129438 | 0.544824 |  |  |  |  |
| 1.129645 | 0.545146 |  |  |  |  |
| 1.129898 | 0.545399 |  |  |  |  |
| 1.130128 | 0.545192 |  |  |  |  |
| 1.130335 | 0.545284 |  |  |  |  |
| 1.130565 | 0.5451 |  |  |  |  |
| 1.130795 | 0.545652 |  |  |  |  |
| 1.131025 | 0.545652 |  |  |  |  |
| 1.131232 | 0.545376 |  |  |  |  |
| 1.131485 | 0.545123 |  |  |  |  |
| 1.131715 | 0.545744 |  |  |  |  |
| 1.131922 | 0.545537 |  |  |  |  |
| 1.132152 | 0.545537 |  |  |  |  |
| 1.132359 | 0.545813 |  |  |  |  |
| 1.132589 | 0.545859 |  |  |  |  |
| 1.132819 | 0.545928 |  |  |  |  |
| 1.133049 | 0.545698 |  |  |  |  |
| 1.133279 | 0.54602 |  |  |  |  |
| 1.133509 | 0.545859 |  |  |  |  |
| 1.133739 | 0.546135 |  |  |  |  |
| 1.133946 | 0.546135 |  |  |  |  |
| 1.134176 | 0.546066 |  |  |  |  |
| 1.134406 | 0.546135 |  |  |  |  |
| 1.134636 | 0.546526 |  |  |  |  |
| 1.134866 | 0.546204 |  |  |  |  |
| 1.135096 | 0.546043 |  |  |  |  |
| 1.135303 | 0.546204 |  |  |  |  |
| 1.135533 | 0.546526 |  |  |  |  |
| 1.135763 | 0.54648 |  |  |  |  |
| 1.135993 | 0.546549 |  |  |  |  |
| 1.136223 | 0.546595 |  |  |  |  |
| 1.136453 | 0.546572 |  |  |  |  |
| 1.136683 | 0.546779 |  |  |  |  |
| 1.13689 | 0.546986 |  |  |  |  |
| 1.13712 | 0.546549 |  |  |  |  |
| 1.13735 | 0.546848 |  |  |  |  |
| 1.137557 | 0.546871 |  |  |  |  |
| 1.13781 | 0.547055 |  |  |  |  |
| 1.13804 | 0.546779 |  |  |  |  |
| 1.138247 | 0.546894 |  |  |  |  |
| 1.138477 | 0.547193 |  |  |  |  |
| 1.138707 | 0.547285 |  |  |  |  |
| 1.138937 | 0.54717 |  |  |  |  |
| 1.139144 | 0.547101 |  |  |  |  |
| 1.139397 | 0.547262 |  |  |  |  |
| 1.139604 | 0.547377 |  |  |  |  |
| 1.139834 | 0.547331 |  |  |  |  |
| 1.140064 | 0.547055 |  |  |  |  |
| 1.140271 | 0.547492 |  |  |  |  |
| 1.140501 | 0.547469 |  |  |  |  |
| 1.140731 | 0.547354 |  |  |  |  |
| 1.140961 | 0.547446 |  |  |  |  |
| 1.141191 | 0.547791 |  |  |  |  |
| 1.141421 | 0.547952 |  |  |  |  |
| 1.141651 | 0.54763 |  |  |  |  |
| 1.141858 | 0.547584 |  |  |  |  |
| 1.142088 | 0.547354 |  |  |  |  |
| 1.142318 | 0.547998 |  |  |  |  |
| 1.142548 | 0.548159 |  |  |  |  |
| 1.142778 | 0.548021 |  |  |  |  |
| 1.143008 | 0.547883 |  |  |  |  |
| 1.143215 | 0.54786 |  |  |  |  |
| 1.143445 | 0.547906 |  |  |  |  |
| 1.143675 | 0.548044 |  |  |  |  |
| 1.143905 | 0.548274 |  |  |  |  |
| 1.144135 | 0.548297 |  |  |  |  |
| 1.144365 | 0.548274 |  |  |  |  |
| 1.144572 | 0.548021 |  |  |  |  |
| 1.144802 | 0.54809 |  |  |  |  |
| 1.145032 | 0.548182 |  |  |  |  |
| 1.145239 | 0.548481 |  |  |  |  |
| 1.145492 | 0.548504 |  |  |  |  |
| 1.145722 | 0.548251 |  |  |  |  |
| 1.145929 | 0.548274 |  |  |  |  |
| 1.146159 | 0.548688 |  |  |  |  |
| 1.146389 | 0.548573 |  |  |  |  |
| 1.146619 | 0.548573 |  |  |  |  |
| 1.146826 | 0.548596 |  |  |  |  |
| 1.147079 | 0.548711 |  |  |  |  |
| 1.147309 | 0.548849 |  |  |  |  |
| 1.147516 | 0.548757 |  |  |  |  |
| 1.147746 | 0.548826 |  |  |  |  |
| 1.147976 | 0.548757 |  |  |  |  |
| 1.148183 | 0.54901 |  |  |  |  |
| 1.148413 | 0.548918 |  |  |  |  |
| 1.148666 | 0.548872 |  |  |  |  |
| 1.148873 | 0.548987 |  |  |  |  |
| 1.149103 | 0.549171 |  |  |  |  |
| 1.149333 | 0.549217 |  |  |  |  |
| 1.149563 | 0.549033 |  |  |  |  |
| 1.14977 | 0.549102 |  |  |  |  |
| 1.15 | 0.549401 |  |  |  |  |
| 1.15023 | 0.549102 |  |  |  |  |
| 1.15046 | 0.54924 |  |  |  |  |
| 1.15069 | 0.549355 |  |  |  |  |
| 1.150897 | 0.549516 |  |  |  |  |
| 1.151127 | 0.549516 |  |  |  |  |
| 1.151357 | 0.549493 |  |  |  |  |
| 1.151587 | 0.549447 |  |  |  |  |
| 1.151817 | 0.549539 |  |  |  |  |
| 1.152047 | 0.549746 |  |  |  |  |
| 1.152277 | 0.549907 |  |  |  |  |
| 1.152484 | 0.549723 |  |  |  |  |
| 1.152714 | 0.549539 |  |  |  |  |
| 1.152944 | 0.549861 |  |  |  |  |
| 1.153174 | 0.550068 |  |  |  |  |
| 1.153404 | 0.549815 |  |  |  |  |
| 1.153634 | 0.549907 |  |  |  |  |
| 1.153841 | 0.550459 |  |  |  |  |
| 1.154071 | 0.550413 |  |  |  |  |
| 1.154301 | 0.550367 |  |  |  |  |
| 1.154508 | 0.550022 |  |  |  |  |
| 1.154761 | 0.550137 |  |  |  |  |
| 1.154991 | 0.550367 |  |  |  |  |
| 1.155221 | 0.550321 |  |  |  |  |
| 1.155428 | 0.550321 |  |  |  |  |
| 1.155658 | 0.550321 |  |  |  |  |
| 1.155888 | 0.550597 |  |  |  |  |
| 1.156095 | 0.550528 |  |  |  |  |
| 1.156325 | 0.550574 |  |  |  |  |
| 1.156555 | 0.550597 |  |  |  |  |
| 1.156785 | 0.550666 |  |  |  |  |
| 1.157015 | 0.550735 |  |  |  |  |
| 1.157245 | 0.550873 |  |  |  |  |
| 1.157452 | 0.550758 |  |  |  |  |
| 1.157682 | 0.550919 |  |  |  |  |
| 1.157912 | 0.551172 |  |  |  |  |
| 1.158142 | 0.551149 |  |  |  |  |
| 1.158372 | 0.550758 |  |  |  |  |
| 1.158602 | 0.55085 |  |  |  |  |
| 1.158809 | 0.551241 |  |  |  |  |
| 1.159039 | 0.551034 |  |  |  |  |
| 1.159269 | 0.551172 |  |  |  |  |
| 1.159499 | 0.551126 |  |  |  |  |
| 1.159729 | 0.551241 |  |  |  |  |
| 1.159959 | 0.551563 |  |  |  |  |
| 1.160189 | 0.551241 |  |  |  |  |
| 1.160396 | 0.55131 |  |  |  |  |
| 1.160626 | 0.551402 |  |  |  |  |
| 1.160856 | 0.551632 |  |  |  |  |
| 1.161086 | 0.551747 |  |  |  |  |
| 1.161316 | 0.551586 |  |  |  |  |
| 1.161546 | 0.551563 |  |  |  |  |
| 1.161753 | 0.551862 |  |  |  |  |
| 1.161983 | 0.551793 |  |  |  |  |
| 1.162213 | 0.551586 |  |  |  |  |
| 1.16242 | 0.551701 |  |  |  |  |
| 1.162673 | 0.551632 |  |  |  |  |
| 1.162903 | 0.552 |  |  |  |  |
| 1.16311 | 0.552023 |  |  |  |  |
| 1.16334 | 0.551931 |  |  |  |  |
| 1.16357 | 0.552184 |  |  |  |  |
| 1.163777 | 0.552 |  |  |  |  |
| 1.164007 | 0.552184 |  |  |  |  |
| 1.16426 | 0.552115 |  |  |  |  |
| 1.164467 | 0.552322 |  |  |  |  |
| 1.164697 | 0.552161 |  |  |  |  |
| 1.164927 | 0.552529 |  |  |  |  |
| 1.165157 | 0.55223 |  |  |  |  |
| 1.165364 | 0.552506 |  |  |  |  |
| 1.165594 | 0.552529 |  |  |  |  |
| 1.165847 | 0.552552 |  |  |  |  |
| 1.166054 | 0.55246 |  |  |  |  |
| 1.166284 | 0.552598 |  |  |  |  |
| 1.166514 | 0.552644 |  |  |  |  |
| 1.166721 | 0.552736 |  |  |  |  |
| 1.166951 | 0.552437 |  |  |  |  |
| 1.167181 | 0.552713 |  |  |  |  |
| 1.167411 | 0.552851 |  |  |  |  |
| 1.167641 | 0.552989 |  |  |  |  |
| 1.167871 | 0.552989 |  |  |  |  |
| 1.168078 | 0.552782 |  |  |  |  |
| 1.168308 | 0.552989 |  |  |  |  |
| 1.168538 | 0.552966 |  |  |  |  |
| 1.168768 | 0.553127 |  |  |  |  |
| 1.168998 | 0.553035 |  |  |  |  |
| 1.169228 | 0.552874 |  |  |  |  |
| 1.169435 | 0.553058 |  |  |  |  |
| 1.169665 | 0.553311 |  |  |  |  |
| 1.169895 | 0.552966 |  |  |  |  |
| 1.170102 | 0.552989 |  |  |  |  |
| 1.170355 | 0.55338 |  |  |  |  |
| 1.170585 | 0.553357 |  |  |  |  |
| 1.170815 | 0.553357 |  |  |  |  |
| 1.171022 | 0.553127 |  |  |  |  |
| 1.171252 | 0.553242 |  |  |  |  |
| 1.171482 | 0.553472 |  |  |  |  |
| 1.171689 | 0.553403 |  |  |  |  |
| 1.171942 | 0.553472 |  |  |  |  |
| 1.172172 | 0.553587 |  |  |  |  |
| 1.172379 | 0.553817 |  |  |  |  |
| 1.172609 | 0.553817 |  |  |  |  |
| 1.172839 | 0.553633 |  |  |  |  |
| 1.173046 | 0.553495 |  |  |  |  |
| 1.173276 | 0.554093 |  |  |  |  |
| 1.173529 | 0.553955 |  |  |  |  |
| 1.173759 | 0.553863 |  |  |  |  |
| 1.173966 | 0.553702 |  |  |  |  |
| 1.174196 | 0.554231 |  |  |  |  |
| 1.174403 | 0.553886 |  |  |  |  |
| 1.174633 | 0.55407 |  |  |  |  |
| 1.174863 | 0.554116 |  |  |  |  |
| 1.175093 | 0.554231 |  |  |  |  |
| 1.175323 | 0.554093 |  |  |  |  |
| 1.175553 | 0.554346 |  |  |  |  |
| 1.17576 | 0.554208 |  |  |  |  |
| 1.17599 | 0.554484 |  |  |  |  |
| 1.17622 | 0.554599 |  |  |  |  |
| 1.17645 | 0.554783 |  |  |  |  |
| 1.17668 | 0.554714 |  |  |  |  |
| 1.17691 | 0.554553 |  |  |  |  |
| 1.17714 | 0.554668 |  |  |  |  |
| 1.177347 | 0.554806 |  |  |  |  |
| 1.177577 | 0.554898 |  |  |  |  |
| 1.177807 | 0.554898 |  |  |  |  |
| 1.178037 | 0.554829 |  |  |  |  |
| 1.178267 | 0.555197 |  |  |  |  |
| 1.178497 | 0.554967 |  |  |  |  |
| 1.178704 | 0.555128 |  |  |  |  |
| 1.178934 | 0.555197 |  |  |  |  |
| 1.179164 | 0.555381 |  |  |  |  |
| 1.179394 | 0.555105 |  |  |  |  |
| 1.179601 | 0.554944 |  |  |  |  |
| 1.179854 | 0.555243 |  |  |  |  |
| 1.180061 | 0.555312 |  |  |  |  |
| 1.180291 | 0.555335 |  |  |  |  |
| 1.180521 | 0.555289 |  |  |  |  |
| 1.180728 | 0.555243 |  |  |  |  |
| 1.180958 | 0.555381 |  |  |  |  |
| 1.181188 | 0.555726 |  |  |  |  |
| 1.181441 | 0.555427 |  |  |  |  |
| 1.181648 | 0.555381 |  |  |  |  |
| 1.181878 | 0.555565 |  |  |  |  |
| 1.182108 | 0.555749 |  |  |  |  |
| 1.182315 | 0.555634 |  |  |  |  |
| 1.182545 | 0.555772 |  |  |  |  |
| 1.182775 | 0.555703 |  |  |  |  |
| 1.183005 | 0.55614 |  |  |  |  |
| 1.183235 | 0.555956 |  |  |  |  |
| 1.183465 | 0.555749 |  |  |  |  |
| 1.183672 | 0.555864 |  |  |  |  |
| 1.183902 | 0.556278 |  |  |  |  |
| 1.184132 | 0.556094 |  |  |  |  |
| 1.184362 | 0.556186 |  |  |  |  |
| 1.184592 | 0.556209 |  |  |  |  |
| 1.184822 | 0.556186 |  |  |  |  |
| 1.185052 | 0.556278 |  |  |  |  |
| 1.185259 | 0.556301 |  |  |  |  |
| 1.185489 | 0.556048 |  |  |  |  |
| 1.185719 | 0.556186 |  |  |  |  |
| 1.185949 | 0.556508 |  |  |  |  |
| 1.186179 | 0.556416 |  |  |  |  |
| 1.186409 | 0.556278 |  |  |  |  |
| 1.186616 | 0.556439 |  |  |  |  |
| 1.186846 | 0.556876 |  |  |  |  |
| 1.187076 | 0.556439 |  |  |  |  |
| 1.187283 | 0.5566 |  |  |  |  |
| 1.187536 | 0.556531 |  |  |  |  |
| 1.187766 | 0.55637 |  |  |  |  |
| 1.187973 | 0.556531 |  |  |  |  |
| 1.188203 | 0.556738 |  |  |  |  |
| 1.188433 | 0.556738 |  |  |  |  |
| 1.18864 | 0.556899 |  |  |  |  |
| 1.18887 | 0.556669 |  |  |  |  |
| 1.189123 | 0.55683 |  |  |  |  |
| 1.189353 | 0.556876 |  |  |  |  |
| 1.18956 | 0.556738 |  |  |  |  |
| 1.18979 | 0.557129 |  |  |  |  |
| 1.19002 | 0.557014 |  |  |  |  |
| 1.190227 | 0.556807 |  |  |  |  |
| 1.190457 | 0.557175 |  |  |  |  |
| 1.19071 | 0.557244 |  |  |  |  |
| 1.190917 | 0.557198 |  |  |  |  |
| 1.191147 | 0.557152 |  |  |  |  |
| 1.191377 | 0.557267 |  |  |  |  |
| 1.191584 | 0.557359 |  |  |  |  |
| 1.191814 | 0.557382 |  |  |  |  |
| 1.192044 | 0.557474 |  |  |  |  |
| 1.192274 | 0.557543 |  |  |  |  |
| 1.192504 | 0.557543 |  |  |  |  |
| 1.192734 | 0.557566 |  |  |  |  |
| 1.192941 | 0.557865 |  |  |  |  |
| 1.193171 | 0.557451 |  |  |  |  |
| 1.193401 | 0.557819 |  |  |  |  |
| 1.193631 | 0.558003 |  |  |  |  |
| 1.193861 | 0.55775 |  |  |  |  |
| 1.194091 | 0.557819 |  |  |  |  |
| 1.194298 | 0.557842 |  |  |  |  |
| 1.194528 | 0.558256 |  |  |  |  |
| 1.194758 | 0.558187 |  |  |  |  |
| 1.194988 | 0.558118 |  |  |  |  |
| 1.195218 | 0.55798 |  |  |  |  |
| 1.195448 | 0.558233 |  |  |  |  |
| 1.195678 | 0.558049 |  |  |  |  |
| 1.195885 | 0.558095 |  |  |  |  |
| 1.196115 | 0.558394 |  |  |  |  |
| 1.196345 | 0.558348 |  |  |  |  |
| 1.196552 | 0.558371 |  |  |  |  |
| 1.196805 | 0.558279 |  |  |  |  |
| 1.197035 | 0.558279 |  |  |  |  |
| 1.197242 | 0.558302 |  |  |  |  |
| 1.197472 | 0.558463 |  |  |  |  |
| 1.197702 | 0.558601 |  |  |  |  |
| 1.197909 | 0.558716 |  |  |  |  |
| 1.198139 | 0.558486 |  |  |  |  |
| 1.198369 | 0.55867 |  |  |  |  |
| 1.198599 | 0.558693 |  |  |  |  |
| 1.198829 | 0.558624 |  |  |  |  |
| 1.199059 | 0.558601 |  |  |  |  |
| 1.199266 | 0.5589 |  |  |  |  |
| 1.199496 | 0.558877 |  |  |  |  |
| 1.199726 | 0.558969 |  |  |  |  |
| 1.199956 | 0.55867 |  |  |  |  |
| 1.200186 | 0.558946 |  |  |  |  |
| 1.200416 | 0.55913 |  |  |  |  |
| 1.200646 | 0.558923 |  |  |  |  |
| 1.200853 | 0.559015 |  |  |  |  |
| 1.201083 | 0.558969 |  |  |  |  |
| 1.201313 | 0.559084 |  |  |  |  |
| 1.201543 | 0.559107 |  |  |  |  |
| 1.201773 | 0.559337 |  |  |  |  |
| 1.202003 | 0.559222 |  |  |  |  |
| 1.20221 | 0.559337 |  |  |  |  |
| 1.20244 | 0.559337 |  |  |  |  |
| 1.20267 | 0.559475 |  |  |  |  |
| 1.2029 | 0.559636 |  |  |  |  |
| 1.20313 | 0.559728 |  |  |  |  |
| 1.20336 | 0.559843 |  |  |  |  |
| 1.20359 | 0.559567 |  |  |  |  |
| 1.203797 | 0.559429 |  |  |  |  |
| 1.204027 | 0.559682 |  |  |  |  |
| 1.204257 | 0.559843 |  |  |  |  |
| 1.204464 | 0.559728 |  |  |  |  |
| 1.204717 | 0.559682 |  |  |  |  |
| 1.204924 | 0.559613 |  |  |  |  |
| 1.205154 | 0.559797 |  |  |  |  |
| 1.205384 | 0.560027 |  |  |  |  |
| 1.205614 | 0.559728 |  |  |  |  |
| 1.205821 | 0.55982 |  |  |  |  |
| 1.206051 | 0.559728 |  |  |  |  |
| 1.206304 | 0.560234 |  |  |  |  |
| 1.206511 | 0.560119 |  |  |  |  |
| 1.206741 | 0.560234 |  |  |  |  |
| 1.206971 | 0.560165 |  |  |  |  |
| 1.207178 | 0.560441 |  |  |  |  |
| 1.207408 | 0.560073 |  |  |  |  |
| 1.207638 | 0.56028 |  |  |  |  |
| 1.207868 | 0.560326 |  |  |  |  |
| 1.208098 | 0.560533 |  |  |  |  |
| 1.208328 | 0.560625 |  |  |  |  |
| 1.208558 | 0.560464 |  |  |  |  |
| 1.208765 | 0.560372 |  |  |  |  |
| 1.208995 | 0.560326 |  |  |  |  |
| 1.209225 | 0.560556 |  |  |  |  |
| 1.209455 | 0.56074 |  |  |  |  |
| 1.209685 | 0.560602 |  |  |  |  |
| 1.209915 | 0.560763 |  |  |  |  |
| 1.210122 | 0.560832 |  |  |  |  |
| 1.210352 | 0.560947 |  |  |  |  |
| 1.210582 | 0.560763 |  |  |  |  |
| 1.210812 | 0.561062 |  |  |  |  |
| 1.211042 | 0.561062 |  |  |  |  |
| 1.211272 | 0.560763 |  |  |  |  |
| 1.211479 | 0.561315 |  |  |  |  |
| 1.211709 | 0.561062 |  |  |  |  |
| 1.211939 | 0.561131 |  |  |  |  |
| 1.212146 | 0.5612 |  |  |  |  |
| 1.212399 | 0.561131 |  |  |  |  |
| 1.212629 | 0.561223 |  |  |  |  |
| 1.212836 | 0.561522 |  |  |  |  |
| 1.213066 | 0.561407 |  |  |  |  |
| 1.213296 | 0.561476 |  |  |  |  |
| 1.213503 | 0.56143 |  |  |  |  |
| 1.213733 | 0.561361 |  |  |  |  |
| 1.213986 | 0.561913 |  |  |  |  |
| 1.214216 | 0.561522 |  |  |  |  |
| 1.214423 | 0.561476 |  |  |  |  |
| 1.214653 | 0.56143 |  |  |  |  |
| 1.214883 | 0.561913 |  |  |  |  |
| 1.21509 | 0.561752 |  |  |  |  |
| 1.21532 | 0.561867 |  |  |  |  |
| 1.215573 | 0.561683 |  |  |  |  |
| 1.21578 | 0.561982 |  |  |  |  |
| 1.21601 | 0.56212 |  |  |  |  |
| 1.21624 | 0.561844 |  |  |  |  |
| 1.216447 | 0.562005 |  |  |  |  |
| 1.216677 | 0.562074 |  |  |  |  |
| 1.216907 | 0.562051 |  |  |  |  |
| 1.217137 | 0.562212 |  |  |  |  |
| 1.217367 | 0.562373 |  |  |  |  |
| 1.217597 | 0.56212 |  |  |  |  |
| 1.217804 | 0.56235 |  |  |  |  |
| 1.218034 | 0.56212 |  |  |  |  |
| 1.218264 | 0.562442 |  |  |  |  |
| 1.218494 | 0.562281 |  |  |  |  |
| 1.218724 | 0.562557 |  |  |  |  |
| 1.218954 | 0.562534 |  |  |  |  |
| 1.219184 | 0.56235 |  |  |  |  |
| 1.219391 | 0.562396 |  |  |  |  |
| 1.219621 | 0.562787 |  |  |  |  |
| 1.219851 | 0.562856 |  |  |  |  |
| 1.220081 | 0.562718 |  |  |  |  |
| 1.220311 | 0.562649 |  |  |  |  |
| 1.220541 | 0.562488 |  |  |  |  |
| 1.220748 | 0.562787 |  |  |  |  |
| 1.220978 | 0.562925 |  |  |  |  |
| 1.221208 | 0.562787 |  |  |  |  |
| 1.221415 | 0.562994 |  |  |  |  |
| 1.221645 | 0.56304 |  |  |  |  |
| 1.221898 | 0.563224 |  |  |  |  |
| 1.222128 | 0.563063 |  |  |  |  |
| 1.222335 | 0.562902 |  |  |  |  |
| 1.222565 | 0.563408 |  |  |  |  |
| 1.222772 | 0.563178 |  |  |  |  |
| 1.223002 | 0.563178 |  |  |  |  |
| 1.223232 | 0.56327 |  |  |  |  |
| 1.223462 | 0.563293 |  |  |  |  |
| 1.223692 | 0.563431 |  |  |  |  |
| 1.223922 | 0.563431 |  |  |  |  |
| 1.224152 | 0.563477 |  |  |  |  |
| 1.224359 | 0.563431 |  |  |  |  |
| 1.224589 | 0.563569 |  |  |  |  |
| 1.224819 | 0.563431 |  |  |  |  |
| 1.225049 | 0.563546 |  |  |  |  |
| 1.225279 | 0.5635 |  |  |  |  |
| 1.225509 | 0.563822 |  |  |  |  |
| 1.225716 | 0.563937 |  |  |  |  |
| 1.225946 | 0.563914 |  |  |  |  |
| 1.226176 | 0.56373 |  |  |  |  |
| 1.226406 | 0.563983 |  |  |  |  |
| 1.226636 | 0.563983 |  |  |  |  |
| 1.226866 | 0.563914 |  |  |  |  |
| 1.227073 | 0.563822 |  |  |  |  |
| 1.227303 | 0.563937 |  |  |  |  |
| 1.227533 | 0.564259 |  |  |  |  |
| 1.227763 | 0.563868 |  |  |  |  |
| 1.227993 | 0.564167 |  |  |  |  |
| 1.228223 | 0.564075 |  |  |  |  |
| 1.228453 | 0.564351 |  |  |  |  |
| 1.22866 | 0.564512 |  |  |  |  |
| 1.22889 | 0.564374 |  |  |  |  |
| 1.22912 | 0.56442 |  |  |  |  |
| 1.229327 | 0.564765 |  |  |  |  |
| 1.22958 | 0.564535 |  |  |  |  |
| 1.22981 | 0.564167 |  |  |  |  |
| 1.230017 | 0.564351 |  |  |  |  |
| 1.230247 | 0.564512 |  |  |  |  |
| 1.230477 | 0.564696 |  |  |  |  |
| 1.230684 | 0.564535 |  |  |  |  |
| 1.230914 | 0.564512 |  |  |  |  |
| 1.231167 | 0.564903 |  |  |  |  |
| 1.231374 | 0.564903 |  |  |  |  |
| 1.231604 | 0.56488 |  |  |  |  |
| 1.231834 | 0.564719 |  |  |  |  |
| 1.232041 | 0.56488 |  |  |  |  |
| 1.232271 | 0.564926 |  |  |  |  |
| 1.232501 | 0.565087 |  |  |  |  |
| 1.232754 | 0.564857 |  |  |  |  |
| 1.232961 | 0.56511 |  |  |  |  |
| 1.233191 | 0.565087 |  |  |  |  |
| 1.233421 | 0.565087 |  |  |  |  |
| 1.233628 | 0.565087 |  |  |  |  |
| 1.233858 | 0.565248 |  |  |  |  |
| 1.234088 | 0.565179 |  |  |  |  |
| 1.234318 | 0.565363 |  |  |  |  |
| 1.234548 | 0.565294 |  |  |  |  |
| 1.234778 | 0.565363 |  |  |  |  |
| 1.234985 | 0.565455 |  |  |  |  |
| 1.235215 | 0.565639 |  |  |  |  |
| 1.235445 | 0.565823 |  |  |  |  |
| 1.235675 | 0.565547 |  |  |  |  |
| 1.235905 | 0.565225 |  |  |  |  |
| 1.236135 | 0.565639 |  |  |  |  |
| 1.236342 | 0.565685 |  |  |  |  |
| 1.236572 | 0.565708 |  |  |  |  |
| 1.236802 | 0.565754 |  |  |  |  |
| 1.237009 | 0.565708 |  |  |  |  |
| 1.237262 | 0.566053 |  |  |  |  |
| 1.237492 | 0.565846 |  |  |  |  |
| 1.237699 | 0.565708 |  |  |  |  |
| 1.237929 | 0.566076 |  |  |  |  |
| 1.238159 | 0.565961 |  |  |  |  |
| 1.238389 | 0.566329 |  |  |  |  |
| 1.238596 | 0.566214 |  |  |  |  |
| 1.238849 | 0.566076 |  |  |  |  |
| 1.239079 | 0.566237 |  |  |  |  |
| 1.239286 | 0.566122 |  |  |  |  |
| 1.239516 | 0.566053 |  |  |  |  |
| 1.239746 | 0.566053 |  |  |  |  |
| 1.239953 | 0.566076 |  |  |  |  |
| 1.240183 | 0.566145 |  |  |  |  |
| 1.240413 | 0.566375 |  |  |  |  |
| 1.240643 | 0.566191 |  |  |  |  |
| 1.240873 | 0.566237 |  |  |  |  |
| 1.241103 | 0.566398 |  |  |  |  |
| 1.24131 | 0.56649 |  |  |  |  |
| 1.24154 | 0.566398 |  |  |  |  |
| 1.24177 | 0.566421 |  |  |  |  |
| 1.242 | 0.566605 |  |  |  |  |
| 1.24223 | 0.566582 |  |  |  |  |
| 1.24246 | 0.566582 |  |  |  |  |
| 1.242667 | 0.566582 |  |  |  |  |
| 1.242897 | 0.566904 |  |  |  |  |
| 1.243127 | 0.566881 |  |  |  |  |
| 1.243357 | 0.566812 |  |  |  |  |
| 1.243587 | 0.566697 |  |  |  |  |
| 1.243817 | 0.567019 |  |  |  |  |
| 1.244047 | 0.566927 |  |  |  |  |
| 1.244254 | 0.566743 |  |  |  |  |
| 1.244484 | 0.566927 |  |  |  |  |
| 1.244714 | 0.566996 |  |  |  |  |
| 1.244944 | 0.566743 |  |  |  |  |
| 1.245174 | 0.567088 |  |  |  |  |
| 1.245404 | 0.567272 |  |  |  |  |
| 1.245611 | 0.567088 |  |  |  |  |
| 1.245841 | 0.56741 |  |  |  |  |
| 1.246071 | 0.567456 |  |  |  |  |
| 1.246301 | 0.567226 |  |  |  |  |
| 1.246508 | 0.567387 |  |  |  |  |
| 1.246761 | 0.56741 |  |  |  |  |
| 1.246968 | 0.567594 |  |  |  |  |
| 1.247198 | 0.567525 |  |  |  |  |
| 1.247428 | 0.567203 |  |  |  |  |
| 1.247635 | 0.567801 |  |  |  |  |
| 1.247865 | 0.567709 |  |  |  |  |
| 1.248095 | 0.567571 |  |  |  |  |
| 1.248348 | 0.567525 |  |  |  |  |
| 1.248555 | 0.567732 |  |  |  |  |
| 1.248785 | 0.567548 |  |  |  |  |
| 1.249015 | 0.567686 |  |  |  |  |
| 1.249222 | 0.567502 |  |  |  |  |
| 1.249452 | 0.567663 |  |  |  |  |
| 1.249682 | 0.568077 |  |  |  |  |
| 1.249912 | 0.56787 |  |  |  |  |
| 1.250142 | 0.567916 |  |  |  |  |
| 1.250372 | 0.567916 |  |  |  |  |
| 1.250579 | 0.568008 |  |  |  |  |
| 1.250809 | 0.568146 |  |  |  |  |
| 1.251039 | 0.568215 |  |  |  |  |
| 1.251269 | 0.568353 |  |  |  |  |
| 1.251499 | 0.568123 |  |  |  |  |
| 1.251729 | 0.568353 |  |  |  |  |
| 1.251959 | 0.568169 |  |  |  |  |
| 1.252166 | 0.568422 |  |  |  |  |
| 1.252396 | 0.56856 |  |  |  |  |
| 1.252626 | 0.568399 |  |  |  |  |
| 1.252856 | 0.56833 |  |  |  |  |
| 1.253086 | 0.568284 |  |  |  |  |
| 1.253316 | 0.568422 |  |  |  |  |
| 1.253523 | 0.56879 |  |  |  |  |
| 1.253753 | 0.568468 |  |  |  |  |
| 1.253983 | 0.568652 |  |  |  |  |
| 1.25419 | 0.56879 |  |  |  |  |
| 1.254443 | 0.568721 |  |  |  |  |
| 1.254673 | 0.568951 |  |  |  |  |
| 1.25488 | 0.568675 |  |  |  |  |
| 1.25511 | 0.568629 |  |  |  |  |
| 1.25534 | 0.568514 |  |  |  |  |
| 1.255547 | 0.568767 |  |  |  |  |
| 1.255777 | 0.568997 |  |  |  |  |
| 1.25603 | 0.568767 |  |  |  |  |
| 1.256237 | 0.568606 |  |  |  |  |
| 1.256467 | 0.569066 |  |  |  |  |
| 1.256697 | 0.56902 |  |  |  |  |
| 1.256927 | 0.568951 |  |  |  |  |
| 1.257134 | 0.568997 |  |  |  |  |
| 1.257364 | 0.56925 |  |  |  |  |
| 1.257617 | 0.569135 |  |  |  |  |
| 1.257824 | 0.569043 |  |  |  |  |
| 1.258054 | 0.568905 |  |  |  |  |
| 1.258284 | 0.569526 |  |  |  |  |
| 1.258491 | 0.56948 |  |  |  |  |
| 1.258721 | 0.569296 |  |  |  |  |
| 1.258951 | 0.56925 |  |  |  |  |
| 1.259181 | 0.569411 |  |  |  |  |
| 1.259411 | 0.569664 |  |  |  |  |
| 1.259641 | 0.569434 |  |  |  |  |
| 1.259848 | 0.569388 |  |  |  |  |
| 1.260078 | 0.569503 |  |  |  |  |
| 1.260308 | 0.569756 |  |  |  |  |
| 1.260538 | 0.569733 |  |  |  |  |
| 1.260768 | 0.569917 |  |  |  |  |
| 1.260998 | 0.569687 |  |  |  |  |
| 1.261205 | 0.569756 |  |  |  |  |
| 1.261435 | 0.56994 |  |  |  |  |
| 1.261665 | 0.569733 |  |  |  |  |
| 1.261872 | 0.569871 |  |  |  |  |
| 1.262125 | 0.569825 |  |  |  |  |
| 1.262355 | 0.569871 |  |  |  |  |
| 1.262585 | 0.570009 |  |  |  |  |
| 1.262792 | 0.569963 |  |  |  |  |
| 1.263022 | 0.570124 |  |  |  |  |
| 1.263252 | 0.570239 |  |  |  |  |
| 1.263459 | 0.570055 |  |  |  |  |
| 1.263712 | 0.569894 |  |  |  |  |
| 1.263942 | 0.570124 |  |  |  |  |
| 1.264149 | 0.570147 |  |  |  |  |
| 1.264379 | 0.570423 |  |  |  |  |
| 1.264609 | 0.570216 |  |  |  |  |
| 1.264816 | 0.570216 |  |  |  |  |
| 1.265046 | 0.570699 |  |  |  |  |
| 1.265276 | 0.570584 |  |  |  |  |
| 1.265506 | 0.570768 |  |  |  |  |
| 1.265736 | 0.570561 |  |  |  |  |
| 1.265966 | 0.570446 |  |  |  |  |
| 1.266173 | 0.570883 |  |  |  |  |
| 1.266403 | 0.570607 |  |  |  |  |
| 1.266633 | 0.570584 |  |  |  |  |
| 1.266863 | 0.570561 |  |  |  |  |
| 1.267093 | 0.570814 |  |  |  |  |
| 1.267323 | 0.570906 |  |  |  |  |
| 1.267553 | 0.570607 |  |  |  |  |
| 1.26776 | 0.570584 |  |  |  |  |
| 1.26799 | 0.570998 |  |  |  |  |
| 1.26822 | 0.570952 |  |  |  |  |
| 1.26845 | 0.571366 |  |  |  |  |
| 1.26868 | 0.57109 |  |  |  |  |
| 1.26891 | 0.571113 |  |  |  |  |
| 1.269117 | 0.571389 |  |  |  |  |
| 1.269347 | 0.571182 |  |  |  |  |
| 1.269577 | 0.570975 |  |  |  |  |
| 1.269784 | 0.57132 |  |  |  |  |
| 1.270037 | 0.57155 |  |  |  |  |
| 1.270267 | 0.571343 |  |  |  |  |
| 1.270497 | 0.571458 |  |  |  |  |
| 1.270704 | 0.571458 |  |  |  |  |
| 1.270934 | 0.571596 |  |  |  |  |
| 1.271141 | 0.571573 |  |  |  |  |
| 1.271371 | 0.571366 |  |  |  |  |
| 1.271624 | 0.571297 |  |  |  |  |
| 1.271854 | 0.571711 |  |  |  |  |
| 1.272061 | 0.571665 |  |  |  |  |
| 1.272291 | 0.571642 |  |  |  |  |
| 1.272498 | 0.571665 |  |  |  |  |
| 1.272728 | 0.571757 |  |  |  |  |
| 1.272958 | 0.571619 |  |  |  |  |
| 1.273211 | 0.571826 |  |  |  |  |
| 1.273418 | 0.571619 |  |  |  |  |
| 1.273648 | 0.571895 |  |  |  |  |
| 1.273878 | 0.572033 |  |  |  |  |
| 1.274085 | 0.57201 |  |  |  |  |
| 1.274315 | 0.572056 |  |  |  |  |
| 1.274545 | 0.571918 |  |  |  |  |
| 1.274775 | 0.572079 |  |  |  |  |
| 1.275005 | 0.572263 |  |  |  |  |
| 1.275235 | 0.572033 |  |  |  |  |
| 1.275442 | 0.571918 |  |  |  |  |
| 1.275672 | 0.571941 |  |  |  |  |
| 1.275902 | 0.572148 |  |  |  |  |
| 1.276132 | 0.572171 |  |  |  |  |
| 1.276362 | 0.572286 |  |  |  |  |
| 1.276592 | 0.572194 |  |  |  |  |
| 1.276822 | 0.572539 |  |  |  |  |
| 1.277029 | 0.572654 |  |  |  |  |
| 1.277259 | 0.572332 |  |  |  |  |
| 1.277466 | 0.572608 |  |  |  |  |
| 1.277719 | 0.572746 |  |  |  |  |
| 1.277949 | 0.572562 |  |  |  |  |
| 1.278179 | 0.572723 |  |  |  |  |
| 1.278386 | 0.572355 |  |  |  |  |
| 1.278616 | 0.572723 |  |  |  |  |
| 1.278846 | 0.572631 |  |  |  |  |
| 1.279053 | 0.572562 |  |  |  |  |
| 1.279306 | 0.572723 |  |  |  |  |
| 1.279536 | 0.572654 |  |  |  |  |
| 1.279743 | 0.572884 |  |  |  |  |
| 1.279973 | 0.572838 |  |  |  |  |
| 1.280203 | 0.572769 |  |  |  |  |
| 1.28041 | 0.572861 |  |  |  |  |
| 1.28064 | 0.573068 |  |  |  |  |
| 1.280893 | 0.57293 |  |  |  |  |
| 1.281123 | 0.57316 |  |  |  |  |
| 1.28133 | 0.573022 |  |  |  |  |
| 1.28156 | 0.57339 |  |  |  |  |
| 1.28179 | 0.57362 |  |  |  |  |
| 1.281997 | 0.573252 |  |  |  |  |
| 1.282227 | 0.573298 |  |  |  |  |
| 1.282457 | 0.573229 |  |  |  |  |
| 1.282687 | 0.573367 |  |  |  |  |
| 1.282917 | 0.573344 |  |  |  |  |
| 1.283147 | 0.573229 |  |  |  |  |
| 1.283354 | 0.573183 |  |  |  |  |
| 1.283584 | 0.573551 |  |  |  |  |
| 1.283814 | 0.573574 |  |  |  |  |
| 1.284044 | 0.573275 |  |  |  |  |
| 1.284274 | 0.57339 |  |  |  |  |
| 1.284504 | 0.573482 |  |  |  |  |
| 1.284711 | 0.573689 |  |  |  |  |
| 1.284941 | 0.573712 |  |  |  |  |
| 1.285171 | 0.573551 |  |  |  |  |
| 1.285401 | 0.573758 |  |  |  |  |
| 1.285631 | 0.573896 |  |  |  |  |
| 1.285861 | 0.573505 |  |  |  |  |
| 1.286068 | 0.573942 |  |  |  |  |
| 1.286298 | 0.573804 |  |  |  |  |
| 1.286528 | 0.573965 |  |  |  |  |
| 1.286758 | 0.574057 |  |  |  |  |
| 1.286988 | 0.573942 |  |  |  |  |
| 1.287218 | 0.573942 |  |  |  |  |
| 1.287448 | 0.57408 |  |  |  |  |
| 1.287655 | 0.574057 |  |  |  |  |
| 1.287885 | 0.574264 |  |  |  |  |
| 1.288115 | 0.574218 |  |  |  |  |
| 1.288322 | 0.573988 |  |  |  |  |
| 1.288552 | 0.574402 |  |  |  |  |
| 1.288805 | 0.574333 |  |  |  |  |
| 1.289012 | 0.574356 |  |  |  |  |
| 1.289242 | 0.574356 |  |  |  |  |
| 1.289472 | 0.574678 |  |  |  |  |
| 1.289679 | 0.574287 |  |  |  |  |
| 1.289909 | 0.57431 |  |  |  |  |
| 1.290139 | 0.574402 |  |  |  |  |
| 1.290369 | 0.574471 |  |  |  |  |
| 1.290599 | 0.574448 |  |  |  |  |
| 1.290829 | 0.574563 |  |  |  |  |
| 1.291036 | 0.57454 |  |  |  |  |
| 1.291266 | 0.57454 |  |  |  |  |
| 1.291496 | 0.574678 |  |  |  |  |
| 1.291726 | 0.574609 |  |  |  |  |
| 1.291956 | 0.574701 |  |  |  |  |
| 1.292186 | 0.574701 |  |  |  |  |
| 1.292416 | 0.574839 |  |  |  |  |
| 1.292623 | 0.574793 |  |  |  |  |
| 1.292853 | 0.574701 |  |  |  |  |
| 1.293083 | 0.574931 |  |  |  |  |
| 1.293313 | 0.574816 |  |  |  |  |
| 1.293543 | 0.575069 |  |  |  |  |
| 1.293773 | 0.574517 |  |  |  |  |
| 1.29398 | 0.574908 |  |  |  |  |
| 1.29421 | 0.575069 |  |  |  |  |
| 1.29444 | 0.575069 |  |  |  |  |
| 1.294647 | 0.574977 |  |  |  |  |
| 1.2949 | 0.574885 |  |  |  |  |
| 1.29513 | 0.575023 |  |  |  |  |
| 1.295337 | 0.575276 |  |  |  |  |
| 1.295567 | 0.575069 |  |  |  |  |
| 1.295797 | 0.575 |  |  |  |  |
| 1.296004 | 0.575253 |  |  |  |  |
| 1.296234 | 0.57546 |  |  |  |  |
| 1.296487 | 0.575437 |  |  |  |  |
| 1.296694 | 0.575276 |  |  |  |  |
| 1.296924 | 0.575368 |  |  |  |  |
| 1.297154 | 0.575667 |  |  |  |  |
| 1.297384 | 0.57523 |  |  |  |  |
| 1.297591 | 0.575299 |  |  |  |  |
| 1.297821 | 0.575575 |  |  |  |  |
| 1.298074 | 0.575529 |  |  |  |  |
| 1.298281 | 0.57546 |  |  |  |  |
| 1.298511 | 0.575552 |  |  |  |  |
| 1.298741 | 0.575667 |  |  |  |  |
| 1.298948 | 0.575805 |  |  |  |  |
| 1.299178 | 0.575644 |  |  |  |  |
| 1.299408 | 0.57569 |  |  |  |  |
| 1.299638 | 0.575851 |  |  |  |  |
| 1.299868 | 0.57592 |  |  |  |  |
| 1.300098 | 0.576012 |  |  |  |  |
| 1.300328 | 0.576058 |  |  |  |  |
| 1.300535 | 0.576035 |  |  |  |  |
| 1.300765 | 0.575713 |  |  |  |  |
| 1.300995 | 0.576334 |  |  |  |  |
| 1.301225 | 0.576104 |  |  |  |  |
| 1.301455 | 0.576058 |  |  |  |  |
| 1.301662 | 0.575989 |  |  |  |  |
| 1.301892 | 0.576104 |  |  |  |  |
| 1.302122 | 0.576196 |  |  |  |  |
| 1.302352 | 0.576288 |  |  |  |  |
| 1.302582 | 0.576242 |  |  |  |  |
| 1.302812 | 0.576472 |  |  |  |  |
| 1.303042 | 0.576219 |  |  |  |  |
| 1.303249 | 0.576242 |  |  |  |  |
| 1.303479 | 0.576265 |  |  |  |  |
| 1.303709 | 0.576357 |  |  |  |  |
| 1.303916 | 0.576587 |  |  |  |  |
| 1.304169 | 0.576426 |  |  |  |  |
| 1.304399 | 0.576288 |  |  |  |  |
| 1.304606 | 0.576564 |  |  |  |  |
| 1.304836 | 0.576656 |  |  |  |  |
| 1.305066 | 0.576564 |  |  |  |  |
| 1.305296 | 0.576472 |  |  |  |  |
| 1.305503 | 0.576633 |  |  |  |  |
| 1.305733 | 0.576679 |  |  |  |  |
| 1.305986 | 0.57684 |  |  |  |  |
| 1.306193 | 0.576771 |  |  |  |  |
| 1.306423 | 0.576794 |  |  |  |  |
| 1.306653 | 0.576978 |  |  |  |  |
| 1.30686 | 0.576978 |  |  |  |  |
| 1.30709 | 0.576909 |  |  |  |  |
| 1.30732 | 0.576886 |  |  |  |  |
| 1.30755 | 0.576932 |  |  |  |  |
| 1.30778 | 0.577116 |  |  |  |  |
| 1.30801 | 0.577139 |  |  |  |  |
| 1.308217 | 0.577116 |  |  |  |  |
| 1.308447 | 0.577185 |  |  |  |  |
| 1.308677 | 0.577185 |  |  |  |  |
| 1.308907 | 0.577254 |  |  |  |  |
| 1.309137 | 0.576725 |  |  |  |  |
| 1.309367 | 0.577093 |  |  |  |  |
| 1.309574 | 0.577323 |  |  |  |  |
| 1.309804 | 0.577484 |  |  |  |  |
| 1.310034 | 0.577323 |  |  |  |  |
| 1.310264 | 0.577231 |  |  |  |  |
| 1.310494 | 0.577231 |  |  |  |  |
| 1.310724 | 0.577714 |  |  |  |  |
| 1.310954 | 0.577369 |  |  |  |  |
| 1.311161 | 0.577254 |  |  |  |  |
| 1.311391 | 0.577668 |  |  |  |  |
| 1.311621 | 0.577576 |  |  |  |  |
| 1.311828 | 0.577622 |  |  |  |  |
| 1.312081 | 0.577392 |  |  |  |  |
| 1.312311 | 0.577346 |  |  |  |  |
| 1.312518 | 0.578013 |  |  |  |  |
| 1.312748 | 0.578128 |  |  |  |  |
| 1.312978 | 0.577691 |  |  |  |  |
| 1.313185 | 0.577599 |  |  |  |  |
| 1.313415 | 0.577875 |  |  |  |  |
| 1.313668 | 0.577944 |  |  |  |  |
| 1.313875 | 0.577668 |  |  |  |  |
| 1.314105 | 0.577599 |  |  |  |  |
| 1.314335 | 0.577783 |  |  |  |  |
| 1.314542 | 0.577875 |  |  |  |  |
| 1.314772 | 0.578174 |  |  |  |  |
| 1.315002 | 0.577898 |  |  |  |  |
| 1.315232 | 0.577944 |  |  |  |  |
| 1.315462 | 0.57799 |  |  |  |  |
| 1.315692 | 0.578312 |  |  |  |  |
| 1.315922 | 0.577852 |  |  |  |  |
| 1.316129 | 0.578082 |  |  |  |  |
| 1.316359 | 0.578243 |  |  |  |  |
| 1.316589 | 0.578335 |  |  |  |  |
| 1.316819 | 0.578128 |  |  |  |  |
| 1.317049 | 0.578082 |  |  |  |  |
| 1.317279 | 0.578473 |  |  |  |  |
| 1.317486 | 0.578473 |  |  |  |  |
| 1.317716 | 0.578496 |  |  |  |  |
| 1.317946 | 0.578496 |  |  |  |  |
| 1.318176 | 0.578703 |  |  |  |  |
| 1.318406 | 0.578749 |  |  |  |  |
| 1.318636 | 0.579094 |  |  |  |  |
| 1.318866 | 0.578818 |  |  |  |  |
| 1.319073 | 0.578634 |  |  |  |  |
| 1.319303 | 0.578749 |  |  |  |  |
| 1.31951 | 0.578657 |  |  |  |  |
| 1.319763 | 0.578887 |  |  |  |  |
| 1.319993 | 0.578565 |  |  |  |  |
| 1.320223 | 0.578795 |  |  |  |  |
| 1.32043 | 0.578933 |  |  |  |  |
| 1.32066 | 0.579071 |  |  |  |  |
| 1.32089 | 0.579094 |  |  |  |  |
| 1.321097 | 0.578933 |  |  |  |  |
| 1.32135 | 0.579324 |  |  |  |  |
| 1.32158 | 0.579048 |  |  |  |  |
| 1.321787 | 0.57891 |  |  |  |  |
| 1.322017 | 0.578979 |  |  |  |  |
| 1.322247 | 0.579117 |  |  |  |  |
| 1.322454 | 0.579163 |  |  |  |  |
| 1.322684 | 0.579278 |  |  |  |  |
| 1.322937 | 0.579094 |  |  |  |  |
| 1.323144 | 0.579439 |  |  |  |  |
| 1.323374 | 0.579278 |  |  |  |  |
| 1.323604 | 0.579278 |  |  |  |  |
| 1.323811 | 0.579232 |  |  |  |  |
| 1.324041 | 0.5796 |  |  |  |  |
| 1.324271 | 0.579577 |  |  |  |  |
| 1.324501 | 0.579462 |  |  |  |  |
| 1.324731 | 0.579462 |  |  |  |  |
| 1.324961 | 0.579531 |  |  |  |  |
| 1.325191 | 0.579715 |  |  |  |  |
| 1.325398 | 0.579439 |  |  |  |  |
| 1.325628 | 0.579577 |  |  |  |  |
| 1.325858 | 0.579416 |  |  |  |  |
| 1.326088 | 0.58006 |  |  |  |  |
| 1.326318 | 0.579646 |  |  |  |  |
| 1.326548 | 0.579715 |  |  |  |  |
| 1.326755 | 0.579554 |  |  |  |  |
| 1.326985 | 0.579853 |  |  |  |  |
| 1.327215 | 0.579991 |  |  |  |  |
| 1.327445 | 0.579968 |  |  |  |  |
| 1.327675 | 0.579899 |  |  |  |  |
| 1.327905 | 0.58006 |  |  |  |  |
| 1.328112 | 0.579968 |  |  |  |  |
| 1.328342 | 0.580198 |  |  |  |  |
| 1.328572 | 0.579784 |  |  |  |  |
| 1.328779 | 0.580083 |  |  |  |  |
| 1.329032 | 0.580152 |  |  |  |  |
| 1.329262 | 0.580129 |  |  |  |  |
| 1.329492 | 0.580014 |  |  |  |  |
| 1.329699 | 0.580198 |  |  |  |  |
| 1.329929 | 0.580313 |  |  |  |  |
| 1.330159 | 0.580474 |  |  |  |  |
| 1.330366 | 0.580221 |  |  |  |  |
| 1.330596 | 0.580405 |  |  |  |  |
| 1.330849 | 0.580175 |  |  |  |  |
| 1.331056 | 0.580336 |  |  |  |  |
| 1.331286 | 0.580382 |  |  |  |  |
| 1.331516 | 0.580198 |  |  |  |  |
| 1.331723 | 0.580451 |  |  |  |  |
| 1.331953 | 0.580566 |  |  |  |  |
| 1.332183 | 0.580681 |  |  |  |  |
| 1.332436 | 0.580612 |  |  |  |  |
| 1.332643 | 0.580497 |  |  |  |  |
| 1.332873 | 0.580612 |  |  |  |  |
| 1.33308 | 0.580934 |  |  |  |  |
| 1.33331 | 0.580704 |  |  |  |  |
| 1.33354 | 0.580658 |  |  |  |  |
| 1.33377 | 0.580842 |  |  |  |  |
| 1.334 | 0.58075 |  |  |  |  |
| 1.33423 | 0.580865 |  |  |  |  |
| 1.334437 | 0.581026 |  |  |  |  |
| 1.334667 | 0.580842 |  |  |  |  |
| 1.334897 | 0.581187 |  |  |  |  |
| 1.335127 | 0.580934 |  |  |  |  |
| 1.335357 | 0.580819 |  |  |  |  |
| 1.335587 | 0.581164 |  |  |  |  |
| 1.335817 | 0.581187 |  |  |  |  |
| 1.336024 | 0.581233 |  |  |  |  |
| 1.336254 | 0.581072 |  |  |  |  |
| 1.336484 | 0.581164 |  |  |  |  |
| 1.336691 | 0.58144 |  |  |  |  |
| 1.336944 | 0.58144 |  |  |  |  |
| 1.337174 | 0.58121 |  |  |  |  |
| 1.337381 | 0.581371 |  |  |  |  |
| 1.337611 | 0.581394 |  |  |  |  |
| 1.337841 | 0.581624 |  |  |  |  |
| 1.338048 | 0.581555 |  |  |  |  |
| 1.338278 | 0.581371 |  |  |  |  |
| 1.338531 | 0.581394 |  |  |  |  |
| 1.338738 | 0.581578 |  |  |  |  |
| 1.338968 | 0.581831 |  |  |  |  |
| 1.339198 | 0.581739 |  |  |  |  |
| 1.339405 | 0.581739 |  |  |  |  |
| 1.339635 | 0.581693 |  |  |  |  |
| 1.339865 | 0.581808 |  |  |  |  |
| 1.340118 | 0.58144 |  |  |  |  |
| 1.340325 | 0.581486 |  |  |  |  |
| 1.340555 | 0.582153 |  |  |  |  |
| 1.340785 | 0.582038 |  |  |  |  |
| 1.340992 | 0.581785 |  |  |  |  |
| 1.341222 | 0.581877 |  |  |  |  |
| 1.341452 | 0.581785 |  |  |  |  |
| 1.341682 | 0.582222 |  |  |  |  |
| 1.341912 | 0.582084 |  |  |  |  |
| 1.342142 | 0.581831 |  |  |  |  |
| 1.342349 | 0.58213 |  |  |  |  |
| 1.342579 | 0.582199 |  |  |  |  |
| 1.342809 | 0.582291 |  |  |  |  |
| 1.343039 | 0.582176 |  |  |  |  |
| 1.343269 | 0.582222 |  |  |  |  |
| 1.343499 | 0.58236 |  |  |  |  |
| 1.343706 | 0.582429 |  |  |  |  |
| 1.343936 | 0.58236 |  |  |  |  |
| 1.344166 | 0.58213 |  |  |  |  |
| 1.344373 | 0.582268 |  |  |  |  |
| 1.344626 | 0.582475 |  |  |  |  |
| 1.344856 | 0.582337 |  |  |  |  |
| 1.345063 | 0.582314 |  |  |  |  |
| 1.345293 | 0.582153 |  |  |  |  |
| 1.345523 | 0.582613 |  |  |  |  |
| 1.345753 | 0.58259 |  |  |  |  |
| 1.34596 | 0.582406 |  |  |  |  |
| 1.346213 | 0.582521 |  |  |  |  |
| 1.346443 | 0.582682 |  |  |  |  |
| 1.34665 | 0.582613 |  |  |  |  |
| 1.34688 | 0.582981 |  |  |  |  |
| 1.34711 | 0.582774 |  |  |  |  |
| 1.347317 | 0.58305 |  |  |  |  |
| 1.347547 | 0.582981 |  |  |  |  |
| 1.3478 | 0.582751 |  |  |  |  |
| 1.34803 | 0.582935 |  |  |  |  |
| 1.348237 | 0.582774 |  |  |  |  |
| 1.348467 | 0.583096 |  |  |  |  |
| 1.348697 | 0.582774 |  |  |  |  |
| 1.348904 | 0.582912 |  |  |  |  |
| 1.349134 | 0.583165 |  |  |  |  |
| 1.349364 | 0.583119 |  |  |  |  |
| 1.349594 | 0.582866 |  |  |  |  |
| 1.349824 | 0.583188 |  |  |  |  |
| 1.350031 | 0.582935 |  |  |  |  |
| 1.350261 | 0.583464 |  |  |  |  |
| 1.350491 | 0.583303 |  |  |  |  |
| 1.350721 | 0.583234 |  |  |  |  |
| 1.350951 | 0.583303 |  |  |  |  |
| 1.351181 | 0.583119 |  |  |  |  |
| 1.351411 | 0.583464 |  |  |  |  |
| 1.351618 | 0.583418 |  |  |  |  |
| 1.351848 | 0.583418 |  |  |  |  |
| 1.352078 | 0.583418 |  |  |  |  |
| 1.352308 | 0.583786 |  |  |  |  |
| 1.352538 | 0.583625 |  |  |  |  |
| 1.352768 | 0.583671 |  |  |  |  |
| 1.352975 | 0.583441 |  |  |  |  |
| 1.353205 | 0.583694 |  |  |  |  |
| 1.353435 | 0.583625 |  |  |  |  |
| 1.353665 | 0.583694 |  |  |  |  |
| 1.353872 | 0.583671 |  |  |  |  |
| 1.354125 | 0.583694 |  |  |  |  |
| 1.354355 | 0.583901 |  |  |  |  |
| 1.354562 | 0.583878 |  |  |  |  |
| 1.354792 | 0.583786 |  |  |  |  |
| 1.355022 | 0.584085 |  |  |  |  |
| 1.355229 | 0.583947 |  |  |  |  |
| 1.355459 | 0.584315 |  |  |  |  |
| 1.355712 | 0.583993 |  |  |  |  |
| 1.355919 | 0.584131 |  |  |  |  |
| 1.356149 | 0.584223 |  |  |  |  |
| 1.356379 | 0.584223 |  |  |  |  |
| 1.356586 | 0.584269 |  |  |  |  |
| 1.356816 | 0.584292 |  |  |  |  |
| 1.357046 | 0.58443 |  |  |  |  |
| 1.357276 | 0.584292 |  |  |  |  |
| 1.357506 | 0.584315 |  |  |  |  |
| 1.357736 | 0.584453 |  |  |  |  |
| 1.357943 | 0.584292 |  |  |  |  |
| 1.358173 | 0.584499 |  |  |  |  |
| 1.358403 | 0.584407 |  |  |  |  |
| 1.358633 | 0.584338 |  |  |  |  |
| 1.358863 | 0.584453 |  |  |  |  |
| 1.359093 | 0.584913 |  |  |  |  |
| 1.359323 | 0.584476 |  |  |  |  |
| 1.35953 | 0.584614 |  |  |  |  |
| 1.35976 | 0.584637 |  |  |  |  |
| 1.35999 | 0.58489 |  |  |  |  |
| 1.36022 | 0.584568 |  |  |  |  |
| 1.36045 | 0.584637 |  |  |  |  |
| 1.36068 | 0.584821 |  |  |  |  |
| 1.360887 | 0.584821 |  |  |  |  |
| 1.361117 | 0.584959 |  |  |  |  |
| 1.361347 | 0.584706 |  |  |  |  |
| 1.361577 | 0.584913 |  |  |  |  |
| 1.361807 | 0.585097 |  |  |  |  |
| 1.362037 | 0.584936 |  |  |  |  |
| 1.362244 | 0.585235 |  |  |  |  |
| 1.362474 | 0.584913 |  |  |  |  |
| 1.362704 | 0.584867 |  |  |  |  |
| 1.362911 | 0.584867 |  |  |  |  |
| 1.363141 | 0.585074 |  |  |  |  |
| 1.363394 | 0.584982 |  |  |  |  |
| 1.363601 | 0.585235 |  |  |  |  |
| 1.363831 | 0.585097 |  |  |  |  |
| 1.364061 | 0.585051 |  |  |  |  |
| 1.364291 | 0.585235 |  |  |  |  |
| 1.364498 | 0.58512 |  |  |  |  |
| 1.364728 | 0.585235 |  |  |  |  |
| 1.364981 | 0.585396 |  |  |  |  |
| 1.365188 | 0.58535 |  |  |  |  |
| 1.365418 | 0.585304 |  |  |  |  |
| 1.365648 | 0.585166 |  |  |  |  |
| 1.365855 | 0.585557 |  |  |  |  |
| 1.366085 | 0.585396 |  |  |  |  |
| 1.366315 | 0.585511 |  |  |  |  |
| 1.366545 | 0.585511 |  |  |  |  |
| 1.366775 | 0.585741 |  |  |  |  |
| 1.367005 | 0.585718 |  |  |  |  |
| 1.367235 | 0.585557 |  |  |  |  |
| 1.367442 | 0.585672 |  |  |  |  |
| 1.367672 | 0.585718 |  |  |  |  |
| 1.367902 | 0.585695 |  |  |  |  |
| 1.368132 | 0.585718 |  |  |  |  |
| 1.368362 | 0.58581 |  |  |  |  |
| 1.368592 | 0.585764 |  |  |  |  |
| 1.368799 | 0.58581 |  |  |  |  |
| 1.369029 | 0.585948 |  |  |  |  |
| 1.369259 | 0.585764 |  |  |  |  |
| 1.369489 | 0.585902 |  |  |  |  |
| 1.369719 | 0.585971 |  |  |  |  |
| 1.369949 | 0.585856 |  |  |  |  |
| 1.370156 | 0.58581 |  |  |  |  |
| 1.370386 | 0.586017 |  |  |  |  |
| 1.370616 | 0.586477 |  |  |  |  |
| 1.370823 | 0.586178 |  |  |  |  |
| 1.371076 | 0.58604 |  |  |  |  |
| 1.371306 | 0.586178 |  |  |  |  |
| 1.371513 | 0.586408 |  |  |  |  |
| 1.371743 | 0.586316 |  |  |  |  |
| 1.371973 | 0.5865 |  |  |  |  |
| 1.37218 | 0.586224 |  |  |  |  |
| 1.37241 | 0.586132 |  |  |  |  |
| 1.37264 | 0.586523 |  |  |  |  |
| 1.372893 | 0.586477 |  |  |  |  |
| 1.3731 | 0.586178 |  |  |  |  |
| 1.37333 | 0.5865 |  |  |  |  |
| 1.37356 | 0.586569 |  |  |  |  |
| 1.373767 | 0.586707 |  |  |  |  |
| 1.373997 | 0.586592 |  |  |  |  |
| 1.374227 | 0.586339 |  |  |  |  |
| 1.374457 | 0.586684 |  |  |  |  |
| 1.374687 | 0.586615 |  |  |  |  |
| 1.374917 | 0.586546 |  |  |  |  |
| 1.375124 | 0.586891 |  |  |  |  |
| 1.375354 | 0.586822 |  |  |  |  |
| 1.375584 | 0.587121 |  |  |  |  |
| 1.375814 | 0.586776 |  |  |  |  |
| 1.376044 | 0.586615 |  |  |  |  |
| 1.376274 | 0.586799 |  |  |  |  |
| 1.376481 | 0.587167 |  |  |  |  |
| 1.376711 | 0.586868 |  |  |  |  |
| 1.376941 | 0.586937 |  |  |  |  |
| 1.377148 | 0.58696 |  |  |  |  |
| 1.377401 | 0.587328 |  |  |  |  |
| 1.377631 | 0.587443 |  |  |  |  |
| 1.377861 | 0.587121 |  |  |  |  |
| 1.378068 | 0.587121 |  |  |  |  |
| 1.378298 | 0.587466 |  |  |  |  |
| 1.378528 | 0.587581 |  |  |  |  |
| 1.378735 | 0.587259 |  |  |  |  |
| 1.378988 | 0.587167 |  |  |  |  |
| 1.379218 | 0.587121 |  |  |  |  |
| 1.379425 | 0.587512 |  |  |  |  |
| 1.379655 | 0.587213 |  |  |  |  |
| 1.379885 | 0.587374 |  |  |  |  |
| 1.380092 | 0.587443 |  |  |  |  |
| 1.380322 | 0.58765 |  |  |  |  |
| 1.380575 | 0.587535 |  |  |  |  |
| 1.380782 | 0.587673 |  |  |  |  |
| 1.381012 | 0.587282 |  |  |  |  |
| 1.381219 | 0.587581 |  |  |  |  |
| 1.381449 | 0.587512 |  |  |  |  |
| 1.381679 | 0.587673 |  |  |  |  |
| 1.381909 | 0.587742 |  |  |  |  |
| 1.382139 | 0.587834 |  |  |  |  |
| 1.382369 | 0.588179 |  |  |  |  |
| 1.382576 | 0.587811 |  |  |  |  |
| 1.382806 | 0.587512 |  |  |  |  |
| 1.383036 | 0.587558 |  |  |  |  |
| 1.383243 | 0.588018 |  |  |  |  |
| 1.383496 | 0.587949 |  |  |  |  |
| 1.383726 | 0.587788 |  |  |  |  |
| 1.383933 | 0.587604 |  |  |  |  |
| 1.384163 | 0.588018 |  |  |  |  |
| 1.384393 | 0.587903 |  |  |  |  |
| 1.384623 | 0.588156 |  |  |  |  |
| 1.38483 | 0.587765 |  |  |  |  |
| 1.385083 | 0.58811 |  |  |  |  |
| 1.38529 | 0.587972 |  |  |  |  |
| 1.38552 | 0.587972 |  |  |  |  |
| 1.38575 | 0.588156 |  |  |  |  |
| 1.38598 | 0.588179 |  |  |  |  |
| 1.386187 | 0.588248 |  |  |  |  |
| 1.386417 | 0.588202 |  |  |  |  |
| 1.38667 | 0.588133 |  |  |  |  |
| 1.386877 | 0.588202 |  |  |  |  |
| 1.387107 | 0.58834 |  |  |  |  |
| 1.387337 | 0.588225 |  |  |  |  |
| 1.387544 | 0.588455 |  |  |  |  |
| 1.387774 | 0.588294 |  |  |  |  |
| 1.388004 | 0.588271 |  |  |  |  |
| 1.388234 | 0.588478 |  |  |  |  |
| 1.388464 | 0.58834 |  |  |  |  |
| 1.388694 | 0.588317 |  |  |  |  |
| 1.388901 | 0.588409 |  |  |  |  |
| 1.389131 | 0.588662 |  |  |  |  |
| 1.389361 | 0.588455 |  |  |  |  |
| 1.389591 | 0.588708 |  |  |  |  |
| 1.389821 | 0.58857 |  |  |  |  |
| 1.390051 | 0.588777 |  |  |  |  |
| 1.390281 | 0.588708 |  |  |  |  |
| 1.390488 | 0.588639 |  |  |  |  |
| 1.390718 | 0.588754 |  |  |  |  |
| 1.390948 | 0.588915 |  |  |  |  |
| 1.391178 | 0.588846 |  |  |  |  |
| 1.391408 | 0.588915 |  |  |  |  |
| 1.391615 | 0.588961 |  |  |  |  |
| 1.391845 | 0.589053 |  |  |  |  |
| 1.392075 | 0.589168 |  |  |  |  |
| 1.392305 | 0.588938 |  |  |  |  |
| 1.392512 | 0.589053 |  |  |  |  |
| 1.392765 | 0.589099 |  |  |  |  |
| 1.392995 | 0.589053 |  |  |  |  |
| 1.393202 | 0.589421 |  |  |  |  |
| 1.393432 | 0.589237 |  |  |  |  |
| 1.393662 | 0.589145 |  |  |  |  |
| 1.393869 | 0.58926 |  |  |  |  |
| 1.394099 | 0.589007 |  |  |  |  |
| 1.394352 | 0.589076 |  |  |  |  |
| 1.394559 | 0.588984 |  |  |  |  |
| 1.394789 | 0.589329 |  |  |  |  |
| 1.395019 | 0.589421 |  |  |  |  |
| 1.395249 | 0.589214 |  |  |  |  |
| 1.395456 | 0.589329 |  |  |  |  |
| 1.395686 | 0.589628 |  |  |  |  |
| 1.395916 | 0.589697 |  |  |  |  |
| 1.396146 | 0.589444 |  |  |  |  |
| 1.396376 | 0.589283 |  |  |  |  |
| 1.396606 | 0.589651 |  |  |  |  |
| 1.396813 | 0.589513 |  |  |  |  |
| 1.397043 | 0.589697 |  |  |  |  |
| 1.397273 | 0.589329 |  |  |  |  |
| 1.397503 | 0.58972 |  |  |  |  |
| 1.397733 | 0.589743 |  |  |  |  |
| 1.397963 | 0.589766 |  |  |  |  |
| 1.39817 | 0.58972 |  |  |  |  |
| 1.3984 | 0.589697 |  |  |  |  |
| 1.39863 | 0.589835 |  |  |  |  |
| 1.39886 | 0.589881 |  |  |  |  |
| 1.39909 | 0.589858 |  |  |  |  |
| 1.39932 | 0.589927 |  |  |  |  |
| 1.399527 | 0.589996 |  |  |  |  |
| 1.399757 | 0.590249 |  |  |  |  |
| 1.399987 | 0.590042 |  |  |  |  |
| 1.400194 | 0.589904 |  |  |  |  |
| 1.400424 | 0.589927 |  |  |  |  |
| 1.400677 | 0.590479 |  |  |  |  |
| 1.400907 | 0.59018 |  |  |  |  |
| 1.401114 | 0.590272 |  |  |  |  |
| 1.401344 | 0.590111 |  |  |  |  |
| 1.401574 | 0.590364 |  |  |  |  |
| 1.401781 | 0.590088 |  |  |  |  |
| 1.402011 | 0.590157 |  |  |  |  |
| 1.402264 | 0.589973 |  |  |  |  |
| 1.402471 | 0.590226 |  |  |  |  |
| 1.402701 | 0.590088 |  |  |  |  |
| 1.402931 | 0.590387 |  |  |  |  |
| 1.403138 | 0.590226 |  |  |  |  |
| 1.403368 | 0.590364 |  |  |  |  |
| 1.403598 | 0.59041 |  |  |  |  |
| 1.403851 | 0.590548 |  |  |  |  |
| 1.404058 | 0.590249 |  |  |  |  |
| 1.404288 | 0.590295 |  |  |  |  |
| 1.404495 | 0.590525 |  |  |  |  |
| 1.404725 | 0.590594 |  |  |  |  |
| 1.404955 | 0.590479 |  |  |  |  |
| 1.405185 | 0.590755 |  |  |  |  |
| 1.405415 | 0.590732 |  |  |  |  |
| 1.405645 | 0.590824 |  |  |  |  |
| 1.405875 | 0.590801 |  |  |  |  |
| 1.406082 | 0.590617 |  |  |  |  |
| 1.406312 | 0.590801 |  |  |  |  |
| 1.406542 | 0.590801 |  |  |  |  |
| 1.406772 | 0.59087 |  |  |  |  |
| 1.407002 | 0.59064 |  |  |  |  |
| 1.407232 | 0.590962 |  |  |  |  |
| 1.407439 | 0.591353 |  |  |  |  |
| 1.407669 | 0.590985 |  |  |  |  |
| 1.407899 | 0.590939 |  |  |  |  |
| 1.408106 | 0.590962 |  |  |  |  |
| 1.408359 | 0.590801 |  |  |  |  |
| 1.408589 | 0.590801 |  |  |  |  |
| 1.408796 | 0.590847 |  |  |  |  |
| 1.409026 | 0.591031 |  |  |  |  |
| 1.409256 | 0.591238 |  |  |  |  |
| 1.409463 | 0.591031 |  |  |  |  |
| 1.409693 | 0.591077 |  |  |  |  |
| 1.409946 | 0.590985 |  |  |  |  |
| 1.410153 | 0.591215 |  |  |  |  |
| 1.410383 | 0.591376 |  |  |  |  |
| 1.410613 | 0.591123 |  |  |  |  |
| 1.41082 | 0.591008 |  |  |  |  |
| 1.41105 | 0.59133 |  |  |  |  |
| 1.41128 | 0.591445 |  |  |  |  |
| 1.411533 | 0.591514 |  |  |  |  |
| 1.41174 | 0.591215 |  |  |  |  |
| 1.41197 | 0.591284 |  |  |  |  |
| 1.4122 | 0.591468 |  |  |  |  |
| 1.412407 | 0.591537 |  |  |  |  |
| 1.412637 | 0.591399 |  |  |  |  |
| 1.412867 | 0.591514 |  |  |  |  |
| 1.413097 | 0.591675 |  |  |  |  |
| 1.413327 | 0.591721 |  |  |  |  |
| 1.413557 | 0.591813 |  |  |  |  |
| 1.413787 | 0.591537 |  |  |  |  |
| 1.413994 | 0.591537 |  |  |  |  |
| 1.414224 | 0.591882 |  |  |  |  |
| 1.414454 | 0.591836 |  |  |  |  |
| 1.414684 | 0.591606 |  |  |  |  |
| 1.414914 | 0.591721 |  |  |  |  |
| 1.415144 | 0.591882 |  |  |  |  |
| 1.415351 | 0.591928 |  |  |  |  |
| 1.415581 | 0.591675 |  |  |  |  |
| 1.415811 | 0.591514 |  |  |  |  |
| 1.416041 | 0.591951 |  |  |  |  |
| 1.416271 | 0.592043 |  |  |  |  |
| 1.416501 | 0.591836 |  |  |  |  |
| 1.416708 | 0.59202 |  |  |  |  |
| 1.416938 | 0.592043 |  |  |  |  |
| 1.417168 | 0.592089 |  |  |  |  |
| 1.417375 | 0.592043 |  |  |  |  |
| 1.417628 | 0.592066 |  |  |  |  |
| 1.417858 | 0.591928 |  |  |  |  |
| 1.418065 | 0.592089 |  |  |  |  |
| 1.418295 | 0.592227 |  |  |  |  |
| 1.418525 | 0.592181 |  |  |  |  |
| 1.418732 | 0.59202 |  |  |  |  |
| 1.418962 | 0.592158 |  |  |  |  |
| 1.419192 | 0.592388 |  |  |  |  |
| 1.419445 | 0.592043 |  |  |  |  |
| 1.419652 | 0.592089 |  |  |  |  |
| 1.419882 | 0.592457 |  |  |  |  |
| 1.420112 | 0.592296 |  |  |  |  |
| 1.420319 | 0.592089 |  |  |  |  |
| 1.420549 | 0.592526 |  |  |  |  |
| 1.420779 | 0.592434 |  |  |  |  |
| 1.421009 | 0.592411 |  |  |  |  |
| 1.421239 | 0.592434 |  |  |  |  |
| 1.421469 | 0.592342 |  |  |  |  |
| 1.421676 | 0.592595 |  |  |  |  |
| 1.421906 | 0.592411 |  |  |  |  |
| 1.422136 | 0.59248 |  |  |  |  |
| 1.422366 | 0.592434 |  |  |  |  |
| 1.422596 | 0.592572 |  |  |  |  |
| 1.422826 | 0.592595 |  |  |  |  |
| 1.423033 | 0.59271 |  |  |  |  |
| 1.423263 | 0.592572 |  |  |  |  |
| 1.423493 | 0.592526 |  |  |  |  |
| 1.423723 | 0.592618 |  |  |  |  |
| 1.423953 | 0.592687 |  |  |  |  |
| 1.424183 | 0.592894 |  |  |  |  |
| 1.42439 | 0.592802 |  |  |  |  |
| 1.42462 | 0.592618 |  |  |  |  |
| 1.42485 | 0.592871 |  |  |  |  |
| 1.42508 | 0.592986 |  |  |  |  |
| 1.425287 | 0.592687 |  |  |  |  |
| 1.42554 | 0.592871 |  |  |  |  |
| 1.42577 | 0.593124 |  |  |  |  |
[truncated: 31,736 more chars]
